# Semisynthesis of 5-*O*-ester derivatives of renieramycin T and their cytotoxicity against non-small-cell lung cancer cell lines

**DOI:** 10.1038/s41598-023-48526-2

**Published:** 2023-12-06

**Authors:** Koonchira Buaban, Bhurichaya Innets, Korrakod Petsri, Suwimon Sinsook, Pithi Chanvorachote, Chaisak Chansriniyom, Khanit Suwanborirux, Masashi Yokoya, Naoki Saito, Supakarn Chamni

**Affiliations:** 1 Department of Pharmacognosy and Pharmaceutical Botany, Faculty of Pharmaceutical Sciences, Chulalongkorn University, Bangkok, 10330 Thailand; 2 Natural Products and Nanoparticles Research Unit (NP2), Chulalongkorn University, Bangkok, 10330 Thailand; 3 Center of Excellence in Cancer Cell and Molecular Biology, Faculty of Pharmaceutical Sciences, Chulalongkorn University, Bangkok, 10330 Thailand; 4 Department of Pharmacology and Physiology, Faculty of Pharmaceutical Sciences, Chulalongkorn University, Bangkok, 10330 Thailand; 5 Pharmaceutical Sciences and Technology Program, Faculty of Pharmaceutical Sciences, Chulalongkorn University, Bangkok, 10330 Thailand; 6 Graduate School of Pharmaceutical Sciences, Meiji Pharmaceutical University, 2-522-1 Noshio, Kiyose, Tokyo, 204-8588 Japan

**Keywords:** Chemistry, Medicinal chemistry, Drug discovery and development, Drug discovery, Drug screening, Medicinal chemistry

## Abstract

The semisynthesis of 5-*O*-ester derivatives of renieramycin T was accomplished through the photoredox reaction of renieramycin M (**1**), a bistetrahydroisoquinolinequinone alkaloid isolated from the Thai blue sponge *Xestospongia* sp. This process led to the conversion of compound **1** to renieramycin T (**2**), which was subsequently subjected to Steglich esterification with appropriate acylating agents containing linear alkyl, *N*-*tert*-butoxycarbonyl-L-amino, and heterocyclic aromatic substituent. Notably, the one-pot transformation, combining the photoredox reaction and esterification led to the formation of 7-*O*-ester derivatives of renieramycin S due to hydrolysis. Subsequently, the in vitro cytotoxicity of the 17 semisynthesized derivatives against human non-small-cell lung cancer (NSCLC) cells in parallel with normal cell lines was evaluated. Among the tested compounds, 5-*O*-(3-propanoyl) ester of renieramycin T (**3b**) exhibited potent cytotoxic activity with half-maximal inhibitory concentration (IC_50_) values at 33.44 and 33.88 nM against H292 and H460 cell lines, respectively. These values were within the same range as compound **1** (IC_50_ = 34.43 and 35.63 nM) and displayed twofold higher cytotoxicity compared to compound **2** (IC_50_ = 72.85 and 83.95 nM). The steric characteristics and aromatic orientation of the 5-*O*-ester substituents played significant roles in their cytotoxicity. Notably, derivative **3b** induced apoptosis with minimal necrosis, in contrast to the parental compound **1**. Hence, the relationship between the structure and cytotoxicity of renieramycin–ecteinascidin hybrid alkaloids was investigated. This study emphasizes the potential of the series of 5-*O*-ester derivatives of renieramycin T as promising leads for the further development of potential anti-NSCLC agents.

## Introduction

Lung carcinoma represents a major public health problem that has become one of the leading causes of cancer incidence and mortality worldwide^1^. Behavioral risks such as smoking in conjunction with factors including age, gender, genetics, environment, and air pollution contribute as the main risk factors in the development of lung tumors^2–4^. Non-small-cell lung cancer (NSCLC) stands as the most prevalent type, accounting for 85% of all lung cancer cases^5^. Treatment approaches for NSCLC encompass chemotherapy, radiation therapy, immunotherapy, targeted therapy, and personalized therapy^5, 6^. However, due to poor prognosis, a low 5-year survival rate, and challenges related to drug resistance^5^, ongoing research efforts are devoted to the advancement of NSCLC treatment.

Marine natural products characterized by their distinctive chemical structures and biological activities have emerged as promising sources for potential anticancer drug leads^7–10^. Among these compounds, 1,2,3,4-tetrahydroisoquinoline (THIQ) alkaloids belonging to the saframycin family, including saframycins, renieramycins, safracins, ecteinascidins, and their synthetic derivatives, have demonstrated notable therapeutic impacts in chemotherapy^11, 12^. Trabectedin (or Ecteinascidin 743), which contains THIQ moieties as a fused-ring core structure, was isolated from the Caribbean tunicate *Ecteinascidia turbinata*, and received approval from the United States Food and Drug Administration in 2015 for the treatment of soft tissue sarcoma^13^. Moreover, lurbinectedin (PM01183), a tetrahydropyrroloquinoline analog of trabectedin exhibiting enhanced antitumor activity, was approved in 2020 as a the second-line treatment of metastatic small-cell lung cancer^14, 15^. Anticancer mechanism of trabectedin involves the DNA alkylation by iminium ion at C–21 to generate the permanent covalent bond at the N2 position of guanine within the DNA minor groove^16, 17^. Additionally, trabectedin has been reported to induce apoptosis in human anaplastic large cell lymphoma (JB6) cells through p53 and caspase 3 pathways^18^.

**Figure 1:**
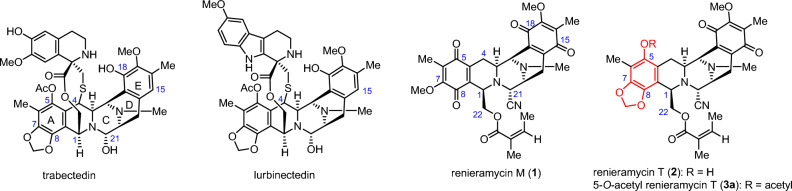
The structure of 1,2,3,4-tetrahydroisoquinoline and bistetrahydroisoquinolinequinone marine alkaloids.

Renieramycin M (**1**) and renieramycin T (**2**) are the THIQ products isolated from the Thai blue sponge *Xestospongia* sp. with **1** being the major compound and **2** the minor compound^19, 20^. Both compounds possess a fused pentacyclic core structure (ring A–E). Compound **1** is classified as a bistetrahydroisoquinolinequinone alkaloid, while compound **2** is categorized as a renieramycin–ecteinascidin hybrid alkaloid, featuring a 1,3-dioxole moiety on ring A, similar to the known chemotherapeutic drugs trabectedin and lurbinectedin (Fig. 1). Compound **2** was successfully semisynthesized from compound **1** through ambient-light-induced intramolecular cyclization in its marine natural habitat. This process was supported by studies demonstrating an efficient intramolecular photoredox reaction, which smoothly converted 7-methoxy-6-methyl-1,2,3,4-tetrahydroisoquinoline-5,8-dione moiety on ring A of compound **1** into a 5-hydroxy-tetrahydroisoquinol-1,3-dioxole moiety of compound **2** in excellent yields^21–24^.

Various studies have reported the structure–cytotoxicity relationship studies of compound **1** and its semisynthetic analogs against the metastatic human H292 and H460 NSCLC cell lines^25, 26^. The underlying mechanisms of anticancer activity for compound **1** involve the induction of apoptosis in lung cancer cells through the p53-dependent pathway^27, 28^, including mitochondria-dependent pathway^29^. Additionally, compound **1** has been found to suppress the levels of anti-apoptotic proteins, namely myeloid cell leukemia-1 (MCL-1), and sensitized anoikis-resistant lung cancer cells to anoikis^30^. Furthermore, compound **1** exhibits potent anti-metastatic property by inhibiting epithelial-to-mesenchymal transition (EMT) in lung cancer^31^, along with the suppression of lung cancer stem cell (CSC) markers^32^. In contrast, limited studies have been conducted on compound **2** and its semisynthetic analogs against NSCLC have not yet been deeply conducted because of its limited quantity in natural sources^20, 33^. Initially, the naturally derived **2** was chemically modified by esterification to obtain only two derivatives including 5-*O*-acetyl renieramycin T (**3a**)^34^ and 5-*O*-(*N*-Boc-L-alanine)-renieramycin T^35^ (Boc: *tert*-butoxycarbonyl). Pharmacological insights into the renieramycin–ecteinascidin hybrid alkaloids have provided valuable information regarding their anti-lung cancer properties. Compound **3a** induces cell death in H292 lung cancer cells through p53-dependent apoptosis, involving the suppression of the antiapoptotic B-cell lymphoma-2 protein, and reduction of the proapoptotic Bax protein^34^. Moreover, 5-*O*-(*N*-Boc-L-alanine)-renieramycin T induced spheroid formation and apoptosis in lung cancer cells, while inhibiting CSC signals by suppressing the Akt protein^35^.

Compounds **1**, **2**, and 5-*O*-acetyl renieramycin T (**3a**) have demonstrated potent cytotoxicity with nanomolar half-maximal inhibitory concentrations (IC_50_) against several human cancer cell lines, including colon, lung, pancreatic, and breast cacinomas^20^. Compound **1** demonstrates promising anticancer effects by inducing apoptosis in lung cancer cells via the p53-dependent pathway^27^. However, the presence of two quinone moieties, compound **1** induces accidental necrosis and increases the reactive oxygen species levels on the lung cancer H23 cell line. Nevertheless, a targeted modification aimed at one quinone group to form the 5-*O*-acetylated hydroquinone significantly diminishes the unintended necrotic effect of the parent compound **1** on lung cancer H23 cells^36^. Consequently, compound **1** has undergone chemical modifications, including hydrogenation, esterification, and air oxidation, to yield diverse 5-*O*-ester monohydroquinone analogs of renieramycin M, enabling the investigation of structure–cytotoxicity relationships in NSCLC^25, 26, 36–38^. Notably, compound **2**, an alkaloid possessing a tetrahydroisoquinolinequinone structure with a 1,3-dioxole motif on ring A similar to the chemotherapeutic drug trabectedin, predominantly induces apoptosis-mediated cell death^39^. These findings provide compelling evidence that modifying the renieramycin core structure to feature a solitary quinone group while incorporating the 1,3-dioxole motif on the ring A system holds significant potential in terms of its anti-lung cancer efficacy. Specifically, it promotes programmed cell death through apoptosis while reducing unintended necrosis.

Based on the reported data, a renieramycin–ecteinascidin hybrid alkaloid exhibits potent cytotoxicity, a unique anticancer mechanism devoid of unwanted toxicity, and benefits from a mild synthetic approach. Thus, in the present study, a novel series of 5-*O*-ester derivatives of **2** was semisynthesized by the intramolecular photoredox reaction of **1** followed by Steglich esterification with suitable acylating agents, including linear alkyl, *N*-*tert*-butyl-carbamate-containing amino acid, and heterocyclic aromatic substituents. Furthermore, a one-pot protocol for the synthesis of 7-*O*-ester derivatives of renieramycin S was investigated. Next, the in vitro cytotoxic of the 5-*O*-ester derivatives of **2** was evaluated against highly metastatic human NSCLC cell lines (H292 and H460) along with the normal cell lines including dermal papilla (DP), human keratinocyte (HaCaT) and non-tumorigenic bronchial epithelial (BEAS-2B) cell lines by using the 3-(4,5-dimethylthiazol-2-yl)-2,5-diphenyltetrazolium bromide (MTT) assay. In addition, compounds **1** and **3b** were preliminarily investigated the mechanisms of cell death using a Hoechst 33342 and propidium iodide (PI) nuclear co-staining assay. The effect of compounds **1** and **3b** on apoptosis and necrosis was confirmed by flow cytometric analysis using Annexin V FITC/PI double staining. Therefore, this study aims to elucidate the structure–cytotoxicity relationship of compound **2** and its semisynthetic derivatives, which can contribute to the development of biologically active tetrahydroisoquinoline marine natural products as potential cytotoxic agents.

## Results and discussion

### Synthesis of 5-*O*-ester derivatives of renieramycin T (3a–3o) and 7-*O*-ester derivatives of renieramycin S (4a and 4b)

**Figure 2:**
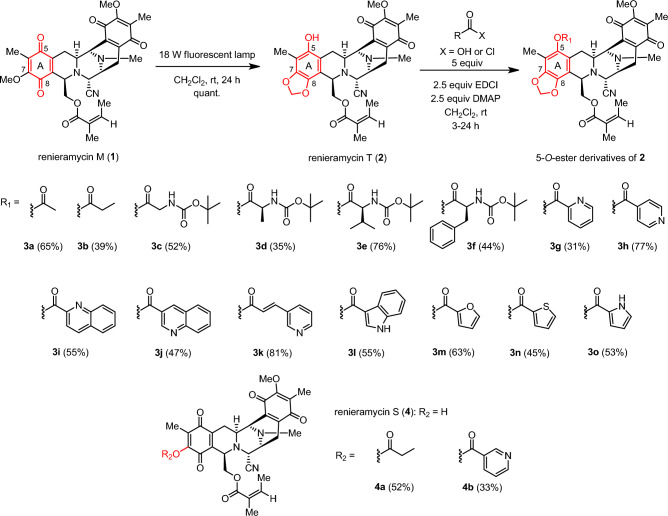
Semisynthesis of 5-*O*-ester derivatives of renieramycin T.

Compound **1** was isolated from *Xestospongia* sp. collected from Si-Chang Island, Thailand, using a previously reported protocol. The extraction process involved pretreatment with a 10% potassium cyanide solution in a pH 7 phosphate buffer, followed by methanolic extraction^19^. The resulting compound **1** was obtained as a stable orange solid and served as a precursor for the mild and regioselective semisynthesis of **2** and its ester derivatives. This semisynthesis involved a two-step chemical modification process consisting of intramolecular photoredox transformation and Steglich esterification (Fig. 2). The naturally derived **1** was irradiated by an 18 W fluorescent lamp for 24 h to form a 1,3-dioxole moiety at C–7 and C–8 via light-induced radical formation, followed by intramolecular cyclization to obtain **2** in an excellent yield^21–24^.

Without purification to avoid unexpected hydrolysis, compound **2** was further reacted with a suitable acylating agent in the presence of 1-ethyl-3-(3-dimethylaminopropyl)carbodiimide (EDCI), a water-soluble carbodiimide coupling reagent, and 4-dimethylaminopyridine (DMAP), a nucleophilic base catalyst, for the Steglich esterification^40^ to produce a new series of 5-*O*-ester derivatives having linear alkyl (**3a** and **3b**), *N*-Boc-L-amino (**3c**–**3f**), and heterocyclic aromatic (**3g**–**3o**) esters. Overall, the chemical modification of **1** into 15 ester derivatives of **2** proceeded smoothly, with acceptable to good yields (31–81%, based on **2** recovery). The reactivity of the acylating agents was controlled by their steric and electronic properties. The chemical transformation was improved by increasing the equivalents of EDCI and DMAP. Interestingly, performing photoredox reaction and Steglich esterification simultaneously as a one-pot procedure led to the transformation of **1** into 7-*O*-ester derivatives of renieramycin S^41^. The studies were conducted by using propionyl chloride and nicotinic acid as the acylating agent to obtain compounds **4a** and **4b**, respectively as the major products. The proposed mechanism involved hydrogen abstraction, electron transfer, hydrolysis, and esterification^24, 41^.

The chemical structures of the semisynthetic compounds (**3a–3o**, **4a**, and **4b**) were fully elucidated by extensive spectroscopic analyses, including nuclear magnetic resonance (NMR) spectroscopy, high-resolution mass spectroscopy, infrared (IR) spectroscopy, and electronic circular dichroism (ECD) (see Supporting Information). The characteristic proton chemical shifts of the 5-*O*-ester derivatives, specifically the methylenedioxy moiety at the newly formed 1,3-dioxole ring fused with ring A were observed as a pair of doublets at 6.01 ± 0.05 ppm. Moreover, the characteristic carbon chemical shifts (δ_C_) at the C–5 quaternary carbon, the OCH_2_O motif located between C–7 and C–8, and the carbonyl moiety of quinone at C–15 and C–18 on ring E appeared at 140.1 ± 0.8, 101.9 ± 0.02, 185.5 ± 0.4, and 182.4 ± 0.4 ppm, respectively. Note that the C–5 quaternary carbon signal of the 5-*O*-ester derivatives was shifted upfield compared to compound **2**, which contains a 5-hydroxyl group. The signals corresponding to the additional ester substituents were consistent with their respective chemical structures. The resulting ester carbonyl moiety at C–1′ showed signals at 163.7 ± 7.6 ppm.

**Figure 3:**
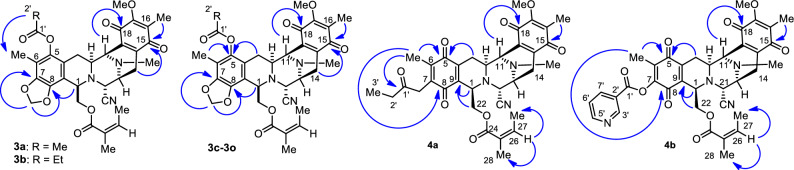
Heteronuclear multiple bond correlations (HMBCs) in the 5-*O*-ester derivatives of renieramycin T and 7-*O*-ester derivatives of renieramycin S.

Furthermore, the formation of the 1,3-dioxole motif at ring A was confirmed by heteronuclear multiple bond correlations (HMBCs) between the methylenedioxy proton and the aromatic carbons at C–7 and C–8, as well as between the methine proton at C–1 and the carbon signal from C–8 (Fig. 3). The presence of the additional ester motif at C–5 was supported by HMBCs between the proton signal of the acyl group and the carbon signal of the methyl group at C–6, which were detected in compounds **3a** and **3b**. Furthermore, compounds **4a** and **4b** showed carbon signals corresponding to four carbonyl moieties on bistetrahydroisoquinolinequinone (rings A and E) and the methoxy group at C–17, similar to **1**^19^ and renieramycin S^42^. The signals of the propanoyl and 3-pyridinecarbonyl ester substituents of **4a** and **4b** were observed, whereas the signals of the methylenedioxy group were absent. According to the HMBCs, the additional ester substituent at C–7 of **4a** was confirmed by the carbon signal of the carbonyl group (C–1′) and the proton signal of the methyl group at C–6. Besides, the long-range correlations were observed between the methylene proton at C–14 and the carbon at C–15, as well as between the methine proton at C–11 and the carbon at C–18, in all derivatives. These correlations indicated the presence of a quinone skeleton on ring E.

### Cytotoxic evaluation of 3a–3o, 4a, and 4b against NSCLC cell lines

The in vitro cytotoxicity of all the semisynthesized esters and their precursors against H292 and H460 human NSCLC cell lines was analyzed based on the mitochondrial dehydrogenase activity via the MTT assay^43^ (Table 1). The compounds were tested at doses of 1–250 nM. Cisplatin and doxorubicin, which are the standard chemotherapeutic drugs for the treatment of NSCLC, were used as positive controls. The series of 5-*O*-ester derivatives of renieramycin T, **3a**–**3o**, showed potent cytotoxicity in nanomolar concentrations against both cell lines. Moreover, the mother compound **1** exhibited strong cytotoxicity (IC_50_ of 34.43 ± 1.70 and 35.63 ± 1.82 nM against the H292 and H460 cell lines, respectively). However, the extended use of **1** is limited by the concerns for unwanted necrosis, which is an unprogrammed form of cell death caused by the presence of two quinone moieties^36^. Alkaloid **2**, which is a highly oxygenated aromatic compound containing a methylenedioxy bridge at ring A and a quinone moiety at ring E, exhibited IC_50_ values of 72.85 ± 2.88 and 83.95 ± 3.63 nM against the H292 and H460 cell lines, respectively. Although it showed approximately a twofold weaker cytotoxicity than compound **1**, the further investigation of 5-*O*-ester derivatives of renieramycin T would provide an initial structure–cytotoxicity relationship study to investigate the promising cytotoxic agents designed to diminish unwanted toxicity. Among the derivatives having 5-*O*-alkyl ester and 5-*O*-amino ester substituents, compounds **3a**–**3d** possessed impressive cytotoxicity similar to **1** against the H292 (IC_50_ from 33.44 ± 0.87 to 43.43 ± 3.00 nM) and H460 (IC_50_ from 33.03 ± 1.55 to 41.63 ± 4.15 nM) cell lines. Derivatives **3b**, which contain 5-*O*-propanoyl ester substituent, displayed the topmost potent cytotoxicity with IC_50_ of 33.44 ± 0.87 and 33.88 ± 1.95 nM against the H292 and H460 cell lines, twofold stronger than that of **2**. According to the structure–cytotoxicity relationship data of the derivatives with amino ester substituents at C–5^37, 38^, the smaller steric substituents generally exhibited stronger cytotoxicity than the larger ones as the potency based on IC_50_ followed the order **3d** > **3e** > **3f**. The 5-*O*-heterocyclic aromatic ester derivatives (**3g**–**3o**) exhibited interesting cytotoxicity with nitrogen-containing aroyl substituents. Compound **3h**, with 4-pyridinecarbonyl ester, showed potent cytotoxicity, with IC_50_ of 35.27 ± 1.09 and 35.65 ± 1.64 nM against the H292 and H460 cell lines, respectively. Compound **3k**, containing 3-pyridineacryloyl ester, exhibited pre-eminent cytotoxicity, with IC_50_ of 36.29 ± 2.06 and 38.75 ± 7.25 nM against the H292 and H460 cell lines, respectively. Interestingly, 5-*O*-(2-quinolinecarbonyl) ester (**3i**) showed twofold stronger cytotoxicity than 5-*O*-(3-quinolinecarbonyl) ester (**3j**), although they are regioisomers. Compounds **3e** and **3j** displayed cytotoxic profiles similar to **2**. The five-membered heterocyclic ester derivatives **3l**–**3o**, containing indole, furan, thiophene, and pyrrole moieties, exhibited decreased cytotoxicity. Among them, 5-*O*-(2-thiophenecarbonyl) ester (**3n**) had the weakest cytotoxicity, with IC_50_ of 136.83 ± 6.30 and 122.83 ± 4.99 nM against the H292 and H460 cell lines, respectively. The IC_50_ values of the 5-*O*-ester derivatives **3a**, **3b**, **3d**, **3h**, **3i**, and **3k** were in the same range as the mother compound **1**. Slight reductions in cytotoxicity were observed with compounds **3c**, **3f**, **3g**, **3j**, **3l**, **3m**, and **3o**. Notably, all tested compounds exhibited greater cytotoxicity compared to cisplatin. Compounds **3a**, **3c**, **3i**, and **3m** demonstrated IC_50_ values equivalent to doxorubicin, while **3b**, **3d**, **3h**, and **3k** exhibited improved cytotoxicity compared to doxorubicin. These findings suggest that the steric property, aromatic orientation including the substituted position of nitrogen in heterocyclic aromatic motifs and the ring size play important roles in improving the cytotoxicity^37^.

**Table 1:** Cytotoxicity of 5-*O*-ester derivatives of renieramycin T (**3a**–**3o**) and 7-*O*-ester derivatives of renieramycin S (**4a** and **4b**) against the H292 and H460 non-small-cell lung cancer cell lines along with dermal papilla (DP) cell line.

Entry	Compound	5-*O*-substituent	IC_50_ ± S.D. (nM)		
			H292	H460	DP
1	**1**	5,8-Dicarbonyl	34.43 ± 1.70	35.63 ± 1.82	7.07 ± 1.50
2	**2**	H	72.85 ± 2.88	83.95 ± 3.63	51.00 ± 0.08
3	**3a**	Acetyl	43.43 ± 3.00	35.71 ± 2.11	11.73 ± 0.59
4	**3b**	Propanoyl	33.44 ± 0.87	33.88 ± 1.95	23.57 ± 0.65
5	**3c**	*N*-Boc-L-glycinoyl	38.96 ± 5.63	41.63 ± 4.15	5.35 ± 0.27
6	**3d**	*N*-Boc-L-alaninoyl	39.45 ± 1.18	33.03 ± 1.55	25.75 ± 1.05
7	**3e**	*N*-Boc-L-valinoyl	89.64 ± 5.03	76.08 ± 2.77	50.00 ± 1.49
8	**3f.**	*N*-Boc-L-phenylalaninoyl	54.11 ± 4.78	50.68 ± 3.50	39.78 ± 9.88
9	**3g**	2-Pyridinecarbonyl	51.46 ± 1.67	43.62 ± 1.36	35.19 ± 4.13
10	**3h**	4-Pyridinecarbonyl	35.27 ± 1.09	35.65 ± 1.64	4.83 ± 2.18
11	**3i**	2-Quinolinecarbonyl	36.52 ± 1.82	43.31 ± 4.29	8.77 ± 1.94
12	**3j**	3-Quinolinecarbonyl	77.80 ± 5.79	86.13 ± 6.34	37.63 ± 0.47
13	**3k**	3-Pyridine acryloyl	36.29 ± 2.06	38.75 ± 7.25	8.36 ± 2.31
14	**3l**	3-Indolecarbonyl	69.37 ± 1.61	57.91 ± 0.77	72.12 ± 3.01
15	**3m**	2-Furancarbonyl	42.50 ± 3.84	42.89 ± 5.21	25.29 ± 0.24
16	**3n**	2-Thiophenecarbonyl	136.83 ± 6.30	122.83 ± 4.99	68.58 ± 6.25
17	**3o**	2-Pyrrolecarbonyl	56.88 ± 2.66	58.33 ± 3.56	50.02 ± 7.81
18	**4a**^a^	5,8-Dicarbonyl	99.87 ± 0.91	164.20 ± 4.16	77.42 ± 9.90
19	**4b**^b^	5,8-Dicarbonyl	100.03 ± 1.59	104.03 ± 2.20	67.48 ± 5.46
20	Cisplatin	–	4.23 × 10^3^ ± 0.40 × 10^3^	3.86 × 10^3^ ± 0.46 × 10^3^	10.77 × 10^3^ ± 1.9 × 10^3^
21	Doxorubicin	–	40.78 ± 6.89	43.93 ± 6.26	58.00 ± 24.35

H292 and H460 non-small-cell lung cancer and DP cell lines were tested for 72 h.

^a^7-*O*-(propanoyl) ester derivative of renieramycin S (**4a**). ^b^7-*O*-(3-pyridinecarbonyl) ester derivative of renieramycin S (**4b**).

Interestingly, the renieramycin-type derivatives **4a** and **4b**, which contained linear and aromatic nitrogen heterocyclic ester substituents exhibited significantly decrease in cytotoxicity, with reductions of 3- and fivefold against the H292 and H460 cell lines, respectively. These findings highlight the crucial role of the renieramycin–ecteinascidin hybrid core structure as an essential pharmacophore, ensuring the maintenance of cytotoxic potency and essential interactions with pharmacologically related biomolecular targets^16, 17^.

### Cytotoxic evaluation of 3a–3o, 4a, and 4b against normal cell lines

**Figure 4:**
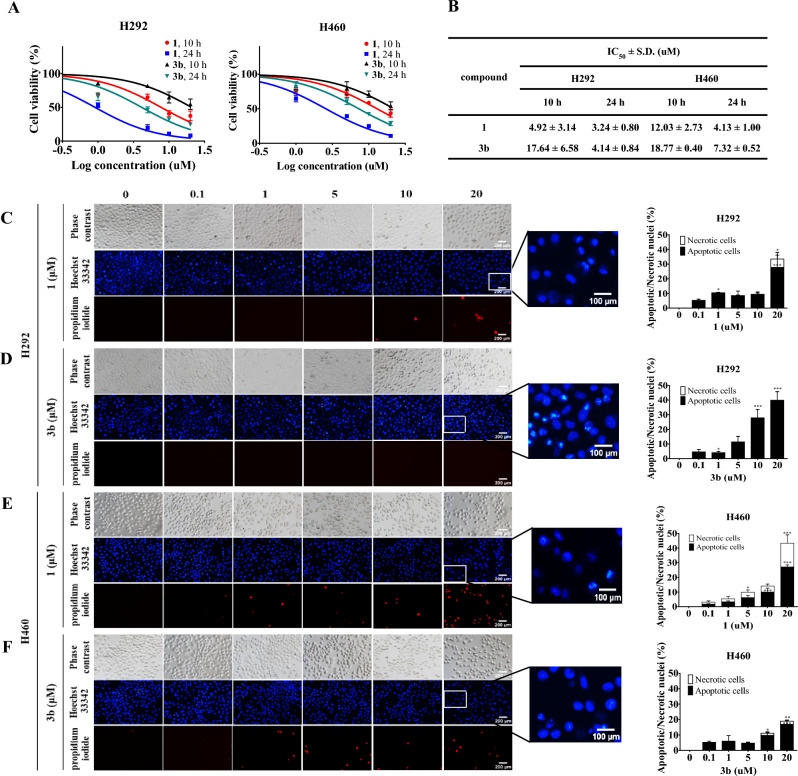
Cell death modes of Renieramycin M (**1**) and 5-*O*-(propanoyl) ester derivative of renieramycin T (**3b**). (**A**) Percentages of cell viability of untreated (control) or treated NSCLC cells with varying dosages of **1** and **3b** (0–20 µM) for 10 and 24 h were represented and investigated by MTT assay. (**B**) IC_50_ values of **1** and **3b** against H292, H460 cell lines were calculated compared to the untreated control. (**C–F**) Morphologies of apoptotic and accidental necrotic cells on H292 and H460 stained with Hoechst 33342 and propidium iodide (PI) were captured using a fluorescence microscope. The percentage of cell death were calculated based on the stained image in H292 and H460 cells. Data were presented as the means of triplicate samples ± SD (n = 3). The cell death of the compound-treated cells was compared to that of the untreated controls; * = *p* < 0.05, ** = *p* < 0.01, and *** = *p* < 0.001.

The cytotoxicity of the 5-*O*-ester derivatives of renieramycin T and their parent compounds was assessed against normal cell lines, including dermal papilla (DP) cell line (Table 1), as well as human keratinocyte (HaCaT) and the non-tumorigenic bronchial epithelial (BEAS-2B) cell lines (see Supporting Information, Table S1). The findings revealed that all renieramycin-type compounds exhibited cytotoxicity within the nanomolar range when tested against the normal cell lines, demonstrating the stronger cytotoxic potency in comparison to H292 and H460 cell lines. Notably, compounds **1** demonstrated robust cytotoxicity against the normal cell lines, with an IC_50_ of 7.07 ± 1.50 nM observed against the DP cell line. Almost all 5-*O*-ester derivatives of renieramycin T possessed significantly reduced cytotoxicity on DP cell in comparison to the mother compounds **1**. Among the series of renieramycin T derivatives, compounds **3c**, **3h**, **3i**, and **3k** exhibited cytotoxicity levels equivalent to the compounds **1**. Furthermore, the results indicated that treating the cytotoxic agents over an extended period (72 h) led to a stronger IC_50_ value compared to shorter durations of treatment (10 and 24 h) (Table S1 and Fig. 4A,B). However, the heightened sensitivity of cytotoxicity against normal cell lines is a commonly observed phenomenon with chemotherapeutic drugs such as cabazitaxel^44^, erlotinib^45^, and elotuzumab^46^. Regarding the in vitro cytotoxic assay against both cancerous and normal cell lines, the 5-*O*-(3-propanoyl) ester of renieramycin T (**3b**) exhibited significant cytotoxicity, indicating its potential utility for in-depth exploration of anti-NSCLC mechanisms.

### Apoptosis and accidental necrosis assays of 1 and 3b against NSCLC cell lines

Apoptosis and accidental necrosis are distinct forms of cell death with different characteristics and underlying mechanisms. Apoptosis is the favored programmed cell death process for eliminating cancer cells. It is a highly regulated and controlled mechanism. In contrast, accidental necrosis is an uncontrolled and unprogrammed form of cell death. Unlike apoptosis, accidental necrosis frequently triggers an inflammatory response that can result in damage to adjacent tissues^47^.

**Figure 5:**
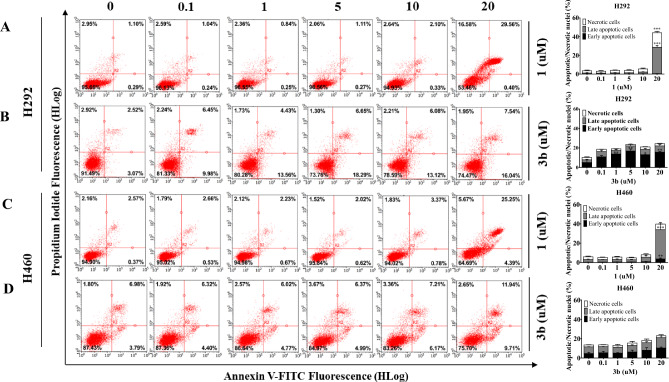
Effects of **1**, and **3b** on apoptotic cell death in NSCLC cells (H460 and H292). (**A–D**) Apoptotic cells were determined after treatment with **1**, and **3b** or untreated (control) based on annexin V-FITC and PI co-staining on H292 and H460 cells. Percentage of cells in each stage on H292 and H460 cells were calculated. Data were presented as the means of triplicate samples ± SD (n = 3). The cell death of the compound-treated cells was compared to that of the untreated controls; * = *p* < 0.05, ** = *p* < 0.01, and *** = *p* < 0.001.

To gain a comprehensive understanding of the anti-NSCLC mechanism exhibited by renieramycin–ecteinascidin hybrid alkaloids, the apoptosis profile of 5-*O*-(3-propanoyl) ester of renieramycin T (**3b**) were further investigated. The co-staining with Hoechst 33342 and propidium iodide (PI) to evaluate the nuclear morphology of apoptotic and necrotic cells in conjugation with cytometric analysis using Annexin V FITC/PI double staining were conducted on both the cisplatin-resistant H292 and cisplatin-sensitive H460 NSCLC cell lines, with a comparative evaluation against the parent compound **1** (Figs. 4, 5).

The anti-proliferative activity of compounds **1** and **3b** against H292 and H460 cells were assessed across a range of concentrations (0–20 µM) under treatment at 10 and 24 h. Notably, both compounds **1** and **3b** demonstrated a dose- and time-dependent anti-NSCLC activity (Fig. 4A,B). The IC_50_ values of compounds** 1** and **3b** on both H292 and H460 cell lines showed significant difference at the short treatment duration (10 h). The cytotoxic effect of **3b** was closely resembled that of the parental compound **1** after 24 h treatment. According to their cytotoxic profiles at short treatment duration, the NSCLC cell lines were treated with various concentrations (0, 0.1, 1, 5, 10, and 20 µM) of compounds** 1** and **3b**, followed by Hoechst 33342 and PI staining to visualize morphological changes by fluorescence microscopy (Fig. 4C–F). The bright blue fluorescence of Hoechst 33342 indicated apoptosis, characterized by DNA condensation and fragmentation. In contrast, the red fluorescence emitted by propidium iodide (PI) indicated necrotic cell death^48^. The percentage of apoptotic and necrotic cells was calculated based on the stained image.

The effects of **3b** compared to** 1** on apoptosis-related morphological changes in NSCLC cells were studied at 7 h. Compounds **1** and **3b** exhibited distinct apoptotic and necrotic cell death mechanisms in both H292 and H460 NSCLC cell lines. For cisplatin-resistant H292 cell lines, the treatment with the highest concentration (20 µM) of mother compound **1** induced both apoptosis and accidental necrosis (Fig. 4C). Conversely, derivative **3b** resulted in morphological apoptosis with no detectable necrotic cells (Fig. 4D). In the case of treatment on cisplatin-sensitive H460 NSCLC cell line, the unwanted necrosis was observed in a dosed-dependent manner based on the concentrations of compound **1** (Fig. 4E). However, compound **3b** induced apoptosis with minimal necrosis (Fig. 4F).

Apoptotic effect of compound **1** and **3b** against both H292 and H460 NSCLC cell lines was further analyzed by flow cytometric analysis (Fig. 5A–D). These results confirmed that the compound **1** at the concentration of 20 µM led to necrotic cell death in both H292 and H460 cell lines in conjugation with late apoptosis. In contrast, derivative **3b** displayed notable evidence of early apoptosis with minimal necrosis in both NSCLC cell lines. This observation corresponds with the Hoechst 33342/PI staining results. The study highlights the potential of 5-*O*-ester derivatives of renieramycin T, particularly compound **3b** as promising leads for the development of improved anticancer drug candidates.

## Experimental section

### General experimental procedures

All the reagents were purchased from Tokyo Chemical Industry (Tokyo, Japan) and used without further purification. The solvents were obtained from Merck (Darmstadt, Germany) and distilled before use. The anhydrous solvents were dried over 4 Å molecular sieves. The reactions were conducted in oven-dried glassware and stirred magnetically under an inert atmosphere using an argon balloon unless otherwise specified.

All reactions were monitored via thin-layer chromatography (TLC) by using aluminum silica gel 60 F254 (Merck) and visualized under ultraviolet light at 254 and 365 nm. Flash column chromatography was also performed by using silica gel (60 Å, 230–400 mesh) as the stationary phase and high-grade solvents, including ethyl acetate and hexane, as the mobile phases. The structures of all the compounds were elucidated by spectroscopic techniques. ^1^H– and ^13^C–NMR spectra were acquired on a Bruker Avance NEO 400 MHz spectrometer. Deuterated chloroform served as the internal standard for both the ^1^H (7.27 ppm) and ^13^C (77.0 ppm) spectra. The optical rotations were measured by a JASCO P-2000 polarimeter using a 1-mL cell with a 1-dm cell path length. ECD spectra were recorded on a JASCO J-815 CD spectrometer. IR spectra were measured with a PerkinElmer Frontier Fourier-transform IR Spectrometer. Accurate mass spectra were recorded on a Bruker Daltonics microTOF mass spectrometer.

The DP, HaCaT, BEAS-2B, H292 and H460 NSCLC cell lines were obtained from the American Type Culture Collection (Manassas, VA, USA). The Roswell Park Memorial Institute (RPMI) 1640 medium, fetal bovine serum (FBS), L-glutamine, penicillin/streptomycin solution, Albumax I, phosphate-buffered saline (PBS), and trypsin–EDTA were procured from Gibco (Gaithersburg, MA, USA). The Dulbecco's Modified Eagle’s Medium (DMEM) were obtained from Gibco (Grand Island, NY, USA). 3-(4,5-dimethylthiazol-2-yl)-2,5-diphenyltetrazoliumbromide (MTT), Hoechst 33342, and propidium iodide (PI) were obtained from Sigma-Aldrich, Co. (St. Louis, MO, USA). Annexin V-FITC/PI apoptosis kit was purchased from ImmunoTools (Gladiolenweg 2, Friesoythe, Germany). Dimethyl sulfoxide (DMSO) was purchased from Merck Millipore (Billerica, MA, USA) or Sigma-Aldrich.

### Isolation and purification of 1

Renieramycin M (**1**) was isolated from Thai blue sponge *Xestospongia* sp. samples collected by scuba diving near Si-Chang Island, in the Gulf of Thailand, at a depth of 3–5 m, with assistance from the Aquatic Recourses Research Institute, Chulalongkorn University and permission from the Department of Fisheries, Ministry of Agriculture and Cooperatives, Thailand (0510.2/8234, 28th October 2019). The fresh blue sponge was mashed and subjected to pretreatment with 10% potassium cyanide in a phosphate buffer solution at pH 7. Next, the mixture was macerated in methanol, filtered, concentrated under reduced pressure, and extracted with hexane and ethyl acetate. The crude extract was then purified through silica gel column chromatography by using hexane and ethyl acetate as the eluents. This process yielded compound **1** as an orange solid, with an isolation yield of 0.02% w/w relative to the dry sponge^19^.

### Semisynthesis of 3a–3o

Naturally derived renieramycin M (**1**) served as the starting material. A solution of compound **1** (25 mg, 0.04 mmol) in dry CH_2_Cl_2_ (40 mL) was stirred vigorously under an 18 W fluorescent lamp at room temperature in an argon atmosphere for 24 h. The reaction mixture was monitored by TLC using a hexane and ethyl acetate solution (1:1 v/v) as the mobile phase. After completion of the transformation, the volatile solvent was removed under reduced pressure, and hexane was added to obtain a yellow precipitate of renieramycin T (**2**), which was dried under a high vacuum. Compound **2** was obtained at an excellent yield (25 mg) and used in the next steps without purification.

Next, mixtures of **2** (25 mg, 0.04 mmol), DMAP (6 mg, 0.05 mmol for **3a**, **3c**, and **3h**; 13 mg, 0.11 mmol for **3b**, **3e**–**3g**, and **3i**–**3o**; 5 mg, 0.04 mmol for **3d**), EDCI (10 mg, 0.05 mmol for **3a**, **3c**, and **3h**; 21 mg, 0.11 mmol for **3b**, **3e**–**3g**, and **3i**–**3o**; 8 mg, 0.04 mmol for **3d**), and the corresponding acylating agents including acids and acid chlorides (0.22 mmol for **3a**–**3c** and **3e**–**3o**; 0.06 mmol for **3d**) in dry CH_2_Cl_2_ (20 mL) were stirred at room temperature under a nitrogen atmosphere for the suitable period (3 h for **3a**, **3d**, **3f**, and **3j**–**3o**; 24 h for **3b**, **3c, 3e**, and **3g**–**3i**). After TLC confirmed completion of the reaction, the mixtures were quenched by the addition of distilled H_2_O (20 mL), followed by extraction with CH_2_Cl_2_ (20 mL, 3 times). The organic layers were combined and dried over anhydrous MgSO_4_, filtered, and concentrated under reduced pressure. The crude products were purified by silica gel flash chromatography using a hexane and ethyl acetate solution as the eluent to yield **3a**–**3o**. The chemical structures of the semisynthetic derivatives of** 2** were characterized by optical rotation, IR spectroscopy, high-resolution electrospray ionization mass spectroscopy, one- and two-dimensional NMR spectroscopy, and ECD spectroscopy. The spectra of **2** and **3d** were matched with the previously reported data^20, 35^.

### Semisynthesis of 4a and 4b

A solution of compound **1** (25 mg, 0.04 mmol) in dry CH_2_Cl_2_ (40 mL) was stirred vigorously under an 18 W fluorescent lamp at room temperature in an argon atmosphere for 24 h. Next, DMAP (13 mg, 0.11 mmol), EDCI (21 mg, 0.11 mmol), and the corresponding acylating agents (0.22 mmol) were added, followed by continuous stirring at ambient temperature under light and inert gas for 24 h. The progress of the reaction was monitored by TLC. After completion of the reaction, the mixture was quenched, extracted, and purified to obtain the pure product. The structural characterization of the product was carried out as described above.

### Cytotoxicity evaluation against NSCLC cell lines

The in vitro cytotoxicity of compounds **3a**–**3o**, **4a**, and **4b** was determined against the H292 and H460 NSCLC cell lines by the MTT colorimetric assay. Each compound was dissolved in DMSO to prepare a 10 mM stock solution. Both the H292 and H460 cells were cultured in RPMI 1640 medium supplemented with 2 mM L-glutamine, 10% FBS, and 100 units/mL of penicillin–streptomycin at 37 °C under 5% CO_2_. The NSCLC cells were trypsinized and seeded with a density of 5 × 10^3^ cells/well in a 96-well plate, followed by overnight incubation. Serial dilutions of the test compounds ranging from 1 to 250 nM were prepared in the presence of DMSO (< 0.2% v/v). Then, the cells were treated with various concentrations of each derivative for 72 h and successively incubated with a 0.5 mg/mL MTT solution for 2 h. After incubation, formazan crystals were solubilized by adding 100 µL of DMSO, and their absorbance was measured at 570 nm by a spectrophotometric microtiter plate reader (PerkinElmer Victor 3 1420 Multilabel Plate Counter). Cell viability was calculated as the percentage of nontreated control cells. The mean IC_50_ values were obtained from three independent experiments. Each experiment was conducted in triplicate with at least five concentrations of the tested compounds. The GraphPad Prism software (version 5) was used to calculate the average IC_50_ and standard deviation (S.D.) values for each experiment. Cisplatin and doxorubicin were used as the positive controls, while 0.2% DMSO served as the negative control.

### Cell viability on normal cell lines

DP and BEAS-2B were seeded into 96-well plates at densities of 1 × 10^4^ cells per well, while HaCaT cells were seeded at densities of 5 × 10^3^ cells per well. The cells were allowed to adhere for 24 h. Subsequently, the cells were treated with various concentrations of compounds **3a**–**3o**, **4a**, and **4b** at 72 h for DP cells and at 24 h for HaCaT and BEAS-2B cells. After the treatment period, cells were incubated with 0.5 mg/ml of MTT for 2 h. The resulting MTT product was measured at 570 nm using a spectrophotometric microplate reader. Cell viability was calculated by comparing the optical density (OD) measurements to those of the untreated control, and the results were expressed as a percentage.

### Apoptosis and accidental necrosis assays

NSCLC cells (H292 and H460) were seeded into a 96-well plate at a density of 1 × 10^4^ cells/well for 24 h. Subsequently, the cells were treated with several concentrations of compounds **1** and **3b** (0–20 µM) for 7 h. After the treatment, the cells were stained with 10 µg/mL of Hoechst 33342 and/or 5 µg/mL of propidium iodide (PI) for 15 min at 37 °C. Visualization and imaging of the stained cells were carried out using a fluorescence microscope (Nikon ECLIPSE Ts2, Tokyo, Japan). Hoechst 33342 selectively stained the nuclei of all cells, with apoptotic cells exhibiting intensely condensed chromatin and/or fragmented nuclei. PI specifically stained the DNA of cells with damaged cell membranes, indicating necrotic cells. The percentage of cell death was analyzed and reported.

### Annexin V-FITC/PI double staining apoptotic assay

Apoptosis cell death was investigated following the manufacturer's protocol (ImmunoTools, Friesoythe, Germany) using FITC–labeled Annexin V/PI. Treated cells was cultured overnight in 24-well plates at a density of 5 × 10^4^ cells/well. Subsequently, cells were treated with various concentrations of **1**, and **3b** (0, 0.1, 1, 5, 10, and 20 µM) for 7 h. After treatment, cells were collected, washed with cold PBS, and then suspended in binding buffer. Each cell suspension was stained with 2.5 µL of annexin V-FITC and 1 µL of PI. The mixture was vortexed, then incubated for 15 min at room temperature. Guava easyCyte flow cytometer (EMD Millipore, Hayward, CA, USA) was utilized to detect live, apoptotic, and necrotic cells. The modes of cell death were classified as necrotic (PI+), and apoptotic cell death, divided into early apoptotic (PI (-) Annexin-V (+)) and late apoptotic (PI (+) Annexin-V (+)).

### Statistical analysis

The data were presented as mean ± standard deviation (S.D.). Statistical analyses performed using a One-Way ANOVA.

## Conclusion

In conclusion, a novel series of 5-*O*-ester of renieramycin T (**3a–3o**) was successfully prepared through a two-step chemical transformation of compound **1**, involving a mild and regioselective photoredox reaction to obtain **2**, followed by Steglich esterification. Interestingly, the one-pot transformation including photoredox reaction and esterification of **1** gave 7-*O*-ester derivatives of renieramycin (**4a** and **4b**). The structure–cytotoxicity relationship study of renieramycin–ecteinascidin hybrid alkaloids as potential cytotoxic agents for the treatment of NSCLC was investigated. The cytotoxicity of 5-*O*-ester derivatives was evaluated against the metastatic H292 and H460 human NSCLC cell lines. The results indicated that the derivatives **3a**, **3b**, **3d**, **3h**, **3i**, and **3k**, possessed significant cytotoxicity, with the same IC_50_ as compound **1** and twofold stronger than compound** 2**. However, the renieramycin S derivatives showed diminished cytotoxicity compared with the mother alkaloids** 1** and **2**. The 5-*O*-ester derivatives of renieramycin T having linear alkyl (**3a** and **3b**), *N*-Boc-L-amino (**3c**–**3f**), and heterocyclic aromatic (**3g**–**3o**) substituents exhibited nanomolar-range cytotoxicity against both the H292 and H460 cell lines. This variation in the anticancer potency was attributed to the chemical skeleton of the 5-*O*-ester substituents, where steric factors and aromatic orientation plays a crucial role in controlling cytotoxicity. Among the prepared derivatives, 5-*O*-(3-propanoyl) ester of renieramycin T (**3b**) exhibited prominent cytotoxicity. Furthermore, the cytotoxic evaluation of 5-*O*-ester derivatives of renieramycin T against normal cell lines, including DP, HaCaT, and BEAS-2B cell lines was demonstrated in comparison to their cytotoxic activity against H292 and H460 cell lines. Apoptotic assay involving staining and cell morphological analysis along with flow cytometric analysis, provided supplementary evidence regarding the anticancer potential of compound **3b**, which demonstrated apoptosis as the mechanism of the cytotoxicity with minimal accidental necrosis, in contrast to the parent compound **1**. These findings emphasized the 5-*O*-ester derivative of renieramycin T, a semisynthesized series of renieramycin–ecteinascidin hybrid derivatives as the promising leads for anti-lung cancer agents.

### Supplementary Information

Supplementary Information.

## Data Availability

The datasets used and/or analyzed during the current study available from the corresponding author on reasonable request. Samples of compounds **1**, **2**, **3a**–**3o**, **4a**, and **4b** are available from S.C.
