# Supplementary material for: Semisynthesis of 5-O-ester derivatives of renieramycin T and their cytotoxicity against non-small-cell lung cancer cell lines
Source: Sci Rep. 2023 Dec 6;13:21485. doi: 10.1038/s41598-023-48526-2 (PMC10700347; doi:10.1038/s41598-023-48526-2)
Supplement: Supplementary file 1 — Supplementary Information. [file 41598_2023_48526_MOESM1_ESM.docx]

Semisynthesis of 5-*O*-Ester Derivatives of Renieramycin T and Their Cytotoxicity against Non-Small-Cell Lung Cancer Cell Lines

Koonchira Buaban^1,2^, Bhurichaya Innets^3,4^, Korrakod Petsri^3,4^, Suwimon Sinsook^1,5^, Pithi Chanvorachote^3,4^, Chaisak Chansriniyom^1,2^, Khanit Suwanborirux^1,2^, Masashi Yokoya^6^, Naoki Saito^6^, and Supakarn Chamni^1,2^*

^1^ Department of Pharmacognosy and Pharmaceutical Botany, Faculty of Pharmaceutical Sciences, Chulalongkorn University, Bangkok 10330, Thailand

^2^ Natural Products and Nanoparticles Research Unit (NP2), Chulalongkorn University, Bangkok 10330, Thailand

^3^ Center of Excellence in Cancer Cell and Molecular Biology, Faculty of Pharmaceutical Sciences, Chulalongkorn University, Bangkok, 10330, Thailand

^4^ Department of Pharmacology and Physiology, Faculty of Pharmaceutical Sciences, Chulalongkorn University, Bangkok, 10330, Thailand

^5^ Pharmaceutical Sciences and Technology Program, Faculty of Pharmaceutical Sciences, Chulalongkorn University, Bangkok 10330, Thailand

^6^ Graduate School of Pharmaceutical Sciences, Meiji Pharmaceutical University, 2-522-1 Noshio, Kiyose, Tokyo 204-8588, Japan

* Corresponding author: supakarn.c@pharm.chula.ac.th; Tel.: +662-218-8357

**Table of Contents**

[Physical and spectroscopic data of **3a** 8](#_Toc140175553)

[Physical and spectroscopic data of **3b** 13](#_Toc140175554)

[Physical and spectroscopic data of **3c** 17](#_Toc140175555)

[Physical and spectroscopic data of **3d** 21](#_Toc140175556)

[Physical and spectroscopic data of **3e** 25](#_Toc140175557)

[Physical and spectroscopic data of **3f** 30](#_Toc140175558)

[Physical and spectroscopic data of **3g** 34](#_Toc140175559)

[Physical and spectroscopic data of **3h** 38](#_Toc140175560)

[Physical and spectroscopic data of **3i** 42](#_Toc140175561)

[Physical and spectroscopic data of **3j** 47](#_Toc140175562)

[Physical and spectroscopic data of **3k** 51](#_Toc140175563)

[Physical and spectroscopic data of **3l** 56](#_Toc140175564)

[Physical and spectroscopic data of **3m** 60](#_Toc140175565)

[Physical and spectroscopic data of **3n** 64](#_Toc140175566)

[Physical and spectroscopic data of **3o** 68](#_Toc140175567)

[Physical and spectroscopic data of **4a** 72](#_Toc140175568)

[Physical and spectroscopic data of **4b** 76](#_Toc140175569)

Cytotoxicity of 5-*O*-ester derivatives of renieramycin T (**3a**−**3o**) and…………………………..81

7-O-ester derivatives of renieramycin S (**4a** and **4b**) against other non-lung cell lines: human keratinocyte (HaCaT) and non-tumorigenic bronchial epithelial (BEAS-2B) cell lines.

**List of Figures**

[**Figure S1.** ^1^H NMR (400 MHz) spectrum of **3a** in CDCl_3_ 9](#_Toc139933961)

[**Figure S2.** ^13^C NMR (100 MHz) spectrum of **3a** in CDCl_3_ 9](#_Toc139933962)

[**Figure S3.** COSY (400 MHz) spectrum of **3a** in CDCl_3_ 10](#_Toc139933963)

[**Figure S4.** HSQC (400 MHz) spectrum of **3a** in CDCl_3_ 10](#_Toc139933964)

[**Figure S5.** HMBC (400 MHz) spectrum of **3a** in CDCl_3_ 11](#_Toc139933965)

[**Figure S6.** ^1^H NMR (400 MHz) spectrum of **3a** in (CD_3_)_2_CO 12](#_Toc139933966)

[**Figure S7.** ^1^H NMR (400 MHz) spectrum of **3b** in CDCl_3_ 14](#_Toc139933967)

[**Figure S8.** ^13^C NMR (100 MHz) spectrum of **3b** in CDCl_3_ 14](#_Toc139933968)

[**Figure S9.** COSY (400 MHz) spectrum of **3b** in CDCl_3_ 15](#_Toc139933969)

[**Figure S10.** HSQC (400 MHz) spectrum of **3b** in CDCl_3_ 15](#_Toc139933970)

[**Figure S11.** HMBC (400 MHz) spectrum of **3b** in CDCl_3_ 16](#_Toc139933971)

[**Figure S12.** ^1^H NMR (400 MHz) spectrum of **3c** in CDCl_3_ 18](#_Toc139933972)

[**Figure S13.** ^13^C NMR (100 MHz) spectrum of **3c** in CDCl_3_ 18](#_Toc139933973)

[**Figure S14.** COSY (400 MHz) spectrum of **3c** in CDCl_3_ 19](#_Toc139933974)

[**Figure S15.** HSQC (400 MHz) spectrum of **3c** in CDCl_3_ 19](#_Toc139933975)

[**Figure S16.** HMBC (400 MHz) spectrum of **3c** in CDCl_3_ 20](#_Toc139933976)

[**Figure S17.** ^1^H NMR (400 MHz) spectrum of **3d** in CDCl_3_ 22](#_Toc139933977)

[**Figure S18.** ^13^C NMR (100 MHz) spectrum of **3d** in CDCl_3_ 22](#_Toc139933978)

[**Figure S19.** COSY (400 MHz) spectrum of **3d** in CDCl_3_ 23](#_Toc139933979)

[**Figure S20.** HSQC (400 MHz) spectrum of **3d** in CDCl_3_ 23](#_Toc139933980)

[**Figure S21.** HMBC (400 MHz) spectrum of **3d** in CDCl_3_ 24](#_Toc139933981)

[**Figure S22.** ^1^H NMR (400 MHz) spectrum of **3e** in CDCl_3_ 26](#_Toc139933982)

[**Figure S23.** ^13^C NMR (100 MHz) spectrum of **3e** in CDCl_3_ 26](#_Toc139933983)

[**Figure S24.** COSY (400 MHz) spectrum of **3e** in CDCl_3_ 27](#_Toc139933984)

[**Figure S25.** HSQC (400 MHz) spectrum of **3e** in CDCl_3_ 27](#_Toc139933985)

[**Figure S26.** HMBC (400 MHz) spectrum of **3e** in CDCl_3_ 28](#_Toc139933986)

[**Figure S27.** ^1^H NMR (400 MHz) spectrum of **3e** in (CD_3_)_2_CO 29](#_Toc139933987)

[**Figure S28.** ^1^H NMR (400 MHz) spectrum of **3f** in CDCl_3_ 31](#_Toc139933988)

[**Figure S29.** ^13^C NMR (100 MHz) spectrum of **3f** in CDCl_3_ 31](#_Toc139933989)

[**Figure S30.** COSY (400 MHz) spectrum of **3f** in CDCl_3_ 32](#_Toc139933990)

[**Figure S31.** HSQC (400 MHz) spectrum of **3f** in CDCl_3_ 32](#_Toc139933991)

[**Figure S32.** HMBC (400 MHz) spectrum of **3f** in CDCl_3_ 33](#_Toc139933992)

[**Figure S33.** ^1^H NMR (400 MHz) spectrum of **3g** in CDCl_3_ 35](#_Toc139933993)

[**Figure S34.** ^13^C NMR (100 MHz) spectrum of **3g** in CDCl_3_ 35](#_Toc139933994)

[**Figure S35.** COSY (400 MHz) spectrum of **3g** in CDCl_3_ 36](#_Toc139933995)

[**Figure S36.** HSQC (400 MHz) spectrum of **3g** in CDCl_3_ 36](#_Toc139933996)

[**Figure S37.** HMBC (400 MHz) spectrum of **3g** in CDCl_3_ 37](#_Toc139933997)

[**Figure S38.** ^1^H NMR (400 MHz) spectrum of **3h** in CDCl_3_ 39](#_Toc139933998)

[**Figure S39.** ^13^C NMR (100 MHz) spectrum of **3h** in CDCl_3_ 39](#_Toc139933999)

[**Figure S40.** COSY (400 MHz) spectrum of **3h** in CDCl_3_ 40](#_Toc139934000)

[**Figure S41.** HSQC (400 MHz) spectrum of **3h** in CDCl_3_ 40](#_Toc139934001)

[**Figure S42.** HMBC (400 MHz) spectrum of **3h** in CDCl_3_ 41](#_Toc139934002)

[**Figure S43.** ^1^H NMR (400 MHz) spectrum of **3i** in CDCl_3_ 43](#_Toc139934003)

[**Figure S44.** ^13^C NMR (100 MHz) spectrum of **3i** in CDCl_3_ 43](#_Toc139934004)

[**Figure S45.** COSY (400 MHz) spectrum of **3i** in CDCl_3_ 44](#_Toc139934005)

[**Figure S46.** HSQC (400 MHz) spectrum of **3i** in CDCl_3_ 44](#_Toc139934006)

[**Figure S47.** HMBC (400 MHz) spectrum of **3i** in CDCl_3_ 45](#_Toc139934007)

[**Figure S48.** ^1^H NMR (400 MHz) spectrum of **3i** in (CD_3_)_2_CO 46](#_Toc139934008)

[**Figure S49.** ^1^H NMR (400 MHz) spectrum of **3j** in CDCl_3_ 48](#_Toc139934009)

[**Figure S50.** ^13^C NMR (100 MHz) spectrum of **3j** in CDCl_3_ 48](#_Toc139934010)

[**Figure S51.** COSY (400 MHz) spectrum of **3j** in CDCl_3_ 49](#_Toc139934011)

[**Figure S52.** HSQC (400 MHz) spectrum of **3j** in CDCl_3_ 49](#_Toc139934012)

[**Figure S53.** HMBC (400 MHz) spectrum of **3j** in CDCl_3_ 50](#_Toc139934013)

[**Figure S54.** ^1^H NMR (400 MHz) spectrum of **3k** in CDCl_3_ 52](#_Toc139934014)

[**Figure S55.** ^13^C NMR (100 MHz) spectrum of **3k** in CDCl_3_ 52](#_Toc139934015)

[**Figure S56.** COSY (400 MHz) spectrum of **3k** in CDCl_3_ 53](#_Toc139934016)

[**Figure S57.** HSQC (400 MHz) spectrum of **3k** in CDCl_3_ 53](#_Toc139934017)

[**Figure S58.** HMBC (400 MHz) spectrum of **3k** in CDCl_3_ 54](#_Toc139934018)

[**Figure S59.** ^1^H NMR (400 MHz) spectrum of **3k** in (CD_3_)_2_CO 55](#_Toc139934019)

[**Figure S60.** ^1^H NMR (400 MHz) spectrum of **3l** in CDCl_3_ 57](#_Toc139934020)

[**Figure S61.** ^13^C NMR (100 MHz) spectrum of **3l** in CDCl_3_ 57](#_Toc139934021)

[**Figure S62.** COSY (400 MHz) spectrum of **3l** in CDCl_3_ 58](#_Toc139934022)

[**Figure S63.** HSQC (400 MHz) spectrum of **3l** in CDCl_3_ 58](#_Toc139934023)

[**Figure S64.** HMBC (400 MHz) spectrum of **3l** in CDCl_3_ 59](#_Toc139934024)

[**Figure S65.** ^1^H NMR (400 MHz) spectrum of **3m** in CDCl_3_ 61](#_Toc139934025)

[**Figure S66.** ^13^C NMR (100 MHz) spectrum of **3m** in CDCl_3_ 61](#_Toc139934026)

[**Figure S67.** COSY (400 MHz) spectrum of **3m** in CDCl_3_ 62](#_Toc139934027)

[**Figure S68.** HSQC (400 MHz) spectrum of **3m** in CDCl_3_ 62](#_Toc139934028)

[**Figure S69.** HMBC (400 MHz) spectrum of **3m** in CDCl_3_ 63](#_Toc139934029)

[**Figure S70.** ^1^H NMR (400 MHz) spectrum of **3n** in CDCl_3_ 65](#_Toc139934030)

[**Figure S71.** ^13^C NMR (100 MHz) spectrum of **3n** in CDCl_3_ 65](#_Toc139934031)

[**Figure S72.** COSY (400 MHz) spectrum of **3n** in CDCl_3_ 66](#_Toc139934032)

[**Figure S73.** HSQC (400 MHz) spectrum of **3n** in CDCl_3_ 66](#_Toc139934033)

[**Figure S74.** HMBC (400 MHz) spectrum of **3n** in CDCl_3_ 67](#_Toc139934034)

[**Figure S75.** ^1^H NMR (400 MHz) spectrum of **3o** in CDCl_3_ 69](#_Toc139934035)

[**Figure S76.** ^13^C NMR (100 MHz) spectrum of **3o** in CDCl_3_ 69](#_Toc139934036)

[**Figure S77.** COSY (400 MHz) spectrum of **3o** in CDCl_3_ 70](#_Toc139934037)

[**Figure S78.** HSQC (400 MHz) spectrum of **3o** in CDCl_3_ 70](#_Toc139934038)

[**Figure S79.** HMBC (400 MHz) spectrum of **3o** in CDCl_3_ 71](#_Toc139934039)

[**Figure S80.** ^1^H NMR (400 MHz) spectrum of **4a** in CDCl_3_ 73](#_Toc139934040)

[**Figure S81.** ^13^C NMR (100 MHz) spectrum of **4a** in CDCl_3_ 73](#_Toc139934041)

[**Figure S82.** COSY (400 MHz) spectrum of **4a** in CDCl_3_ 74](#_Toc139934042)

[**Figure S83.** HSQC (400 MHz) spectrum of **4a** in CDCl_3_ 74](#_Toc139934043)

[**Figure S84.** HMBC (400 MHz) spectrum of **4a** in CDCl_3_ 75](#_Toc139934044)

[**Figure S85.** ^1^H NMR (400 MHz) spectrum of **4b** in CDCl_3_ 77](#_Toc139934045)

[**Figure S86.** ^13^C NMR (100 MHz) spectrum of **4b** in CDCl_3_ 77](#_Toc139934046)

[**Figure S87.** COSY (400 MHz) spectrum of **4b** in CDCl_3_ 78](#_Toc139934047)

[**Figure S88.** HSQC (400 MHz) spectrum of **4b** in CDCl_3_ 78](#_Toc139934048)

[**Figure S89.** HMBC (400 MHz) spectrum of **4b** in CDCl_3_ 79](#_Toc139934049)

**List of Table**

[**Table S1.** Cytotoxicity of 5-*O*-ester derivatives of renieramycin T (**3a*−*3o**) and 7-*O*-ester derivatives of renieramycin S (**4a** and **4b**) against other non-lung cell lines: human keratinocyte (HaCaT) and non-tumorigenic bronchial epithelial (BEAS-2B) cell lines. 81](#_Toc140175278)

# Physical and spectroscopic data of **3a**

*5-O-(acetyl)* *ester derivative of renieramycin T,* **3a**: The title compound was synthesized from **2** (20.0 mg, 0.03 mmol), DMAP (5.1 mg, 0.04 mmol), EDCI (7.8 mg, 0.04 mmol) and acetic anhydride (0.02 mL, 0.17 mmol) to afford **3a**; yield 65% (brsm); yellow amorphous powder; $[]_{D}^{25}$ +10.5 (*c* 0.72, CHCl_3_) (lit. $[]_{D}^{26}$ +15.3 (*c* 0.31, CHCl_3_)^20^; ECD Δ*ε* (*c* 45.33 μM, methanol, 20 ^o^C) −1.6 (300), −2.4 (296), +10.2 (262), −7.0 (223), +6.4 (214), +11.6 (210), +0.9 (206), −13.8 (202), +11.9 (197), −34.0 (195), −21.3 (191) nm; IR (ATR) ν_max_ 2951, 2840, 1651, 1450, 1019, 732 cm^−1^; ^1^H NMR (CDCl_3_, 400 MHz) δ 5.97 (1H, overlapped, 26-H), 5.96 (2H, dd, *J* = 24.8, 1.2 Hz, OCH_2_O), 4.58 (1H, dd, *J* = 11.6, 2.4 Hz, 22-H_α_), 4.16 (1H, t, *J* = 4.4 Hz, 1-H), 4.12 (1H, d, *J* = 2.2 Hz, 21-H), 4.02 (1H, dd, *J* = 11.6, 4.4 Hz, 22-H_β_), 3.98 (1H, overlapped, 11-H), 3.96 (3H, s, 17-OCH_3_), 3.37 (1H, d, *J* = 7.2 Hz, 13-H), 3.23 (1H, dt, *J* = 12.1, 2.9 Hz, 3-H), 2.74 (1H, dd, *J* = 20.8, 7.2 Hz, 14-H_α_), 2.52 (1H, br d, *J* = 13.2 Hz, 4-H_α_), 2.33 (1H, d, *J* = 7.2 Hz, 14-H_β_), 2.30 (3H, s, 2'-CH_3_), 2.27 (3H, s, NCH_3_), 2.02 (3H, s, 6-CH_3_), 1.92 (3H, s, 16-CH_3_), 1.85 (3H, dq, *J* = 7.2, 1.2 Hz, 27-CH_3_), 1.66 (3H, s, 28-CH_3_), 1.61 (1H, overlapped, 4-H_β_); ^13^C NMR (CDCl_3_, 100 MHz) δ 186.0 (C-15), 182.7 (C-18), 168.7 (C-1'), 167.0 (C-24), 155.5 (C-17), 144.9 (C-7), 141.7 (C-20), 140.8 (C-8), 140.3 (C-26), 139.9 (C-5), 135.5 (C-19), 128.9 (C-16), 126.7 (C-25), 120.1 (C-6), 117.3 (21-CN), 112.3 (C-10), 111.9 (C-9), 101.7 (OCH_2_O), 63.1 (C-22), 60.9 (17-OCH_3_), 58.9 (C-21), 55.4 (C-1), 54.8 (C-3), 54.7 (C-11), 54.6 (C-13), 41.4 (NCH_3_), 27.9 (C-4), 21.0 (C-14), 20.4 (28-CH_3_), 20.2 (2'-CH_3_), 15.9 (27-CH_3_), 9.4 (6-CH_3_), 8.6 (16-CH_3_); HRESIMS *m/z* 618.2447 ([M+H]^+^, calculated for C_33_H_36_N_3_O_9_, 618.2446).


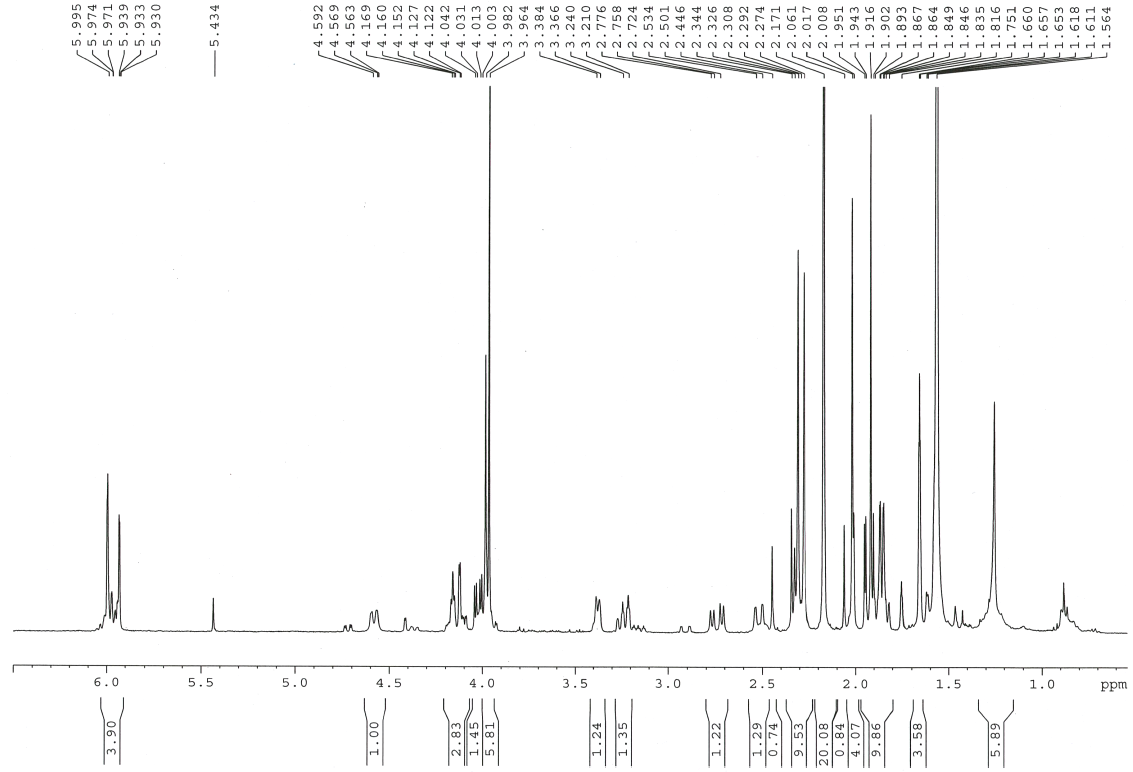


g, h

g, h

g: grease, h: hexane

H_2_O

acetone

dichloromethane

**Figure S1.** ^1^H NMR (400 MHz) spectrum of **3a** in CDCl_3_


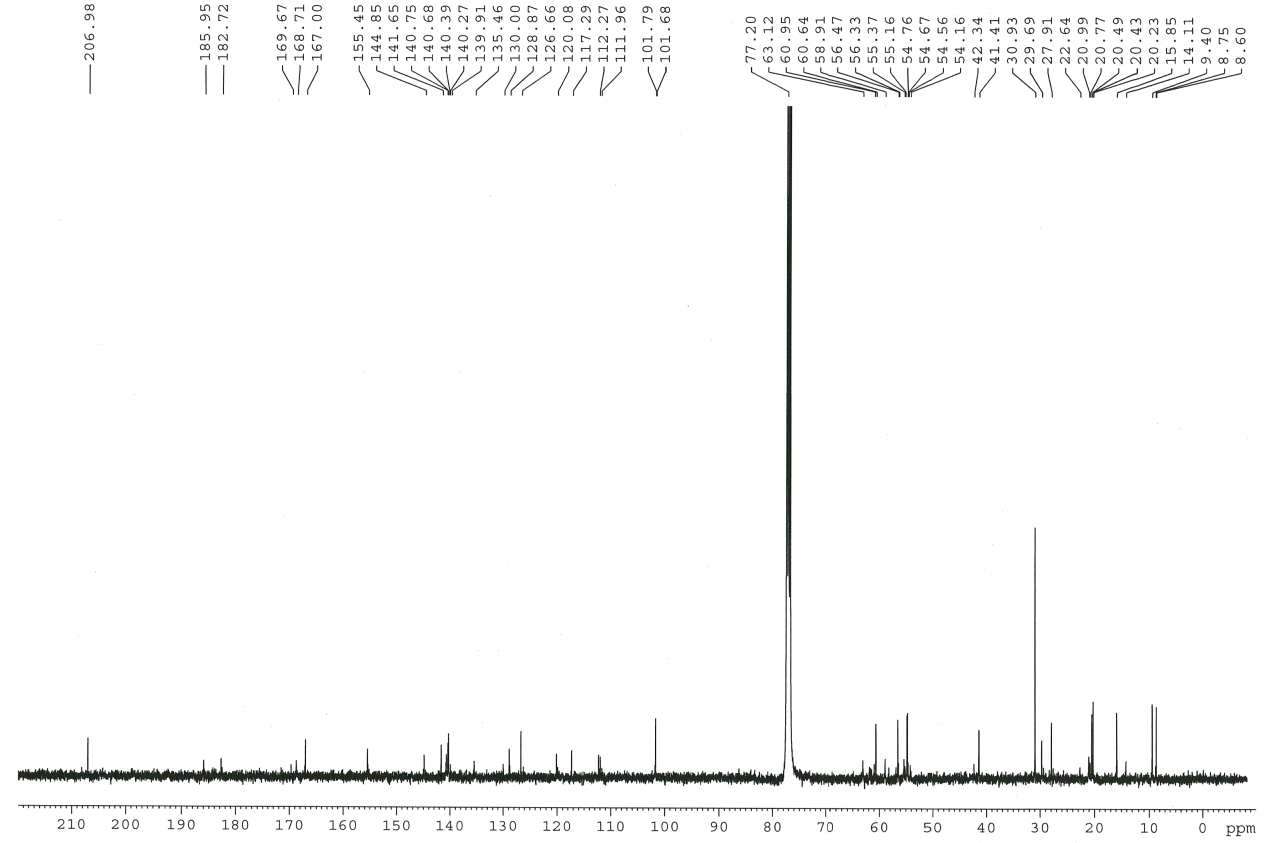


g

h h

acetone

acetone

g: grease, h: hexane

**Figure S2.** ^13^C NMR (100 MHz) spectrum of **3a** in CDCl_3_

**
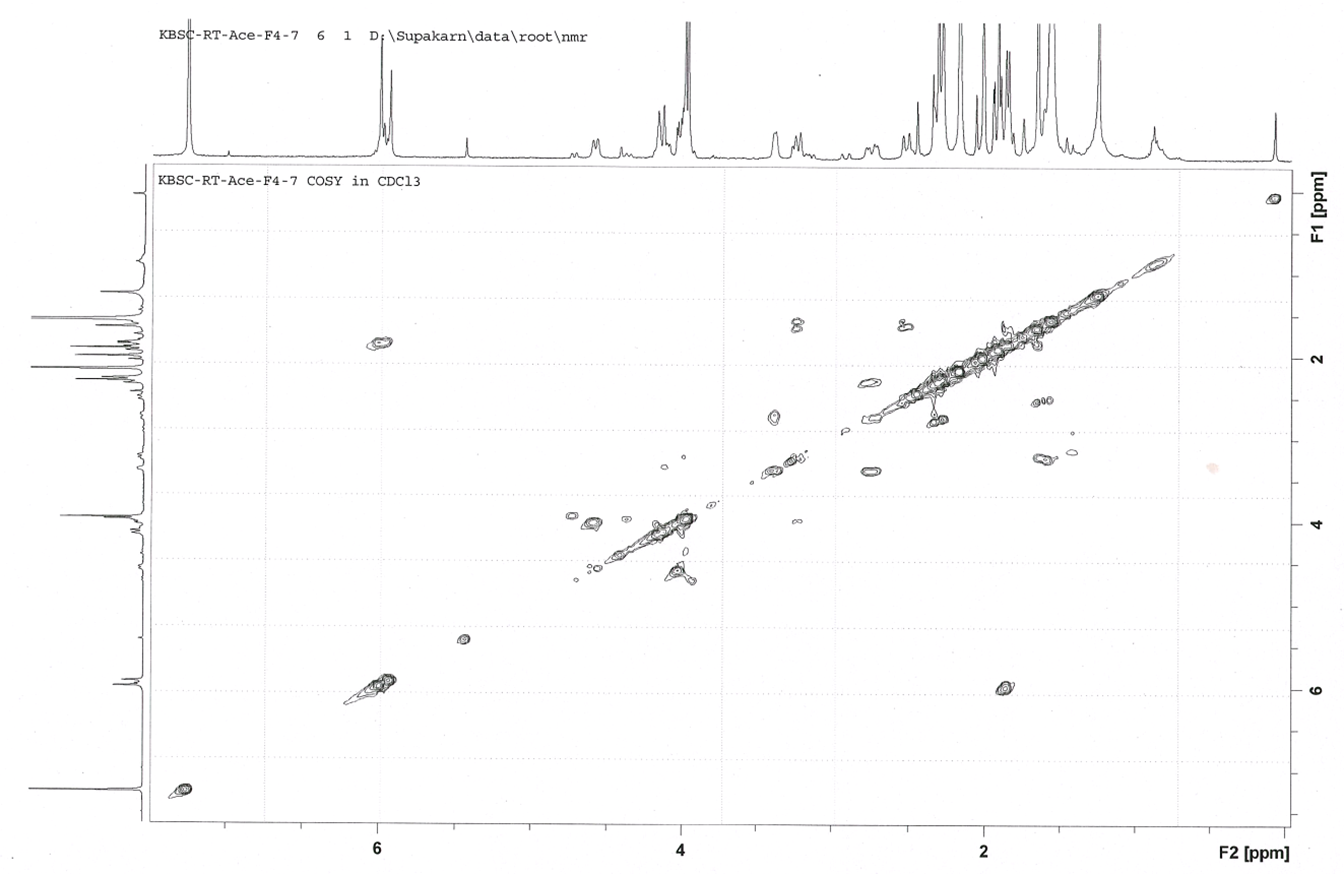
**

**Figure S3.** COSY (400 MHz) spectrum of **3a** in CDCl_3_


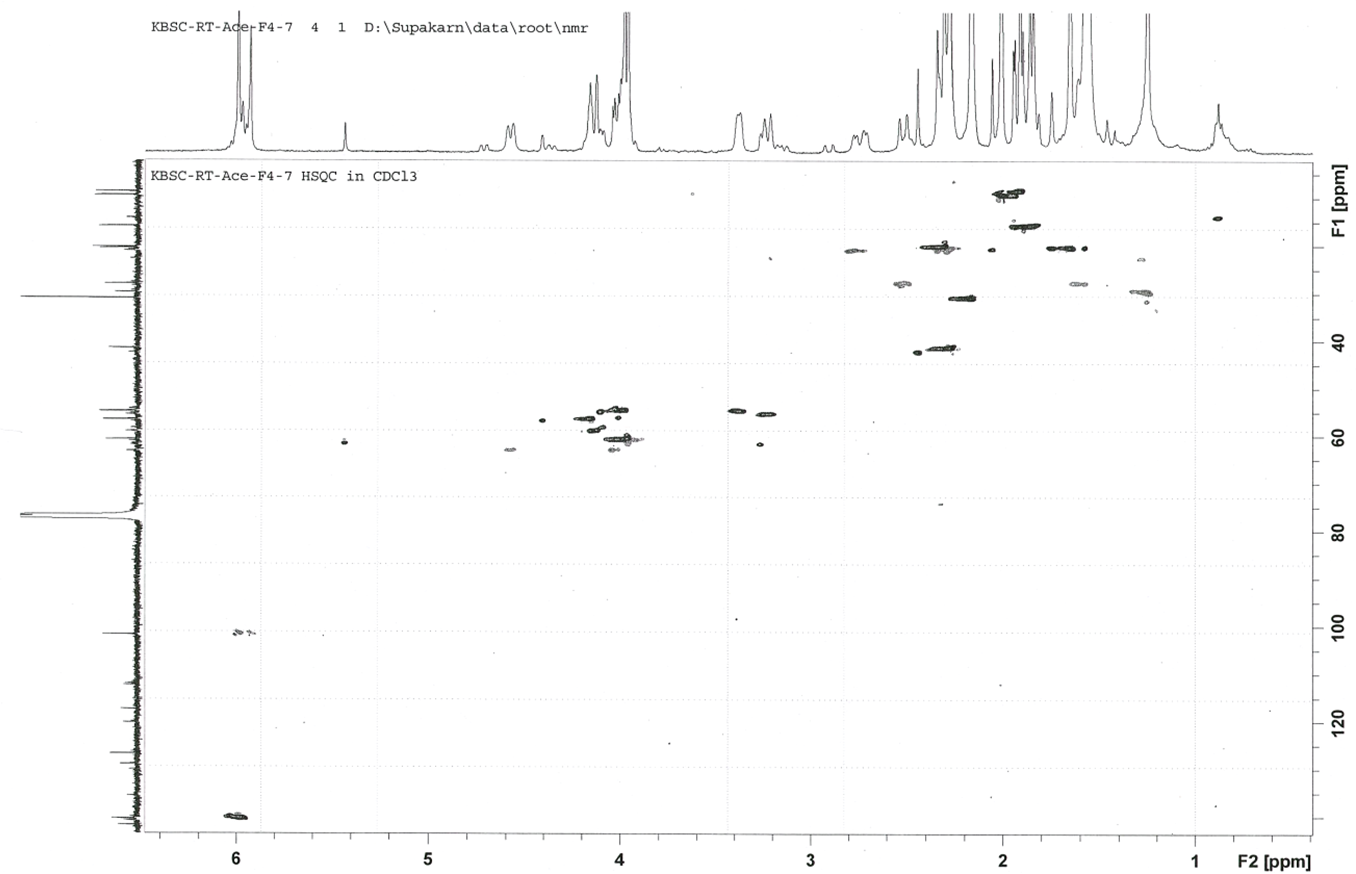


**Figure S4.** HSQC (400 MHz) spectrum of **3a** in CDCl_3_


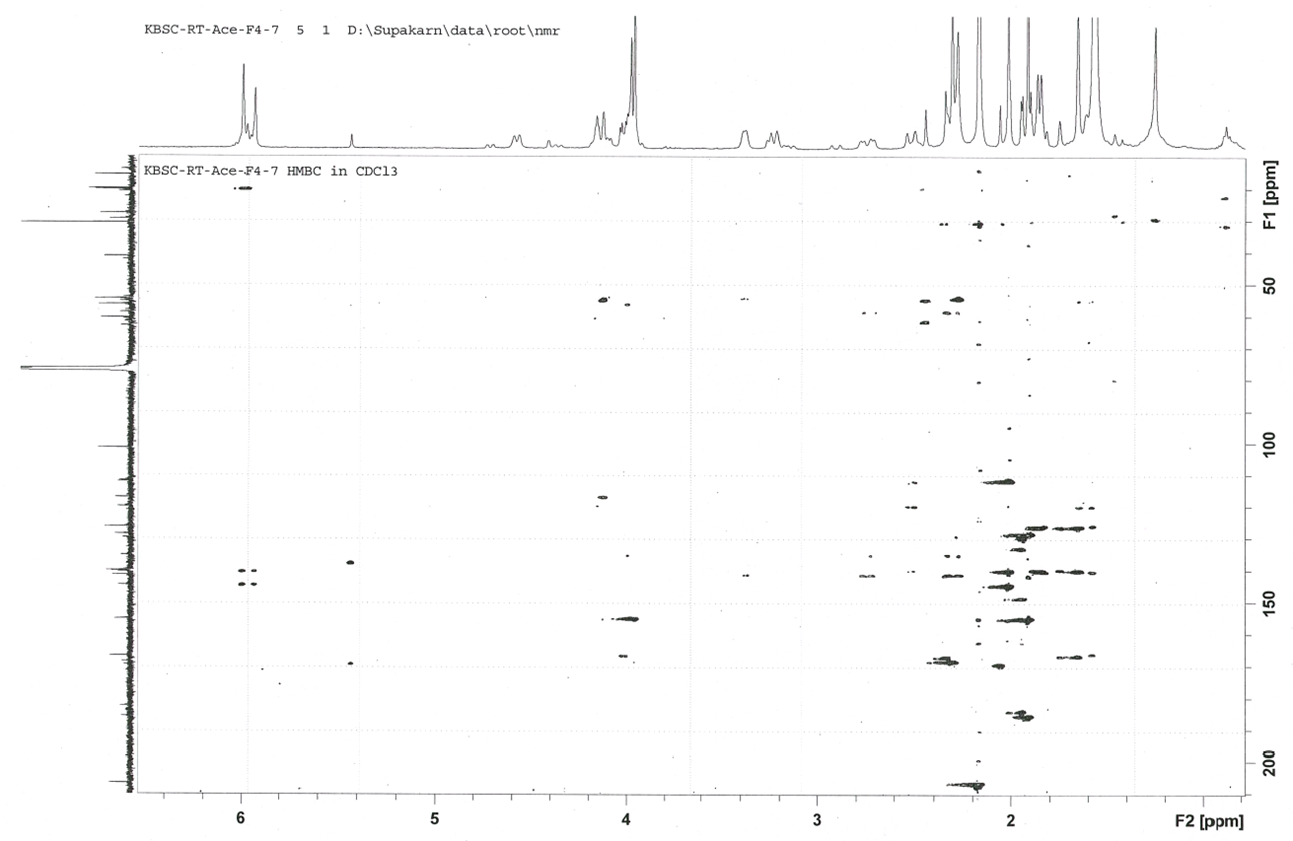


**Figure S5.** HMBC (400 MHz) spectrum of **3a** in CDCl_3_


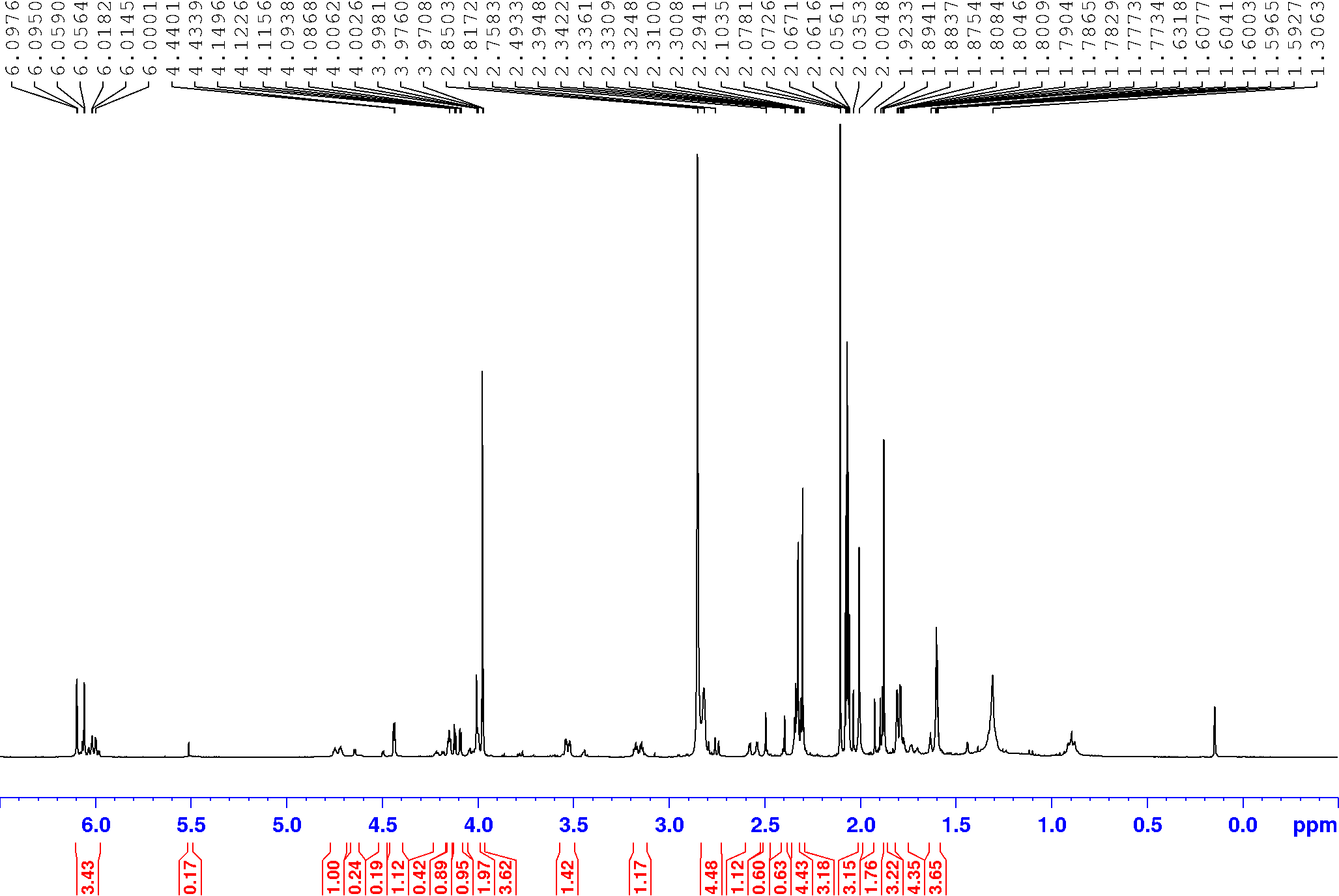


g

g

g: grease

**Figure S6.** ^1^H NMR (400 MHz) spectrum of **3a** in (CD_3_)_2_CO

^1^H NMR ((CD_3_)_2_CO, 400 MHz) δ 6.08 (2H, dd, *J* = 15.4, 1.0 Hz, OCH_2_O), 6.01 (1H, qq, *J* = 7.2, 1.5 Hz, 26-H), 4.44 (1H, br d, *J* = 2.8 Hz, 22-H_α_), 4.15 (1H, t, *J* = 4.9 Hz, 1-H), 4.11 (1H, dd, *J* = 11.5, 2.8 Hz, 22-H_β_), 3.99 (1H, d, *J* = 2.4 Hz, 21-H), 3.98 (1H, overlapped, 11-H), 3.98 (3H, s, 17-OCH_3_), 3.53 (1H, ddd, *J* = 7.4, 2.4, 1.6 Hz, 13-H), 3.17 (1H, ddd, *J* = 11.7, 5.0, 2.5 Hz, 3-H), 2.78 (1H, dd, *J* = 21.1, 7.4 Hz, 14-H_α_), 2.56 (1H, dd, *J* = 15.2, 2.5 Hz, 4-H_α_), 2.33 (1H, overlapped, 14-H_β_), 2.32 (3H, s, 2'-CH_3_), 2.30 (3H, s, NCH_3_), 2.00 (3H, s, 6-CH_3_), 1.88 (3H, s, 16-CH_3_), 1.80 (3H, dq, *J* = 7.2, 1.5 Hz, 27-CH_3_), 1.60 (3H, dq, *J* = 1.5, 1.5 Hz, 28-CH_3_), 1.59 (1H, overlapped, 4-H_β_).

# Physical and spectroscopic data of **3b**

*5-O-(propanoyl)* *ester derivative of renieramycin T,* **3b**: The title compound was synthesized from **2** (25.0 mg, 0.04 mmol), DMAP (13.3 mg, 0.11 mmol), EDCI (20.5 mg, 0.11 mmol) and propionyl chloride (0.02 mL, 0.22 mmol) to afford **3b**; yield 39% (brsm); yellow amorphous powder; $[]_{D}^{25}$ +16.5 (*c* 0.34, CHCl_3_); ECD Δ*ε* (*c* 31.66 μM, methanol, 20 ^o^C) −2.1 (288), +9.7 (257), −4.4 (222), +5.6 (205), −7.5 (203), +12.6 (201), +14.9 (197), −3.3 (193), −29.7 (191) nm; IR (ATR) ν_max_ 2923, 1755, 1713, 1650, 1614, 1455, 1230, 1143, 1087, 953, 733 cm^−1^; ^1^H NMR (CDCl_3_, 400 MHz) δ 6.01 (1H, overlapped, 26-H), 5.99 (2H, dd, *J* = 25.7, 1.4, Hz, OCH_2_O), 4.58 (1H, dd, *J* = 11.5, 3.2 Hz, 22-H_α_), 4.18 (1H, t, *J* = 3.2 Hz, 1-H), 4.14 (1H, d, *J* = 2.4 Hz, 21-H), 4.04 (1H, dd, *J* = 11.5, 4.3 Hz, 22-H_β_), 4.01 (1H, d, *J* = 2.7 Hz, 11-H), 3.98 (3H, s, 17-OCH_3_), 3.39 (1H, d, *J* = 7.5 Hz, 13-H), 3.25 (1H, dt, *J* = 12.2, 2.7 Hz, 3-H), 2.77 (1H, dd, *J* = 20.9, 7.5 Hz, 14-H_α_), 2.64 (2H, q, *J* = 7.6 Hz, 2'-H), 2.54 (1H, dd, *J* = 15.1, 2.7 Hz, 4-H_α_), 2.34 (1H, d, *J* = 20.9 Hz, 14-H_β_), 2.29 (3H, s, NCH_3_), 2.03 (3H, s, 6-CH_3_), 1.94 (3H, s, 16-CH_3_), 1.88 (3H, dq, *J* = 7.3, 1.5 Hz, 27-CH_3_), 1.69 (3H, dq, *J* = 1.5, 1.5 Hz, 28-CH_3_), 1.64 (1H, overlapped, 4-H_β_), 1.34 (3H, t, *J* = 7.6 Hz, 3'-CH_3_); ^13^C NMR (CDCl_3_, 100 MHz) δ 185.9 (C-15), 182.7 (C-18), 171.2 (C-1'), 167.0 (C-24), 155.4 (C-17), 144.9 (C-7), 141.7 (C-20), 140.7 (C-8), 140.4 (C-5), 140.2 (C-26), 135.4 (C-19), 128.9 (C-16), 126.7 (C-25), 120.1 (C-6), 117.3 (21-CN), 112.2 (C-10), 112.0 (C-9), 101.7 (OCH_2_O), 63.4 (C-22), 60.7 (17-OCH_3_), 59.1 (C-21), 56.4 (C-1), 55.5 (C-3), 54.8 (C-13), 54.7 (C-11), 41.4 (NCH_3_), 27.8 (C-4), 27.3 (C-2'), 21.1 (C-14), 20.5 (28-CH_3_), 15.9 (27-CH_3_), 9.4 (6-CH_3_), 9.4 (3'-CH_3_), 8.6 (16-CH_3_); HRESIMS *m/z* 632.2603 ([M+H]^+^, calculated for C_34_H_38_N_3_O_9_, 632.2603).


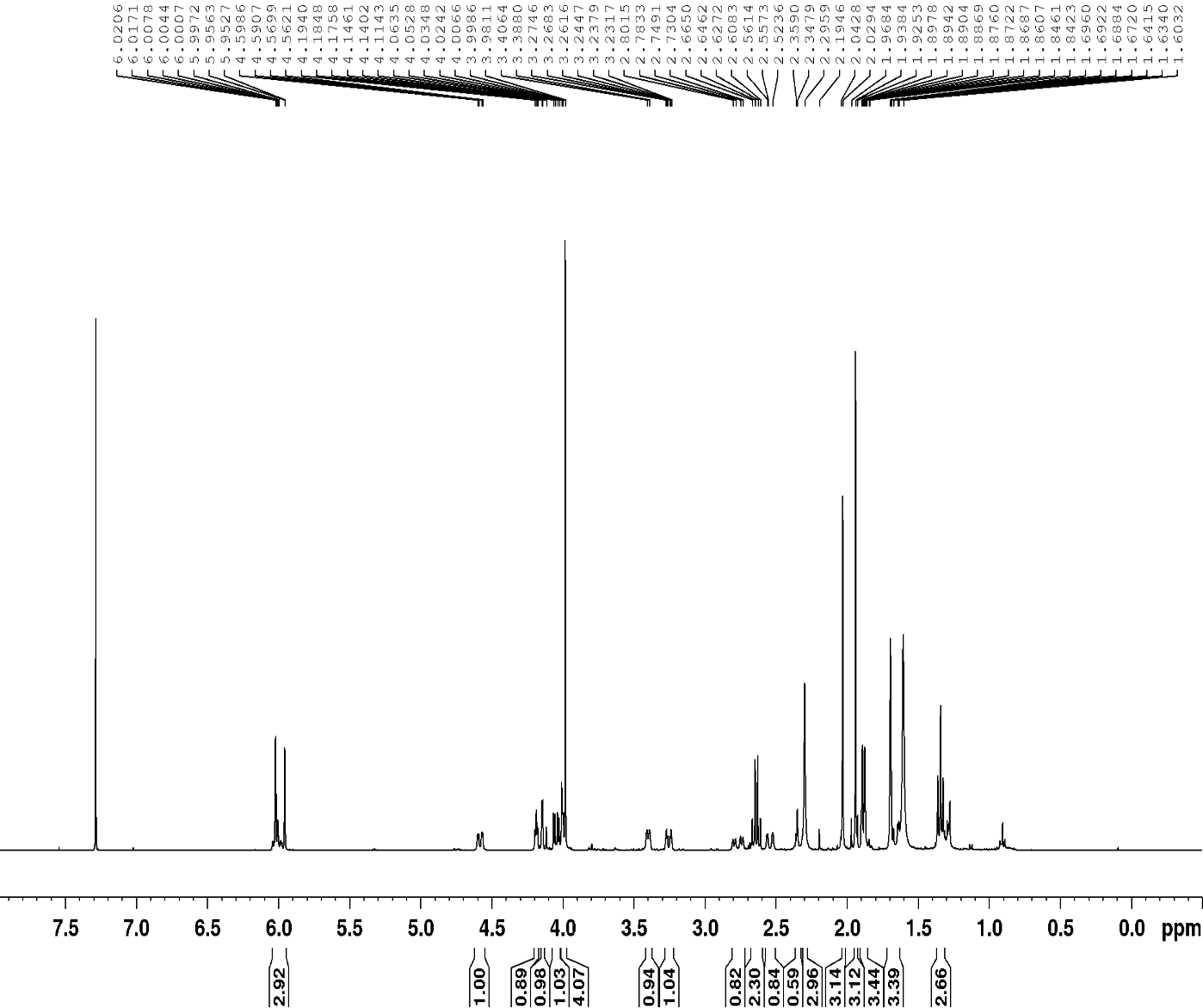


g, h

g, h

g: grease, h: hexane

**Figure S7.** ^1^H NMR (400 MHz) spectrum of **3b** in CDCl_3_

**
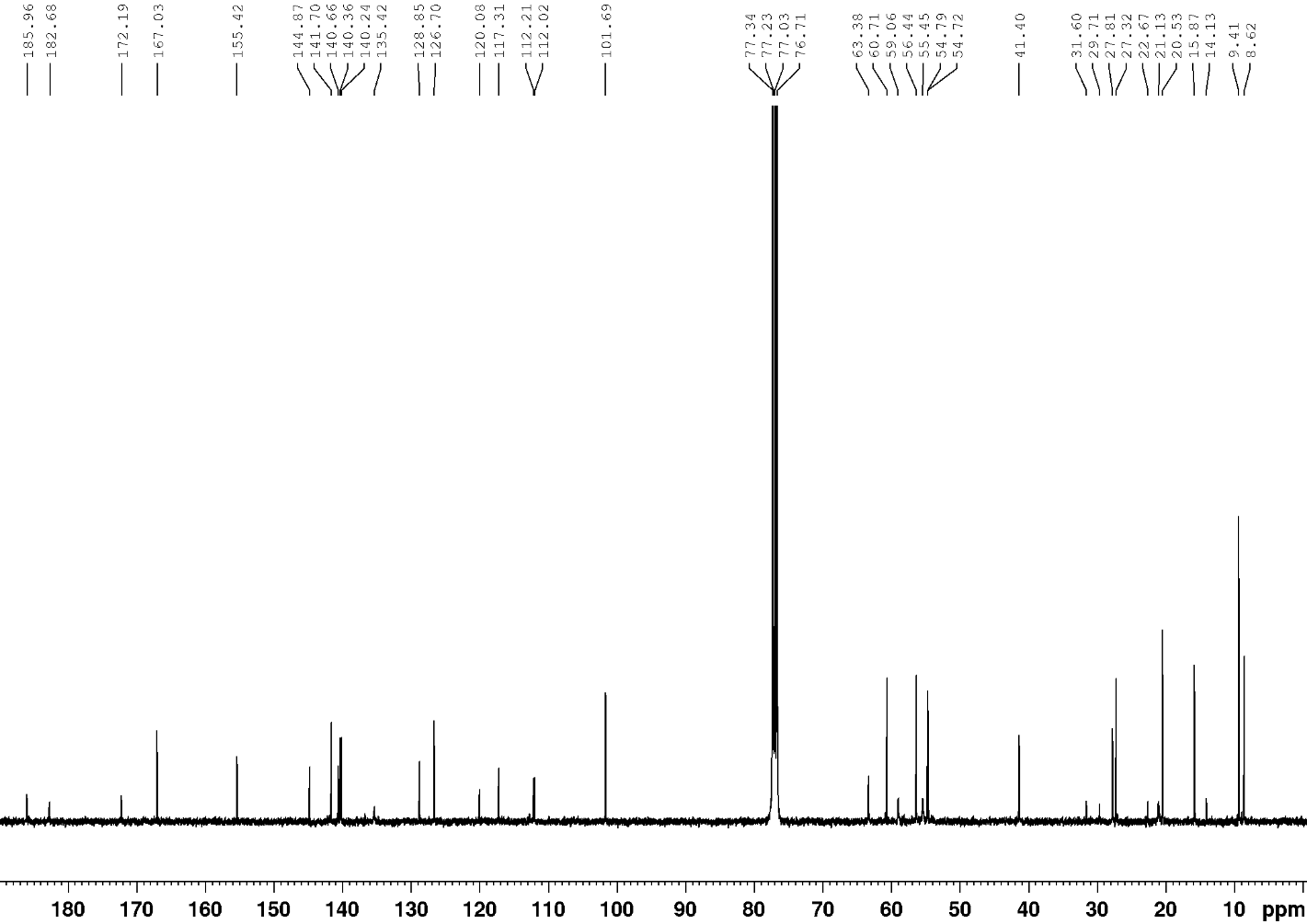
**

g

h

h

h

g: grease, h: hexane

**Figure S8.** ^13^C NMR (100 MHz) spectrum of **3b** in CDCl_3_

**
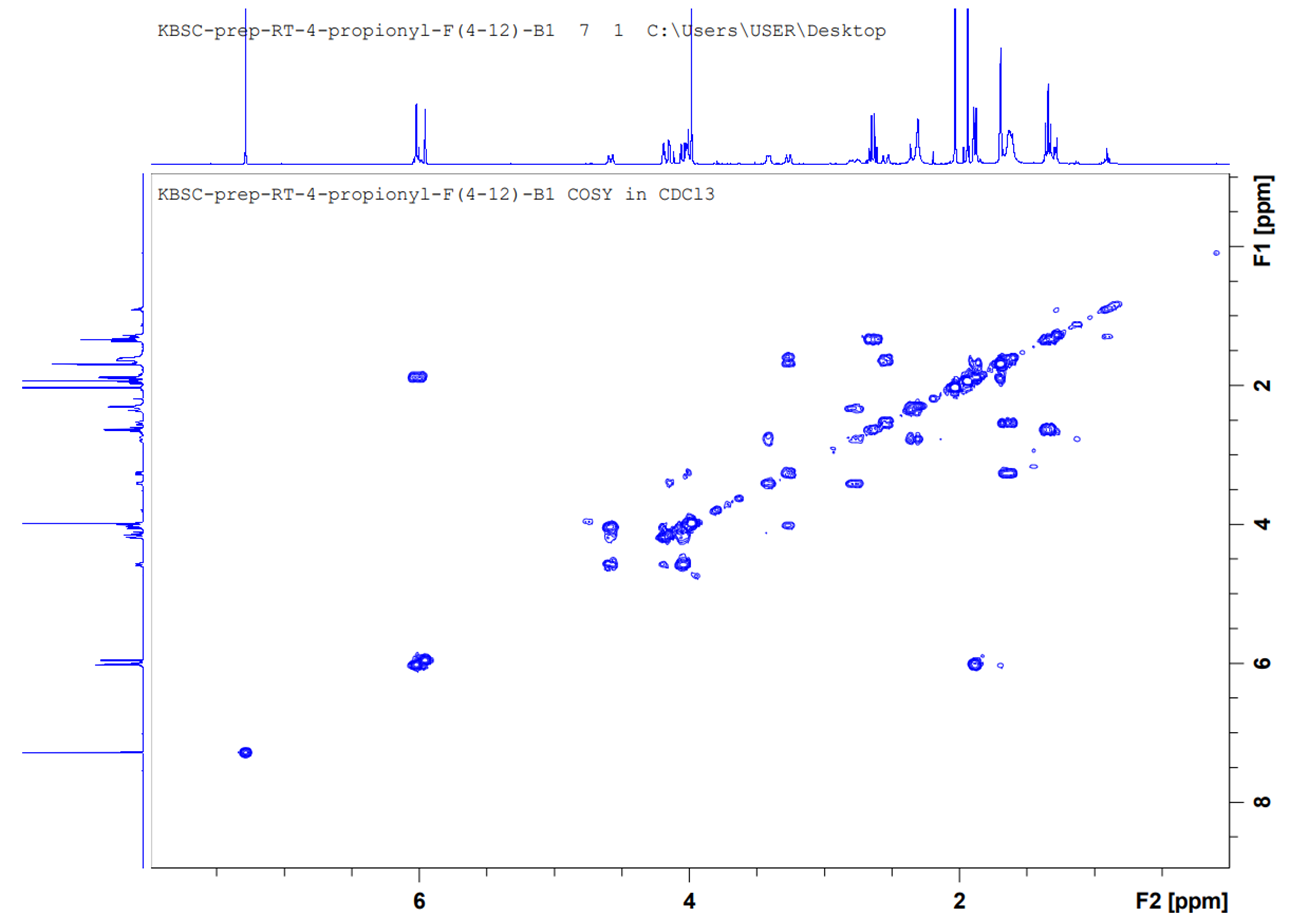
**

**Figure S9.** COSY (400 MHz) spectrum of **3b** in CDCl_3_


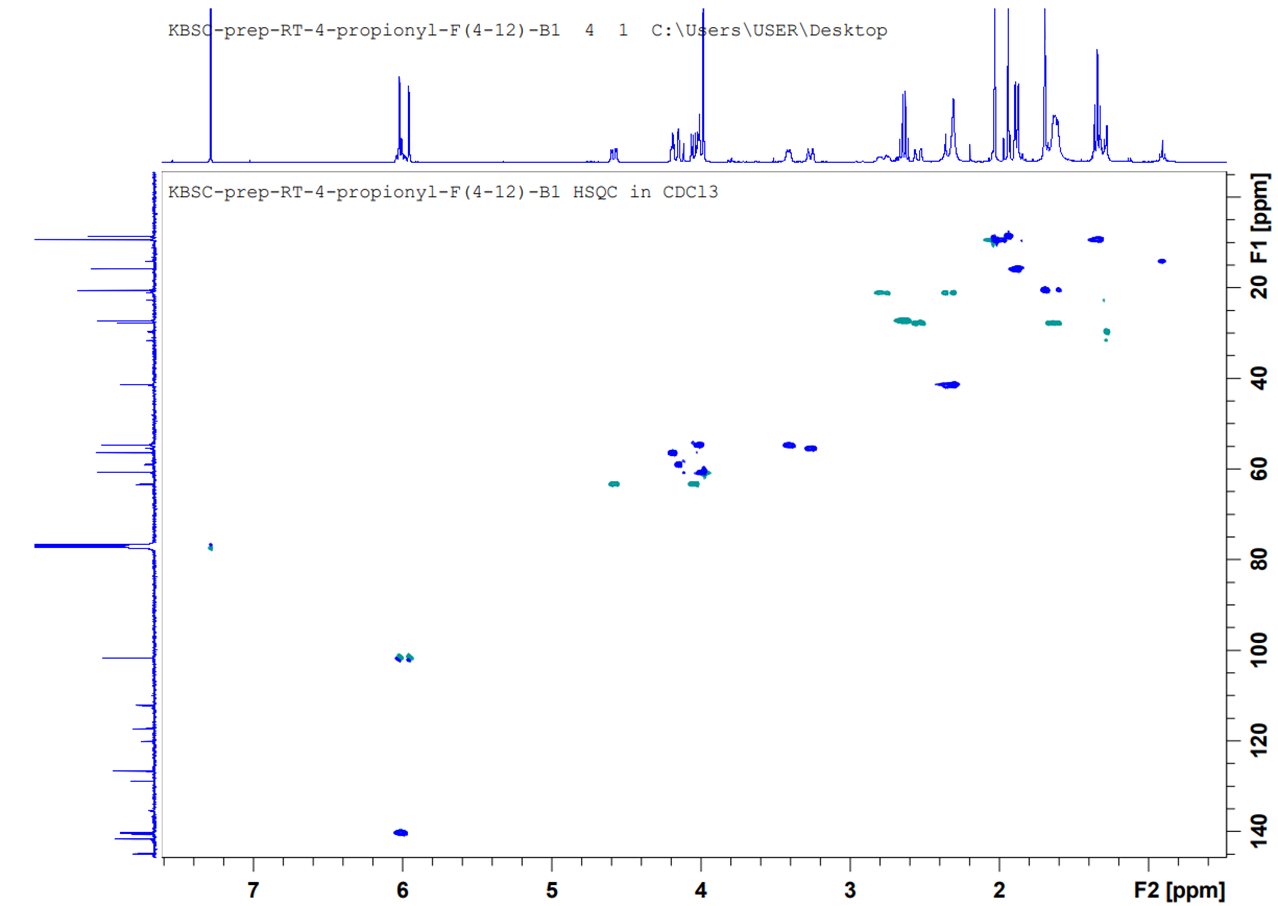


**Figure S10.** HSQC (400 MHz) spectrum of **3b** in CDCl_3_


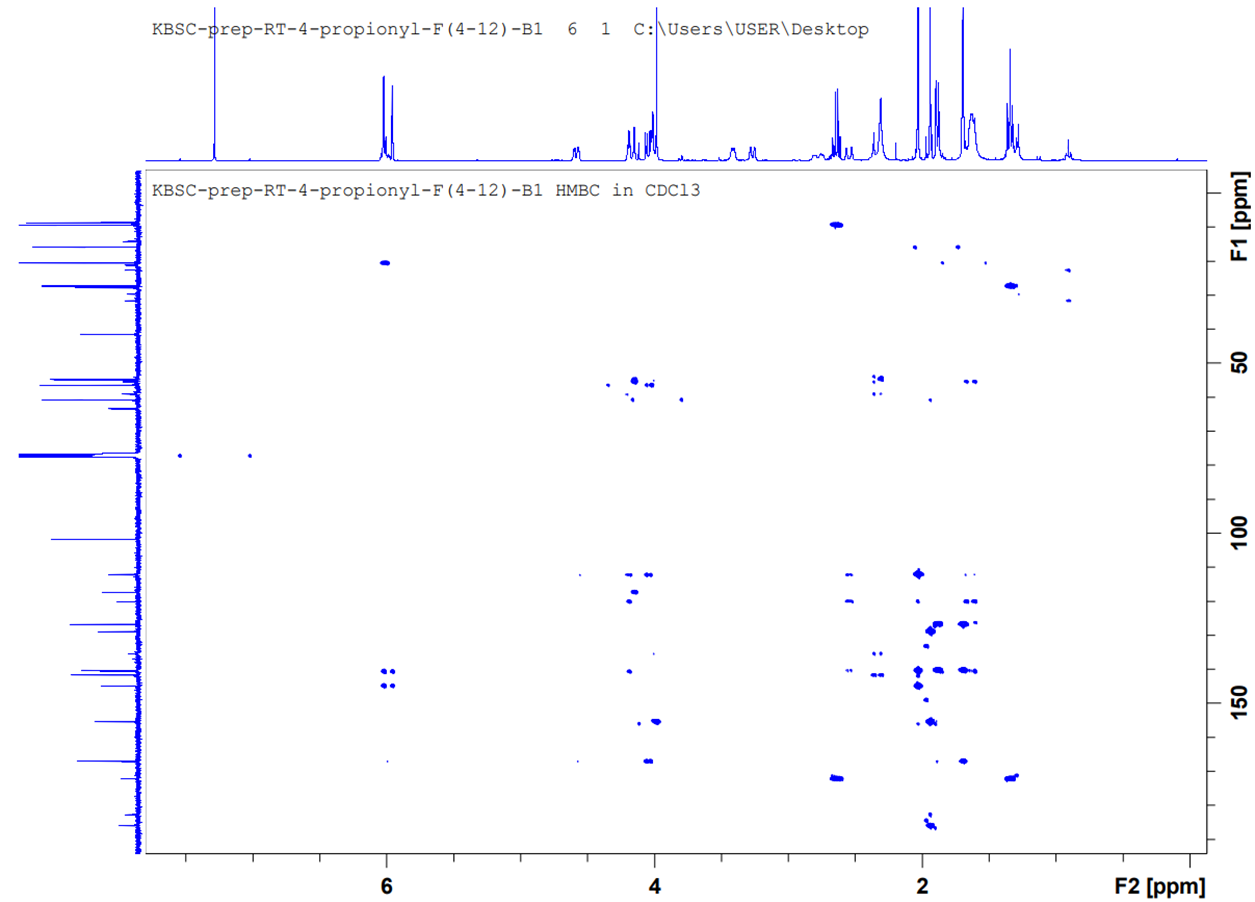


**Figure S11.** HMBC (400 MHz) spectrum of **3b** in CDCl_3_

# Physical and spectroscopic data of **3c**

*5-O-(N-(tert-butoxycarbonyl)-L-glycinoyl) ester derivative of renieramycin T,* **3c**: The title compound was synthesized from **2** (21.8 mg, 0.04 mmol), DMAP (5.6 mg, 0.05 mmol), EDCI (8.6 mg, 0.05 mmol) and *N*-Boc-*L*-glycine (33.2 mg, 0.19 mmol) to afford **3c**; yield 52% (brsm); yellow amorphous powder;$[]_{D}^{25}$ −4.2 (*c* 0.49, CHCl_3_); ECD Δ*ε* (*c* 32.75 μM, methanol, 20 ^o^C) −1.6 (295), +8.5 (261), −4.5 (223), +13.4 (207), −1.3 (202), +16.0 (199), +23.5 (193), −10.4 (191) nm; IR (ATR) ν_max_ 3419 (br), 2951, 1645, 1453, 1018, 719 cm^−1^; ^1^H NMR (CDCl_3_, 400 MHz) δ 5.98 (1H, overlapped, 26-H), 5.97 (2H, dd, *J* = 26.8, 1.2 Hz, OCH_2_O), 5.11 (1H, br s, 3'-NH), 4.58 (1H, dd, *J* = 11.6, 4.9 Hz, 22-H_α_), 4.22 (2H, d, *J* = 4.9 Hz, 2'-H), 4.16 (1H, dd, *J* = 4.9, 4.0 Hz, 1-H), 4.12 (1H, d, *J* = 2.1 Hz, 21-H), 4.02 (1H, dd, *J* = 11.6, 4.0 Hz, 22-H_β_), 3.99 (1H, overlapped, 11-H), 3.96 (3H, s, 17-OCH_3_), 3.38 (1H, dd, *J* = 7.2, 2.1 Hz, 13-H), 3.22 (1H, dt, *J* = 12.0, 2.4 Hz, 3-H), 2.75 (1H, dd, *J* = 20.8, 7.2 Hz, 14-H_α_), 2.48 (1H, overlapped, 4-H_α_), 2.32 (1H, d, *J* = 20.8 Hz, 14-H_β_), 2.28 (3H, s, NCH_3_), 2.02 (3H, s, 6-CH_3_), 1.92 (3H, s, 16-CH_3_), 1.85 (3H, dq, *J* = 7.2, 1.6 Hz, 27-CH_3_), 1.65 (3H, s, 28-CH_3_), 1.61 (1H, overlapped, 4-H_β_), 1.48 (9H, br s, 3 × 7'-CH_3_); ^13^C NMR (CDCl_3_, 100 MHz) δ 185.9 (C-15), 182.7 (C-18), 168.4 (C-1'), 167.0 (C-24), 155.5 (C-4'), 155.4 (C-17), 144.9 (C-7), 141.8 (C-20), 140.9 (C-8), 140.3 (C-26), 140.0 (C-5), 135.2 (C-19), 129.0 (C-16), 126.6 (C-25), 119.8 (C-6), 117.2 (21-CN), 112.2 (C-10), 112.1 (C-9), 101.8 (OCH_2_O), 80.3 (C-6'), 63.0 (C-22), 60.8 (17-OCH_3_), 58.8 (C-21), 56.4 (C-1), 55.2 (C-3), 54.7 (C-11), 54.7 (C-13), 42.1 (C-2'), 41.4 (NCH_3_), 28.3 (3 × 7'-CH_3_), 28.0 (C-4), 21.2 (C-14), 20.5 (28-CH_3_), 15.8 (27-CH_3_), 9.4 (6-CH_3_), 8.7 (16-CH_3_); HRESIMS *m/z* 733.3077 ([M+H]^+^, calculated for C_38_H_45_N_4_O_11_, 733.3079).


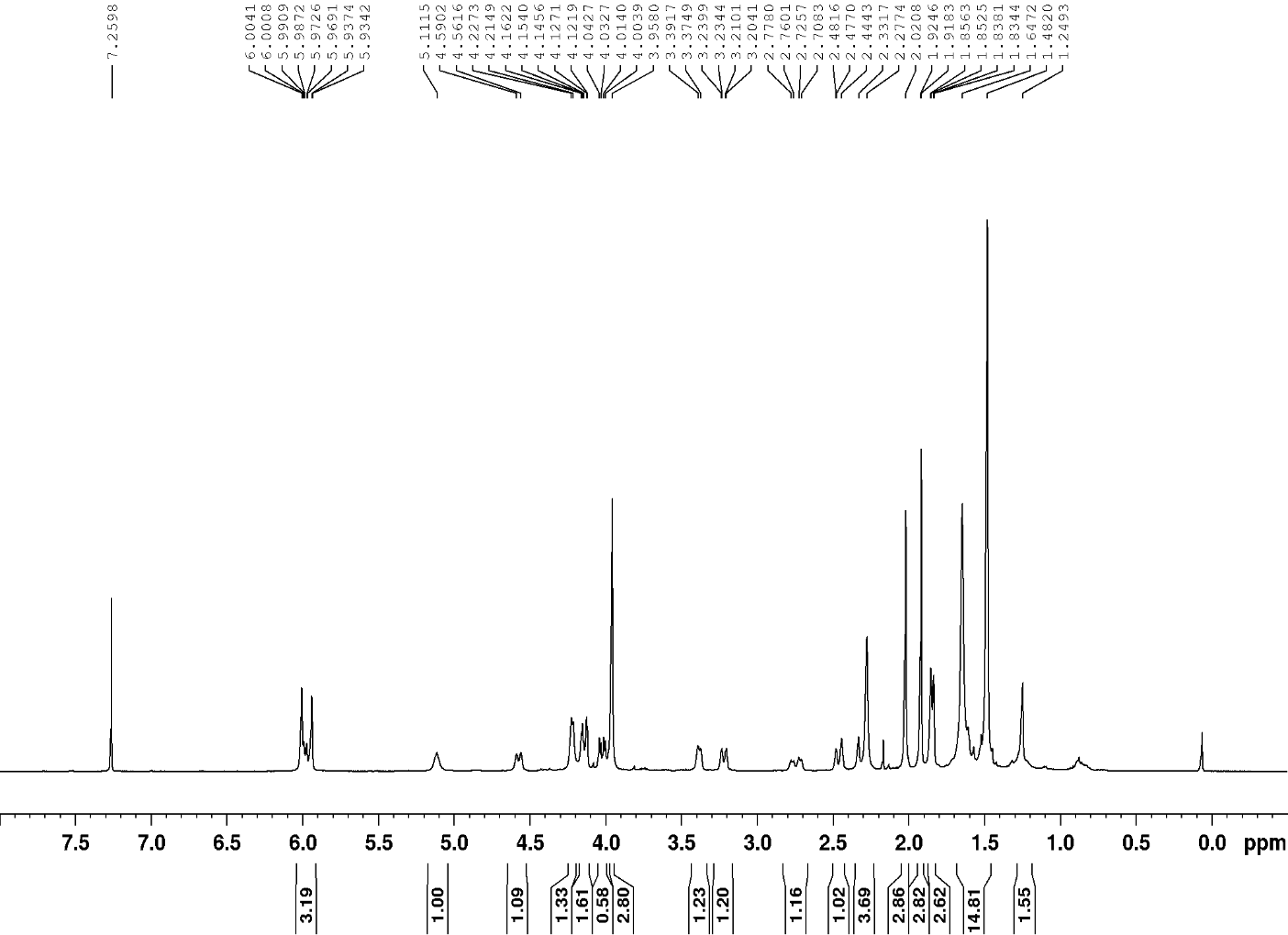


g, h

g, h

g: grease, h: hexane

**Figure S12.** ^1^H NMR (400 MHz) spectrum of **3c** in CDCl_3_


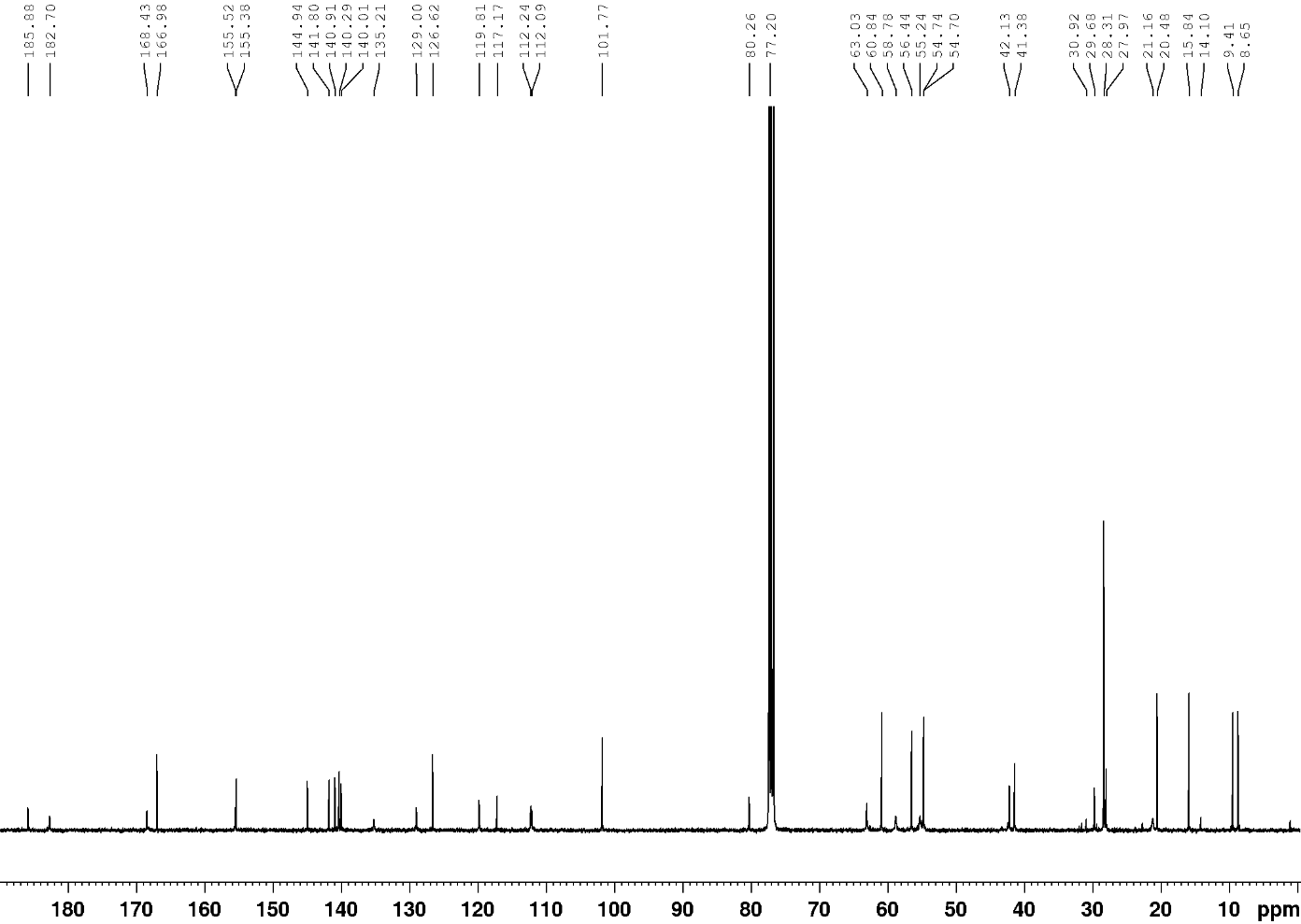


h

h

h

g

g: grease, h: hexane

**Figure S13.** ^13^C NMR (100 MHz) spectrum of **3c** in CDCl_3_

***
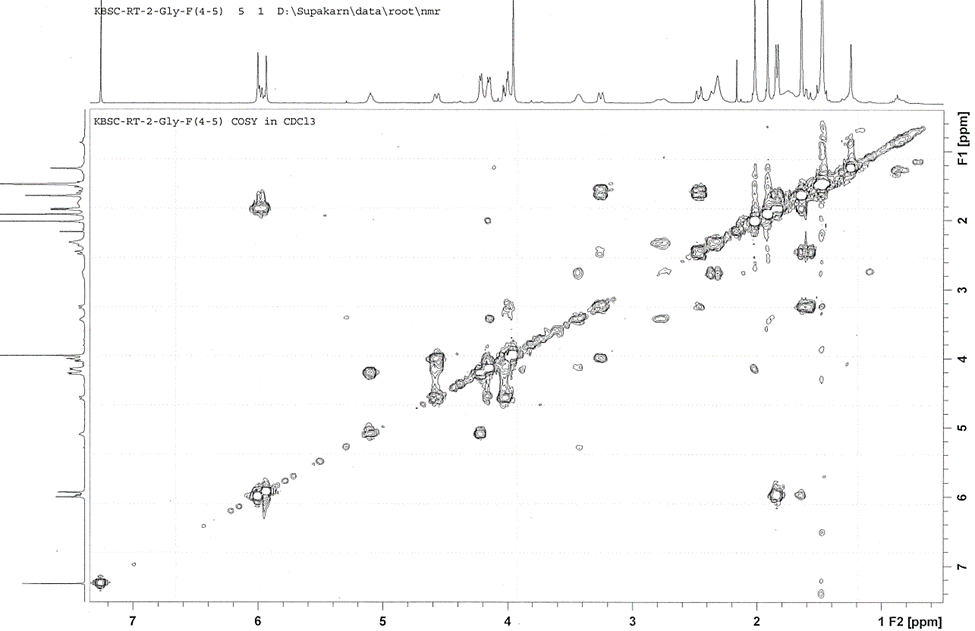
***

**Figure S14.** COSY (400 MHz) spectrum of **3c** in CDCl_3_


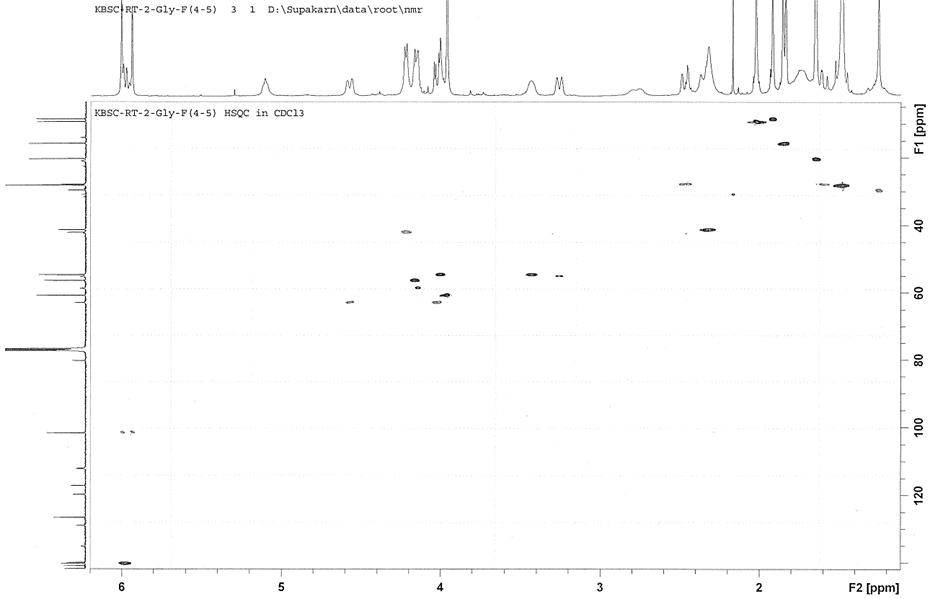


**Figure S15.** HSQC (400 MHz) spectrum of **3c** in CDCl_3_


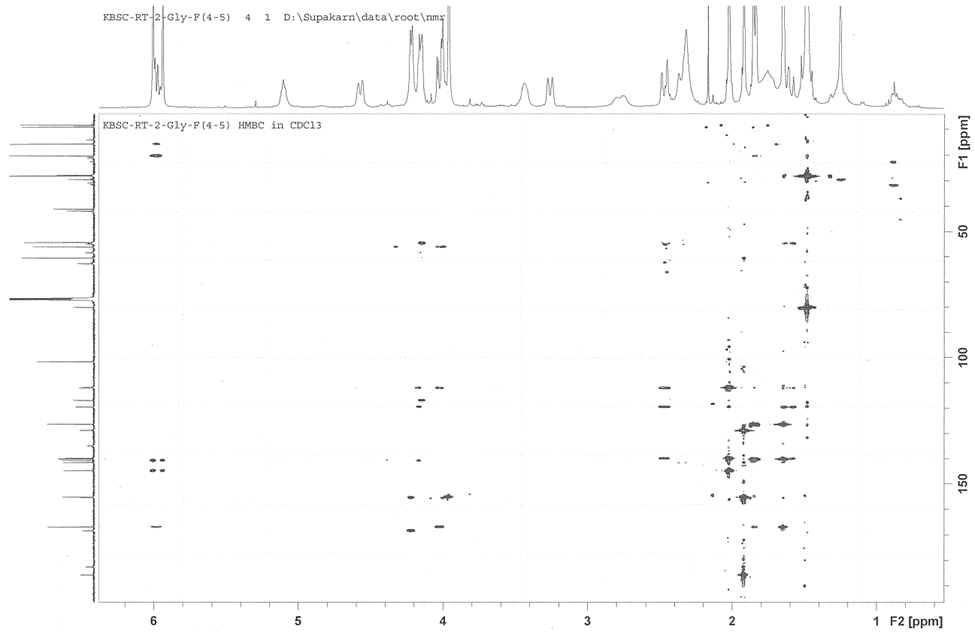


**Figure S16.** HMBC (400 MHz) spectrum of **3c** in CDCl_3_

# Physical and spectroscopic data of **3d**

*5-O-(N-(tert-butoxycarbonyl)-L-alaninoyl) ester derivative of renieramycin T,* **3d**: The title compound was synthesized from **2** (13.7 mg, 0.02 mmol), DMAP (2.9 mg, 0.02 mmol), EDCI (4.6 mg, 0.02 mmol) and *N*-Boc-*L*-alanine (6.8 mg, 0.04 mmol) to afford **3d**; yield 35% (brsm); yellow amorphous powder^35^;$[]_{D}^{25}$ +68.2 (*c* 0.090, CHCl_3_); ECD Δ*ε* (*c* 46.46 μM, methanol, 20 ^o^C) −11.9 (355), +29.9 (260), −21.5 (222), +29.7 (216), +52.2 (210), +10.3 (206), −36.2 (198), +30.8 (197), +15.0 (194), −19.8 (193), +43.5 (191) nm; IR (ATR) ν_max_ 3401 (br), 2924, 2851, 1715, 1654, 1457, 1410, 1377, 1306, 1234, 1151, 1094, 1044, 956, 770, 733 cm^−1^; ^1^H NMR (CDCl_3_, 400 MHz) δ 5.98 (1H, overlapped, 26-H), 5.97 (2H, dd, *J* = 22.4, 1.2 Hz, OCH_2_O), 5.01 (1H, br d, *J* = 7.2 Hz, 3'-NH), 4.59 (1H, t, *J* = 6.8 Hz, 2'-H), 4.53 (1H, dd, *J* = 11.6, 3.6 Hz, 22-H_α_), 4.16 (1H, overlapped, 1-H), 4.11 (1H, overlapped, 21-H), 4.02 (1H, dd, *J* = 11.6, 4.4, 22-H_β_), 3.97 (1H, overlapped, 11-H), 3.96 (3H, s, 17-OCH_3_), 3.36 (1H, d, *J* = 7.6 Hz, 13-H), 3.23 (1H, dt, *J* = 12.4, 2.8 Hz, 3-H), 2.73 (1H, dd, *J* = 20.8, 7.6 Hz, 14-H_α_), 2.55 (1H, m, 4-H_α_), 2.32 (1H, dd, *J* = 15.6, 7.6 Hz, 14-H_β_), 2.28 (3H, s, NCH_3_), 2.04 (3H, s, 6-CH_3_), 1.90 (3H, s, 16-CH_3_), 1.85 (3H, dq, *J* = 7.6, 1.2 Hz, 27-CH_3_), 1.70 (3H, d, *J* = 7.2, 8'-CH_3_), 1.66 (3H, s, 28-CH_3_), 1.61 (1H, overlapped, 4-H_β_), 1.46 (9H, br s, 3 × 7'-CH_3_); ^13^C NMR (CDCl_3_, 100 MHz) δ 186.0 (C-15), 182.7 (C-18), 171.2 (C-1'), 167.0 (C-24), 158.3 (C-4'), 155.2 (C-17), 144.9 (C-7), 141.9 (C-20), 140.9 (C-8), 140.2 (C-26), 139.9 (C-5), 135.3 (C-19), 129.0 (C-16), 126.7 (C-25), 119.9 (C-6), 117.3 (21-CN), 112.3 (C-10), 112.1 (C-9), 101.8 (OCH_2_O), 80.1 (C-6'), 63.6 (C-22), 60.9 (17-OCH_3_), 59.2 (C-21), 56.3 (C-1), 55.5 (C-3), 54.8 (C-11), 54.7 (C-13), 49.3 (C-2'), 41.4 (NCH_3_), 28.3 (3 × 7'-CH_3_), 27.6 (C-4), 21.0 (C-14), 20.5 (28-CH_3_), 18.6 (8'-CH_3_), 15.9 (27-CH_3_), 9.5 (6-CH_3_), 8.6 (16-CH_3_); HRESIMS *m/z* 747.3231 ([M+H]^+^, calculated for C_39_H_47_N_4_O_11_, 747.3236).


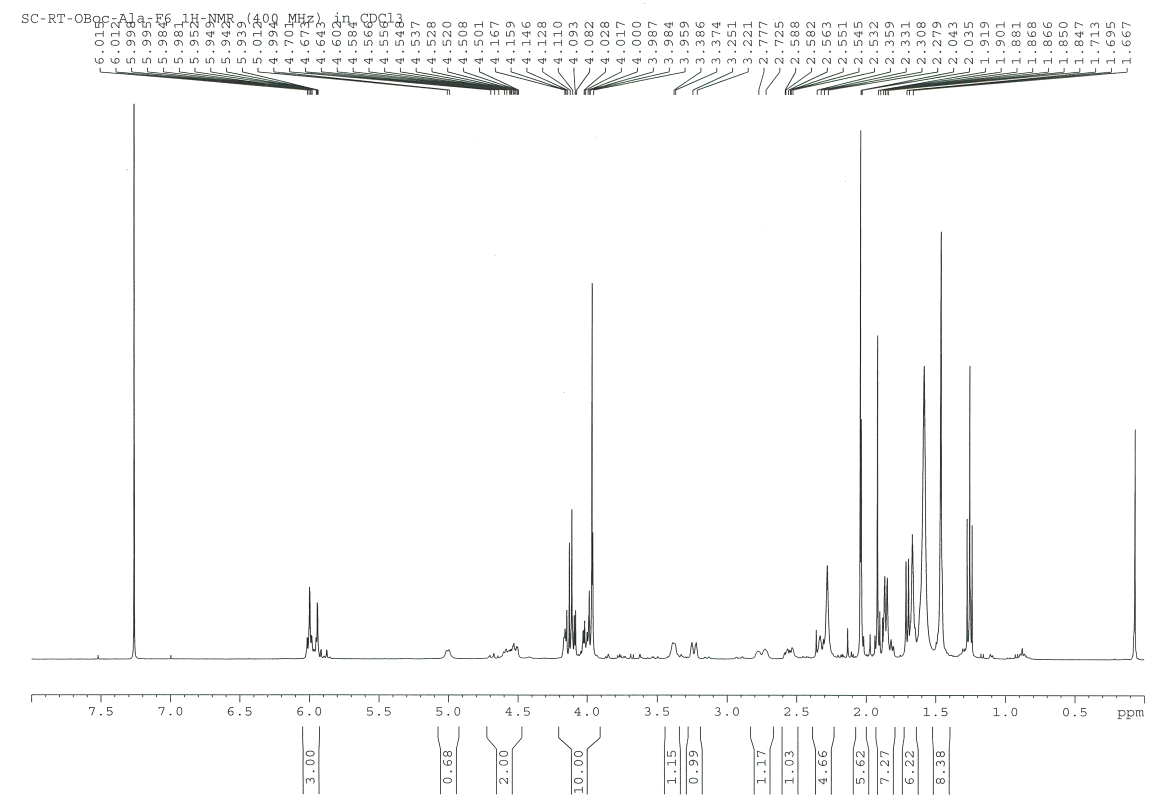


**Figure S17.** ^1^H NMR (400 MHz) spectrum of **3d** in CDCl_3_


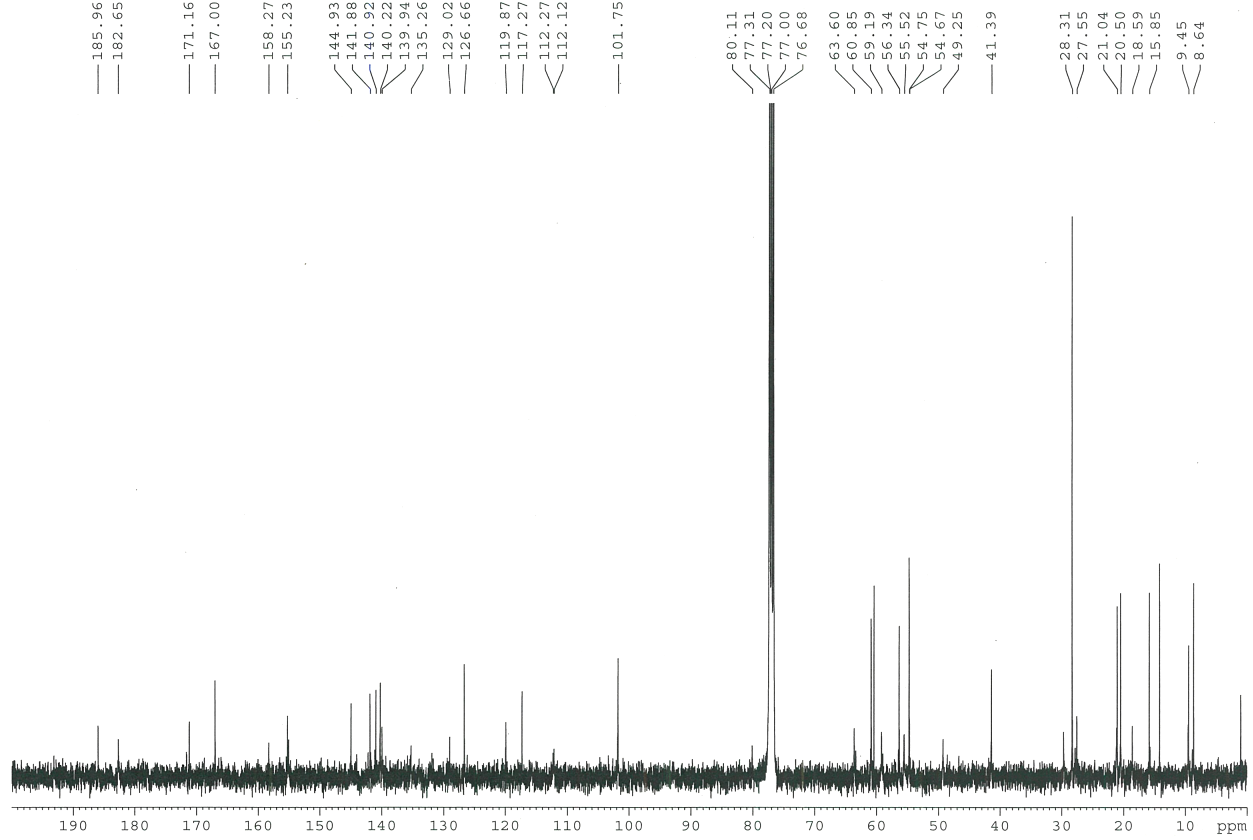


**Figure S18.** ^13^C NMR (100 MHz) spectrum of **3d** in CDCl_3_


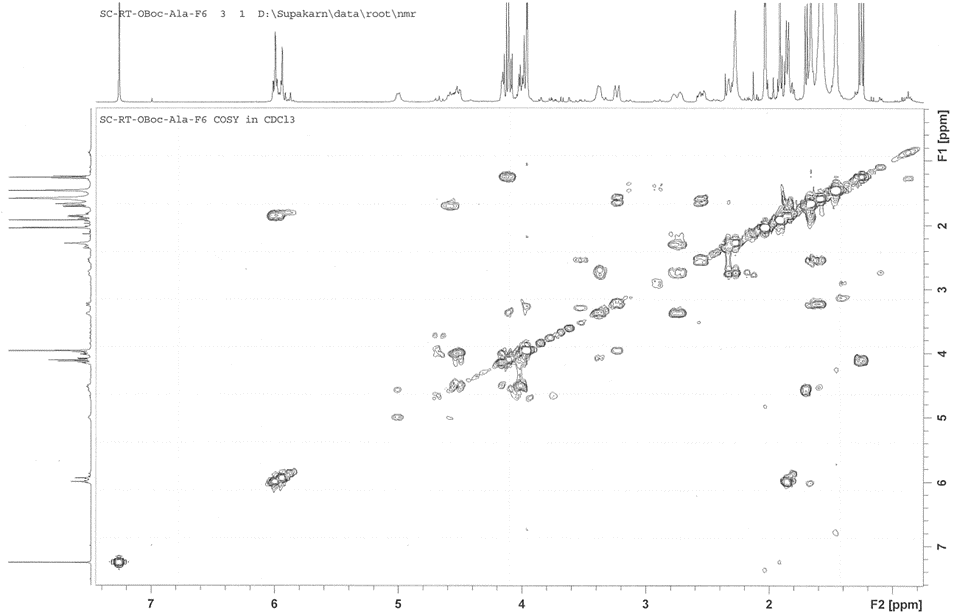


**Figure S19.** COSY (400 MHz) spectrum of **3d** in CDCl_3_


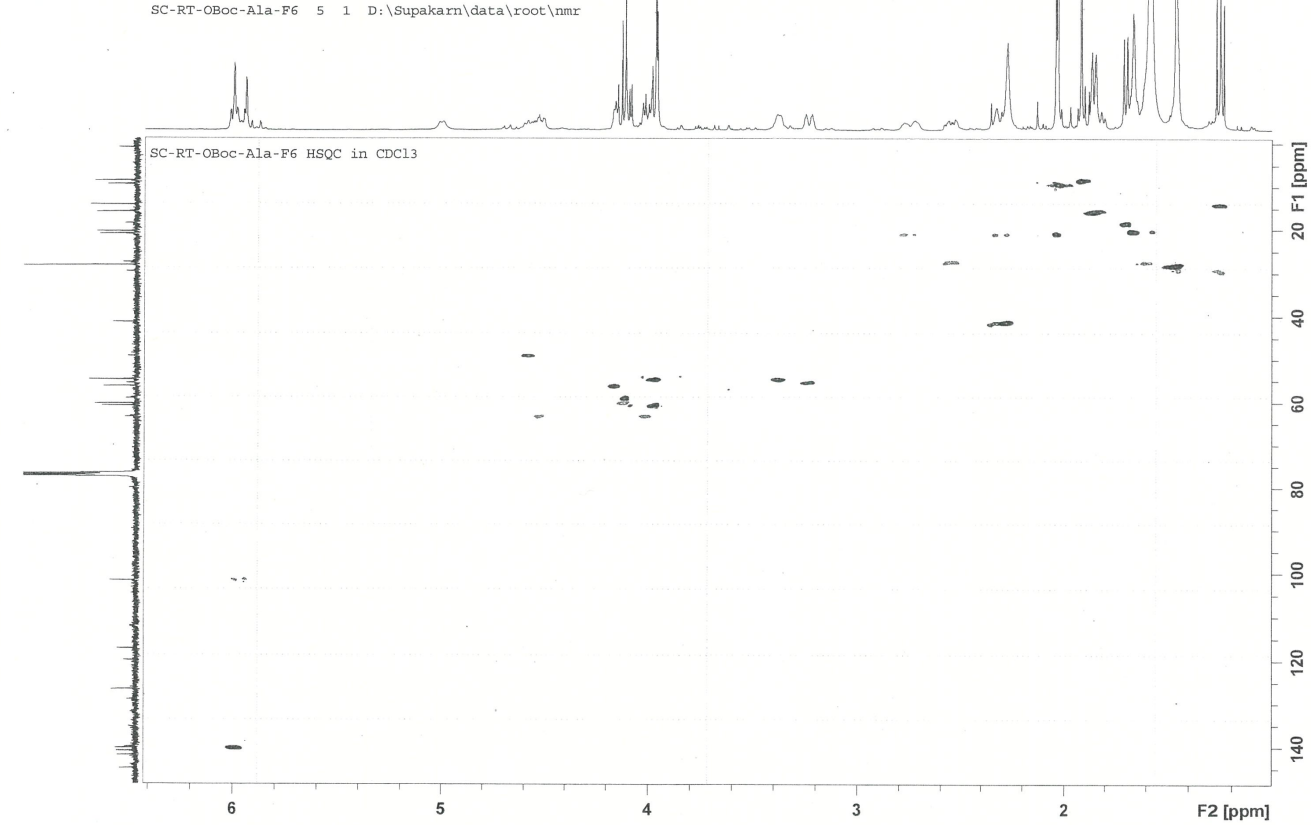


**Figure S20.** HSQC (400 MHz) spectrum of **3d** in CDCl_3_


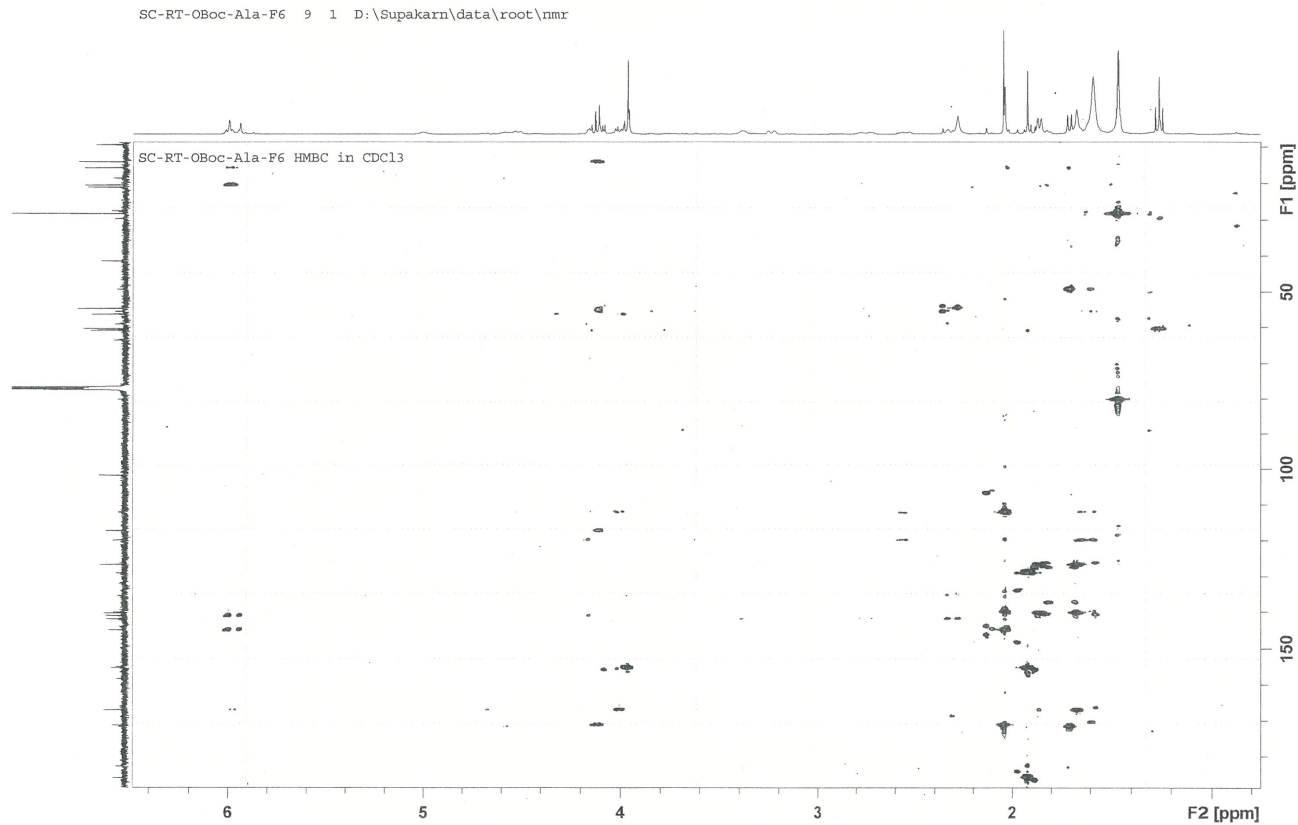


**Figure S21.** HMBC (400 MHz) spectrum of **3d** in CDCl_3_

# Physical and spectroscopic data of **3e**

*5-O-(N-(tert-butoxycarbonyl)-L-valinoyl) ester derivative of renieramycin T,* **3e**: The title compound was synthesized from **2** (25.0 mg, 0.04 mmol), DMAP (13.3 mg, 0.11 mmol), EDCI (20.5 mg, 0.11 mmol) and *N*-Boc-*L*-valine (47.2 mg, 0.22 mmol) to afford **3e**; yield 76% (brsm); yellow amorphous powder; $[]_{D}^{25}$ −5.2 (*c* 0.58, CHCl_3_); ECD Δ*ε* (*c* 31.54 μM, methanol, 20 ^o^C) +4.9 (260), −3.9 (223), +6.6 (211), +2.2 (203), −33.9 (201), +0.5 (196), −14.2 (195), +18.4 (193) nm; IR (ATR) ν_max_ 3356 (br), 2921, 2852, 1713, 1652, 1455, 1373, 1230, 1142, 1079, 953, 770 cm^−1^; ^1^H NMR (CDCl_3_, 400 MHz) δ 5.98 (1H, overlapped, 26-H), 5.98 (2H, dd, *J* = 28.4, 1.2 Hz, OCH_2_O), 4.97 (1H, br d, *J* = 9.6 Hz, 3'-NH), 4.50 (1H, dd, *J* = 9.6, 3.2 Hz, 2'-H), 4.42 (1H, dd, *J* = 11.6, 3.2 Hz, 22-H_α_), 4.16 (1H, d, *J* = 3.2 Hz, 1-H), 4.13 (1H, overlapped, 11-H), 4.03 (1H, overlapped, 21-H), 4.01 (1H, dd, *J* = 11.6, 3.2 Hz, 22-H_β_), 3.96 (3H, s, 17-OCH_3_), 3.41 (1H, br s, 13-H), 3.27 (1H, dd, *J* = 12.2, 2.3 Hz, 3-H), 2.74 (1H, d, *J* = 15.0 Hz, 14-H_α_), 2.57 (1H, dd, *J* = 15.2, 2.3 Hz, 4-H_α_), 2.44 (1H, dq, *J* = 6.8, 3.2 Hz, 8'-H), 2.32 (1H, overlapped, 14-H_β_), 2.27 (3H, s, NCH_3_), 2.01 (3H, s, 6-CH_3_), 1.91 (3H, s, 16-CH_3_), 1.84 (3H, dq, *J* = 7.2, 1.6 Hz, 27-CH_3_), 1.68 (3H, dq, *J* = 7.3, 1.6 Hz, 28-CH_3_), 1.47 (9H, s, 3 × 7'-CH_3_), 1.66 (1H, overlapped, 4-H_β_), 1.18 (3H, d, *J* = 6.8 Hz, 9'-CH_3_), 1.07 (3H, d, *J* = 6.8 Hz, 10'-CH_3_); ^13^C NMR (CDCl_3_, 100 MHz) δ 185.6 (C-15), 182.0 (C-18), 170.8 (C-1'), 167.0 (C-24), 158.3 (C-4'), 155.2 (C-17), 145.1 (C-7), 141.9 (C-20), 141.0 (C-8), 140.2 (C-26), 139.3 (C-5), 134.3 (C-19), 128.9 (C-16), 126.6 (C-25), 119.7 (C-6), 117.0 (21-CN), 112.2 (C-10), 111.9 (C-9), 101.8 (OCH_2_O), 80.1 (C-6'), 64.3 (C-22), 60.9 (17-OCH_3_), 58.8 (C-2'), 58.2 (C-21), 56.3 (C-1), 56.2 (C-3), 54.9 (C-11), 54.7 (C-13), 41.4 (NCH_3_), 30.7 (C-8'), 28.3 (3 × 7'-CH_3_), 27.2 (C-4), 20.5 (28-CH_3_), 20.4 (C-14), 19.8 & 17.4 (9'-CH_3_ & 10'-CH_3_), 15.8 (27-CH_3_), 9.5 (6-CH_3_), 8.7 (16-CH_3_); HRESIMS *m/z* 775.3549 ([M+H]^+^, calculated for C_41_H_51_N_4_O_11_, 775.3549).


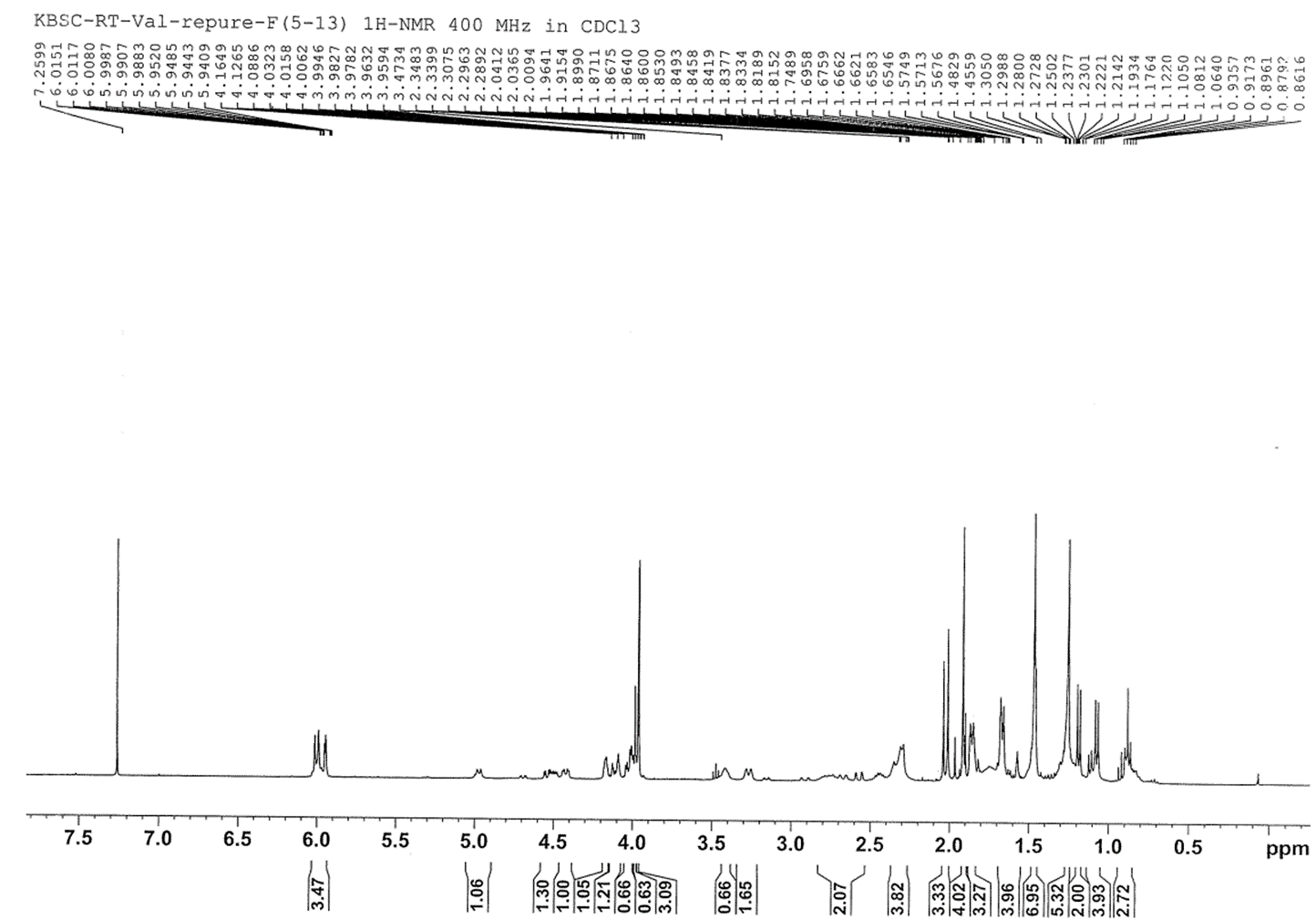


g, h

g, h

g: grease, h: hexane

**Figure S22.** ^1^H NMR (400 MHz) spectrum of **3e** in CDCl_3_


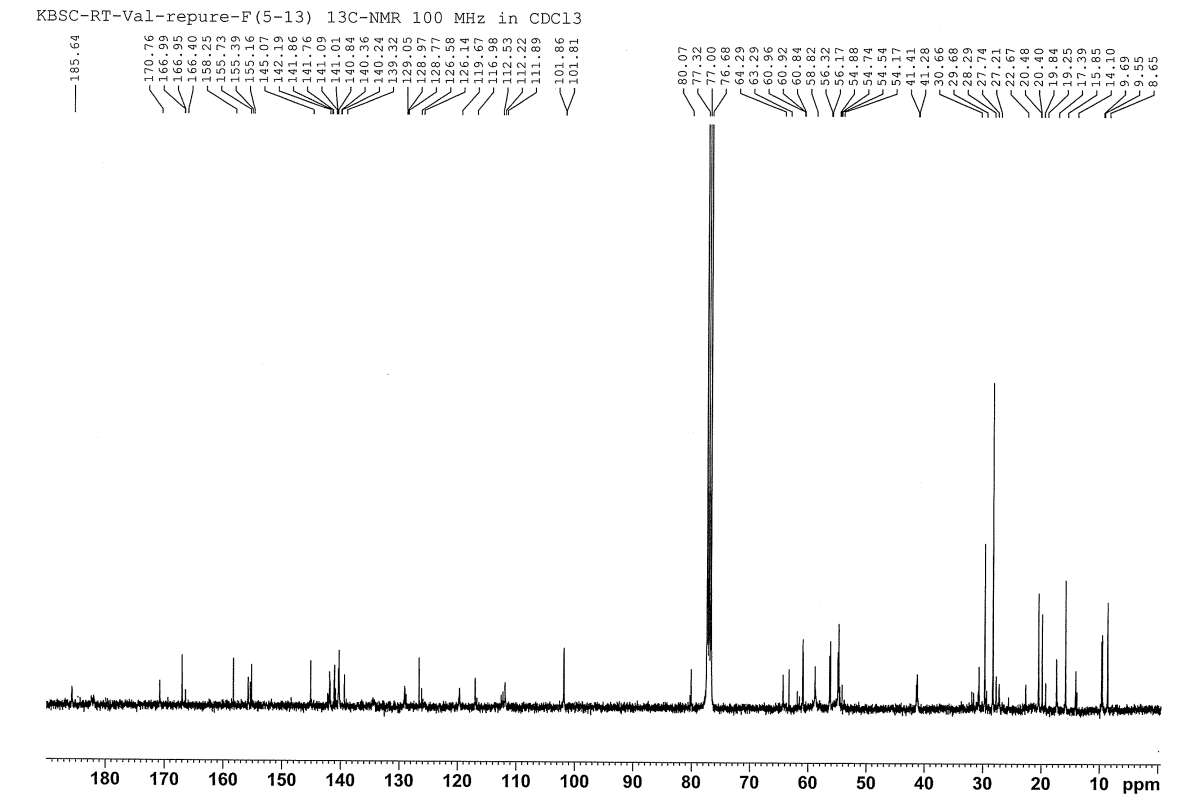


h

h

h

g

g: grease, h: hexane

**Figure S23.** ^13^C NMR (100 MHz) spectrum of **3e** in CDCl_3_


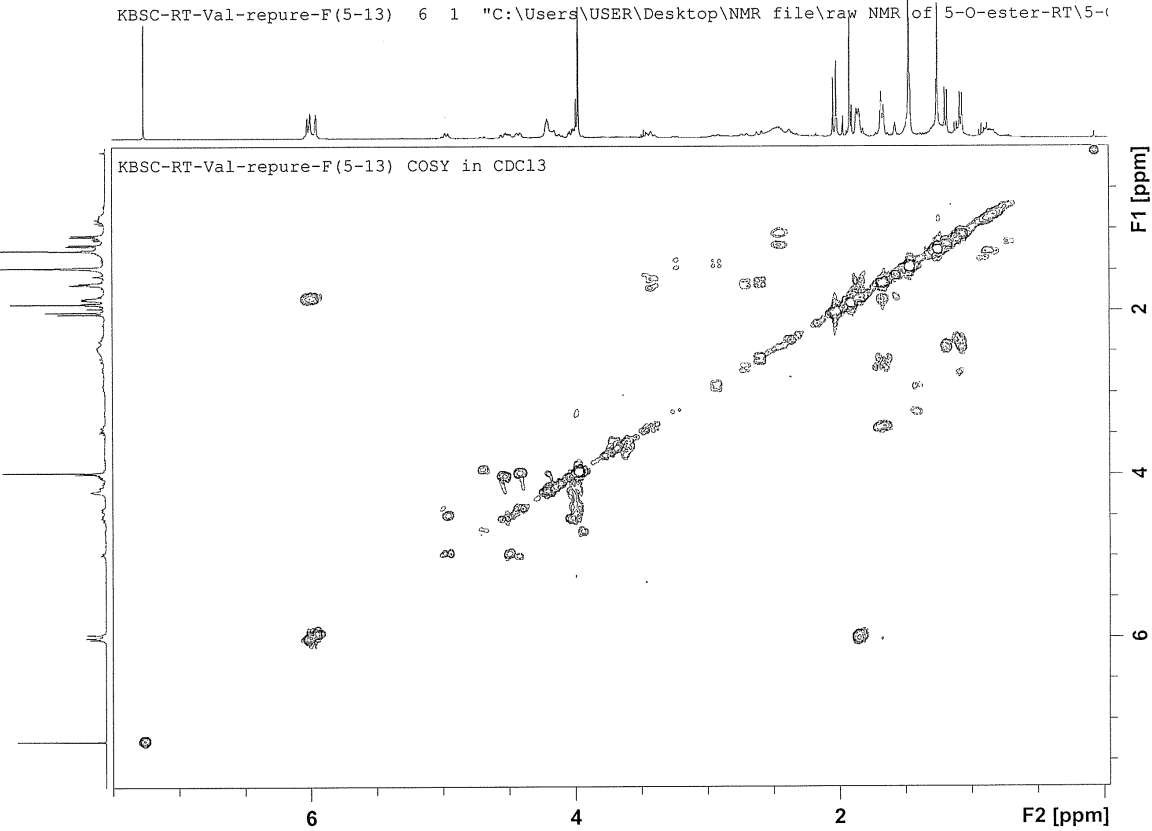


**Figure S24.** COSY (400 MHz) spectrum of **3e** in CDCl_3_


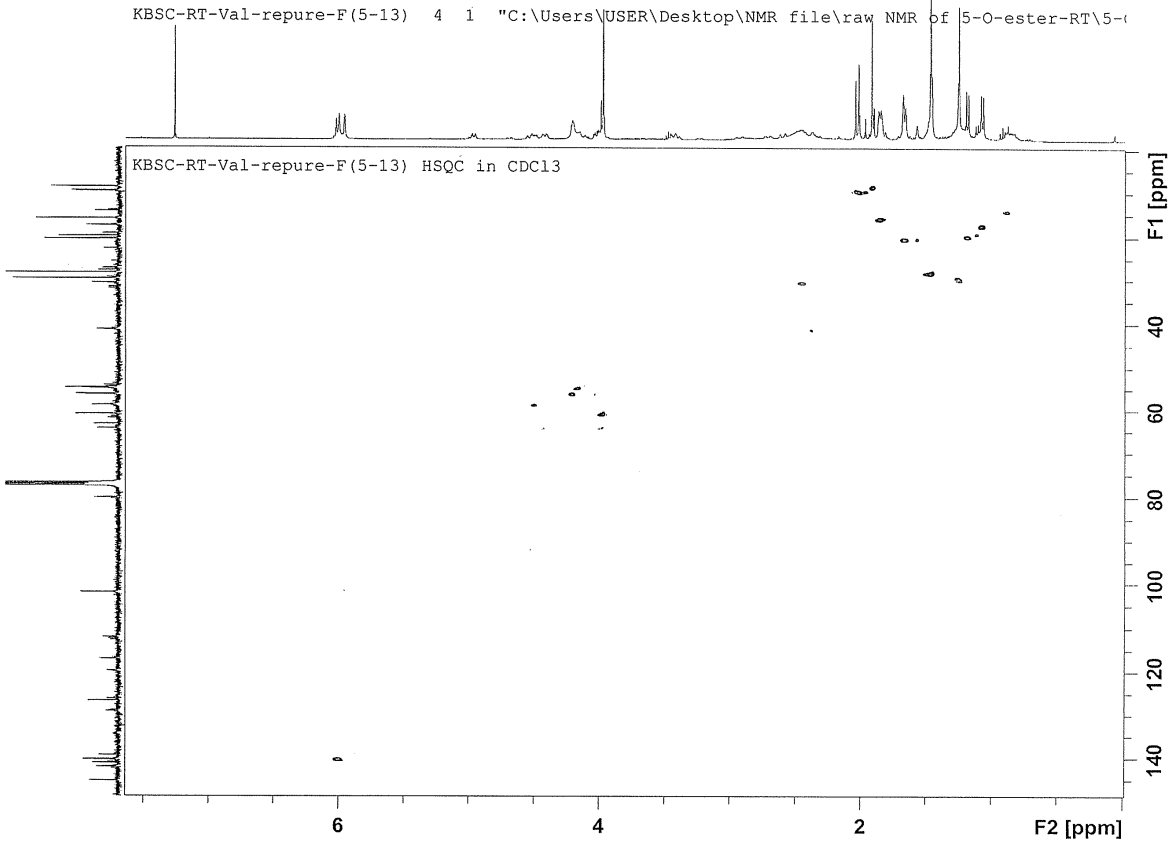


**Figure S25.** HSQC (400 MHz) spectrum of **3e** in CDCl_3_


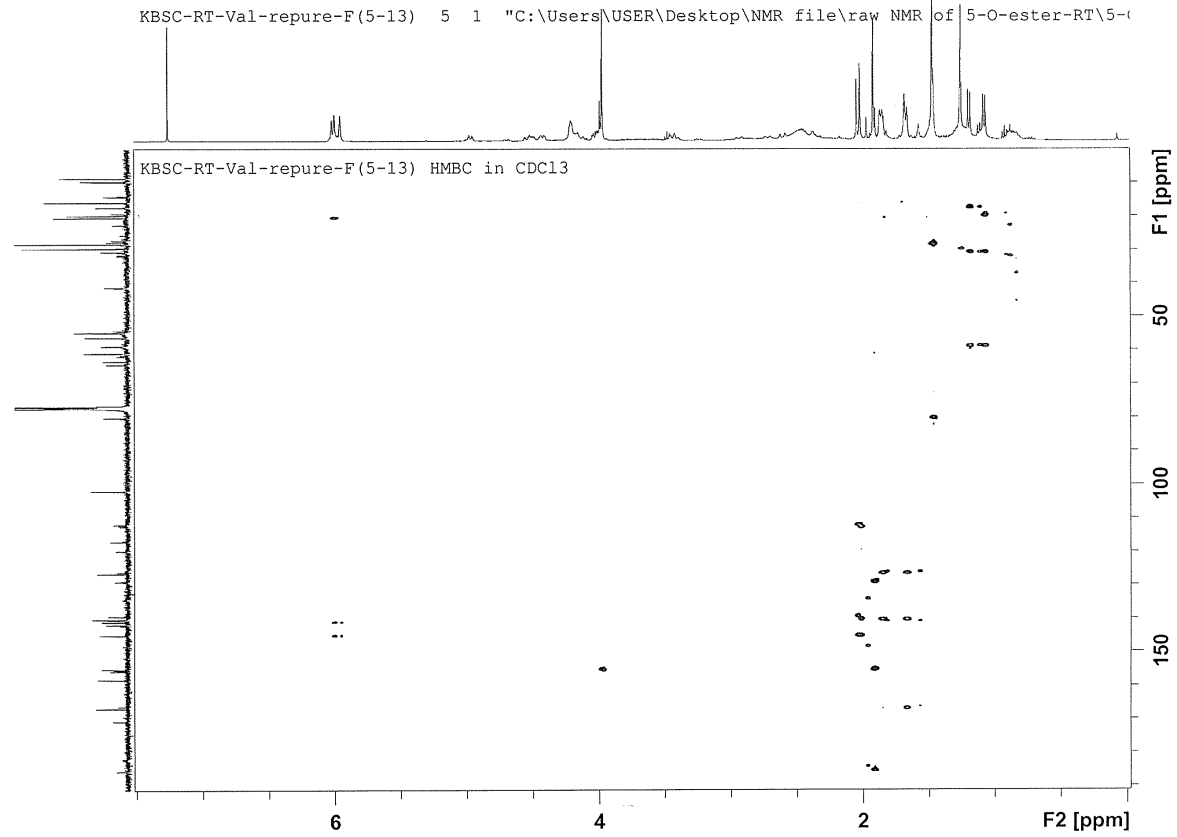


**Figure S26.** HMBC (400 MHz) spectrum of **3e** in CDCl_3_


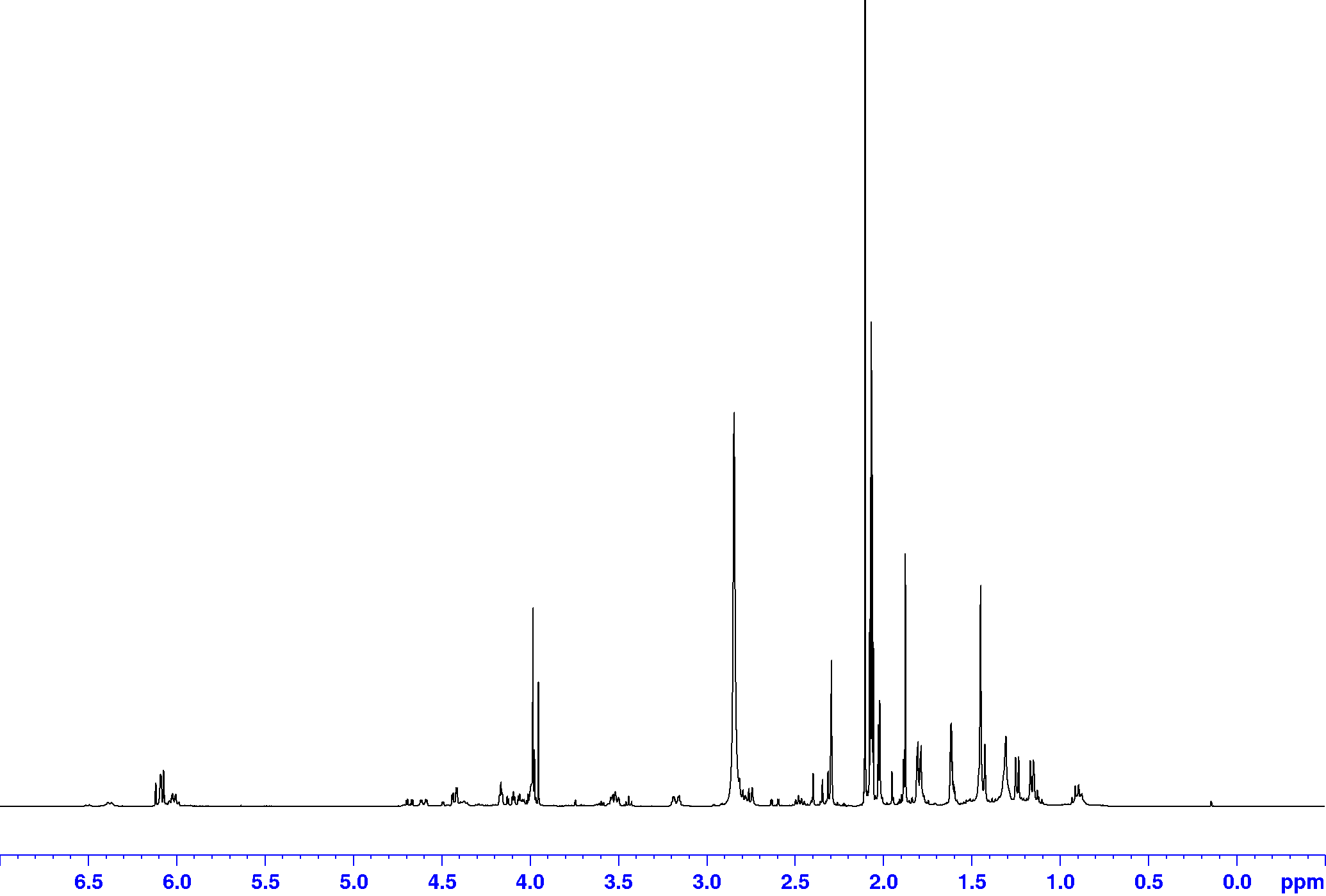


g

g

g: grease

**Figure S27.** ^1^H NMR (400 MHz) spectrum of **3e** in (CD_3_)_2_CO

^1^H NMR ((CD_3_)_2_CO, 400 MHz) δ 6.10 (1H, dd, *J* = 12.9, 1.0 Hz, OCH_2_O), 6.08 (1H, dd, *J* = 7.7, 1.0 Hz, OCH_2_O), 6.01 (1H, qq, *J* = 7.2, 1.5 Hz, 26-H), 4.68 (1H, dd, *J* = 11.5, 3.2 Hz, 3'-NH), 4.60 (1H, dd, *J* = 11.5, 3.2 Hz, 2'-H), 4.43 (1H, dd, *J* = 9.2, 2.4 Hz, 22-H_α_), 4.16 (1H, t, *J* = 3.1 Hz, 1-H), 4.11 (1H, overlapped, 11-H), 4.09 (1H, overlapped, 21-H), 4.02 (1H, overlapped, 22-H_β_), 3.98 (3H, s, 17-OCH_3_), 3.52 (1H, overlapped, 13-H), 3.17 (1H, ddd, *J* = 12.2, 4.7, 2.4 Hz, 3-H), 2.77 (1H, dd, *J* = 21.0, 7.5 Hz, 14-H_α_), 2.61 (1H, dd, *J* = 15.2, 2.4 Hz, 4-H_α_), 2.47 (1H, m, 8'-H), 2.37 (1H, d, *J* = 21.0 Hz, 14-H_β_), 2.29 (3H, s, NCH_3_), 2.02 (3H, s, 6-CH_3_), 1.88 (3H, s, 16-CH_3_), 1.80 (3H, dq, *J* = 7.2, 1.5 Hz, 27-CH_3_), 1.61 (3H, dq, *J* = 7.2, 1.5 Hz, 28-CH_3_), 1.60 (1H, overlapped, 4-H_β_), 1.45 (9H, s, 3 × 7'-CH_3_), 1.24 (3H, d, *J* = 6.8 Hz, 9'-CH_3_), 1.16 (3H, d, *J* = 6.8 Hz, 10'-CH_3_).

# Physical and spectroscopic data of **3f**

*5-O-(N-(tert-butoxycarbonyl)-L-phenylalaninoyl) ester derivative of renieramycin T,* **3f**: The title compound was synthesized from **2** (26.7 mg, 0.05 mmol), DMAP (14.2 mg, 0.12 mmol), EDCI (21.9 mg, 0.12 mmol) and *N-*Boc*-L-*Phenylalanine (61.5 mg, 0.23 mmol) to afford **3f**; yield 44% (brsm); yellow amorphous powder; $[]_{D}^{25}$ +11.4 (*c* 0.80, CHCl_3_); ECD Δ*ε* (*c* 34.03 μM, methanol, 20 ^o^C) −6.4 (347), −2.5 (296), +18.3 (265), −1.0 (228), +14.7 (211), −6.7 (208), −15.6 (203), +13.6 (200), −6.1 (196), −31.6 (194), +11.9 (192) nm; IR (ATR) ν_max_ 3423 (br), 2924, 2852, 1638, 1456, 1233, 1153 cm^−1^; ^1^H NMR (CDCl_3_, 400 MHz) δ 7.35 (4H, m, 10'-H, 11'-H), 7.29 (1H, m, 12'-H), 6.00 (1H, overlapped, 26-H), 5.97 (2H, dd, *J* = 21.8, 1.3 Hz, OCH_2_O), 4.88 (1H, d, *J* = 7.9 Hz, 3'-NH), 4.80 (1H, td, *J* = 9.3, 4.2 Hz, 2'-H), 4.52 (1H, dd, *J* = 11.2, 2.4 Hz, 22-H_α_), 4.16 (1H, t, *J* = 3.6 Hz, 1-H), 4.11 (1H, d, *J* = 2.0 Hz, 21-H), 4.02 (1H, dd, *J* = 11.2, 3.6 Hz, 22-H_β_), 3.96 (1H, d, *J* = 2.4 Hz, 11-H), 3.88 (3H, s, 17-OCH_3_), 3.55 (1H, dd, *J* = 14.4, 4.4 Hz, 8'-H_α_), 3.37 (1H, d, *J* = 7.6 Hz, 13-H), 3.23 (1H, dt, *J* = 12.2, 2.4 Hz, 3-H), 3.07 (1H, dd, *J* = 14.4, 10.0 Hz, 8'-H_β_), 2.74 (1H, dd, *J* = 20.8, 7.6 Hz, 14-H_α_), 2.62 (1H, dd, *J* = 14.6, 2.4 Hz, 4-H_α_), 2.32 (1H, d, *J* = 20.8 Hz, 14-H_β_), 2.26 (3H, s, NCH_3_), 2.02 (3H, s, 6-CH_3_), 1.92 (3H, s, 16-CH_3_), 1.86 (3H, dq, *J* = 7.6, 1.6 Hz, 27-CH_3_), 1.61 (3H, dq, *J* = 7.6, 1.4 Hz, 28-CH_3_),1.58 (1H, overlapped, 4-H_β_), 1.38 (9H, br s, 3 × 7'-CH_3_); ^13^C NMR (CDCl_3_, 100 MHz) δ 186.0 (C-15), 182.7 (C-18), 170.6 (C-1'), 167.0 (C-24), 158.3 (C-4'), 155.2 (C-17), 144.9 (C-7), 141.9 (C-20), 140.9 (C-8), 140.2 (C-26), 139.9 (C-5), 136.2 (C-9'), 135.3 (C-19), 129.3 (2 × C-11'), 129.1 (C-16), 128.7 (2 × C-10'), 127.1 (C-12'), 126.7 (C-25), 119.9 (C-6), 117.3 (21-CN), 112.5 (C-10), 112.1 (C-9), 101.8 (OCH_2_O), 80.1 (C-6'), 63.6 (C-22), 60.8 (17-OCH_3_), 59.2 (C-21), 56.4 (C-1), 55.6 (C-3), 54.8 (C-11), 54.7 (C-13), 54.6 (C-2'), 41.4 (NCH_3_), 38.2 (C-8'), 28.2 (3 × 7'-CH_3_), 27.7 (C-4), 21.1 (C-14), 20.5 (28-CH_3_), 15.9 (27-CH_3_), 9.5 (6-CH_3_), 8.6 (16-CH_3_); HRESIMS *m/z* 823.3547 ([M+H]^+^, calculated for C_45_H_51_N_4_O_11_, 823.3549).


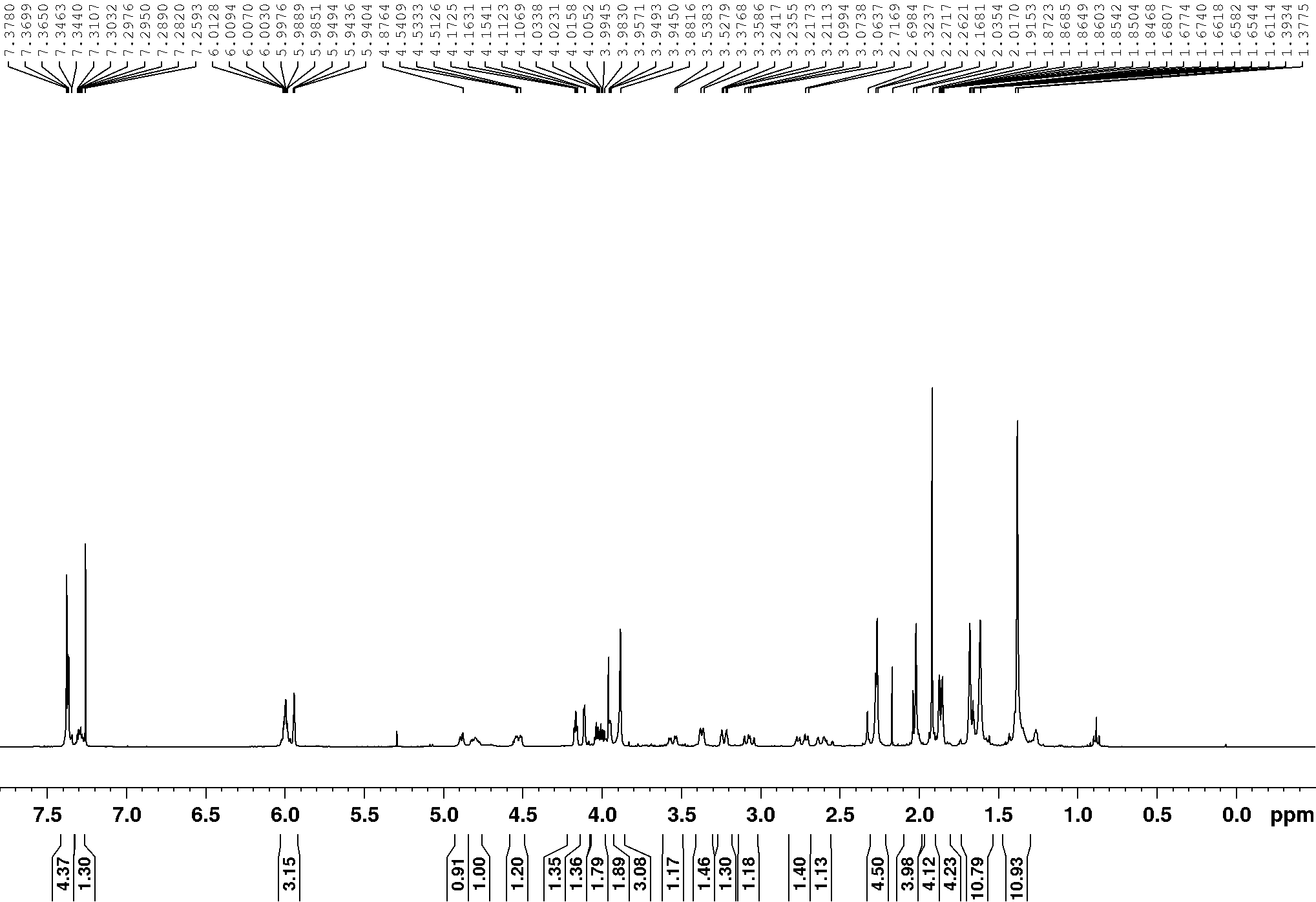


dichloromethane

g, h

g, h

g: grease, h: hexane

**Figure S28.** ^1^H NMR (400 MHz) spectrum of **3f** in CDCl_3_


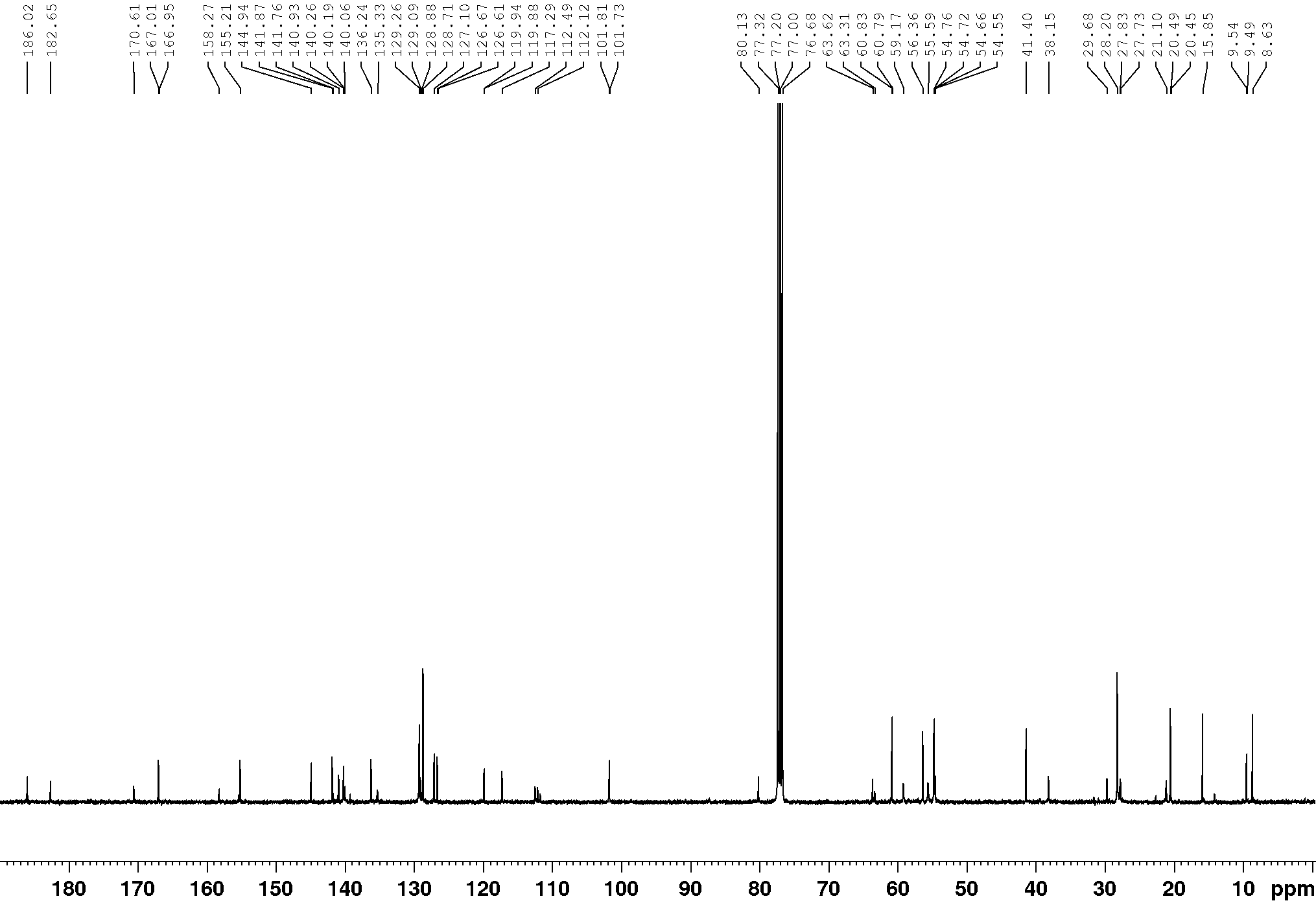


g

h

h

h

g: grease, h: hexane

**Figure S29.** ^13^C NMR (100 MHz) spectrum of **3f** in CDCl_3_

**
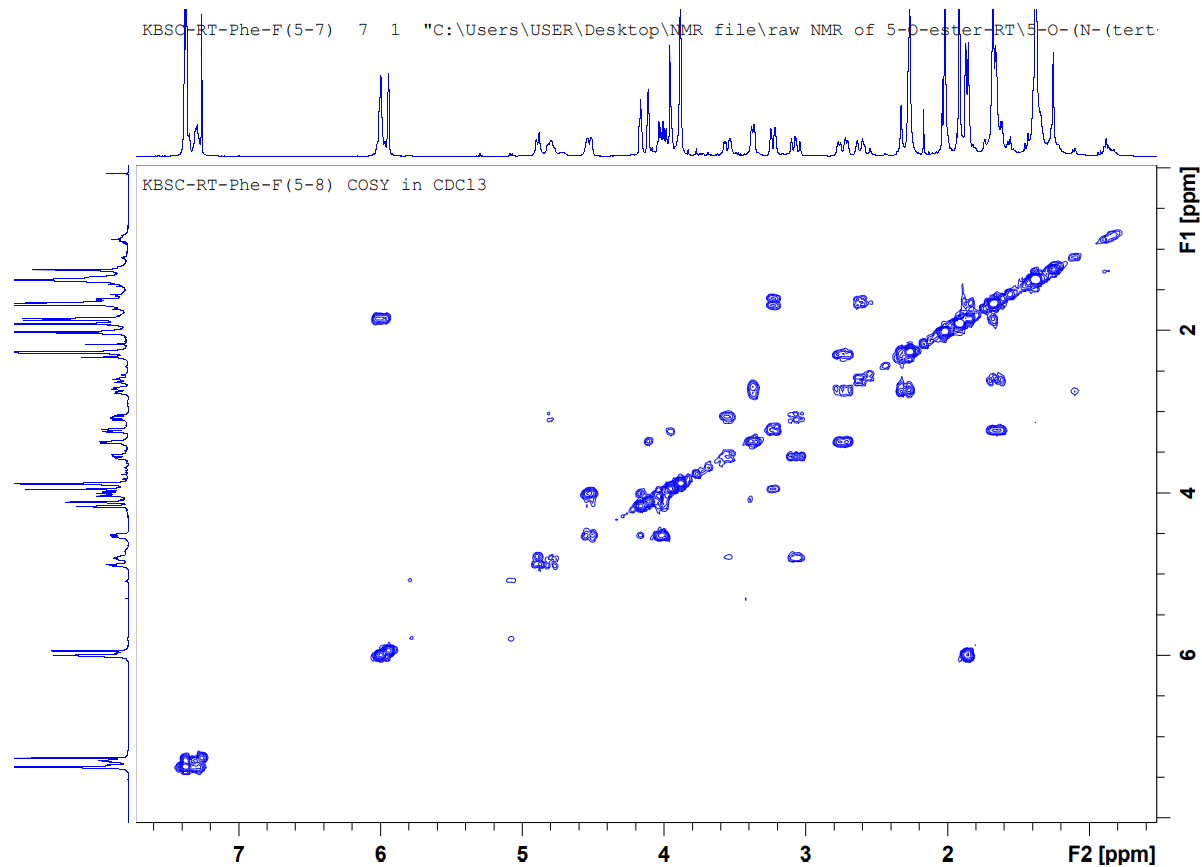
**

**Figure S30.** COSY (400 MHz) spectrum of **3f** in CDCl_3_


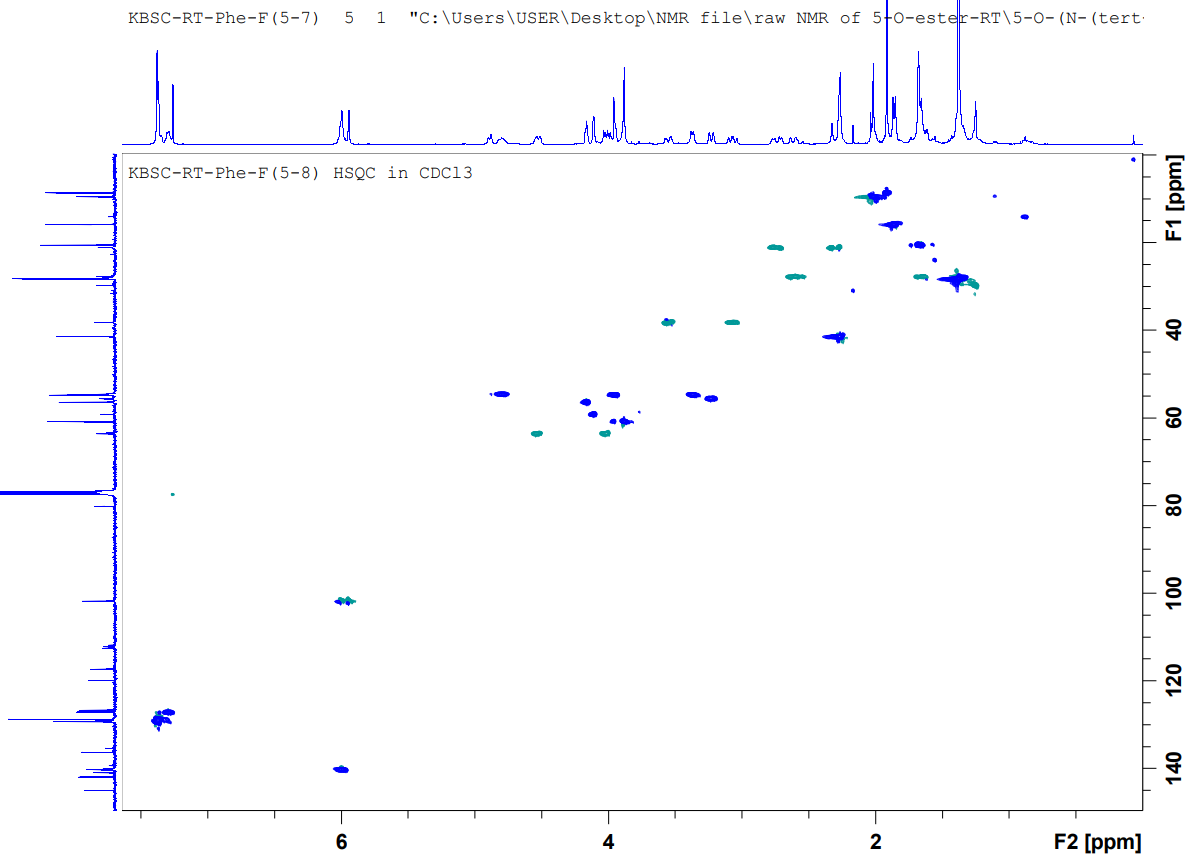


**Figure S31.** HSQC (400 MHz) spectrum of **3f** in CDCl_3_


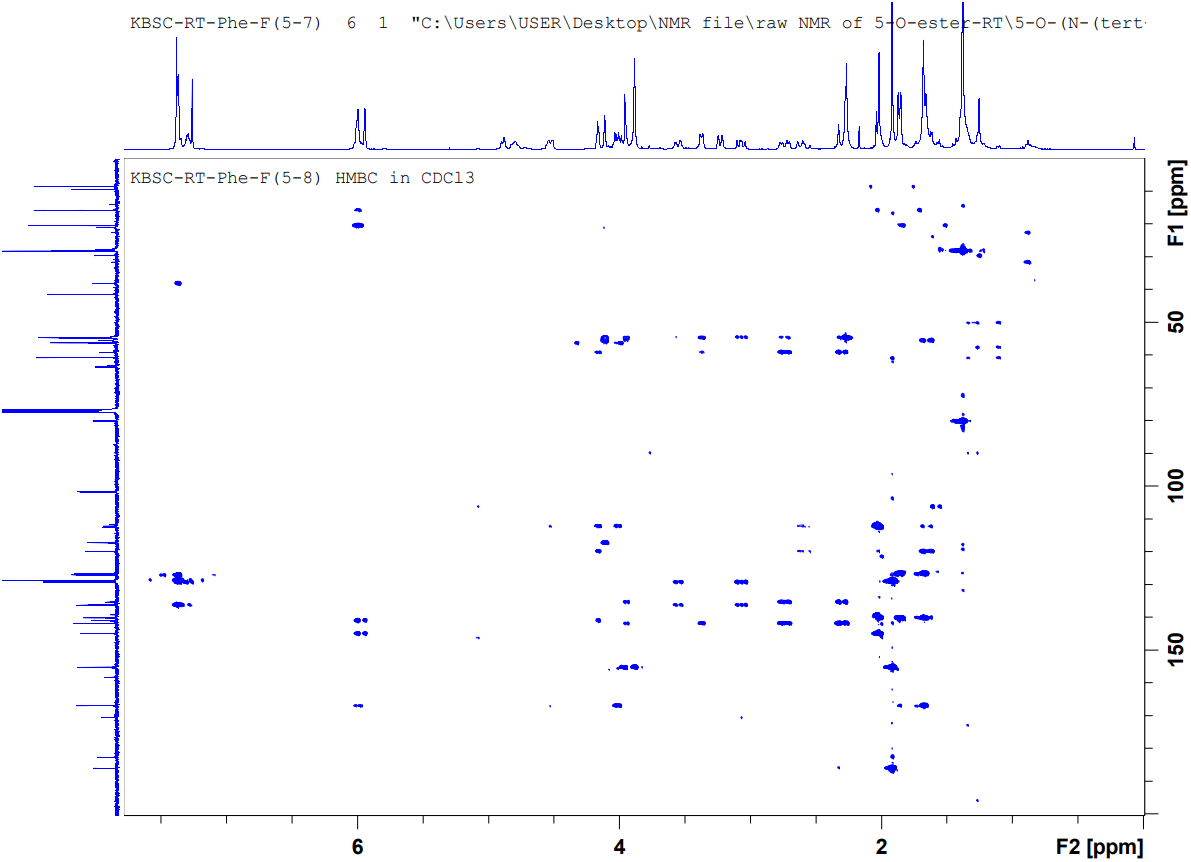


**Figure S32.** HMBC (400 MHz) spectrum of **3f** in CDCl_3_

# Physical and spectroscopic data of **3g**

*5-O-(2-pyridinecarbonyl) ester derivative of renieramycin T,* **3g**: The title compound was synthesized from **2** (25.7 mg, 0.04 mmol), DMAP (13.6 mg, 0.11 mmol), EDCI (21.8 mg, 0.11 mmol) and pyridine-2-carbonyl chloride hydrochloride (39.7 mg, 0.22 mmol) to afford **3g**; yield 31% (brsm); yellow amorphous powder; $[]_{D}^{25}$−2.0 (*c* 0.16, CHCl_3_); ECD Δ*ε* (*c* 73.45 μM, methanol, 20 ^o^C) +14.5 (260), −8.5 (222), +18.8 (209), −18.8 (205), +17.2 (203), −15.0 (200), +4.5 (197.3), −23.3 (195), +5.8 (193) nm; IR (ATR) ν_max_ 2923, 1713, 1650, 1435, 1301, 1232, 1109, 1087, 953, 747 cm^−1^; ^1^H NMR (CDCl_3_, 400 MHz) δ 8.88 (1H, dd, *J* = 4.8, 0.8 Hz, 6'-H), 8.25 (1H, d, *J* = 7.6 Hz, 3'-H), 7.97 (1H, td, *J* = 7.6, 0.8 Hz, 4'-H), 7.62 (1H, ddd, *J* = 7.6, 4.8, 0.8 Hz, 5'-H), 6.01 (2H, dd, *J* = 26.0, 1.2 Hz, OCH_2_O), 5.99 (1H, overlapped, 26-H), 4.66 (1H, dd, *J* = 11.6, 2.8 Hz, 22-H_α_), 4.21 (1H, br t, *J* = 3.6 Hz, 1-H), 4.19 (1H, d, *J* = 2.4 Hz, 21-H), 4.07 (1H, dd, *J* = 11.6, 3.6 Hz, 22-H_β_), 4.02 (1H, overlapped, 11-H), 3.70 (3H, s, 17-OCH_3_), 3.46 (1H, overlapped, 13-H), 3.30 (1H, br d, *J* = 11.6 Hz, 3-H), 2.73 (1H, br s, 14-H_α_), 2.64 (1H, d, *J* = 14.8 Hz, 4-H_α_), 2.40 (1H, overlapped, 14-H_β_), 2.32 (3H, overlapped, NCH_3_), 2.08 (3H, s, 6-CH_3_), 1.87 (3H, s, 16-CH_3_), 1.86 (3H, dq, *J* = 7.2, 1.4 Hz, 27-CH_3_), 1.70 (1H, overlapped, 4-H_β_), 1.67 (3H, dq, *J* = 1.4, 1.4 Hz, 28-CH_3_); ^13^C NMR (CDCl_3_, 100 MHz) δ 185.6 (C-15), 182.2 (C-18), 167.0 (C-24), 162.9 (C-1'), 155.4 (C-17), 150.3 (C-6'), 146.7 (C-2'), 145.1 (C-7), 141.4 (C-20), 140.9 (C-8), 140.5 (C-26), 140.5 (C-5), 137.3 (C-4'), 135.0 (C-19), 128.5 (C-16), 127.7 (C-5'), 126.6 (C-25), 125.7 (C-3'), 119.9 (C-6), 117.1 (21-CN), 112.2 (C-10), 112.2 (C-9), 101.8 (OCH_2_O), 62.6 (C-22), 60.5 (17-OCH_3_), 58.4 (C-21), 56.6 (C-1), 54.8 (C-3), 54.5 (C-11), 54.5 (C-13), 41.2 (NCH_3_), 27.9 (C-4), 21.3 (C-14), 20.6 (28-CH_3_), 15.9 (27-CH_3_), 9.5 (6-CH_3_), 8.5 (16-CH_3_); HRESIMS *m/z* 681.2555 ([M+H]^+^, calculated for C_37_H_37_N_4_O_9_, 681.2555).


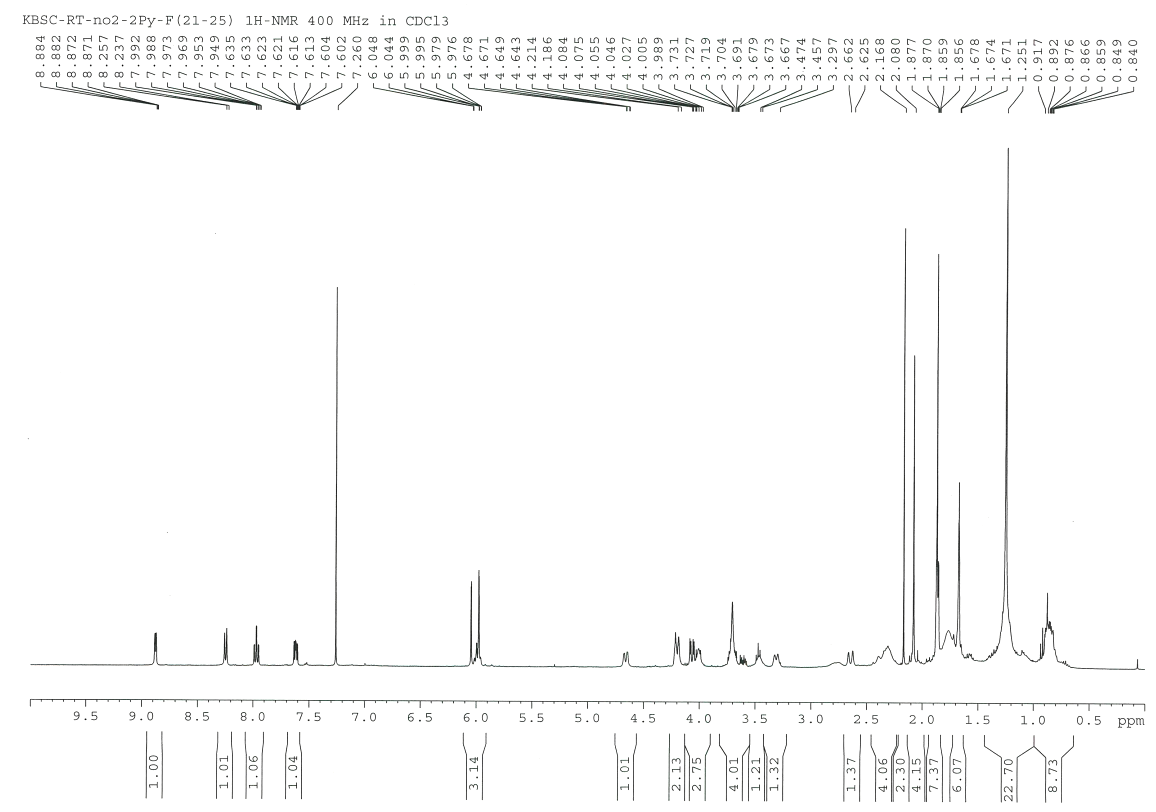


acetone

g, h

g: grease, h: hexane

g, h

**Figure S33.** ^1^H NMR (400 MHz) spectrum of **3g** in CDCl_3_


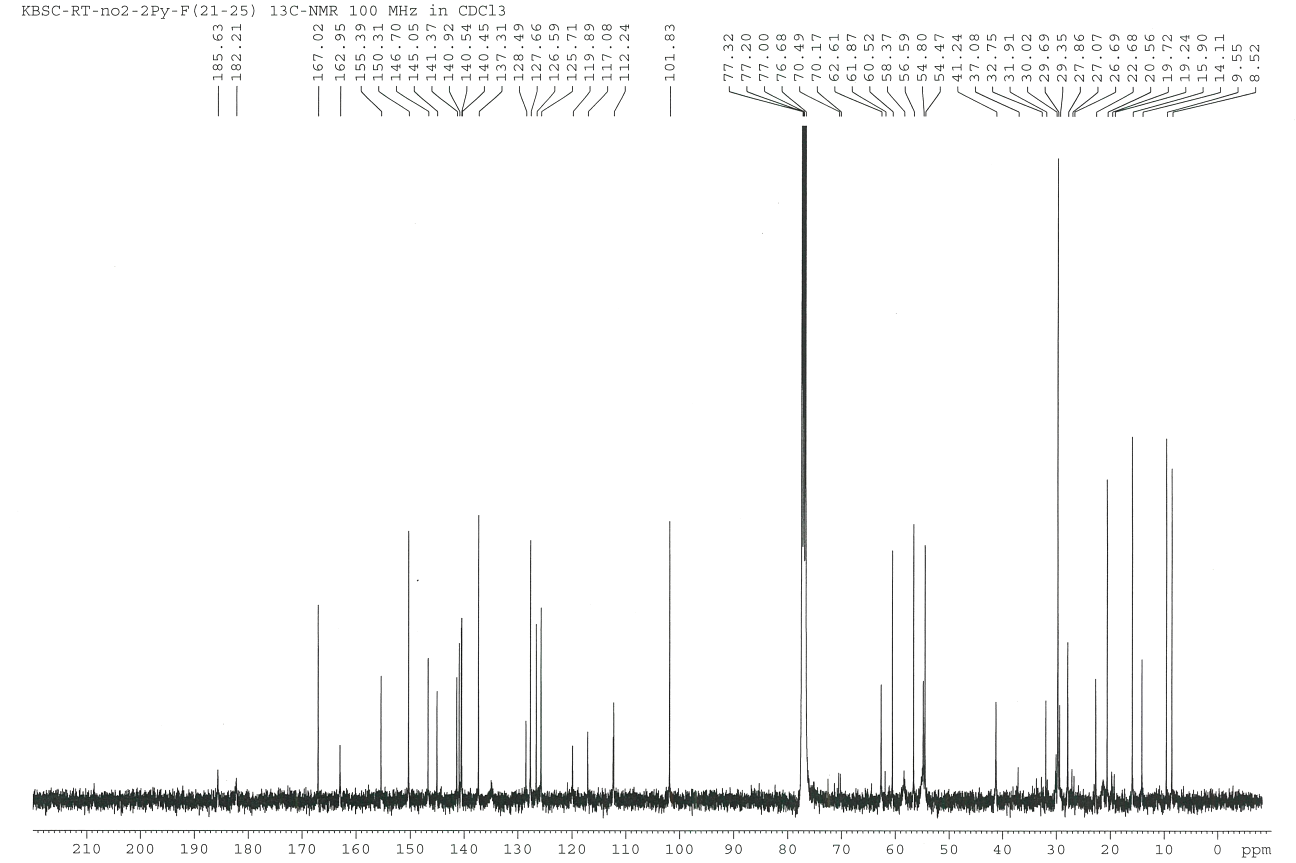


g

h

h

acetone

acetone

h

g: grease, h: hexane

**Figure S34.** ^13^C NMR (100 MHz) spectrum of **3g** in CDCl_3_


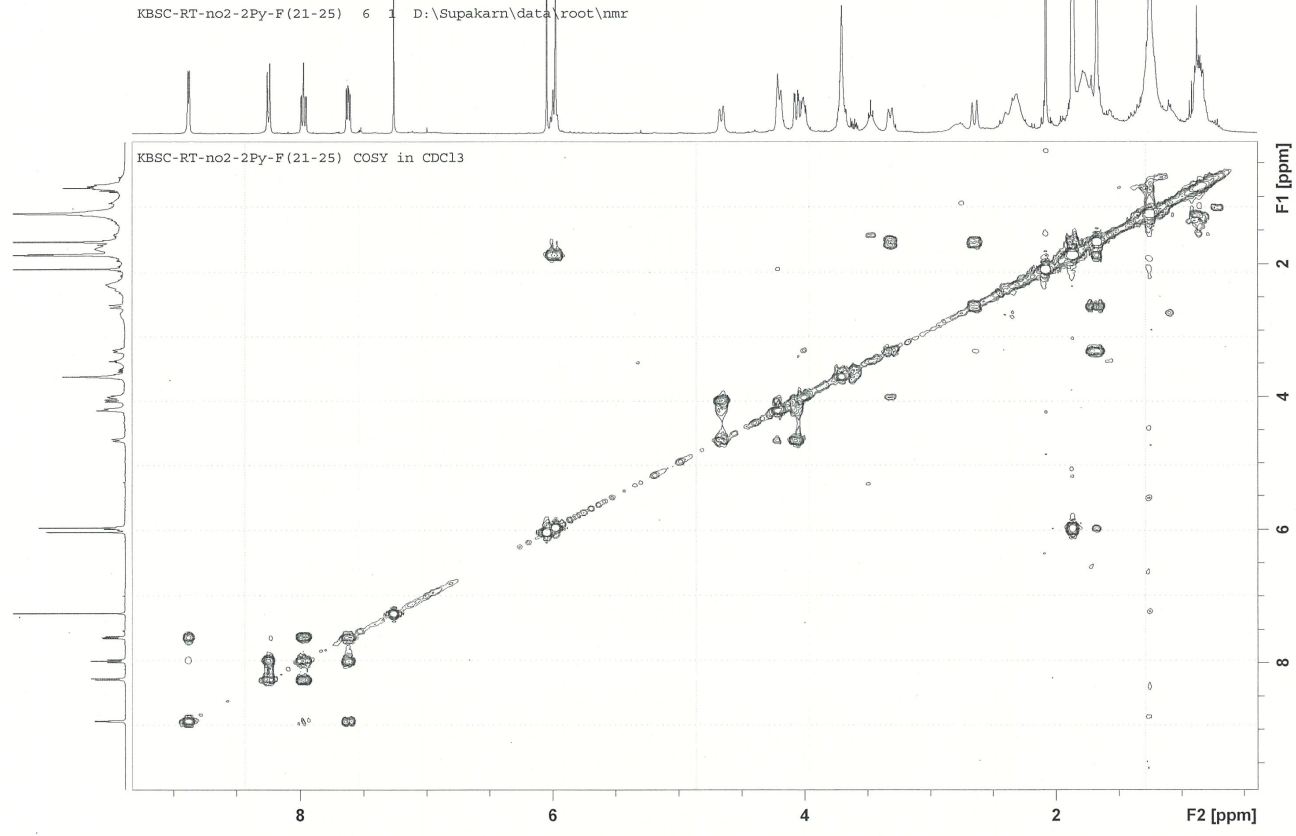


**Figure S35.** COSY (400 MHz) spectrum of **3g** in CDCl_3_


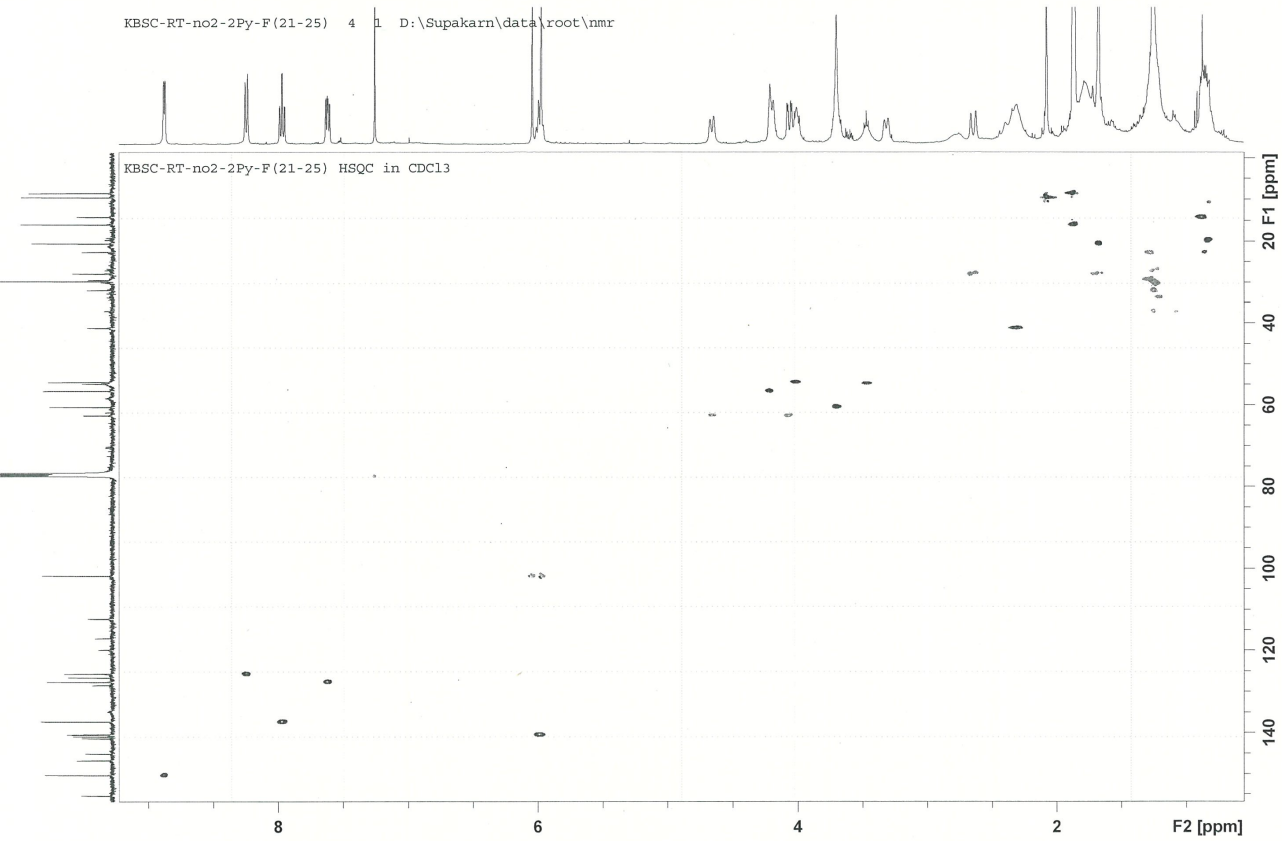


**Figure S36.** HSQC (400 MHz) spectrum of **3g** in CDCl_3_


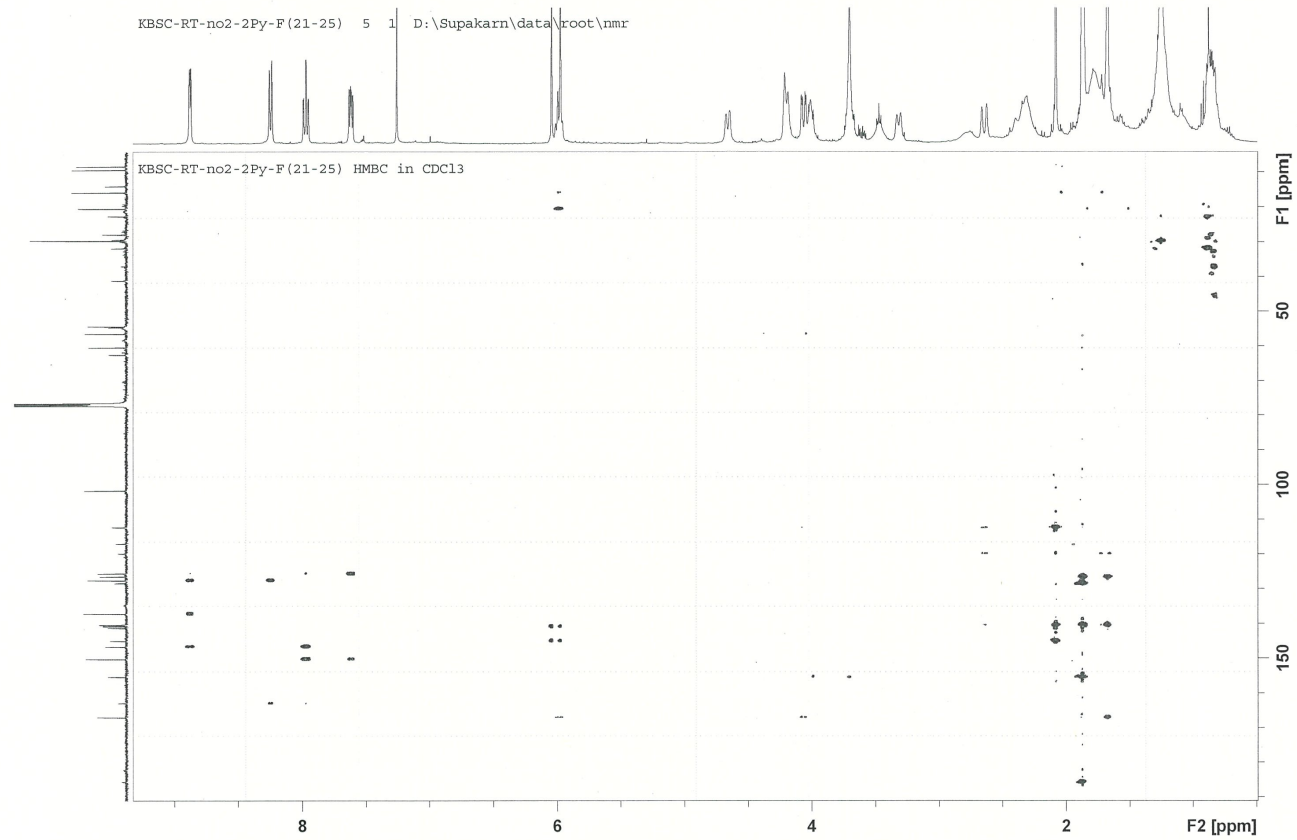


**Figure S37.** HMBC (400 MHz) spectrum of **3g** in CDCl_3_

# Physical and spectroscopic data of **3h**

*5-O-(4-pyridinecarbonyl) ester derivative of renieramycin T,* **3h**: The title compound was synthesized from **2** (40.4 mg, 0.07 mmol), DMAP (10.3 mg, 0.08 mmol), EDCI (15.9 mg, 0.08 mmol) and isonicotinoyl chloride (62.5 mg, 0.35 mmol) to afford **3h**; yield 77% (brsm); yellow amorphous powder; $[]_{D}^{25}$ +6.1 (*c* 0.56, CHCl_3_); ECD Δ*ε* (*c* 15.28 μM, methanol, 20 ^o^C) −3.0 (302), −13.0 (290), +9.4 (278), +50.7 (264), +0.5 (236), −59.7 (224), −84.5 (219), +41.3 (210), −50.9 (205), +47.6 (203) nm; IR (ATR) ν_max_ 3446 (br), 2929, 1747, 1709, 1652, 1455, 1234, 1150, 1108, 1026, 755 cm^−1^; ^1^H NMR (CDCl_3_, 400 MHz) δ 8.94 (2H, d, *J* = 5.6 Hz, 4'-H), 8.06 (2H, d, *J* = 5.6 Hz, 3'-H), 6.02 (2H, d, *J* = 26.8 Hz, OCH_2_O), 6.01 (1H, overlapped, 26-H), 4.61 (1H, dd, *J* = 11.6, 2.8 Hz, 22-H_α_), 4.20 (1H, br t, *J* = 4.0 Hz, 1-H), 4.14 (1H, d, *J* = 2.4 Hz, 21-H), 4.05 (1H, dd, *J* = 11.6, 4.0 Hz, 22-H_β_), 3.92 (1H, d, *J* = 2.4 Hz, 11-H), 3.73 (3H, s, 17-OCH_3_), 3.38 (1H, dd, *J* = 7.2, 2.4 Hz, 13-H), 3.26 (1H, dt, *J* = 12.0, 2.4 Hz, 3-H), 2.73 (1H, dd, *J* = 20.8, 7.2 Hz, 14-H_α_), 2.53 (1H, dd, *J* = 15.2, 1.6 Hz, 4-H_α_), 2.33 (1H, d, *J* = 20.8 Hz, 14-H_β_), 2.25 (3H, s, NCH_3_), 2.05 (3H, s, 6-CH_3_), 1.89 (3H, s, 16-CH_3_), 1.88 (3H, dq, *J* = 7.2, 1.2 Hz, 27-CH_3_), 1.68 (3H, dq, *J* = 1.4, 1.2 Hz, 28-CH_3_), 1.61 (1H, overlapped, 4-H_β_); ^13^C NMR (CDCl_3_, 100 MHz) δ 185.8 (C-15), 182.6 (C-18), 167.0 (C-24), 162.7 (C-1'), 155.2 (C-17), 150.0 (2 × C-4'), 145.1 (C-7), 141.6 (C-20), 141.2 (C-8), 140.4 (C-26), 140.0 (C-5), 137.1 (C-2'), 135.2 (C-19), 128.8 (C-16), 126.6 (C-25), 123.5 (2 × C-3'), 120.1 (C-6), 117.2 (21-CN), 112.6 (C-10), 111.9 (C-9), 101.9 (OCH_2_O), 63.0 (C-22), 60.4 (17-OCH_3_), 58.9 (C-21), 56.5 (C-1), 55.3 (C-3), 54.7 (C-11), 54.5 (C-13), 41.4 (NCH_3_), 28.0 (C-4), 21.0 (C-14), 20.5 (28-CH_3_), 15.9 (27-CH_3_), 9.5 (6-CH_3_), 8.6 (16-CH_3_); HRESIMS *m/z* 681.2554 ([M+H]^+^, calculated for C_37_H_37_N_4_O_9_, 681.2555).

*
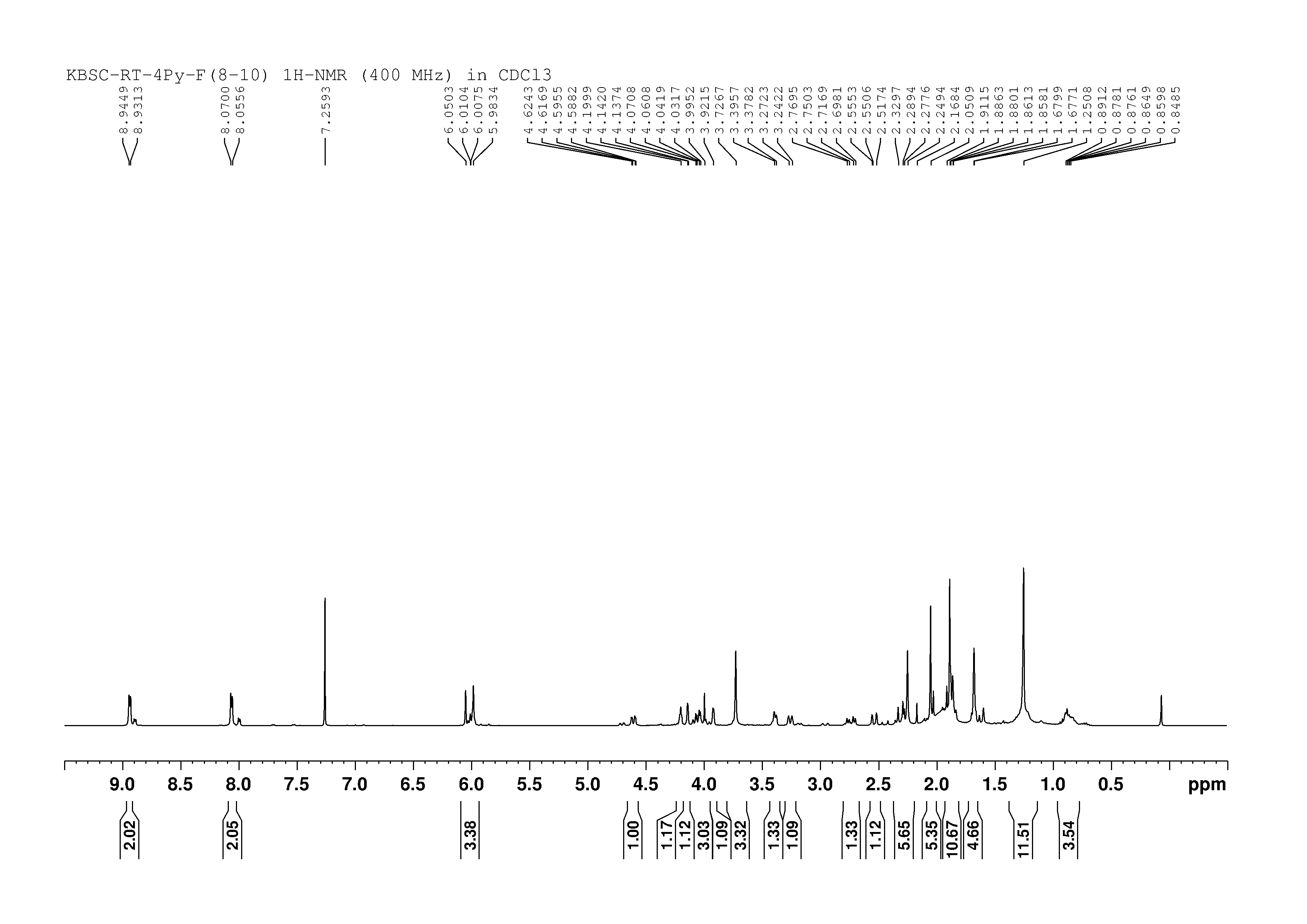
*

g, h

g, h

g: grease, h: hexane

**Figure S38.** ^1^H NMR (400 MHz) spectrum of **3h** in CDCl_3_


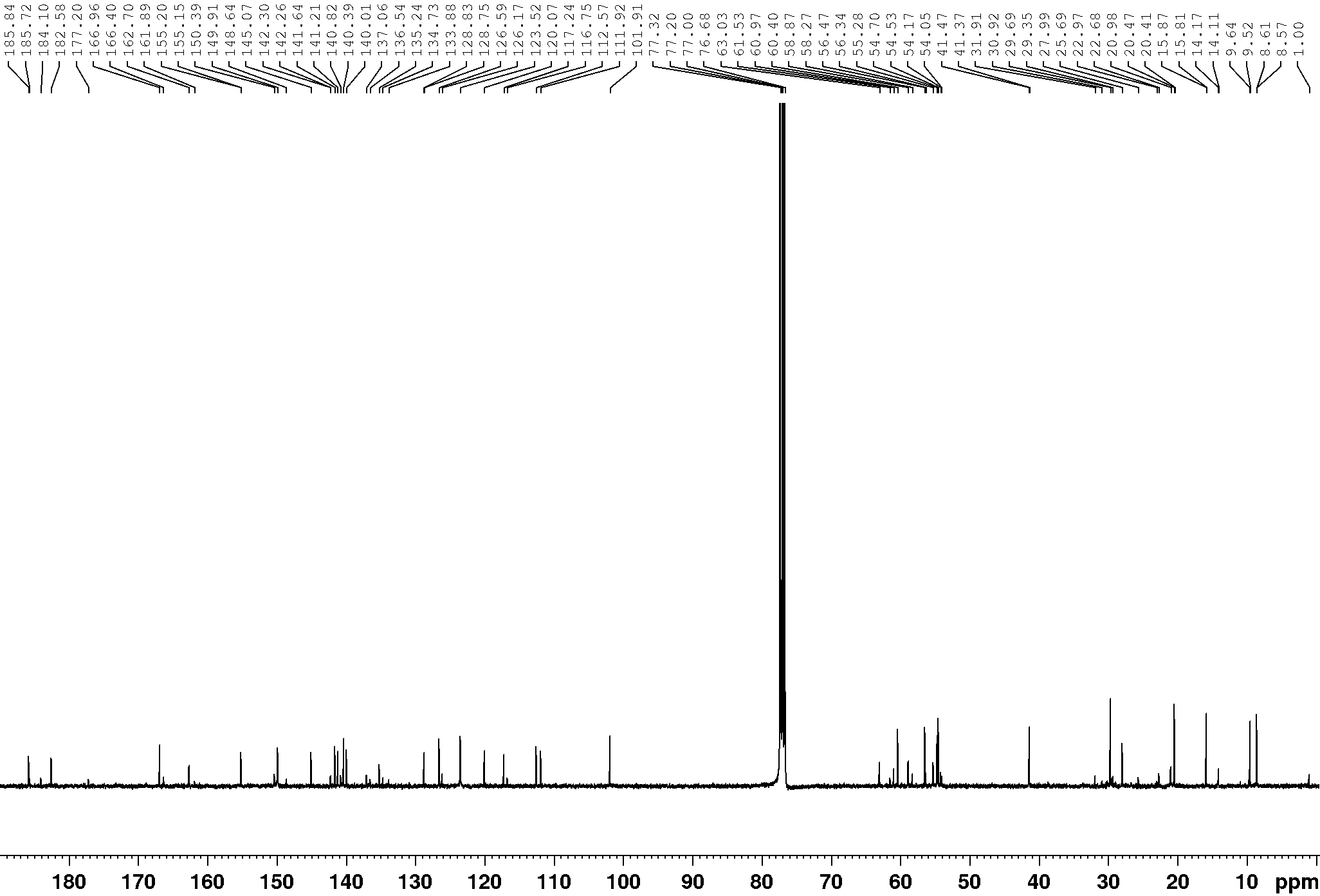


h

g

h

h

g: grease, h: hexane

**Figure S39.** ^13^C NMR (100 MHz) spectrum of **3h** in CDCl_3_


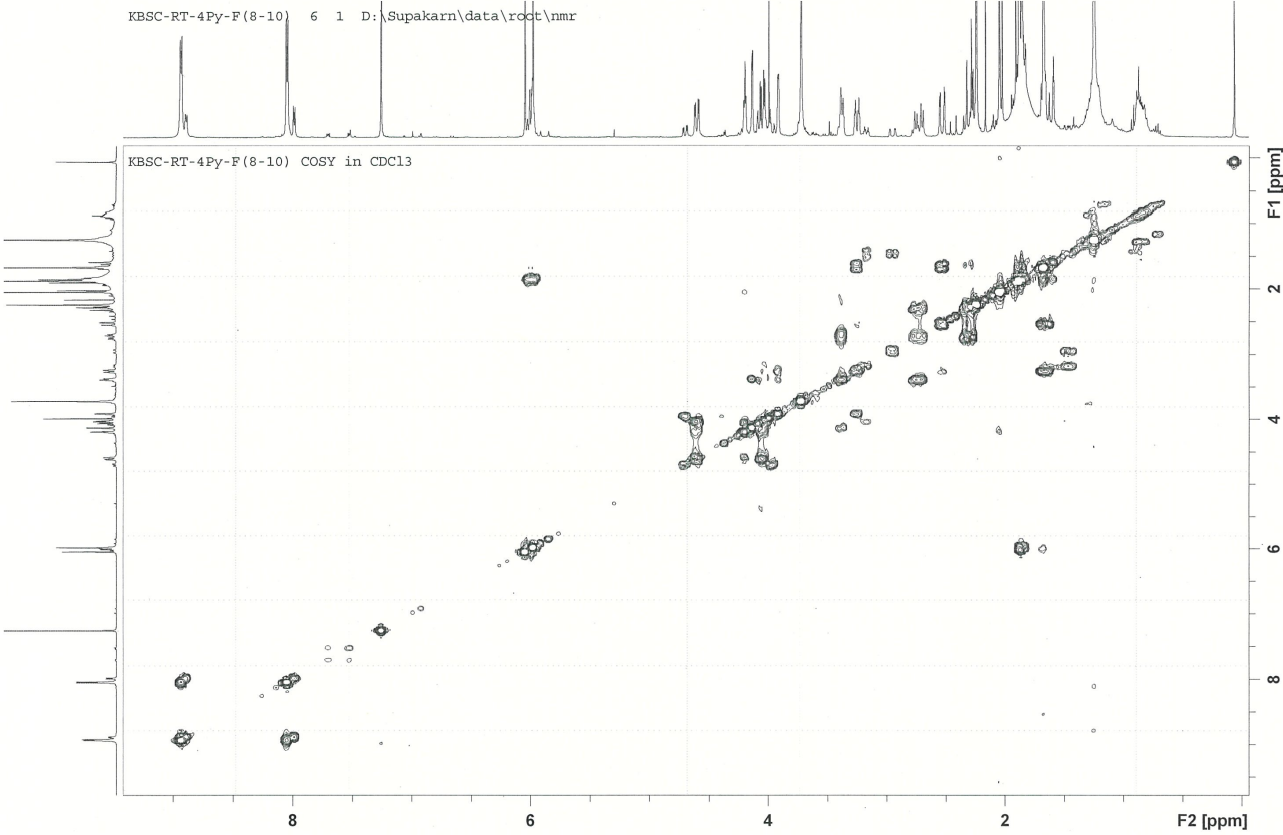


**Figure S40.** COSY (400 MHz) spectrum of **3h** in CDCl_3_


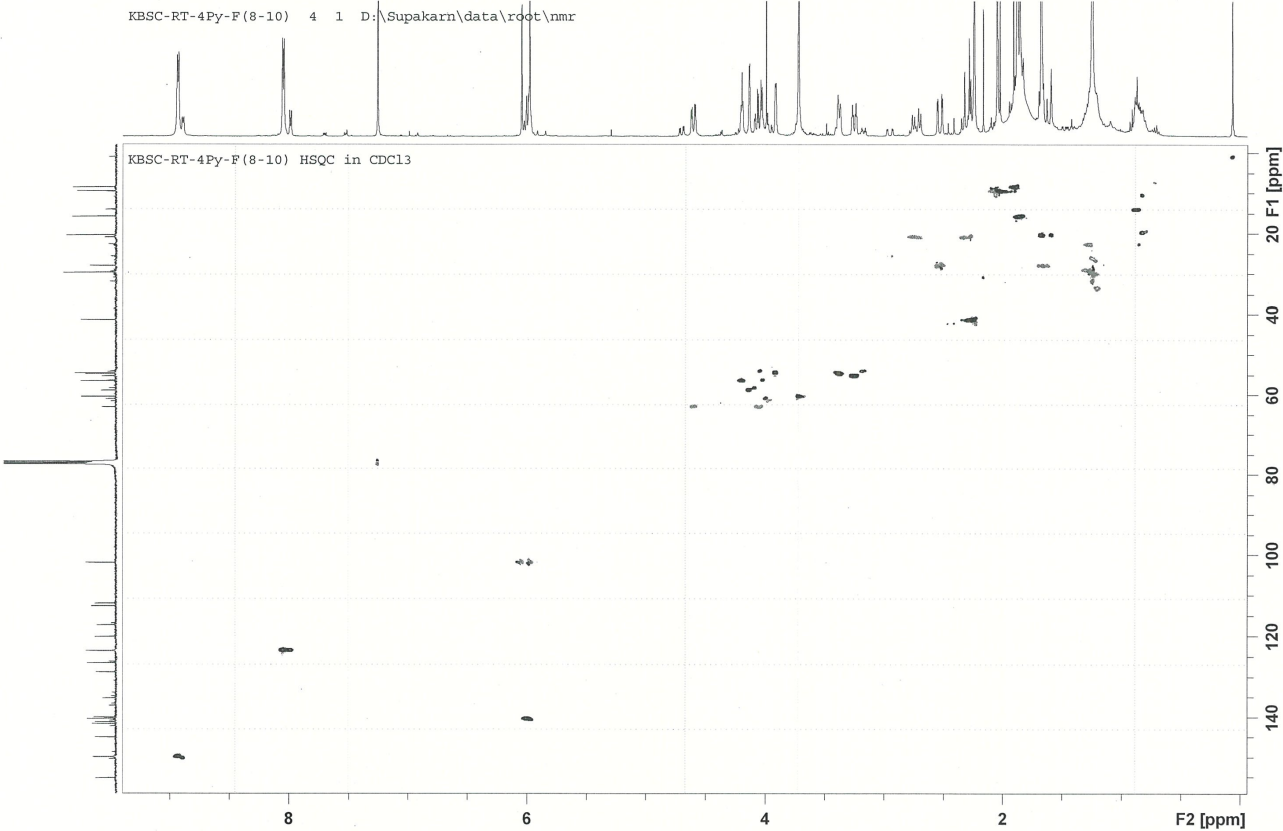


**Figure S41.** HSQC (400 MHz) spectrum of **3h** in CDCl_3_


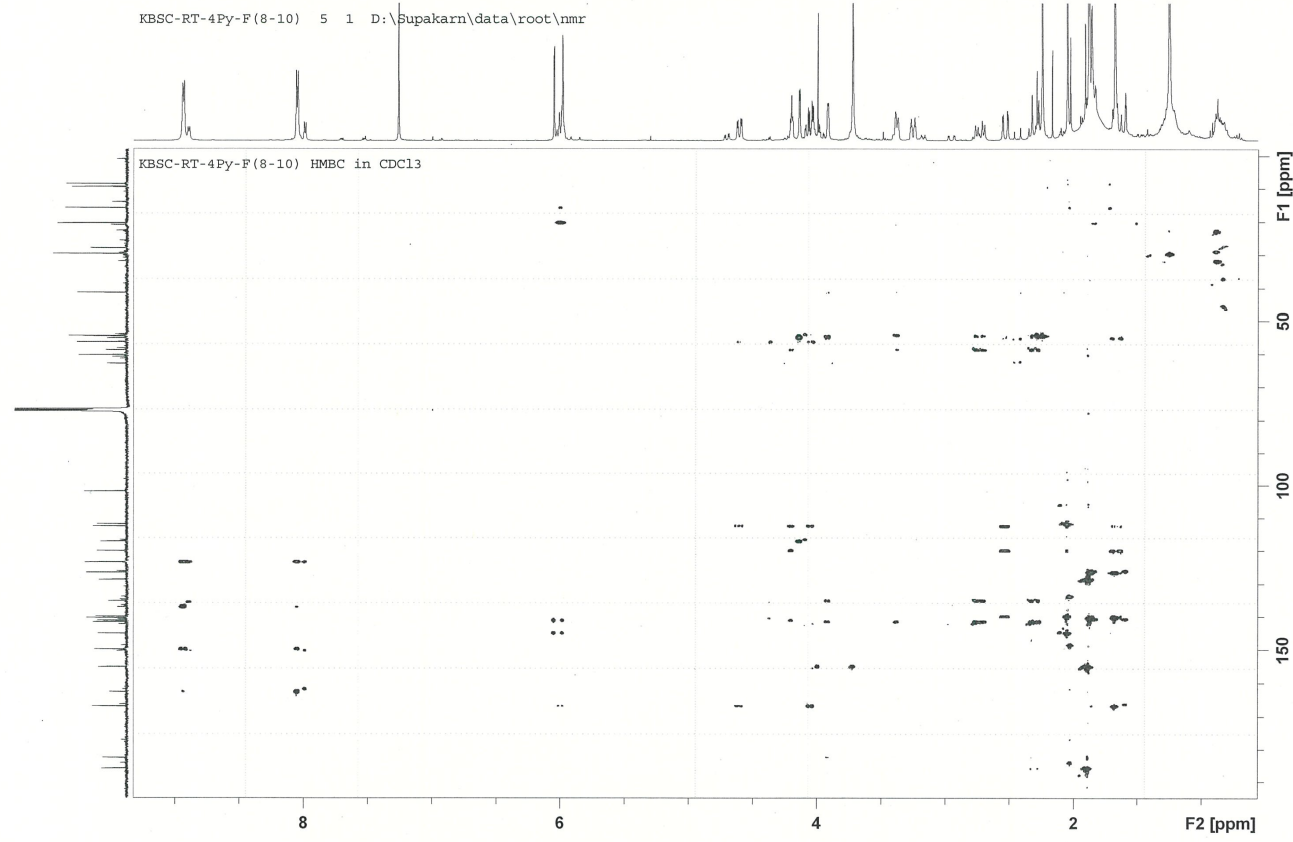


**Figure S42.** HMBC (400 MHz) spectrum of **3h** in CDCl_3_

# Physical and spectroscopic data of **3i**

*5-O-(2-quinolinecarbonyl) ester derivative of renieramycin T,* **3i**: The title compound was synthesized from **2** (25.0 mg, 0.04 mmol), DMAP (13.3 mg, 0.11 mmol), EDCI (20.5 mg, 0.11 mmol) and quinaldic acid (37.6 mg, 0.22 mmol) to afford **3i**; yield 55% (brsm); yellow amorphous powder; $[]_{D}^{25}$ −4.2 (*c* 0.46, CHCl_3_); ECD Δ*ε* (*c* 32.84 μM, methanol, 20 ^o^C) +0.4 (302), −1.1 (294), +15.5 (262), +23.1 (247), −15.5 (216), −24.4 (210), +18.6 (206), −20.4 (198), +13.0 (195), −4.6 (191) nm; IR (ATR) ν_max_ 2921, 2851, 1705, 1630, 1306, 1230, 1022, 950, 769 cm^−1^; ^1^H NMR (CDCl_3_, 400 MHz) δ 8.46 (1H, d, *J* = 8.5 Hz, 10'-H), 8.42 (1H, d, *J* = 8.7 Hz, 8'-H), 8.32 (1H, d, *J* = 8.5 Hz, 11'-H), 8.00 (1H, d, *J* = 8.7 Hz, 5'-H), 7.89 (1H, ddd, *J* = 8.7, 6.9, 1.4 Hz, 7'-H), 7.76 (1H, ddd, *J* = 8.7, 6.9, 1.4 Hz, 6'-H), 6.07 (2H, dd, *J* = 27.9, 1.3 Hz, OCH_2_O), 6.03 (1H, overlapped, 26-H), 4.69 (1H, br d, *J* = 11.6 Hz, 22-H_α_), 4.31 (1H, overlapped, 1-H), 4.30 (1H, overlapped, 21-H), 4.19 (1H, br s, 11-H), 4.11 (1H, dd, *J* = 11.6, 3.7 Hz, 22-H_β_), 3.69 (3H, s, 17-OCH_3_), 3.70 (1H, overlapped, 13-H), 3.53 (1H, overlapped, 3-H), 2.97 (1H, overlapped, 14-H_α_), 2.78 (1H, br d, *J* = 14.6 Hz, 4-H_α_), 2.56 (1H, overlapped, 14-H_β_), 2.49 (3H, s, NCH_3_), 2.15 (3H, s, 6-CH_3_), 1.90 (3H, dq, *J* = 7.3, 1.4 Hz, 27-CH_3_), 1.88 (3H, s, 16-CH_3_), 1.77 (1H, overlapped, 4-H_β_), 1.72 (3H, dq, *J* = 1.4, 1.4 Hz, 28-CH_3_); ^13^C NMR (CDCl_3_, 100 MHz) δ 185.1 (C-15), 181.5 (C-18), 167.1 (C-24), 163.1 (C-1'), 155.4 (C-17), 147.8 (C-4'), 146.5 (C-2'), 145.2 (C-7), 141.3 (C-20), 141.0 (C-8), 140.8 (C-5), 140.6 (C-26), 137.7 (C-10'), 133.3 (C-19), 130.8 (C-8'), 130.7 (C-7'), 129.6 (C-9'), 129.2 (C-6'), 128.6 (C-16), 127.7 (C-5'), 126.6 (C-25), 121.2 (C-11'), 119.5 (C-6), 116.7 (21-CN), 112.6 (C-10), 112.1 (C-9), 101.9 (OCH_2_O), 62.6 (C-22), 60.7 (17-OCH_3_), 56.6 (C-21), 56.6 (C-1), 54.8 (C-13), 54.5 (C-3), 54.5 (C-11), 41.0 (NCH_3_), 27.8 (C-4), 20.9 (C-14), 20.6 (28-CH_3_), 16.0 (27-CH_3_), 9.7 (6-CH_3_), 8.6 (16-CH_3_); HRESIMS *m/z* 731.2711 ([M+H]^+^, calculated for C_41_H_39_N_4_O_9_, 731.2712).


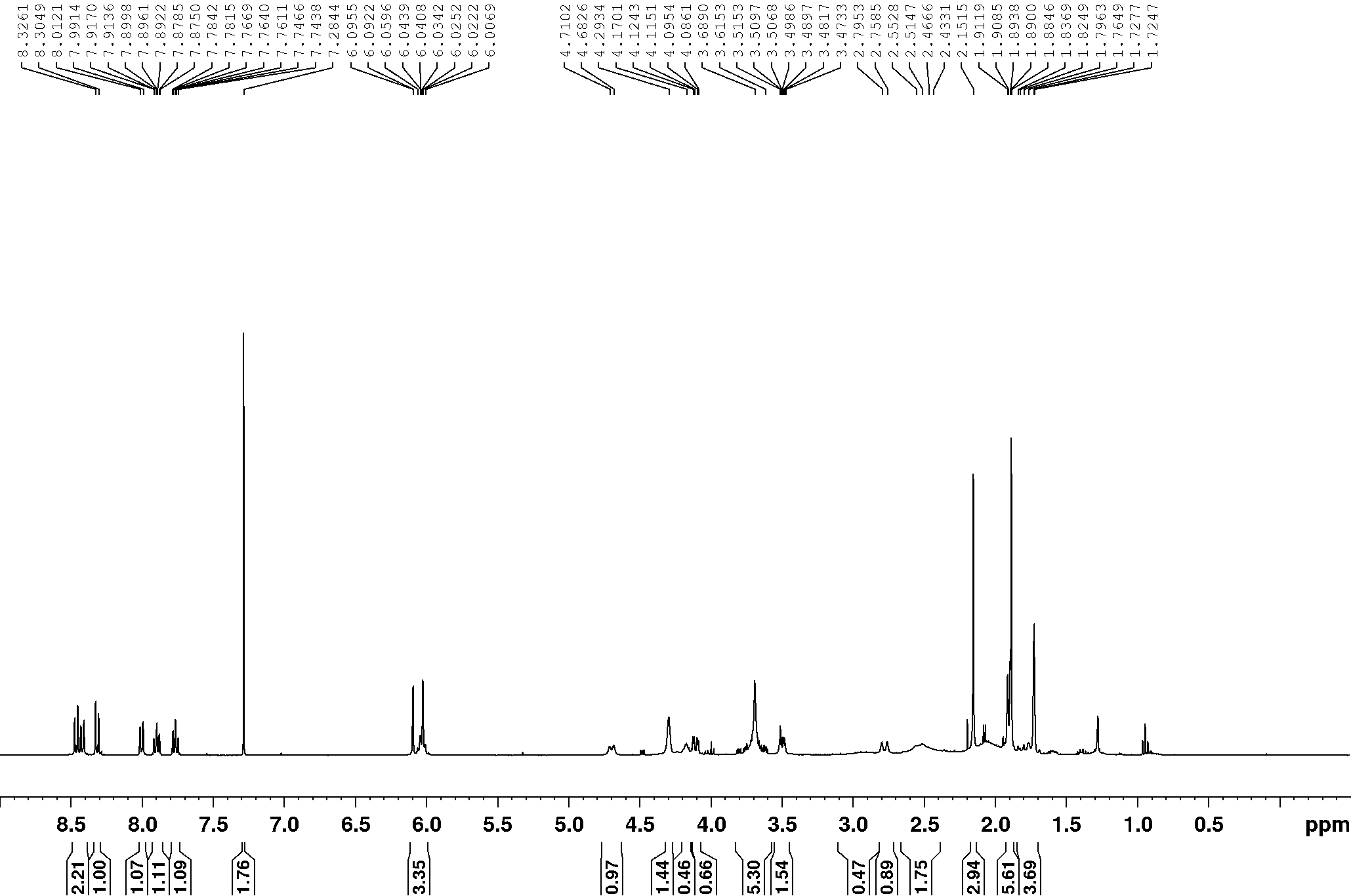


g, h

g, h

g: grease, h: hexane

**Figure S43.** ^1^H NMR (400 MHz) spectrum of **3i** in CDCl_3_


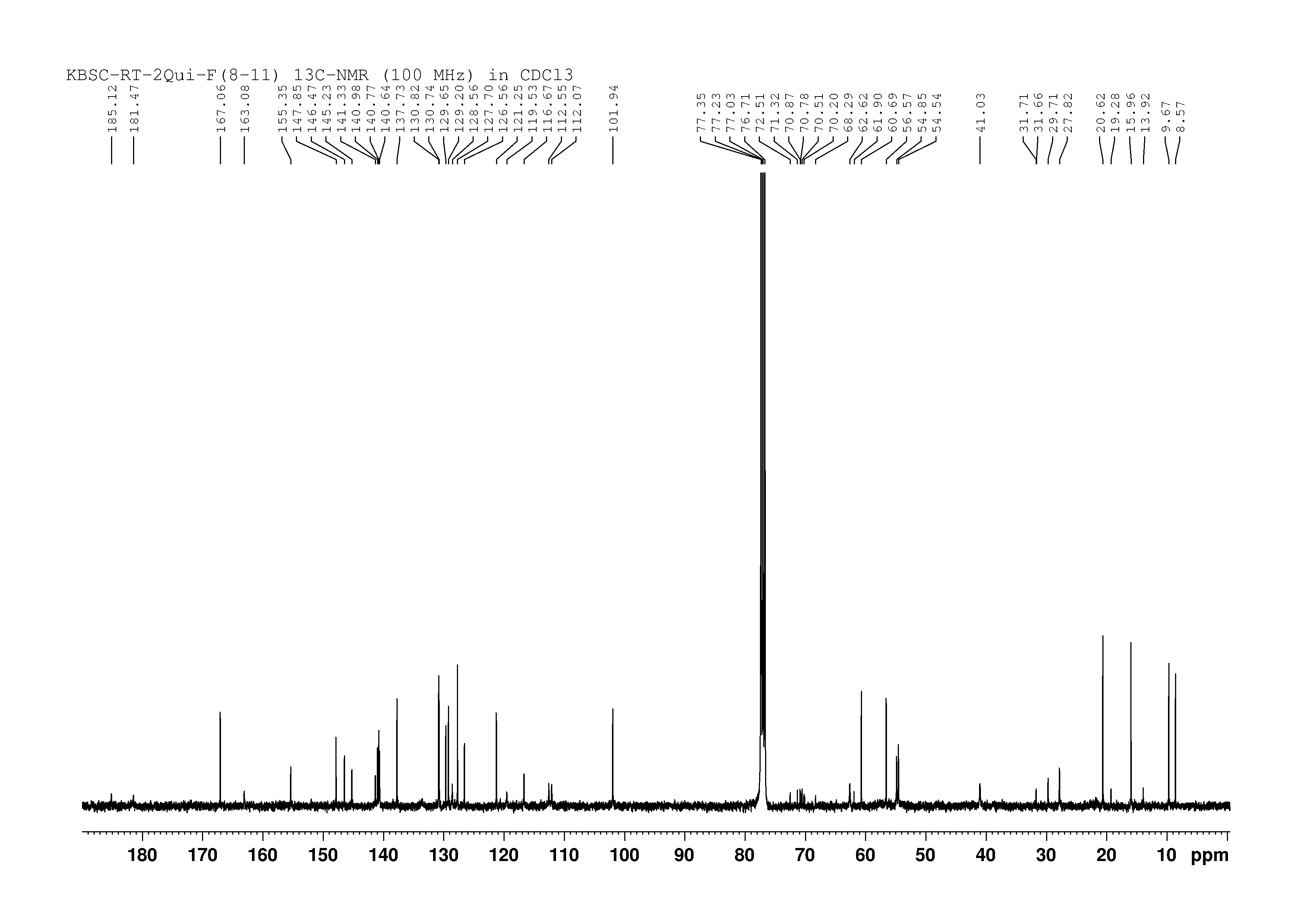


h

g

g: grease, h: hexane

h

h

**Figure S44.** ^13^C NMR (100 MHz) spectrum of **3i** in CDCl_3_


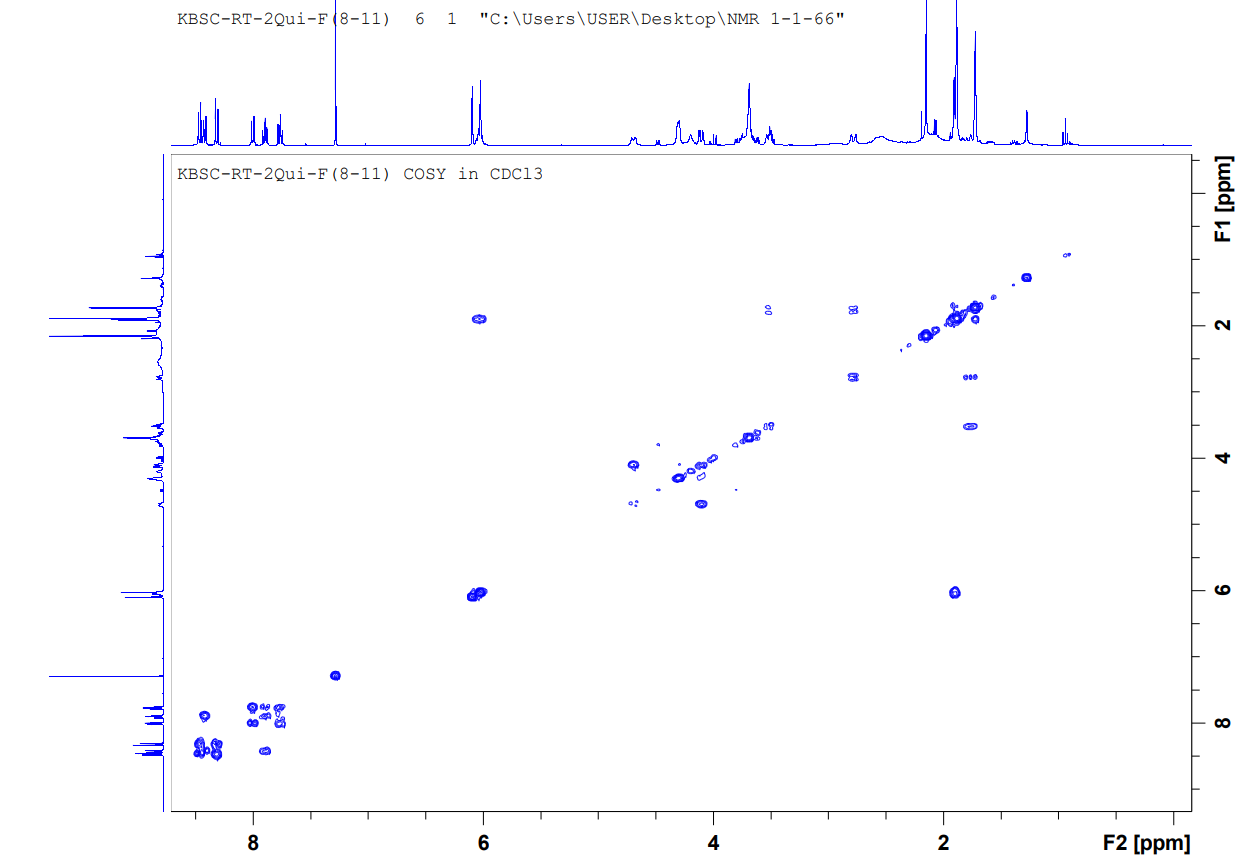


**Figure S45.** COSY (400 MHz) spectrum of **3i** in CDCl_3_


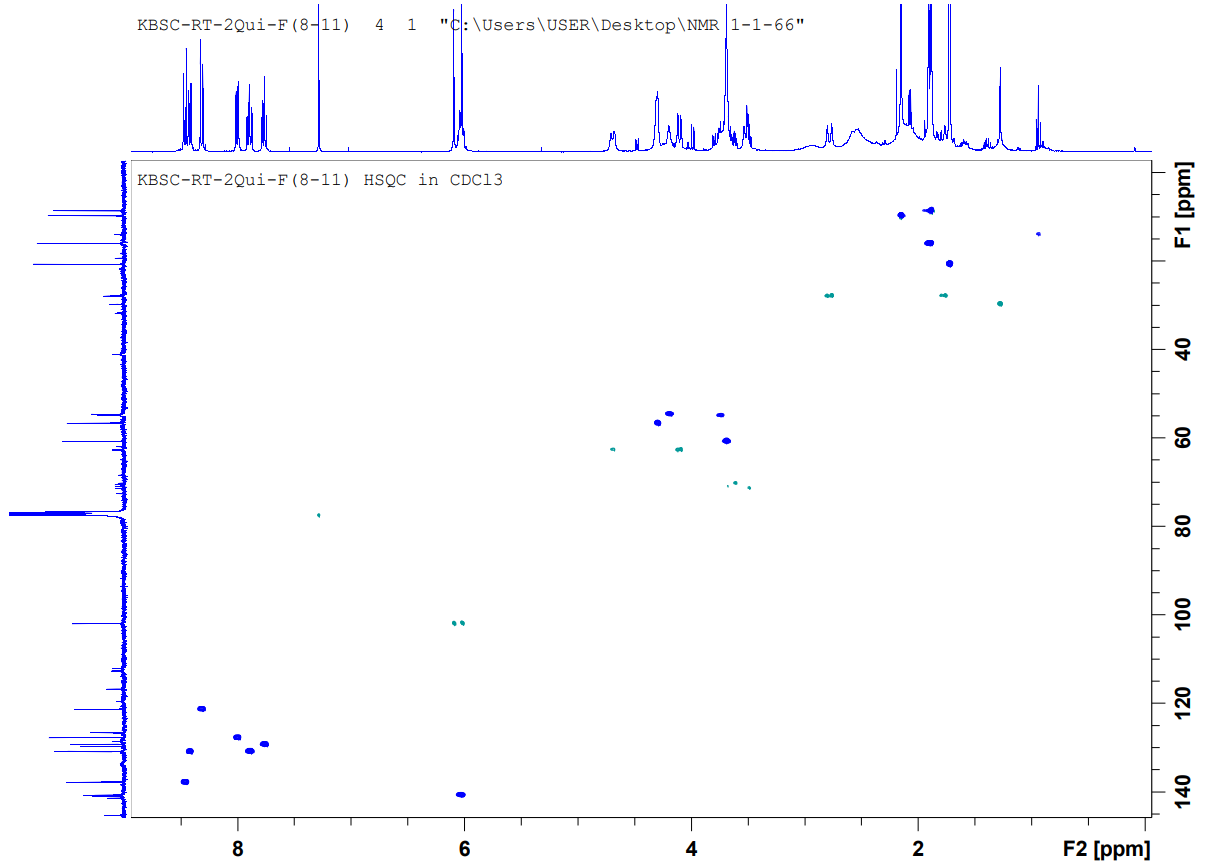


**Figure S46.** HSQC (400 MHz) spectrum of **3i** in CDCl_3_


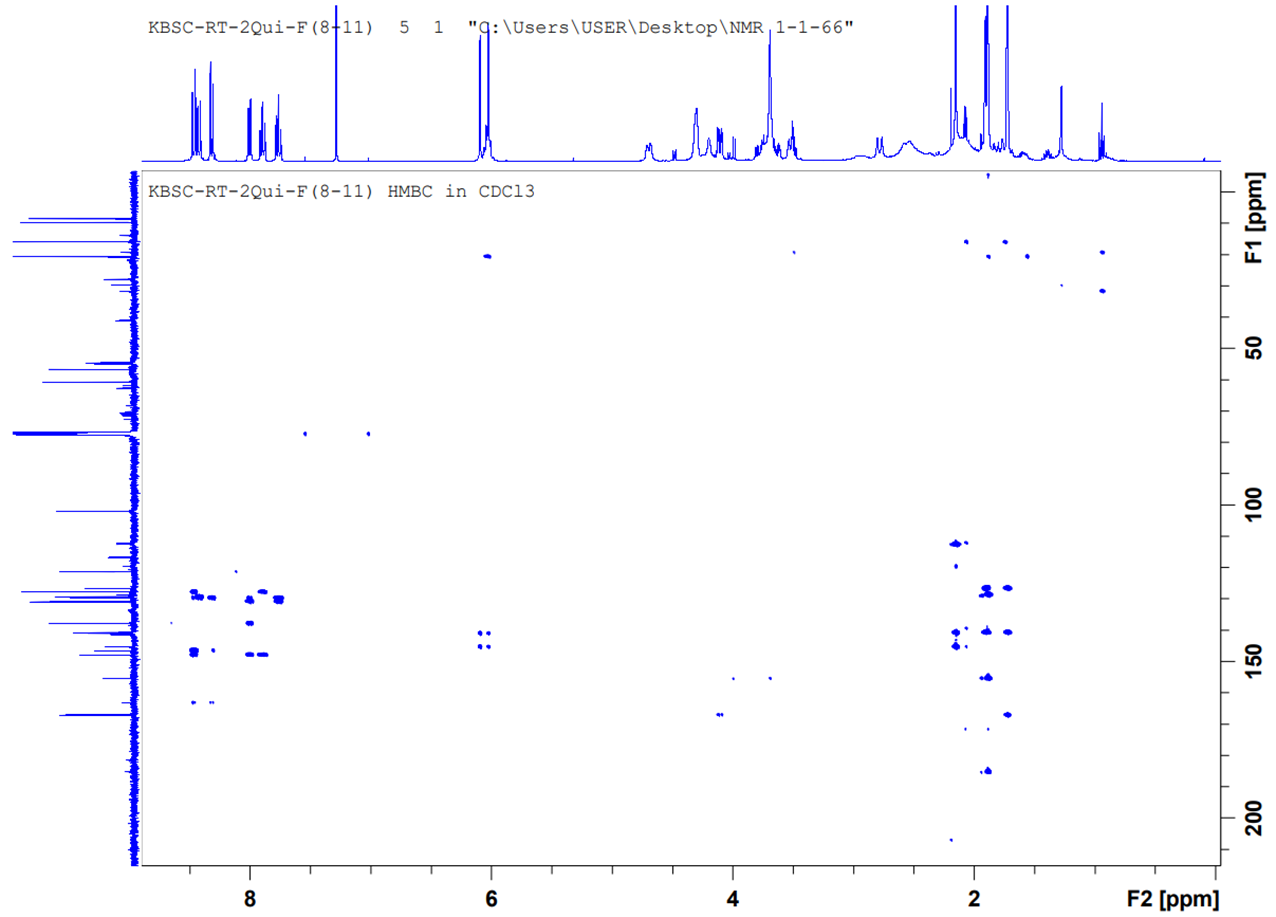


**Figure S47.** HMBC (400 MHz) spectrum of **3i** in CDCl_3_


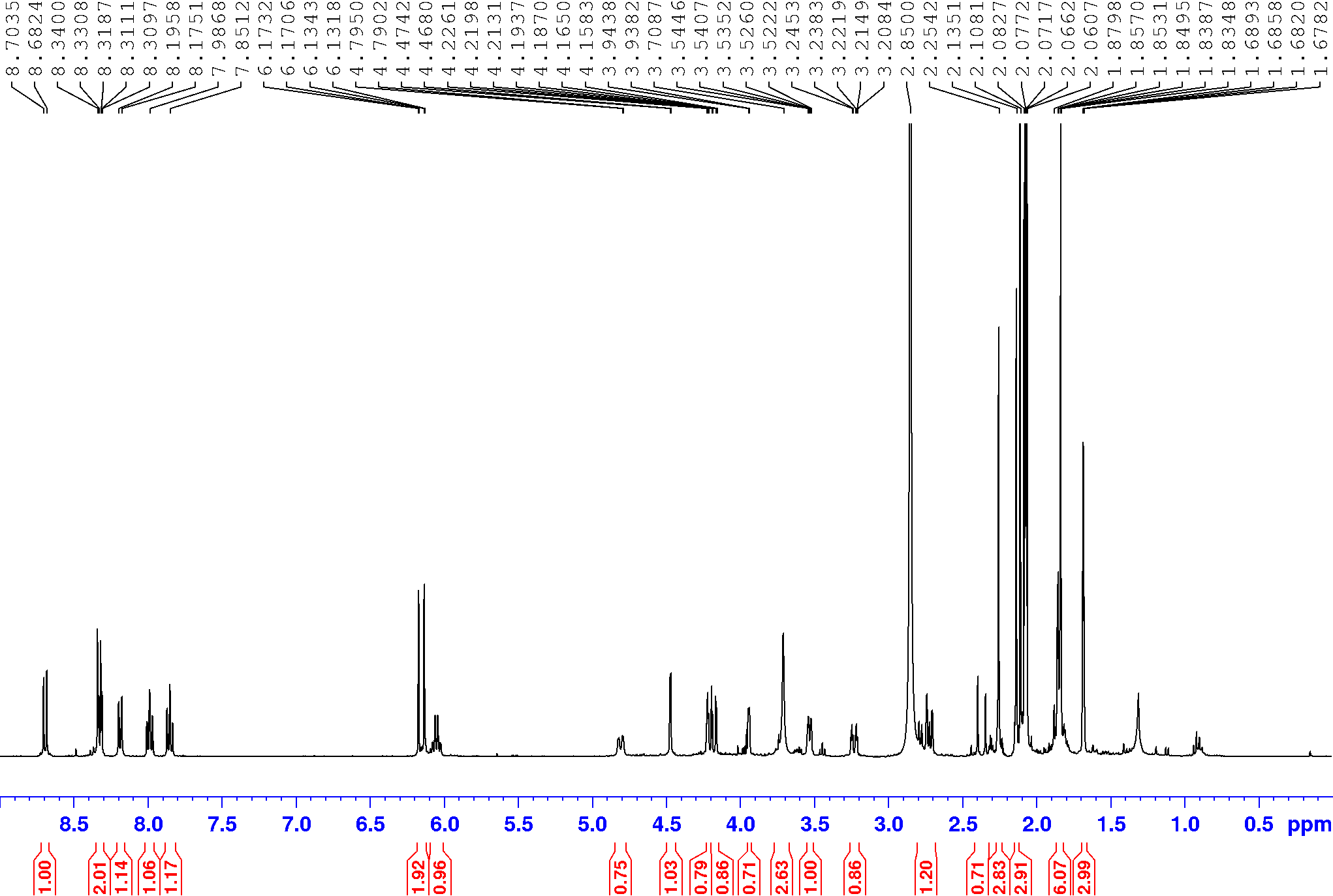


g

g

g: grease

**Figure S48.** ^1^H NMR (400 MHz) spectrum of **3i** in (CD_3_)_2_CO

^1^H NMR ((CD_3_)_2_CO, 400 MHz) δ 8.69 (1H, d, *J* = 8.4 Hz, 10'-H), 8.33 (1H, d, *J* = 8.5 Hz, 8'-H), 8.32 (1H, d, *J* = 8.4 Hz, 11'-H), 8.19 (1H, d, *J* = 8.5 Hz, 5'-H), 7.99 (1H, ddd, *J* = 8.5, 6.9, 1.4 Hz, 7'-H), 7.85 (1H, ddd, *J* = 8.5, 6.9, 1.4 Hz, 6'-H), 6.15 (2H, dd, *J* = 15.6, 1.0 Hz, OCH_2_O), 6.05 (1H, qq, *J* = 7.3, 1.5 Hz, 26-H), 4.81 (1H, dd, *J* = 11.2, 1.8 Hz, 22-H_α_), 4.47 (1H, d, *J* = 2.5 Hz, 1-H), 4.22 (1H, t, *J* = 2.6 Hz, 21-H), 4.18 (1H, dd, *J* = 11.2, 2.5 Hz, 22-H_β_), 3.94 (1H, br d, *J* = 2.3 Hz, 11-H), 3.71 (3H, s, 17-OCH_3_), 3.53 (1H, ddd, *J* = 7.4, 2.6, 1.5 Hz, 13-H), 3.23 (1H, dt, *J* = 12.1, 2.3 Hz, 3-H), 2.76 (1H, dd, *J* = 21.0, 7.4 Hz, 14-H_α_), 2.72 (1H, br d, *J* = 2.3 Hz, 4-H_α_), 2.37 (1H, d, *J* = 21.0 Hz, 14-H_β_), 2.25 (3H, s, NCH_3_), 2.21 (3H, s, 6-CH_3_), 1.85 (3H, dq, *J* = 7.3, 1.5 Hz, 27-CH_3_), 1.83 (3H, s, 16-CH_3_), 1.80 (1H, overlapped, 4-H_β_), 1.68 (3H, dq, *J* = 1.5, 1.4 Hz, 28-CH_3_).

# Physical and spectroscopic data of **3j**

*5-O-(3-quinolinecarbonyl) ester derivative of renieramycin T,* **3j**: The title compound was synthesized from **2** (28.3 mg, 0.05 mmol), DMAP (15.0 mg, 0.12 mmol), EDCI (23.3 mg, 0.12 mmol) and 3-quinoline carboxylic acid (42.6 mg, 0.25 mmol) to afford **3j**; yield 47% (brsm); yellow amorphous powder; $[]_{D}^{25}$ −64.6 (*c* 0.17, CHCl_3_); ECD Δ*ε* (*c* 82.11 μM, methanol, 20 ^o^C) −0.2 (308), −1.7 (295), +13.8 (260), +22.1 (245), −12.6 (222), −14.1 (215), +0.2 (207), +14.2 (201), −1.6 (199), −23.2 (195), +32.7 (192) nm; IR (ATR) ν_max_ 2949, 2838, 1653, 1450, 1411, 1112, 1022, 684 cm^−1^; ^1^H NMR (CDCl_3_, 400 MHz) δ 9.54 (1H, s, 3'-H), 9.11 (1H, s, 11'-H), 8.37 (1H, d, *J* = 7.6 Hz, 6'-H), 8.10 (1H, d, *J* = 7.6 Hz, 9'-H), 7.98 (1H, t, *J* = 7.6 Hz, 7'-H), 7.77 (1H, t, *J* = 7.6 Hz, 8'-H), 6.03 (2H, dd, *J* = 26.4, 1.2 Hz, OCH_2_O), 6.02 (1H, overlapped, 26-H), 4.63 (1H, d, *J* = 11.6 Hz, 22-H_α_), 4.22 (1H, br t, *J* = 4.0 Hz, 1-H), 4.15 (1H, d, *J* = 2.0 Hz, 21-H), 4.08 (1H, dd, *J* = 11.6, 4.0 Hz, 22-H_β_), 3.91 (1H, br d, *J* = 2.4 Hz, 11-H), 3.69 (3H, s, 17-OCH_3_), 3.37 (1H, d, *J* = 7.6 Hz, 13-H), 3.28 (1H, br d, *J* = 12.0 Hz, 3-H), 2.72 (1H, dd, *J* = 20.8, 7.6 Hz, 14-H_α_), 2.61 (1H, dd, *J* = 15.2, 2.0 Hz, 4-H_α_), 2.29 (1H, d, *J* = 20.8 Hz, 14-H_β_), 2.22 (3H, s, NCH_3_), 2.09 (3H, s, 6-CH_3_), 1.89 (3H, overlapped, 27-CH_3_), 1.88 (3H, s, 16-CH_3_), 1.73 (1H, overlapped, 4-H_β_), 1.70 (3H, s, 28-CH_3_); ^13^C NMR (CDCl_3_, 100 MHz) δ 185.8 (C-15), 182.6 (C-18), 167.0 (C-24), 161.4 (C-1'), 155.2 (C-17), 146.3 (C-3'), 145.2 (C-7), 144.0 (C-11'), 141.8 (C-20), 141.4 (C-8), 140.5 (C-26), 139.9 (C-5), 135.4 (C-19), 135.0 (C-7'), 130.0 (C-8'), 130.0 (C-9'), 129.6 (C-5'), 129.1 (C-16), 127.6 (C-10'), 126.9 (C-25), 125.6 (C-6'), 122.3 (C-2'), 120.1 (C-6), 117.2 (21-CN), 112.6 (C-10), 111.9 (C-9), 102.0 (OCH_2_O), 62.8 (C-22), 60.6 (17-OCH_3_), 58.7 (C-21), 56.5 (C-1), 55.2 (C-3), 54.7 (C-13), 54.6 (C-11), 41.4 (NCH_3_), 28.1 (C-4), 21.2 (C-14), 20.5 (28-CH_3_), 15.9 (27-CH_3_), 9.6 (6-CH_3_), 8.6 (16-CH_3_); HRESIMS *m/z* 731.2711 ([M+H]^+^, calculated for C_41_H_39_N_4_O_9_, 731.2712).


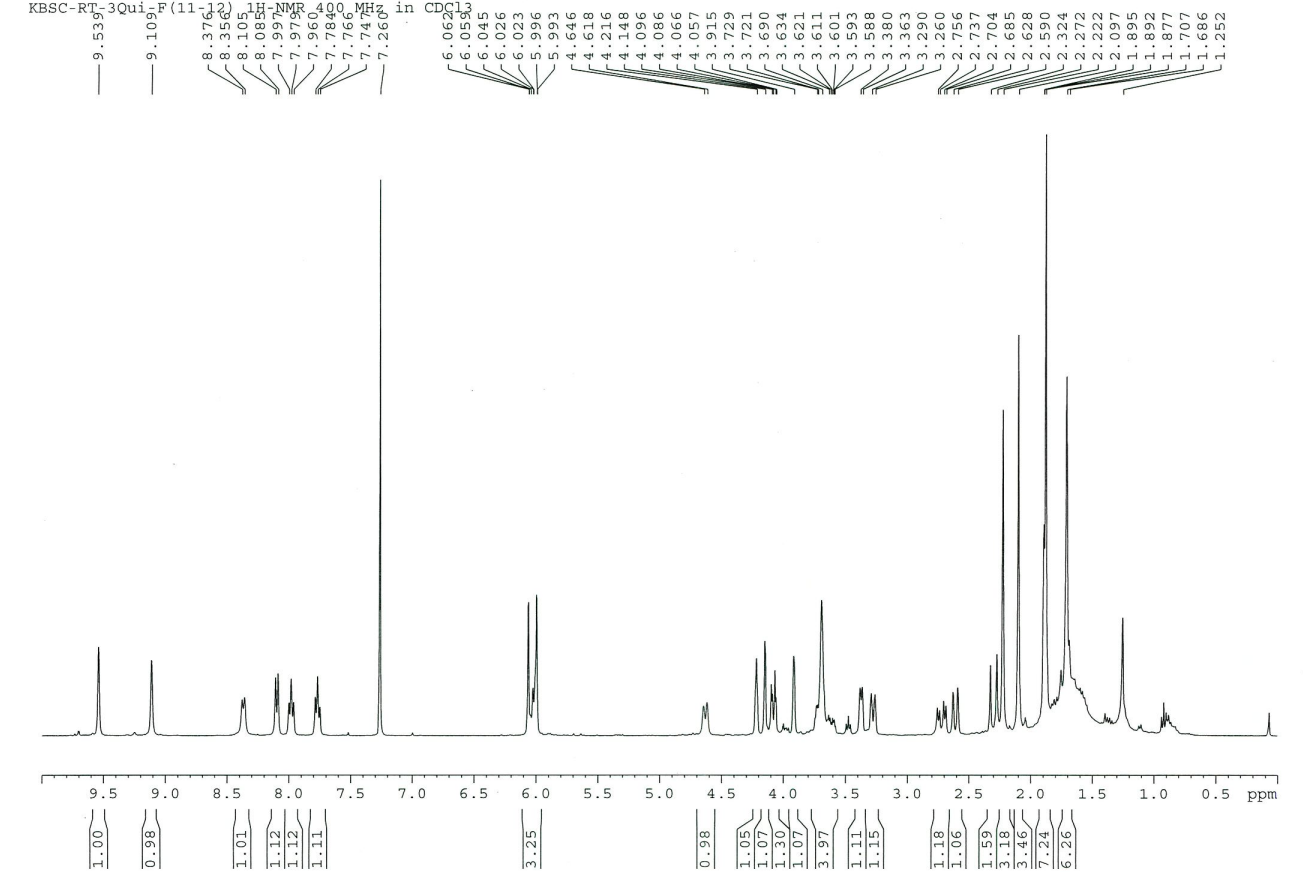


g, h

g, h

g: grease, h: hexane

**Figure S49.** ^1^H NMR (400 MHz) spectrum of **3j** in CDCl_3_


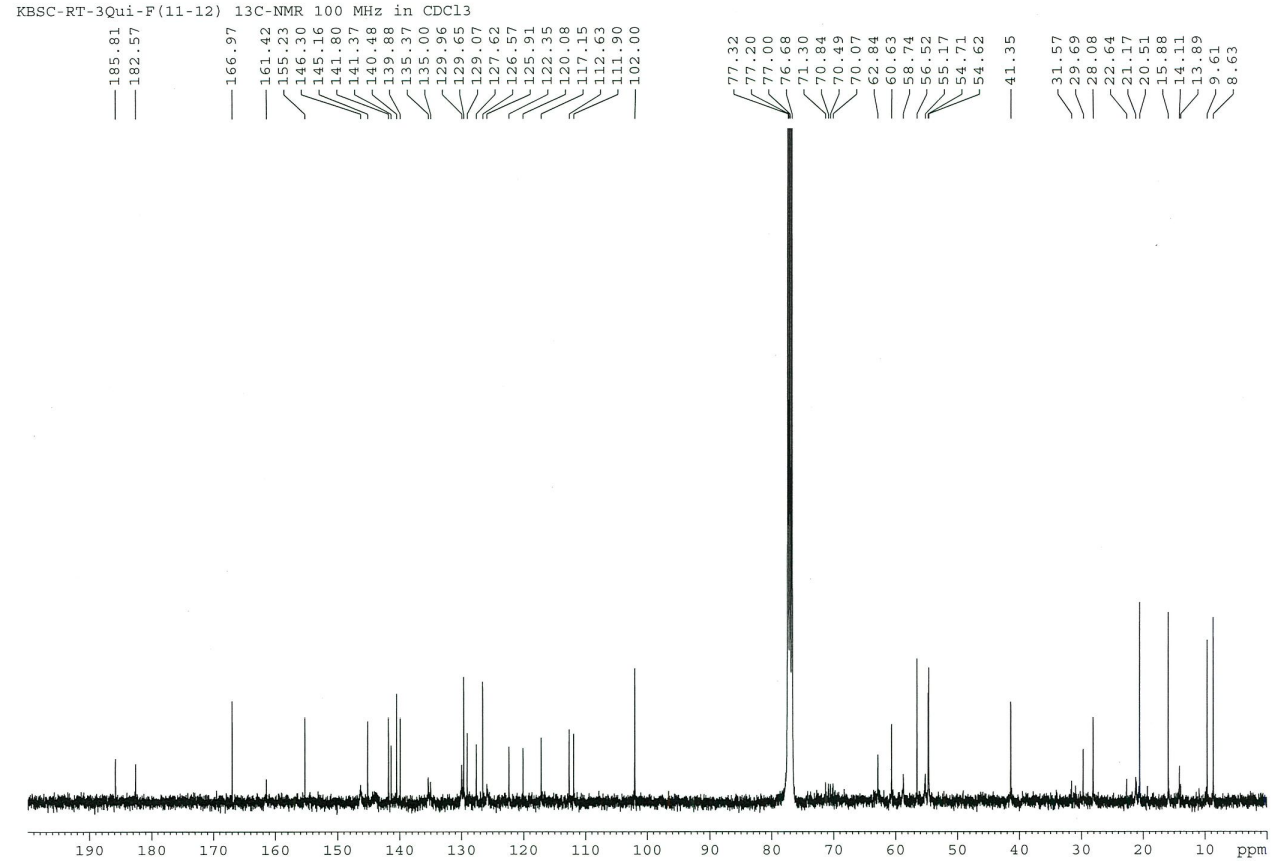


g

g: grease, h: hexane

h

h

h

**Figure S50.** ^13^C NMR (100 MHz) spectrum of **3j** in CDCl_3_


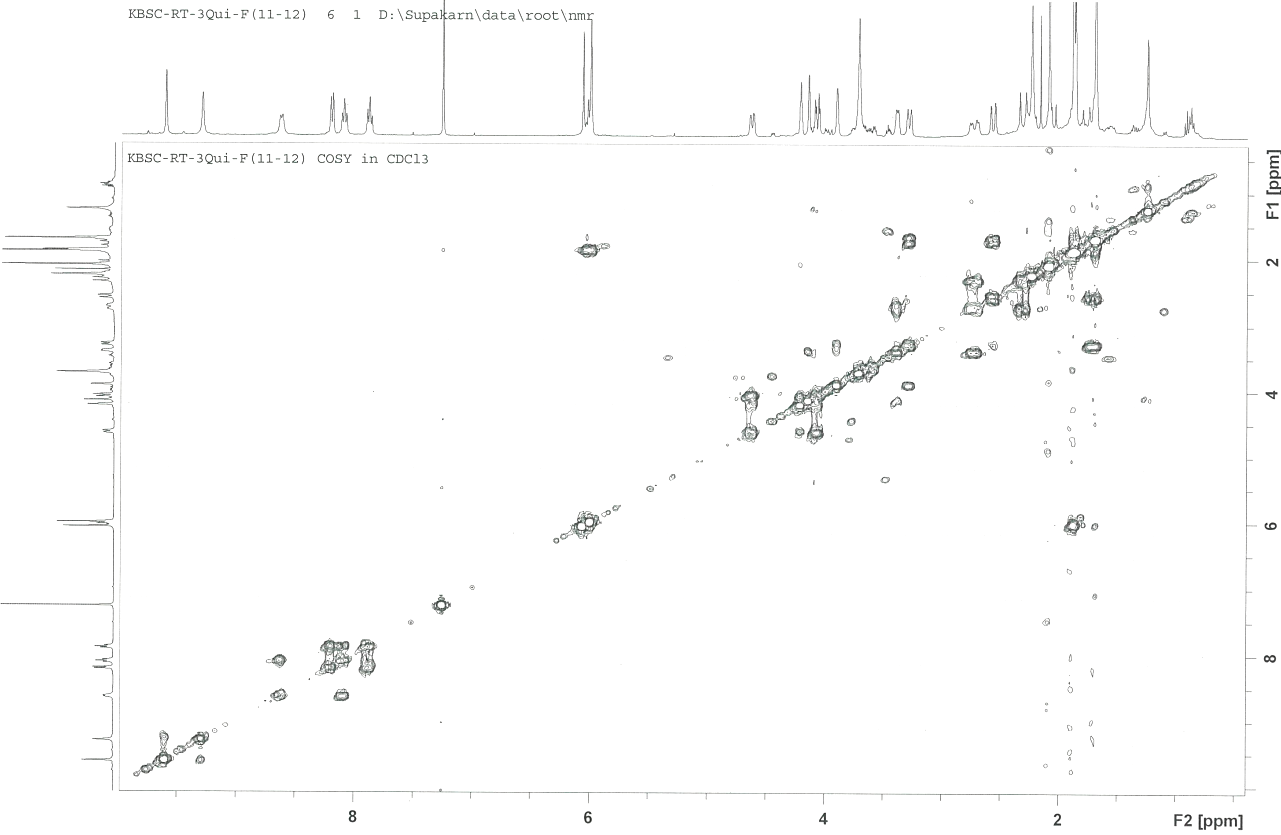


**Figure S51.** COSY (400 MHz) spectrum of **3j** in CDCl_3_


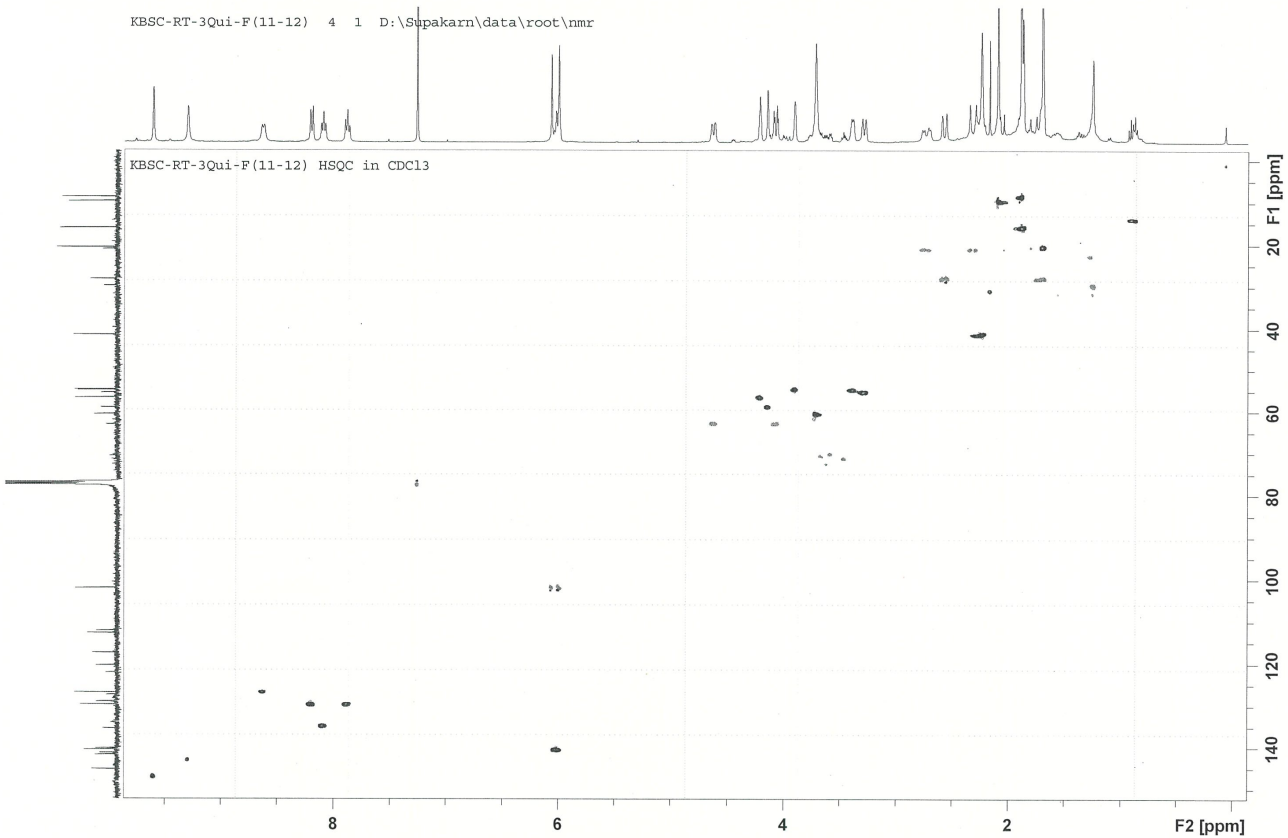


**Figure S52.** HSQC (400 MHz) spectrum of **3j** in CDCl_3_


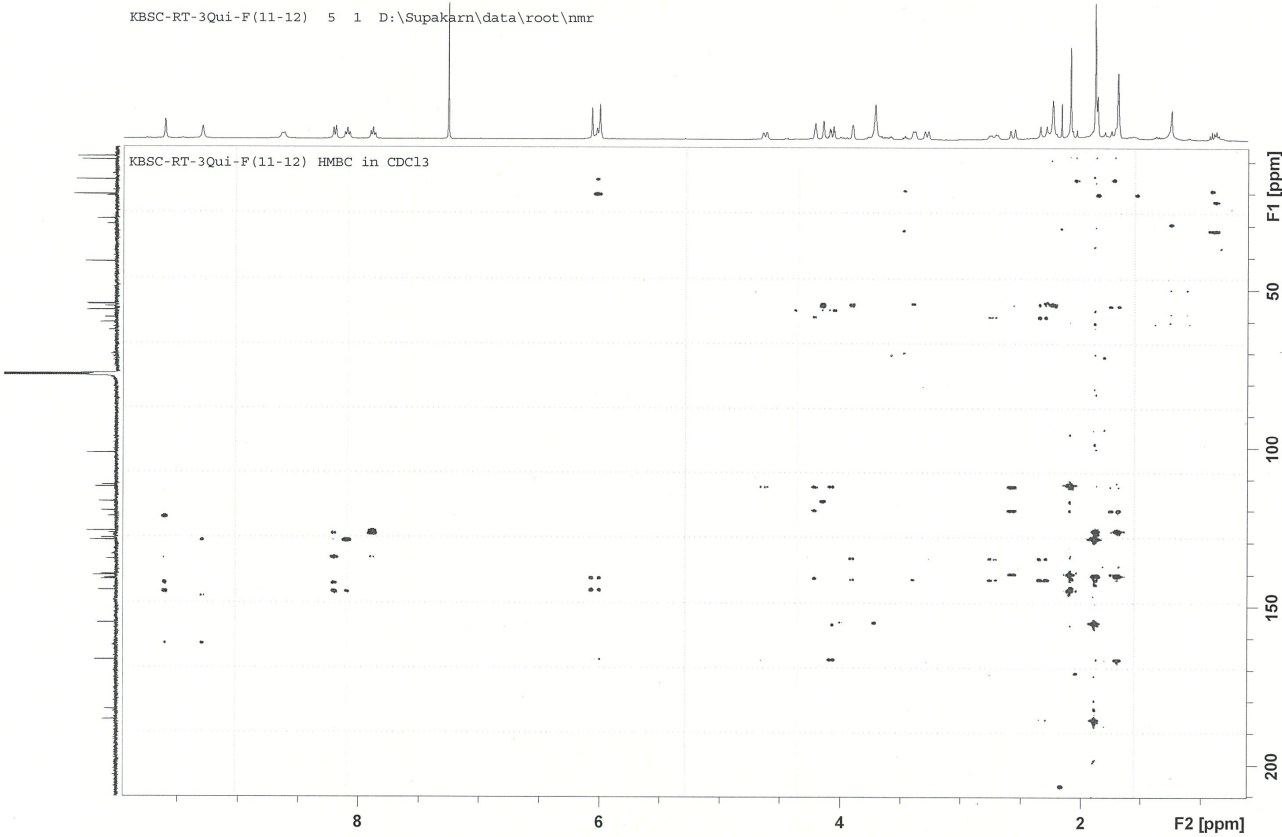


**Figure S53.** HMBC (400 MHz) spectrum of **3j** in CDCl_3_

# Physical and spectroscopic data of **3k**

*5-O-(3-pyridine acryloyl) ester derivative of renieramycin T,* **3k**: The title compound was synthesized from **2** (24.3 mg, 0.04 mmol), DMAP (12.9 mg, 0.11 mmol), EDCI (19.9 mg, 0.11 mmol) and 3-(3-pyridyl)acrylic acid (31.5 mg, 0.21 mmol) to afford **3k**; yield 81% (brsm); yellow amorphous powder; $[]_{D}^{25}$ +45.0 (*c* 0.10, CHCl_3_); ECD Δ*ε* (*c* 62.26 μM, methanol, 20 ^o^C) −1.3 (330), +6.6 (272), −0.6 (241), −4.2 (221), −3.3 (218), +0.6 (211), −5.9 (204), −10.7 (201), +6.9 (199), −9.7 (196), +12.5 (193) nm; IR (ATR) ν_max_ 2923, 1715, 1651, 1451, 1305, 1231, 1144, 1084, 954, 698 cm^−1^; ^1^H NMR (CDCl_3_, 400 MHz) δ 8.94 (1H, br s, 5'-H), 8.75 (1H, d, *J* = 4.0 Hz, 7'-H), 8.24 (1H, d, *J* = 8.0 Hz, 9'-H), 7.84 (1H, d, *J* = 16.0 Hz, 3'-H), 7.67 (1H, m, 8'-H), 6.81 (1H, d, *J* = 16.0 Hz, 2'-H), 6.02 (1H, overlapped, 26-H), 6.00 (2H, dd, *J* = 25.6, 1.6 Hz, OCH_2_O), 4.57 (1H, dd, *J* = 11.6, 3.6 Hz, 22-H_α_), 4.19 (1H, t, *J* = 3.6 Hz, 1-H), 4.13 (1H, d, *J* = 2.0 Hz, 21-H), 4.06 (1H, dd, *J* = 11.6, 4.5 Hz, 22-H_β_), 3.99 (1H, d, *J* = 2.0 Hz, 11-H), 3.86 (3H, s, 17-OCH_3_), 3.39 (1H, br d, *J* = 7.6 Hz, 13-H), 3.26 (1H, dt, *J* = 12.0, 2.0 Hz, 3-H), 2.74 (1H, dd, *J* = 20.8, 7.6 Hz, 14-H_α_), 2.57 (1H, dd, *J* = 15.2, 2.0 Hz, 4-H_α_), 2.29 (1H, overlapped, 14-H_β_), 2.27 (3H, s, NCH_3_), 2.05 (3H, s, 6-CH_3_), 1.91 (3H, s, 16-CH_3_), 1.89 (3H, dq, *J* = 7.2, 1.6 Hz, 27-CH_3_), 1.69 (3H, dq, *J* = 1.6, 1.6 Hz, 28-CH_3_), 1.68 (1H, overlapped, 4-H_β_); ^13^C NMR (CDCl_3_, 100 MHz) δ 185.8 (C-15), 182.5 (C-18), 167.0 (C-24), 163.0 (C-1'), 155.4 (C-17), 145.1 (C-7), 141.9 (C-9'), 141.9 (C-7'), 140.4 (C-26), 140.1 (C-20), 140.1 (C-5'), 140.1 (C-8), 140.0 (C-5), 139.2 (C-3'), 135.8 (C-19), 133.2 (C-4'), 129.3 (C-16), 126.6 (C-8'), 126.4 (C-25), 123.0 (C-2'), 119.9 (C-6), 117.1 (21-CN), 112.3 (C-10), 112.0 (C-9), 101.9 (OCH_2_O), 63.3 (C-22), 60.7 (17-OCH_3_), 59.0 (C-21), 56.4 (C-1), 54.8 (C-3), 54.8 (C-11), 54.8 (C-13), 41.4 (NCH_3_), 27.8 (C-4), 22.7 (C-14), 20.5 (28-CH_3_), 15.9 (27-CH_3_), 9.6 (6-CH_3_), 8.7 (16-CH_3_); HRESIMS *m/z* 707.2712 ([M+H]^+^, calculated for C_39_H_39_N_4_O_9_, 707.2712).


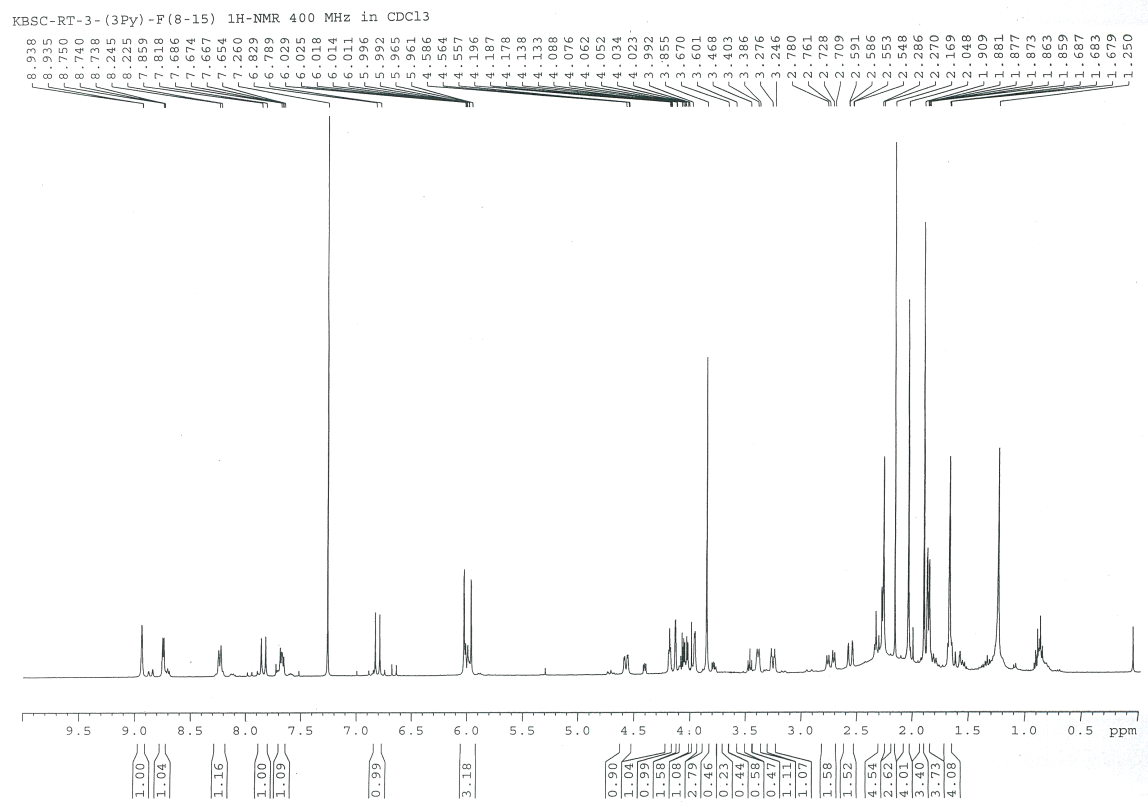


g, h

g, h

g: grease, h: hexane

**Figure S54.** ^1^H NMR (400 MHz) spectrum of **3k** in CDCl_3_


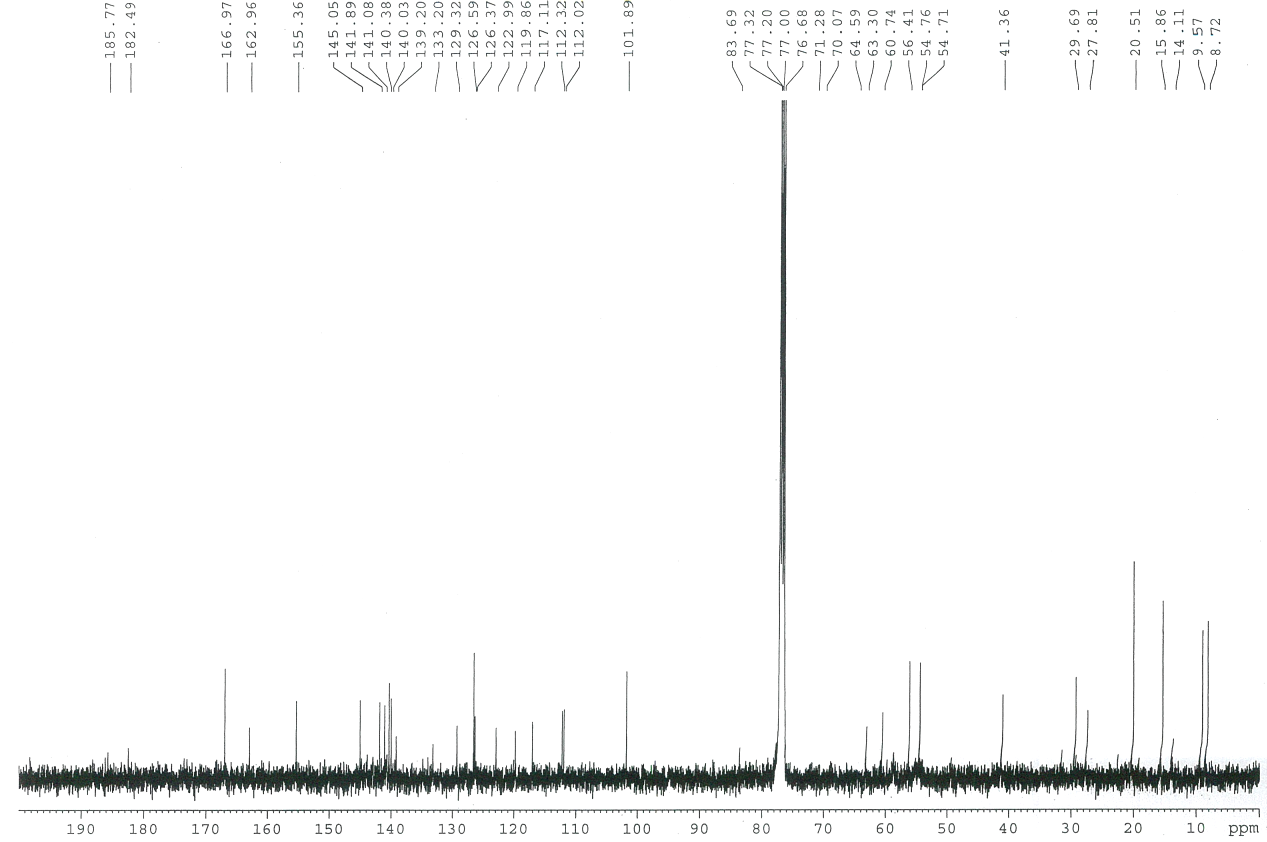


h

g

h

g: grease, h: hexane

h

**Figure S55.** ^13^C NMR (100 MHz) spectrum of **3k** in CDCl_3_


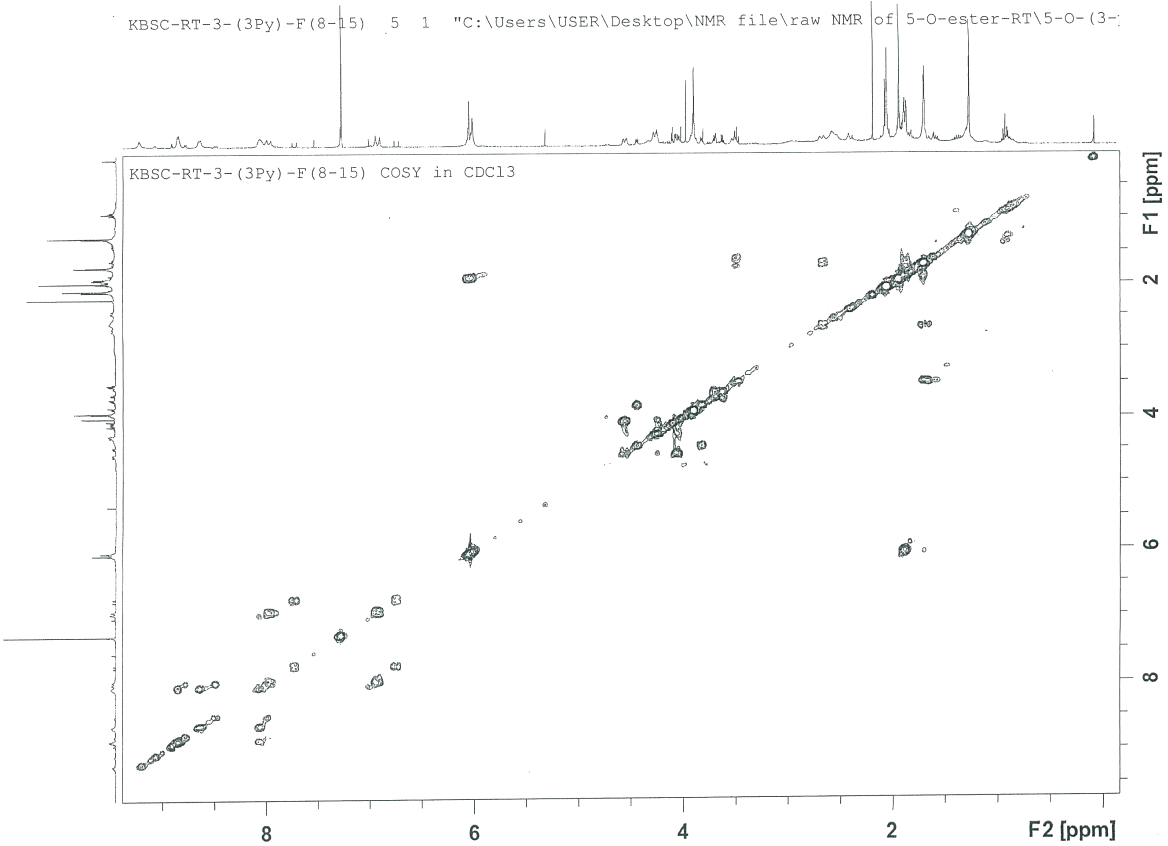


**Figure S56.** COSY (400 MHz) spectrum of **3k** in CDCl_3_


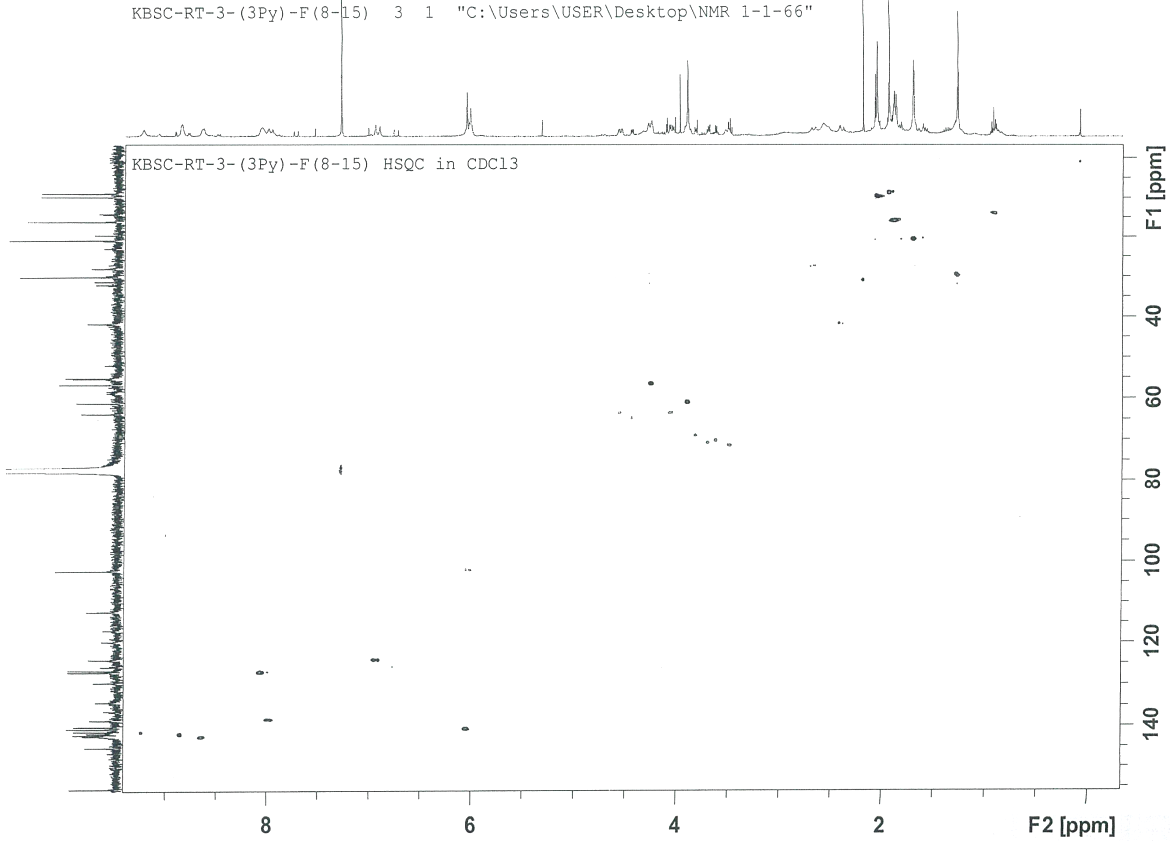


**Figure S57.** HSQC (400 MHz) spectrum of **3k** in CDCl_3_


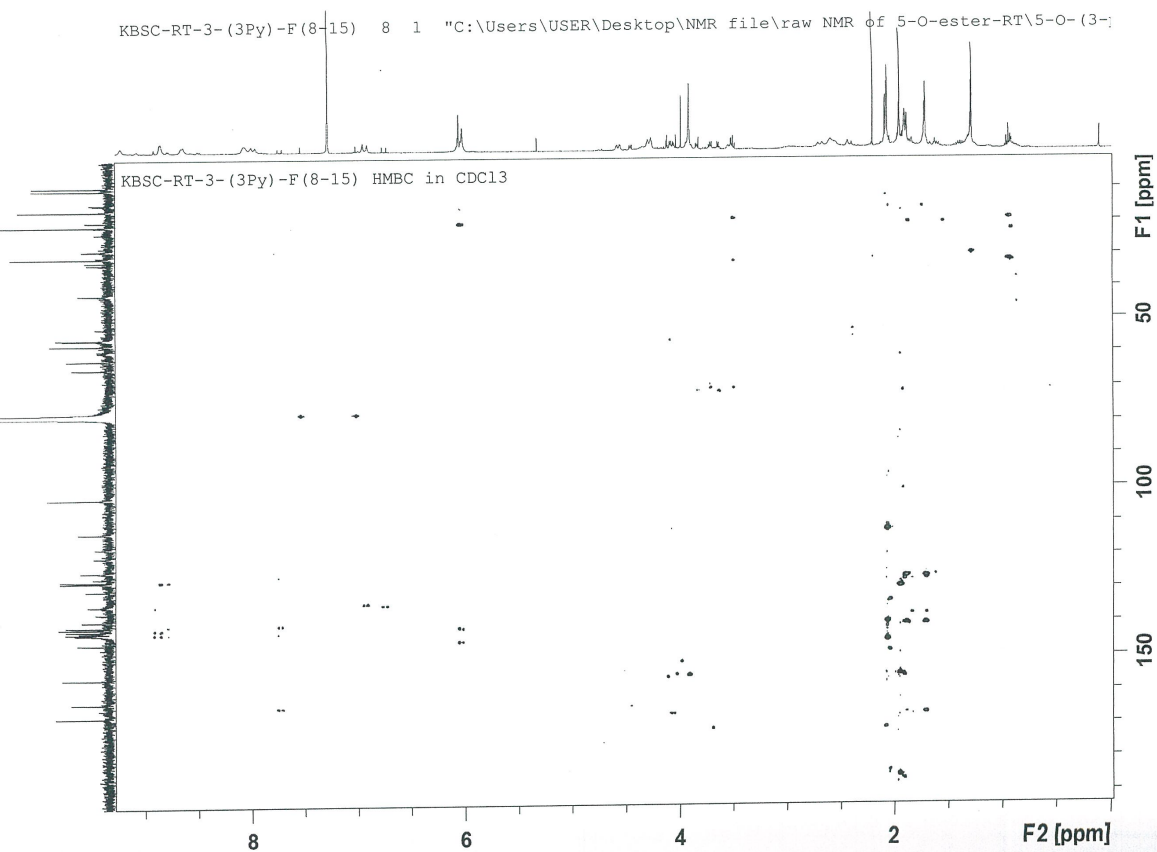


**Figure S58.** HMBC (400 MHz) spectrum of **3k** in CDCl_3_


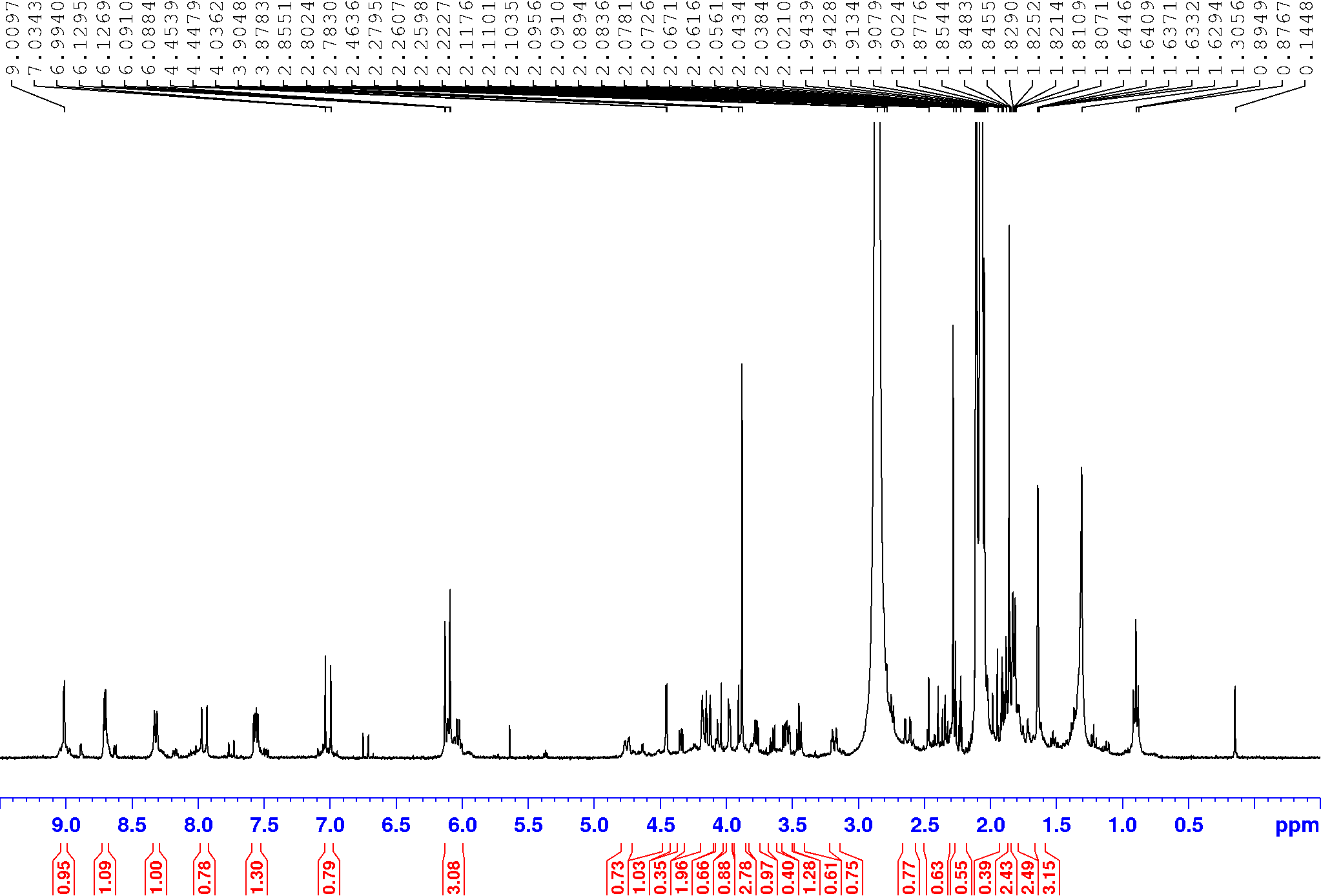


g

g

g: grease

**Figure S59.** ^1^H NMR (400 MHz) spectrum of **3k** in (CD_3_)_2_CO

^1^H NMR ((CD_3_)_2_CO, 400 MHz) δ 9.01 (1H, d, *J* = 2.1 Hz, 5'-H), 8.70 (1H, d, *J* = 4.7 Hz, 7'-H), 8.32 (1H, ddd, *J* = 8.0, 2.1, 1.8 Hz, 9'-H), 7.95 (1H, d, *J* = 16.2 Hz, 3'-H), 7.56 (1H, dd, *J* = 8.0, 4.7 Hz, 8'-H), 7.01 (1H, d, *J* = 16.2 Hz, 2'-H), 6.11 (2H, dd, *J* = 15.4, 1.0 Hz, OCH_2_O), 6.03 (1H, qq, *J* = 7.2, 1.5 Hz, 26-H), 4.75 (1H, dd, *J* = 11.5, 2.4 Hz, 22-H_α_), 4.45 (1H, d, *J* = 2.4 Hz, 1-H), 4.18 (1H, t, *J* = 2.7 Hz, 21-H), 4.13 (1H, dd, *J* = 11.5, 2.4 Hz, 22-H_β_), 3.97 (1H, overlapped, 11-H), 3.88 (3H, s, 17-OCH_3_), 3.54 (1H, overlapped, 13-H), 3.18 (1H, dt, *J* = 12.2, 2.4 Hz, 3-H), 2.77 (1H, dd, *J* = 20.8, 7.5 Hz, 14-H_α_), 2.62 (1H, dd, *J* = 15.2, 2.4 Hz, 4-H_α_), 2.43 (1H, overlapped, 14-H_β_), 2.28 (3H, s, NCH_3_), 2.04 (3H, s, 6-CH_3_), 1.85 (3H, s, 16-CH_3_), 1.82 (3H, dq, *J* = 7.2, 1.5 Hz, 27-CH_3_), 1.64 (3H, dq, *J* = 1.5, 1.5 Hz, 28-CH_3_), 1.61 (1H, overlapped, 4-H_β_).

# Physical and spectroscopic data of **3l**

*5-O-(3-indolecarbonyl) ester derivative of renieramycin T,* **3l**: The title compound was synthesized from **2** (25.9 mg, 0.04 mmol), DMAP (13.7 mg, 0.11 mmol), EDCI (21.2 mg, 0.11 mmol) and indole-3-carboxylic acid (36.3 mg, 0.22 mmol) to afford **3l**; yield 55% (brsm); yellow amorphous powder; $[]_{D}^{25}$ −21.1 (*c* 0.16, CHCl_3_); ECD Δ*ε* (*c* 33.39 μM, methanol, 20 ^o^C) +9.1 (262), −6.5 (227), +0.2 (212), +6.3 (205), −9.9 (202), +26.4 (195), −6.0 (191) nm; IR (ATR) ν_max_ 3347 (br), 2924, 1714, 1651, 1429, 1232, 1144, 1084, 750 cm^−1^; ^1^H NMR (CDCl_3_, 400 MHz) δ 8.87 (1H, br s, 4'-NH), 8.16 (1H, d, *J* = 2.8 Hz, 3'-H), 8.14 (1H, d, *J* = 8.0 Hz, 9'-H), 7.52 (1H, d, *J* = 8.0 Hz, 6'-H), 7.35 (1H, t, *J* = 8.0 Hz, 8'-H), 7.30 (1H, t, *J* = 8.0 Hz, 7'-H), 6.00 (1H, overlapped, 26-H), 6.01 (2H, dd, *J* = 24.0, 1.6 Hz, OCH_2_O), 4.66 (1H, d, *J* = 10.4 Hz, 22-H_α_), 4.24 (1H, overlapped, 1-H), 4.19 (1H, overlapped, 21-H), 4.09 (1H, br d, *J* = 10.4, 4.0 Hz, 22-H_β_), 4.00 (1H, br d, *J* = 2.6 Hz, 11-H), 3.41 (1H, overlapped, 13-H), 3.36 (3H, s, 17-OCH_3_), 3.30 (1H, dd, *J* = 11.4, 2.6 Hz, 3-H), 2.77 (1H, overlapped, 14-H_α_), 2.73 (1H, br d, *J* = 14.2, 2.6 Hz, 4-H_α_), 2.33 (1H, d, *J* = 17.2 Hz, 14-H_β_), 2.26 (3H, s, NCH_3_), 2.12 (3H, s, 6-CH_3_), 1.91 (3H, dq, *J* = 7.6, 1.6 Hz, 27-CH_3_), 1.86 (3H, s, 16-CH_3_), 1.74 (3H, overlapped, 28-CH_3_), 1.72 (1H, overlapped, 4-H_β_); ^13^C NMR (CDCl_3_, 100 MHz) δ 185.9 (C-15), 182.4 (C-18), 167.1 (C-24), 162.3 (C-1'), 155.6 (C-17), 144.9 (C-7), 141.2 (C-20), 140.6 (C-8), 140.5 (C-5), 140.3 (C-26), 136.2 (C-19), 136.2 (C-5'), 131.8 (C-3'), 127.9 (C-16), 126.8 (C-25), 126.1 (C-10'), 123.6 (C-8'), 122.4 (C-7'), 121.3 (C-9'), 120.7 (C-6), 117.4 (21-CN), 112.8 (C-10), 112.2 (C-9), 111.7 (C-6'), 107.0 (C-2'), 101.7 (OCH_2_O), 63.3 (C-22), 60.3 (17-OCH_3_), 58.9 (C-21), 56.6 (C-1), 55.4 (C-3), 55.0 (C-13), 54.5 (C-11), 41.4 (NCH_3_), 28.0 (C-4), 21.0 (C-14), 20.6 (28-CH_3_), 15.9 (27-CH_3_), 9.6 (6-CH_3_), 8.5 (16-CH_3_); HRESIMS *m/z* 719.2710 ([M+H]^+^, calculated for C_40_H_39_N_4_O_9_, 719.2712).


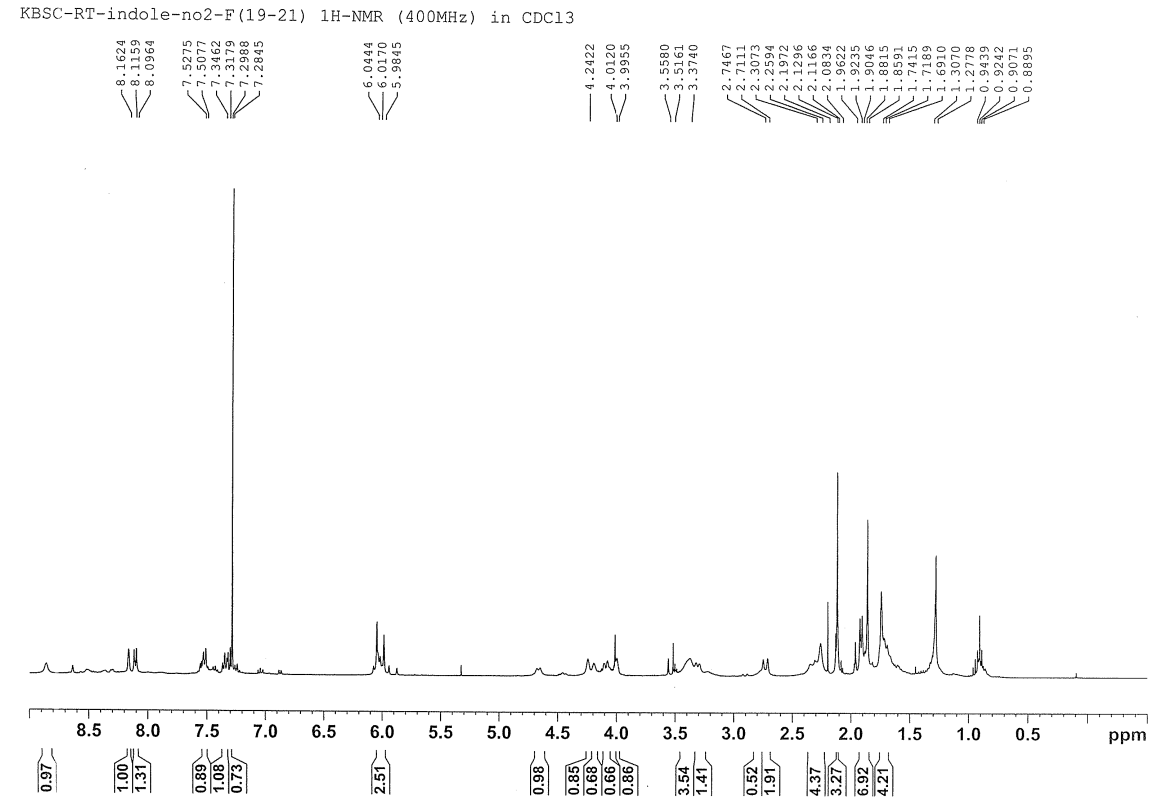


g, h

g, h

g: grease, h: hexane

**Figure S60.** ^1^H NMR (400 MHz) spectrum of **3l** in CDCl_3_


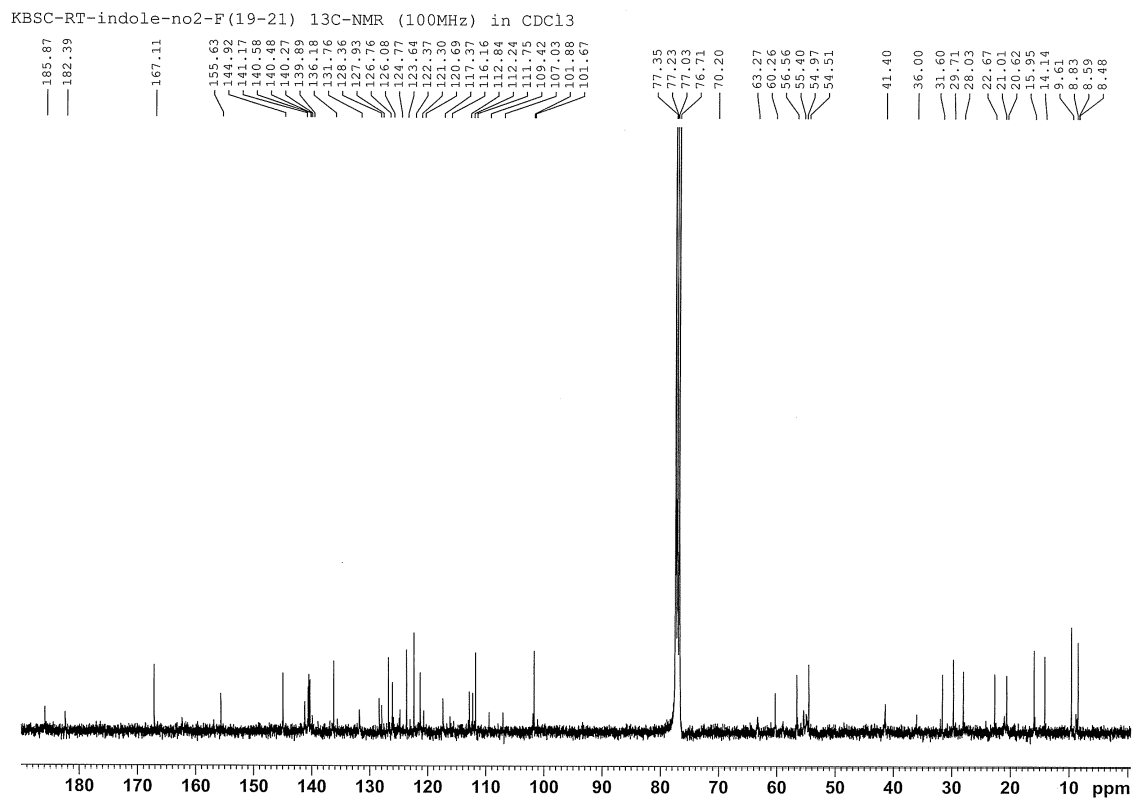


g: grease, h: hexane

g

h

h

h

**Figure S61.** ^13^C NMR (100 MHz) spectrum of **3l** in CDCl_3_


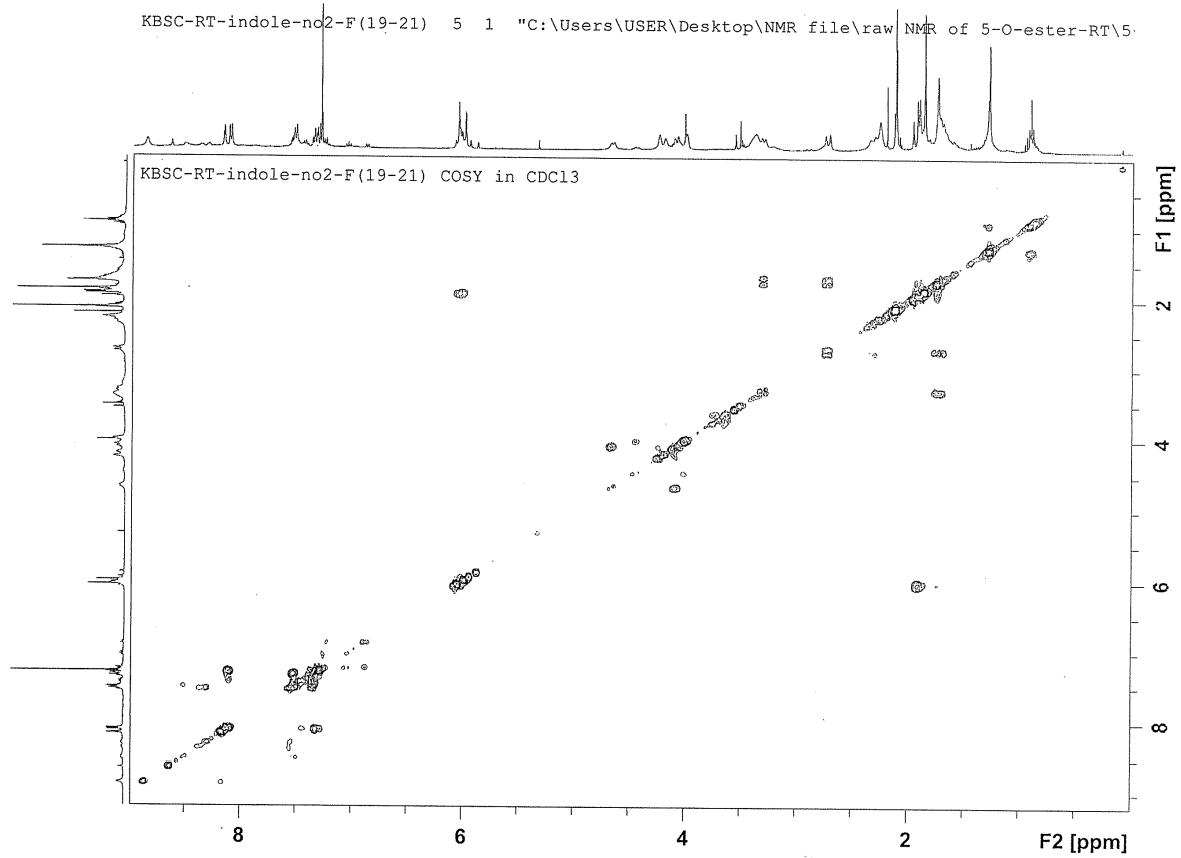


**Figure S62.** COSY (400 MHz) spectrum of **3l** in CDCl_3_


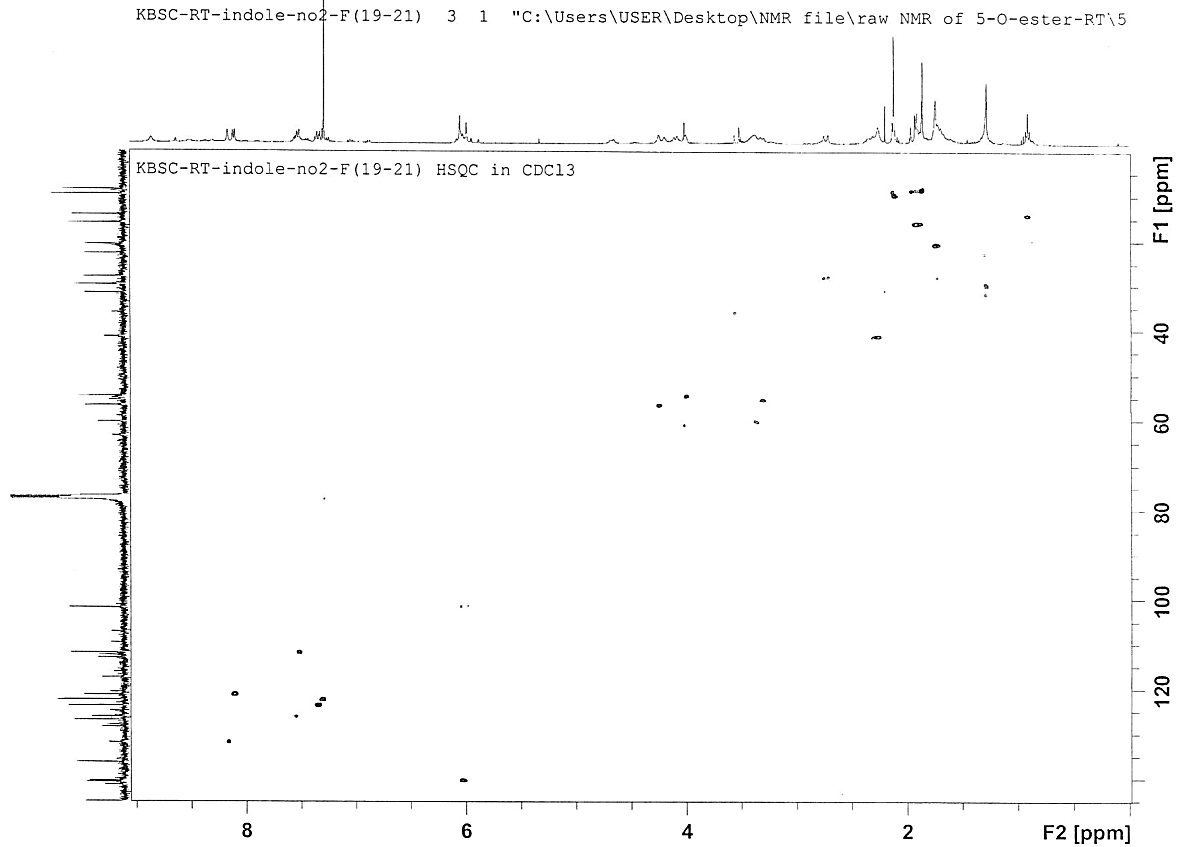


**Figure S63.** HSQC (400 MHz) spectrum of **3l** in CDCl_3_


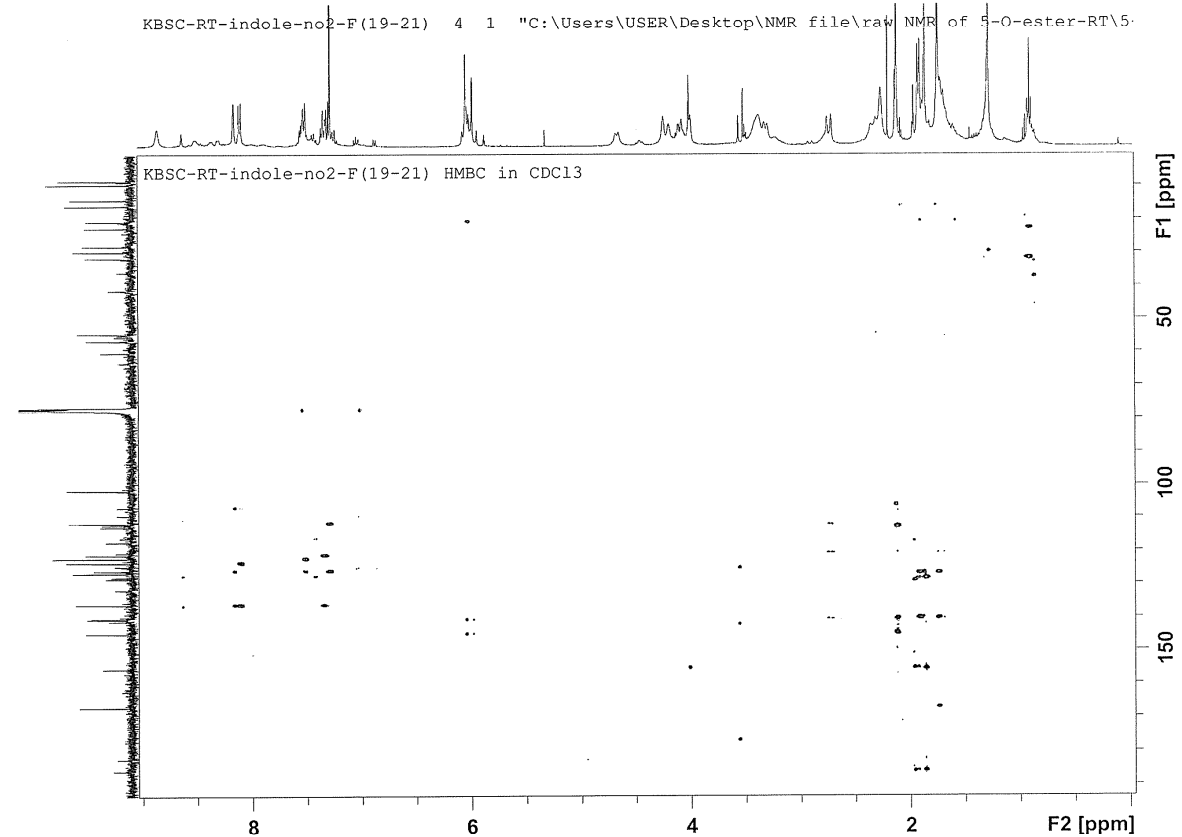


**Figure S64.** HMBC (400 MHz) spectrum of **3l** in CDCl_3_

# Physical and spectroscopic data of **3m**

*5-O-(2-furancarbonyl) ester derivative of renieramycin T,* **3m**: The title compound was synthesized from **2** (25.0 mg, 0.04 mmol), DMAP (13.3 mg, 0.11 mmol), EDCI (20.5 mg, 0.11 mmol) and 2-furancarboxylic acid (24.3 mg, 0.04 mmol) to afford **3m**; yield 63% (brsm); yellow amorphous powder; $[]_{D}^{25}$ −8.3 (*c* 0.41, CHCl_3_); ECD Δ*ε* (*c* 68.69 μM, methanol, 20 ^o^C) +1.0 (326), −6.2 (312), −13.5 (288), +13.4 (282), +31.1 (269), +16.4 (258), −1.6 (251), +24.9 (226), +5.2 (213), −30.4 (208), −25.3 (198) nm; IR (ATR) ν_max_ 2922, 2851, 1738, 1651, 1455, 1289, 1231, 1099, 953, 769 cm^−1^; ^1^H NMR (CDCl_3_, 400 MHz) δ 7.70 (1H, dd, *J* = 1.7, 0.8 Hz, 4'-H), 7.40 (1H, dd, *J* = 3.5, 0.8 Hz, 6'-H), 6.65 (1H, dd, *J* = 3.5, 1.7 Hz, 5'-H), 5.99 (1H, overlapped, 26-H), 5.98 (2H, dd, *J* = 27.6, 1.4 Hz, OCH_2_O), 4.65 (1H, dd, *J* = 11.6, 2.9 Hz, 22-H_α_), 4.20 (1H, br t, *J* = 3.4 Hz, 1-H), 4.18 (1H, br d, *J* = 2.0 Hz, 21-H), 4.05 (1H, dd, *J* = 11.6, 4.1 Hz, 22-H_β_), 4.03 (1H, overlapped, 11-H), 3.80 (3H, s, 17-OCH_3_), 3.46 (1H, overlapped, 13-H), 3.29 (1H, dt, *J* = 11.8, 2.2 Hz, 3-H), 2.78 (1H, overlapped, 14-H_α_), 2.59 (1H, dd, *J* = 15.2, 2.2 Hz, 4-H_α_), 2.36 (1H, br d, *J* = 20.6 Hz, 14-H_β_), 2.32 (3H, s, NCH_3_), 2.06 (3H, s, 6-CH_3_), 1.88 (3H, s, 16-CH_3_), 1.86 (3H, dq, *J* = 7.3, 1.5 Hz, 27-CH_3_), 1.67 (3H, dq, *J* = 1.6, 1.5 Hz, 28-CH_3_), 1.66 (1H, overlapped, 4-H_β_); ^13^C NMR (CDCl_3_, 100 MHz) δ 185.7 (C-15), 182.4 (C-18), 167.0 (C-24), 156.1 (C-1'), 155.5 (C-17), 147.4 (C-4'), 145.0 (C-7), 143.3 (C-2'), 141.3 (C-20), 140.9 (C-8), 140.4 (C-26), 139.7 (C-5), 135.1 (C-19), 128.4 (C-16), 126.6 (C-25), 120.3 (C-6), 119.6 (C-6'), 117.1 (21-CN), 112.4 (C-5'), 112.4 (C-10), 112.4 (C-9), 101.8 (OCH_2_O), 62.6 (C-22), 60.5 (17-OCH_3_), 58.5 (C-21), 56.6 (C-1), 55.0 (C-3), 54.5 (C-11), 54.5 (C-13), 41.3 (NCH_3_), 28.0 (C-4), 21.1 (C-14), 20.5 (28-CH_3_), 15.9 (27-CH_3_), 9.5 (6-CH_3_), 8.5 (16-CH_3_); HRESIMS *m/z* 670.2394 ([M+H]^+^, calculated for C_36_H_36_N_3_O_10_, 670.2395).


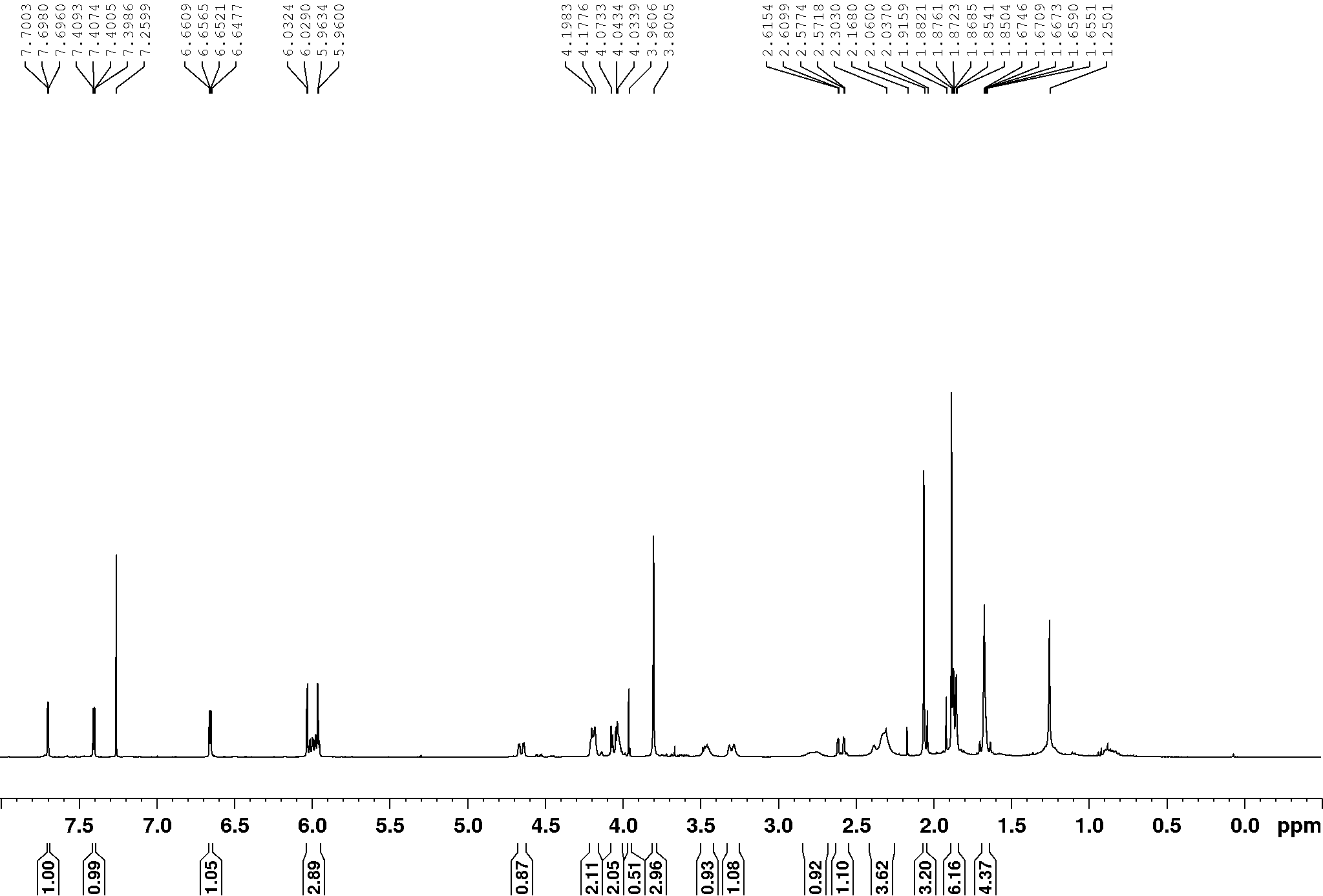


g

g

g: grease

**Figure S65.** ^1^H NMR (400 MHz) spectrum of **3m** in CDCl_3_


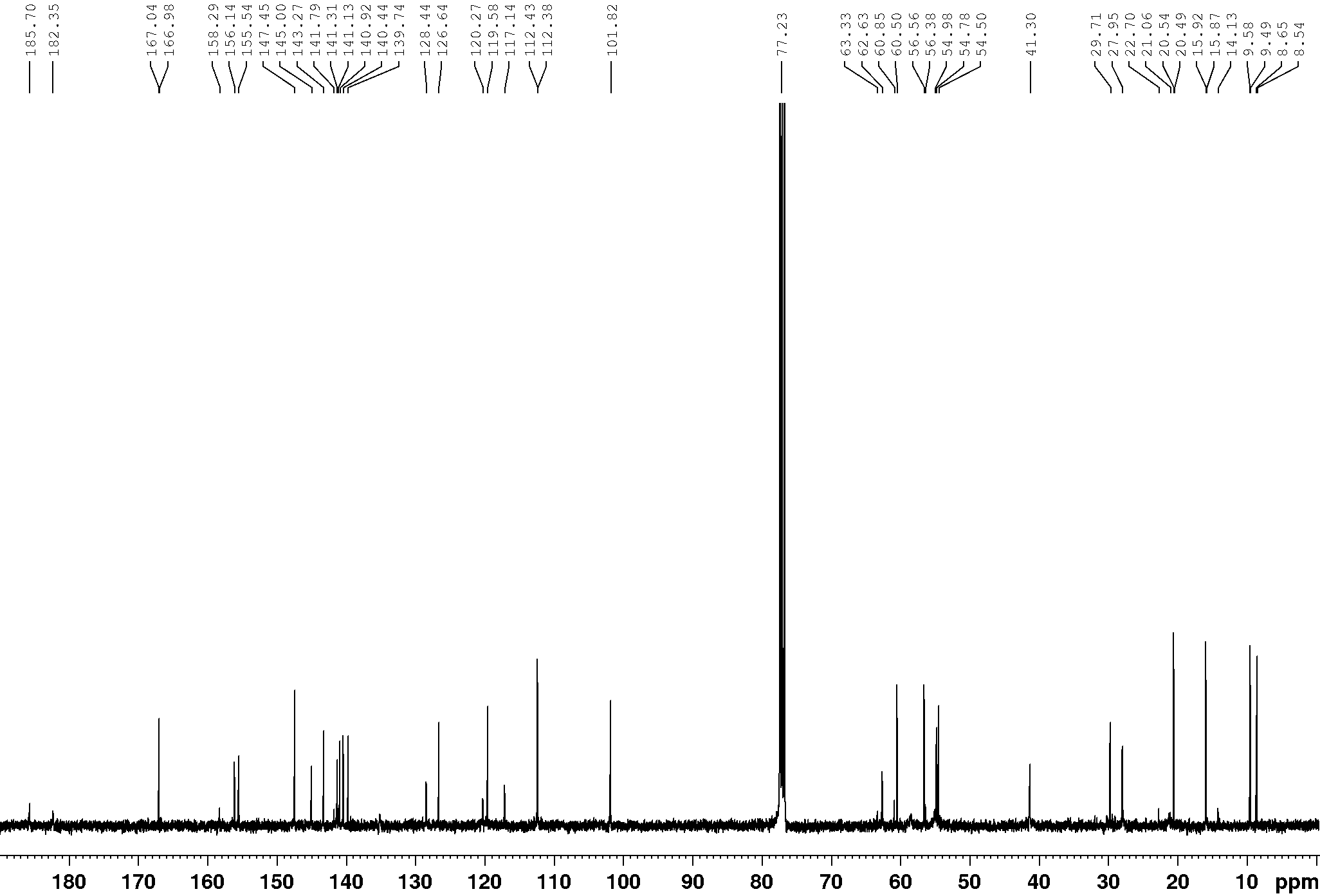


g

g: grease

**Figure S66.** ^13^C NMR (100 MHz) spectrum of **3m** in CDCl_3_


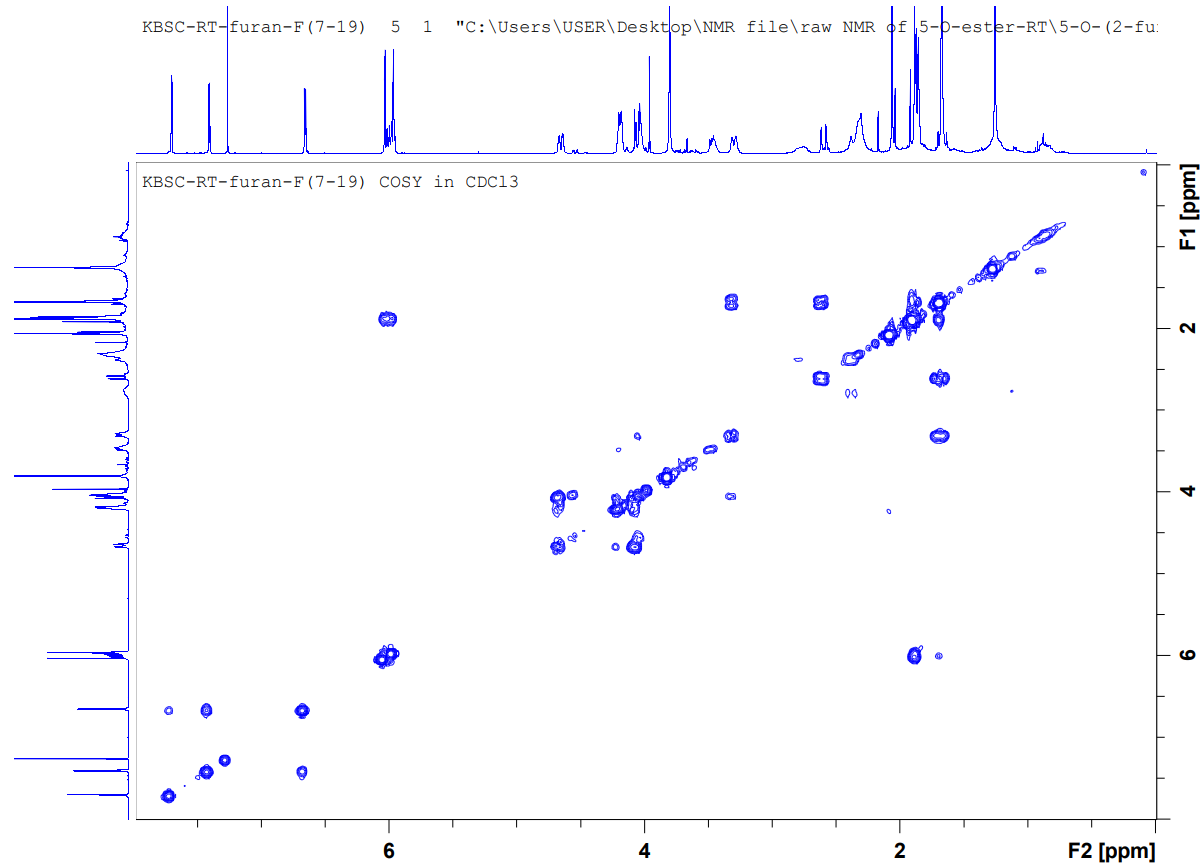


**Figure S67.** COSY (400 MHz) spectrum of **3m** in CDCl_3_


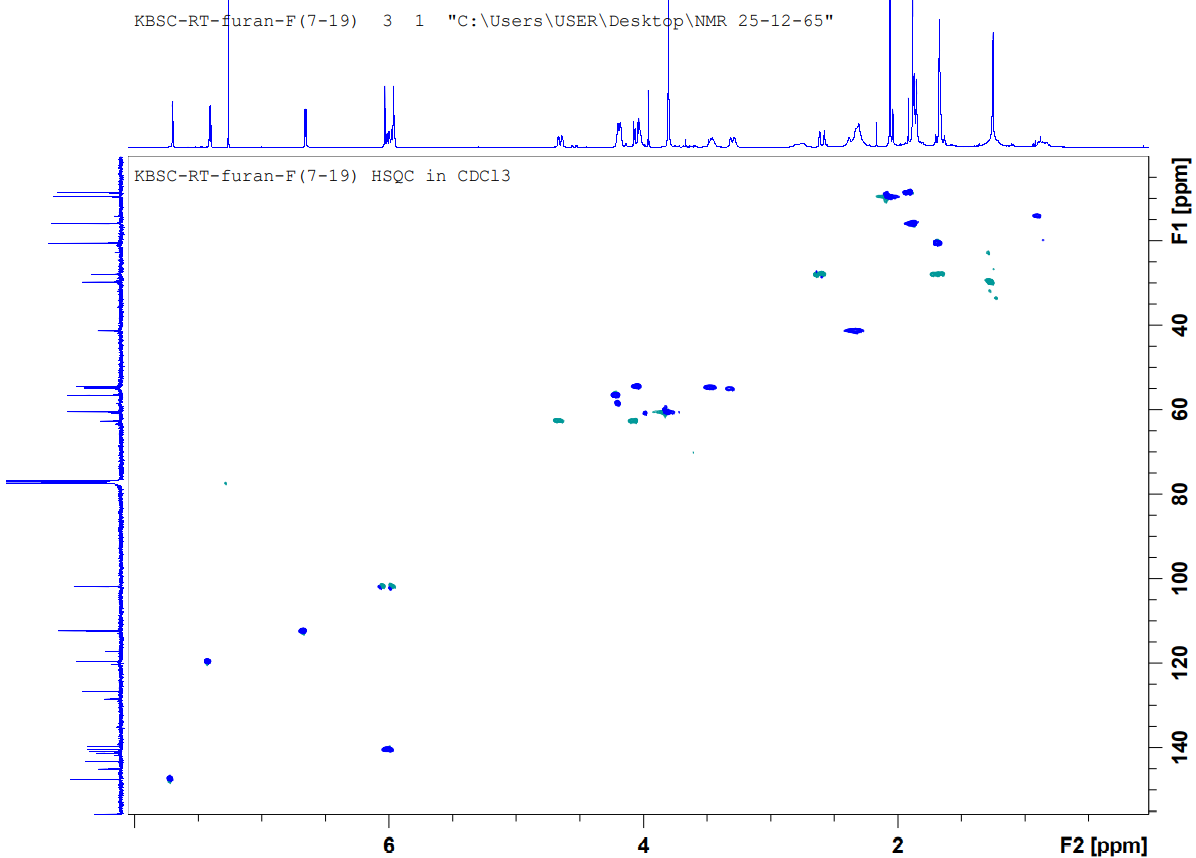


**Figure S68.** HSQC (400 MHz) spectrum of **3m** in CDCl_3_


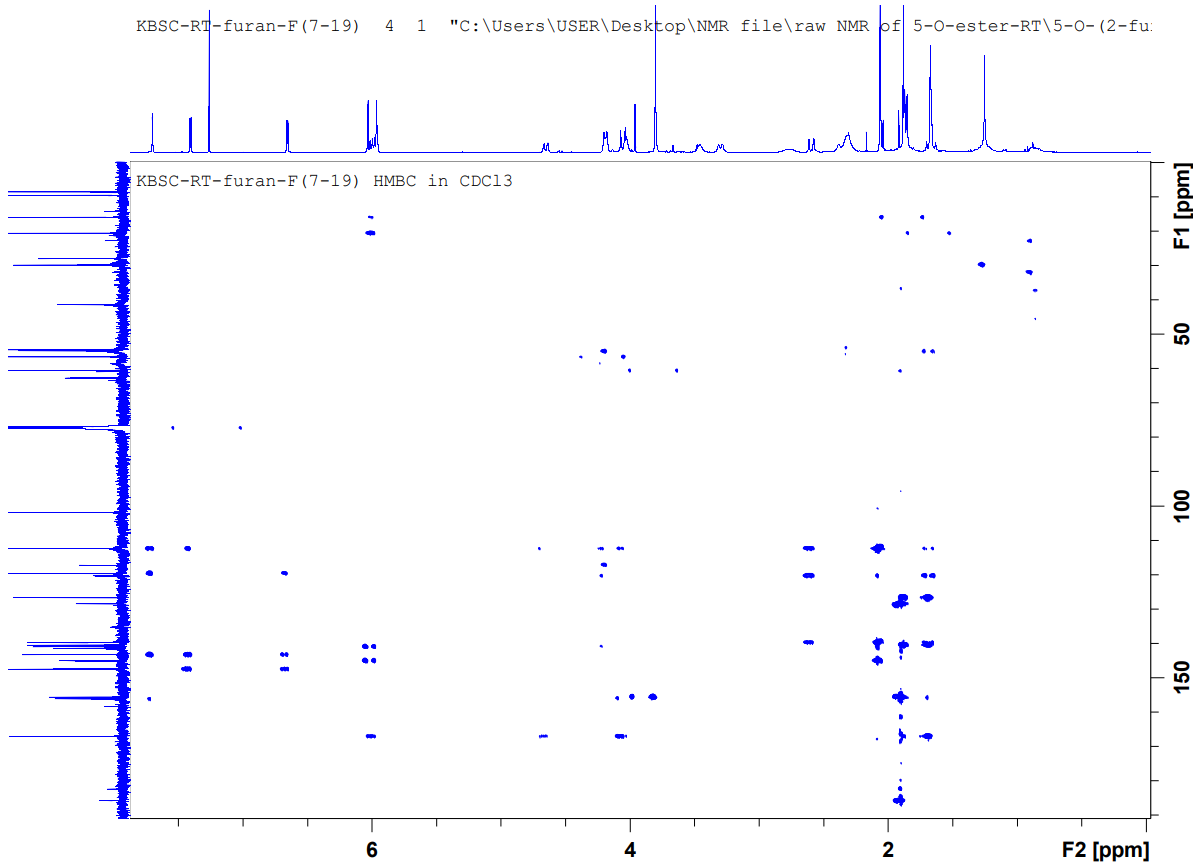


**Figure S69.** HMBC (400 MHz) spectrum of **3m** in CDCl_3_

# Physical and spectroscopic data of **3n**

*5-O-(2-thiophenecarbonyl) ester derivative of renieramycin T,* **3n**: The title compound was synthesized from **2** (25.0 mg, 0.04 mmol), DMAP (13.3 mg, 0.11 mmol), EDCI (20.5 mg, 0.11 mmol) and 2-thiophenecarboxylic acid (27.8 mg, 0.22 mmol) to afford **3n**; yield 45% (brsm); yellow amorphous powder; $[]_{D}^{25}$ −102.8 (*c* 0.10, CHCl_3_); ECD Δ*ε* (*c* 17.50 μM, methanol, 20 ^o^C) −2.1 (301), −4.9 (291), +26.6 (261), −18.5 (221), +7.6 (213), +10.7 (208), −12.3 (204), −0.3 (202), +14.0 (199), −8.3 (196), +17.9 (193) nm; IR (ATR) ν_max_ 2924, 1715, 1651, 1414, 1232, 1146, 1084, 953, 733 cm^−1^; ^1^H NMR (CDCl_3_, 400 MHz) δ 7.96 (1H, dd, *J* = 3.8, 1.2 Hz, 4'-H), 7.74 (1H, dd, *J* = 4.9, 1.2 Hz, 6'-H), 7.25 (1H, dd, *J* = 4.9, 3.8 Hz, 5'-H), 6.02 (2H, dd, *J* = 27.8, 1.4 Hz, OCH_2_O), 6.01 (1H, overlapped, 26-H), 4.65 (1H, dd, *J* = 11.4, 2.3 Hz, 22-H_α_), 4.23 (1H, dd, *J* = 4.0, 2.3 Hz, 1-H), 4.21 (1H, d, *J* = 2.2 Hz, 21-H), 4.08 (1H, dd, *J* = 11.4, 4.0 Hz, 22-H_β_), 4.07 (1H, overlapped, 11-H), 3.79 (3H, s, 17-OCH_3_), 3.49 (1H, overlapped, 13-H), 3.35 (1H, dt, *J* = 12.0, 2.2 Hz, 3-H), 2.79 (1H, overlapped, 14-H_α_), 2.66 (1H, dd, *J* = 15.3, 2.2 Hz, 4-H_α_), 2.39 (1H, overlapped, 14-H_β_), 2.35 (3H, s, NCH_3_), 2.09 (3H, s, 6-CH_3_), 1.90 (3H, s, 16-CH_3_), 1.89 (3H, dq, *J* = 7.1, 1.5 Hz, 27-CH_3_), 1.69 (3H, ddd, *J* = 9.0, 1.5, 1.4 Hz, 28-CH_3_), 1.71 (1H, overlapped, 4-H_β_); ^13^C NMR (CDCl_3_, 100 MHz) δ 185.7 (C-15), 182.3 (C-18), 167.0 (C-24), 159.8 (C-1'), 155.5 (C-17), 145.0 (C-7), 141.8 (C-20), 140.9 (C-8), 140.4 (C-26), 140.2 (C-5), 135.0 (C-19), 134.7 (C-4'), 133.8 (C-6'), 131.9 (C-2'), 129.0 (C-16), 128.2 (C-5'), 126.7 (C-25), 120.3 (C-6), 117.1 (21-CN), 112.5 (C-10), 112.2 (C-9), 101.8 (OCH_2_O), 62.9 (C-22), 60.5 (17-OCH_3_), 58.6 (C-21), 56.5 (C-1), 55.2 (C-3), 54.8 (C-13), 54.5 (C-11), 41.3 (NCH_3_), 28.0 (C-4), 21.1 (C-14), 20.5 (28-CH_3_), 15.9 (27-CH_3_), 9.5 (6-CH_3_), 8.5 (16-CH_3_); HRESIMS *m/z* 686.2167 ([M+H]^+^, calculated for C_36_H_36_N_3_O_9_S, 686.2167).


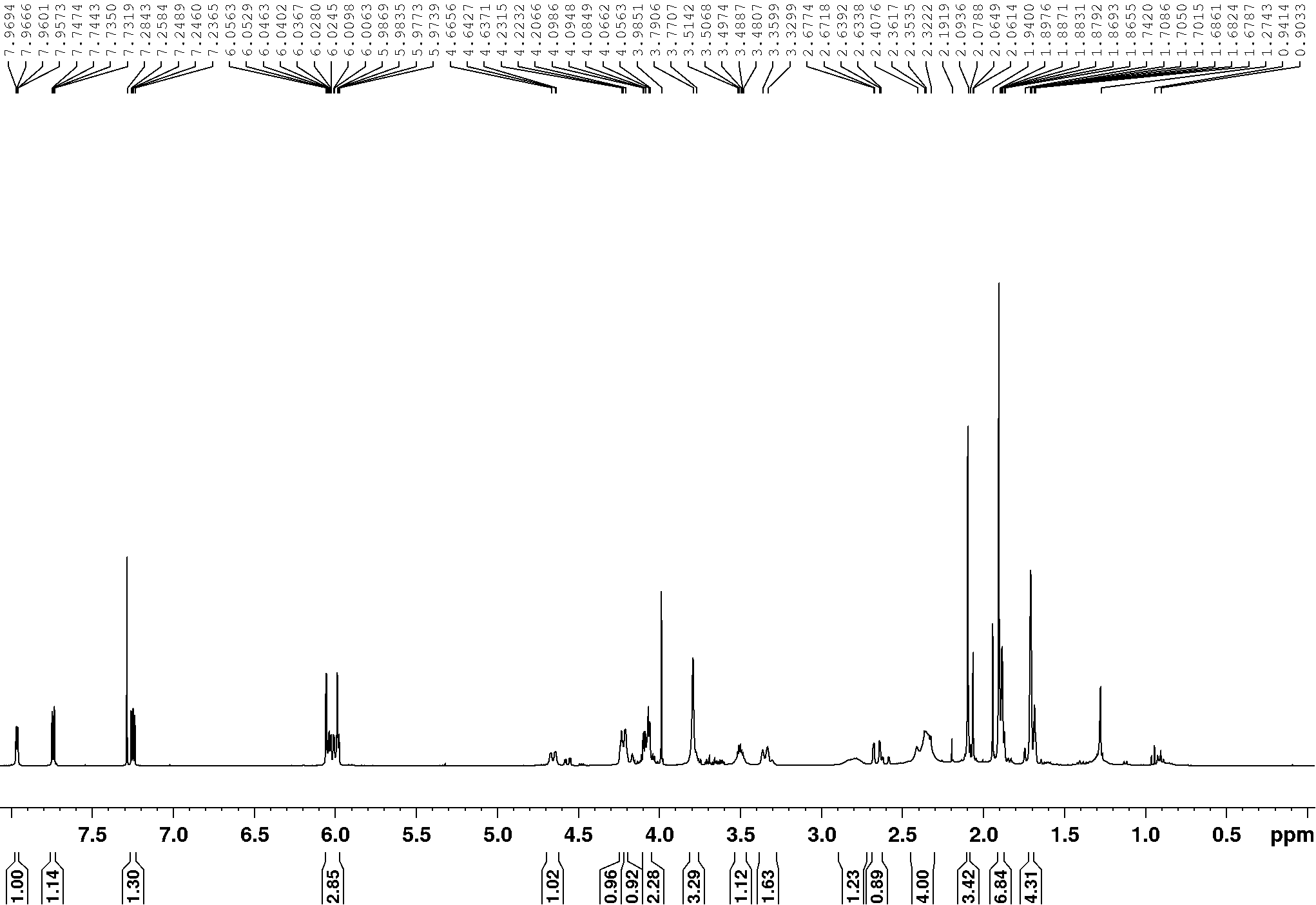


g: grease

g

g

**Figure S70.** ^1^H NMR (400 MHz) spectrum of **3n** in CDCl_3_


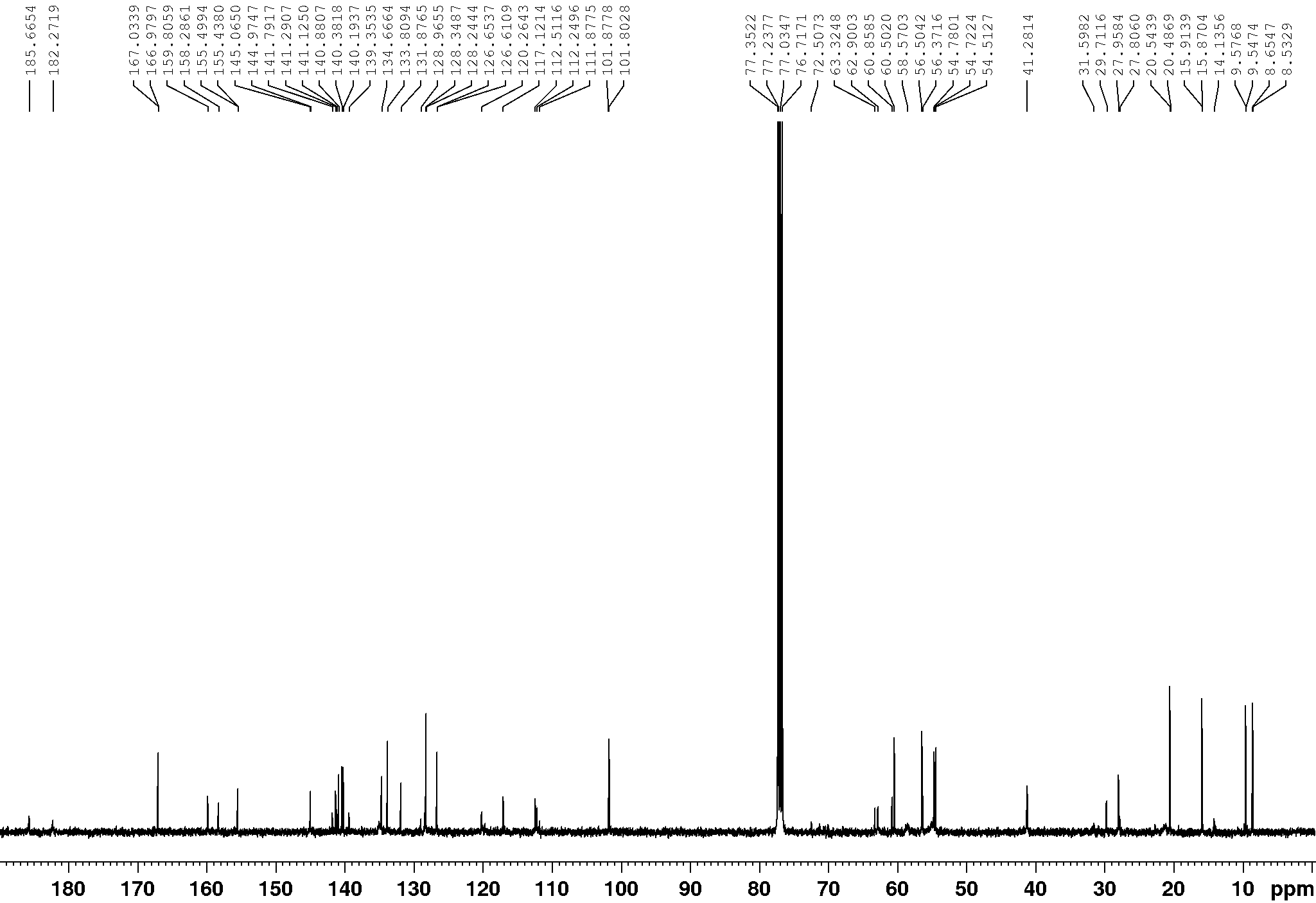


g

g: grease

**Figure S71.** ^13^C NMR (100 MHz) spectrum of **3n** in CDCl_3_


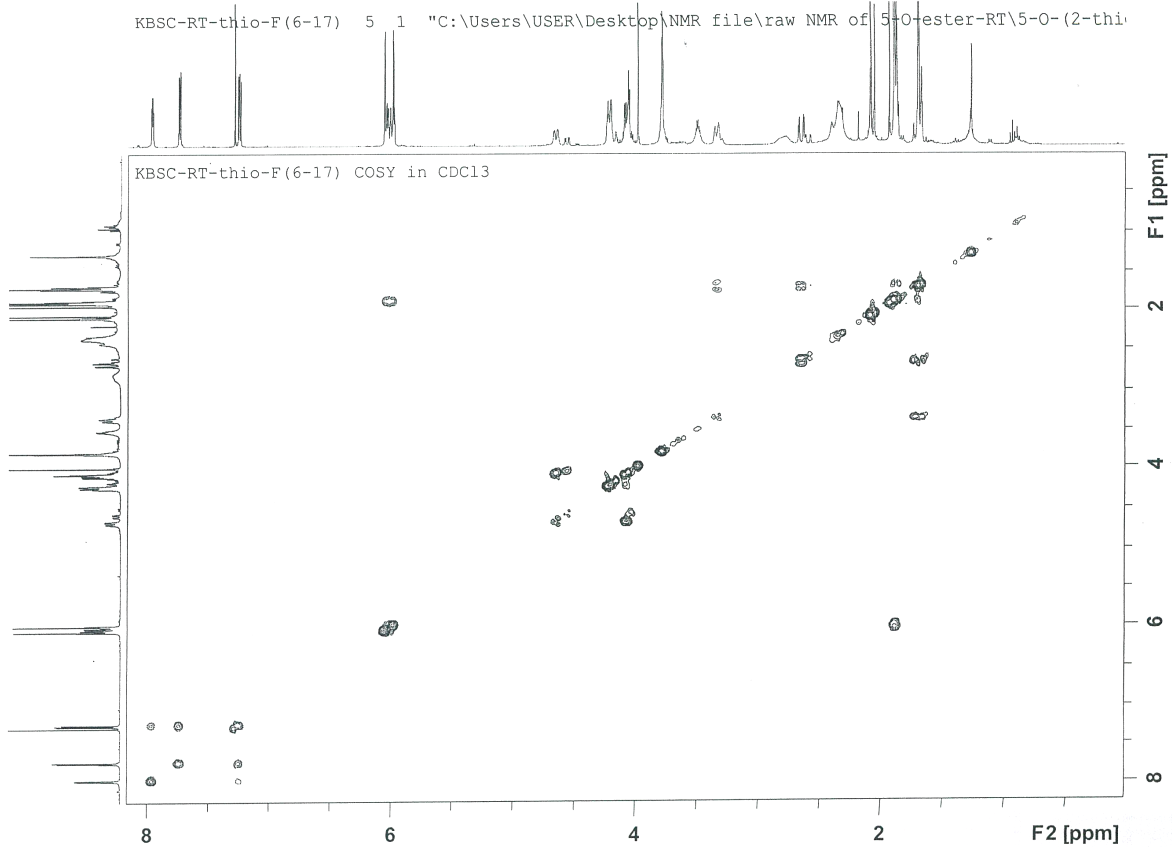


**Figure S72.** COSY (400 MHz) spectrum of **3n** in CDCl_3_


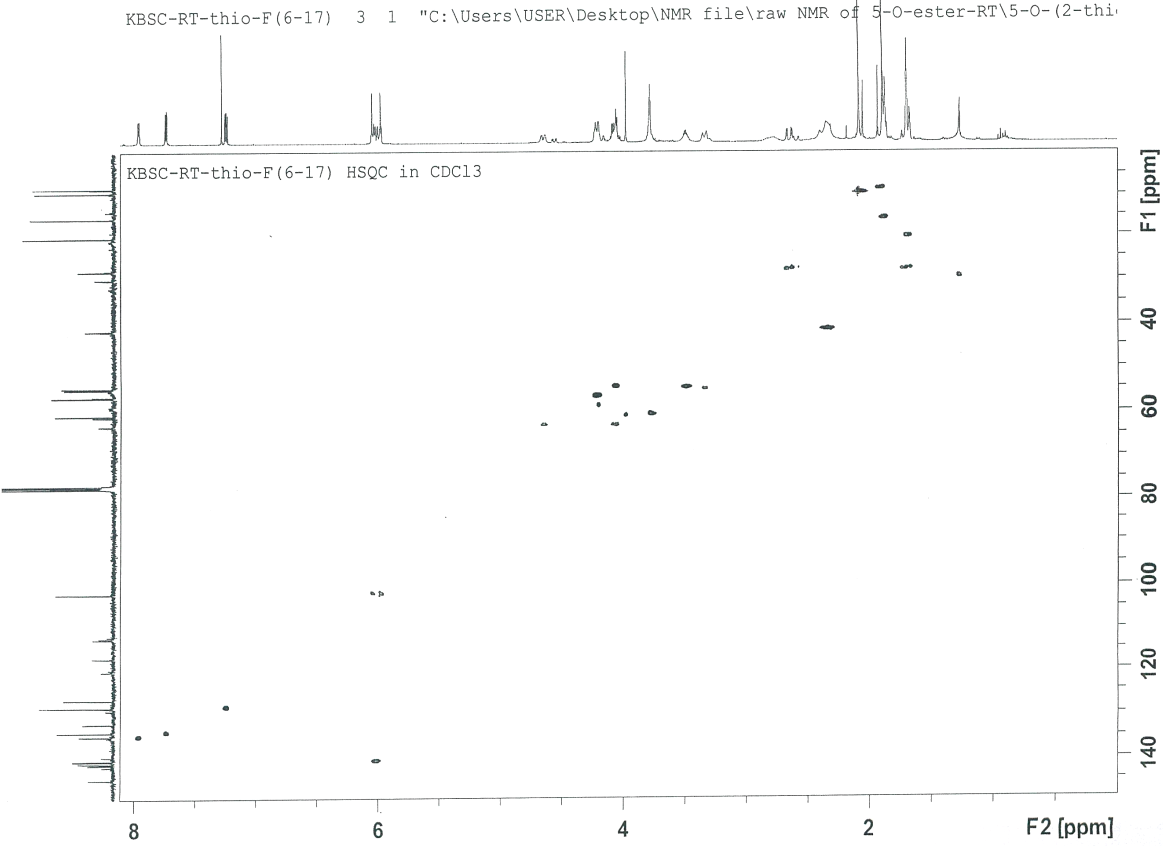


**Figure S73.** HSQC (400 MHz) spectrum of **3n** in CDCl_3_


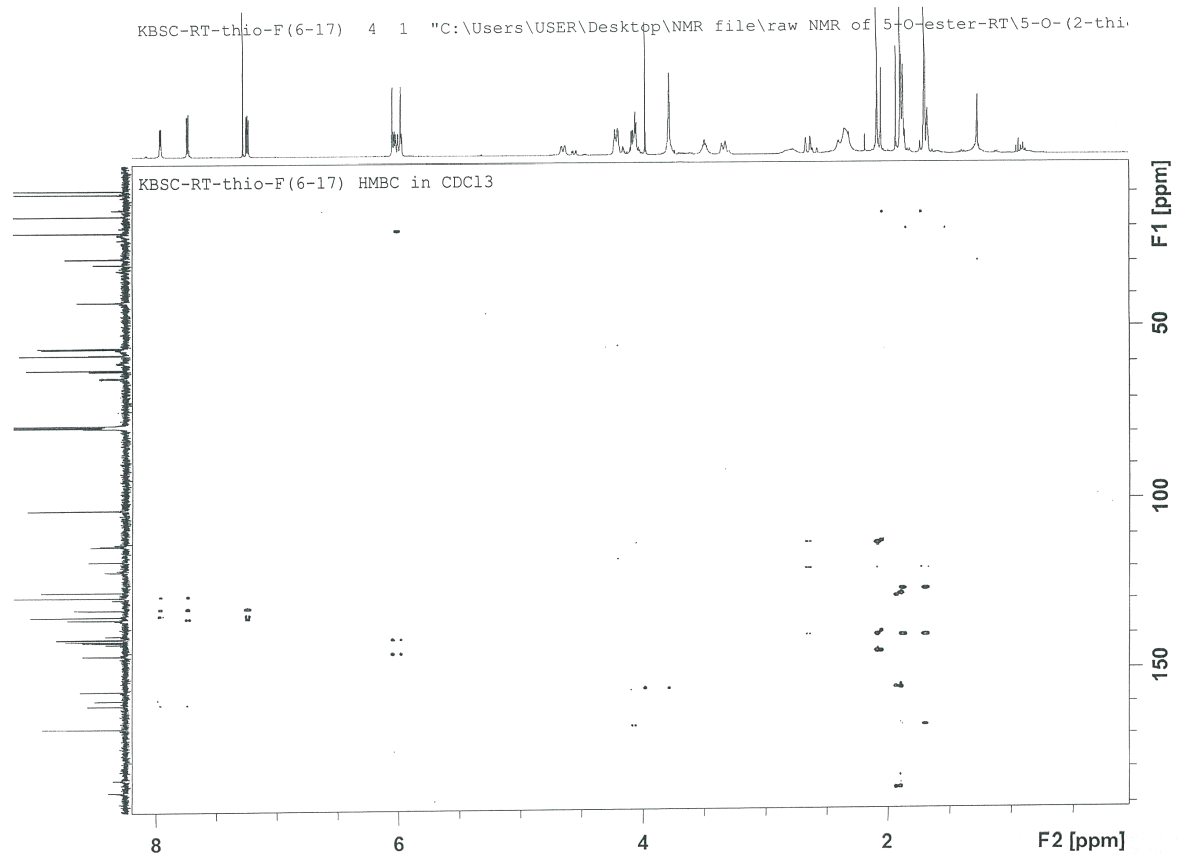


**Figure S74.** HMBC (400 MHz) spectrum of **3n** in CDCl_3_

# Physical and spectroscopic data of **3o**

*5-O-(2-pyrrolecarbonyl) ester derivative of renieramycin T,* **3o**: The title compound was synthesized from **2** (25.0 mg, 0.04 mmol), DMAP (13.3 mg, 0.11 mmol), EDCI (20.5 mg, 0.11 mmol) and pyrrole-2-carboxylic acid (24.1 mg, 0.22 mmol) to afford **3o**; yield 53% (brsm); yellow amorphous powder; $[]_{D}^{25}$ −75.1 (*c* 0.11, CHCl_3_); ECD Δ*ε* (*c* 65.80 μM, methanol, 20 ^o^C) −1.5 (308), −4.8 (291), +22.4 (269), −6.8 (223), +4.3 (211), +15.0 (199), −18.8 (197), −15.8 (194), +3.3 (192) nm; IR (ATR) ν_max_ 2923, 1714, 1651, 1447, 1404, 1233, 1147, 1086, 953, 753 cm^−1^; ^1^H NMR (CDCl_3_, 400 MHz) δ 9.24 (1H, br s, 3'-NH), 7.16 (1H, ddd, *J* = 3.8, 2.6, 1.4 Hz, 4'-H), 7.11 (1H, ddd, *J* = 3.8, 2.6, 1.4 Hz, 6'-H), 6.42 (1H, dt, *J* = 3.8, 2.6 Hz, 5'-H), 6.02 (2H, dd, *J* = 26.4, 1.3 Hz, OCH_2_O), 6.01 (1H, overlapped, 26-H), 4.62 (1H, dd, *J* = 11.6, 2.6 Hz, 22-H_α_), 4.25 (1H, overlapped, 1-H), 4.24 (1H, overlapped, 21-H), 4.12 (1H, overlapped, 11-H), 4.06 (1H, dd, *J* = 11.6, 4.2 Hz, 22-H_β_), 3.78 (3H, s, 17-OCH_3_), 3.57 (1H, br s, 13-H), 3.39 (1H, br d, *J* = 11.7 Hz, 3-H), 2.85 (1H, overlapped, 14-H_α_), 2.69 (1H, dd, *J* = 15.2, 1.9 Hz, 4-H_α_), 2.46 (1H, overlapped,14-H_β_), 2.41 (3H, s, NCH_3_), 2.08 (3H, s, 6-CH_3_), 1.91 (3H, s, 16-CH_3_), 1.89 (3H, dq, *J* = 7.3, 1.4 Hz, 27-CH_3_), 1.71 (3H, dq, *J* = 1.4, 1.4 Hz, 28-CH_3_), 1.68 (1H, overlapped, 4-H_β_); ^13^C NMR (CDCl_3_, 100 MHz) δ 185.6 (C-15), 182.1 (C-18), 167.0 (C-24), 158.6 (C-1'), 155.4 (C-17), 145.0 (C-7), 141.2 (C-20), 140.8 (C-8), 140.4 (C-26), 140.1 (C-5), 134.7 (C-19), 128.4 (C-16), 126.6 (C-25), 124.1 (C-6'), 121.1 (C-2'), 120.2 (C-6), 117.1 (C-4'), 116.9 (21-CN), 112.8 (C-10), 112.1 (C-9), 111.2 (C-5'), 101.8 (OCH_2_O), 63.2 (C-22), 60.4 (17-OCH_3_), 58.5 (C-21), 56.5 (C-1), 55.3 (C-3), 54.8 (C-13), 54.6 (C-11), 41.2 (NCH_3_), 27.8 (C-4), 20.7 (C-14), 20.5 (28-CH_3_), 15.9 (27-CH_3_), 9.6 (6-CH_3_), 8.6 (16-CH_3_); HRESIMS *m/z* 669.2555 ([M+H]^+^, calculated for C_36_H_37_N_4_O_9_, 669.2555).


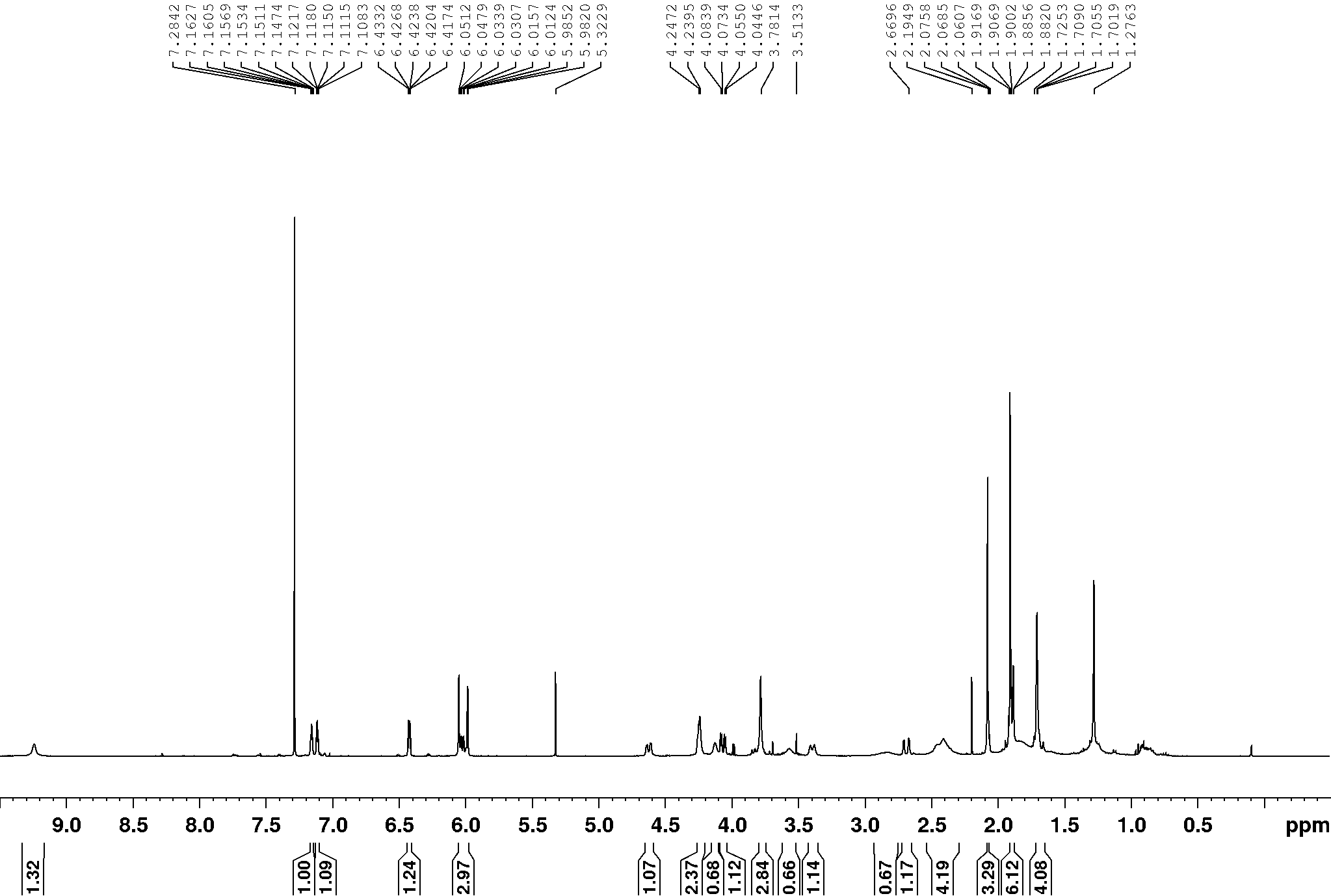


g, h

g, h

g: grease, h: hexane

dichloromethane

**Figure S75.** ^1^H NMR (400 MHz) spectrum of **3o** in CDCl_3_


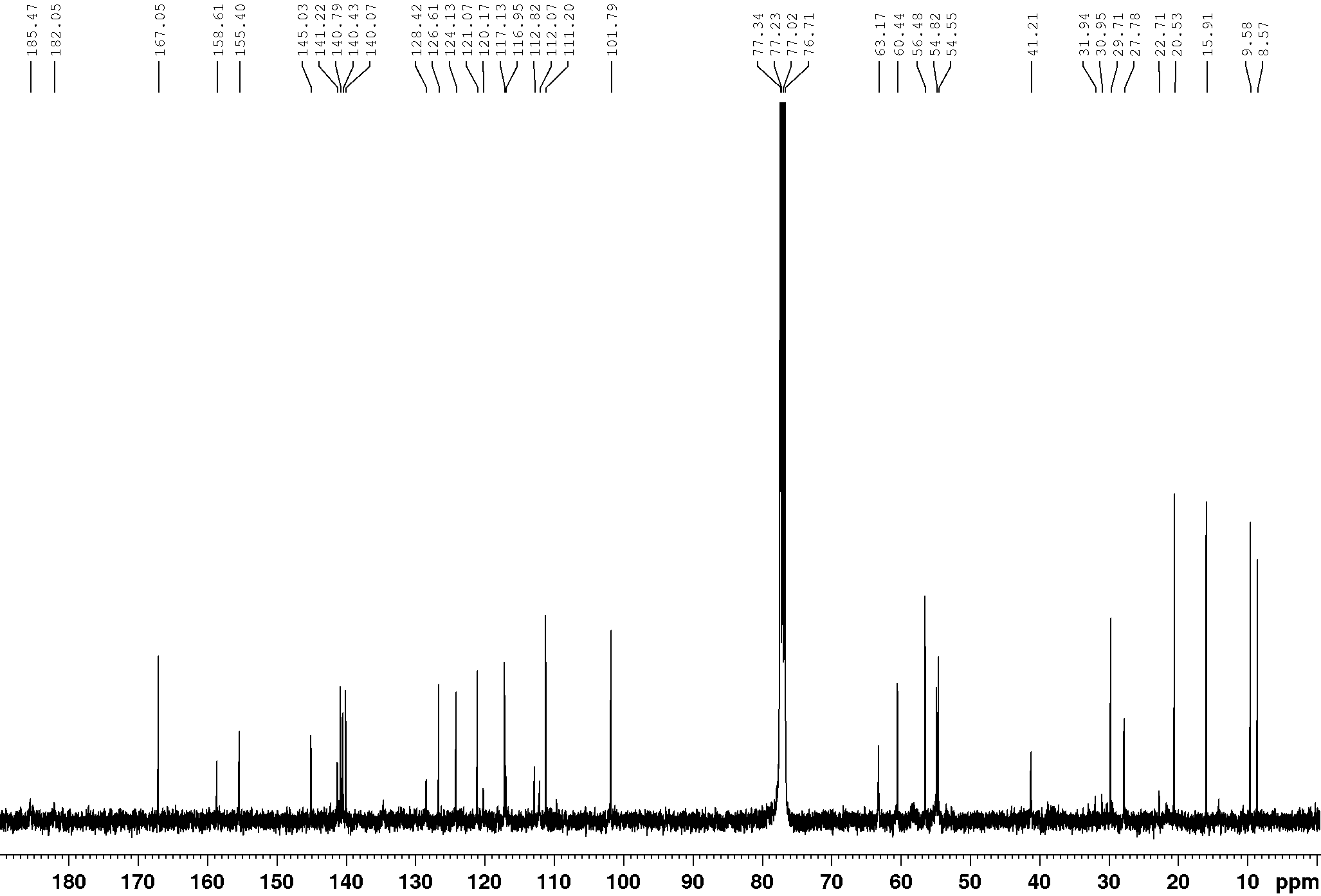


h

g

h

h

g: grease, h: hexane

**Figure S76.** ^13^C NMR (100 MHz) spectrum of **3o** in CDCl_3_


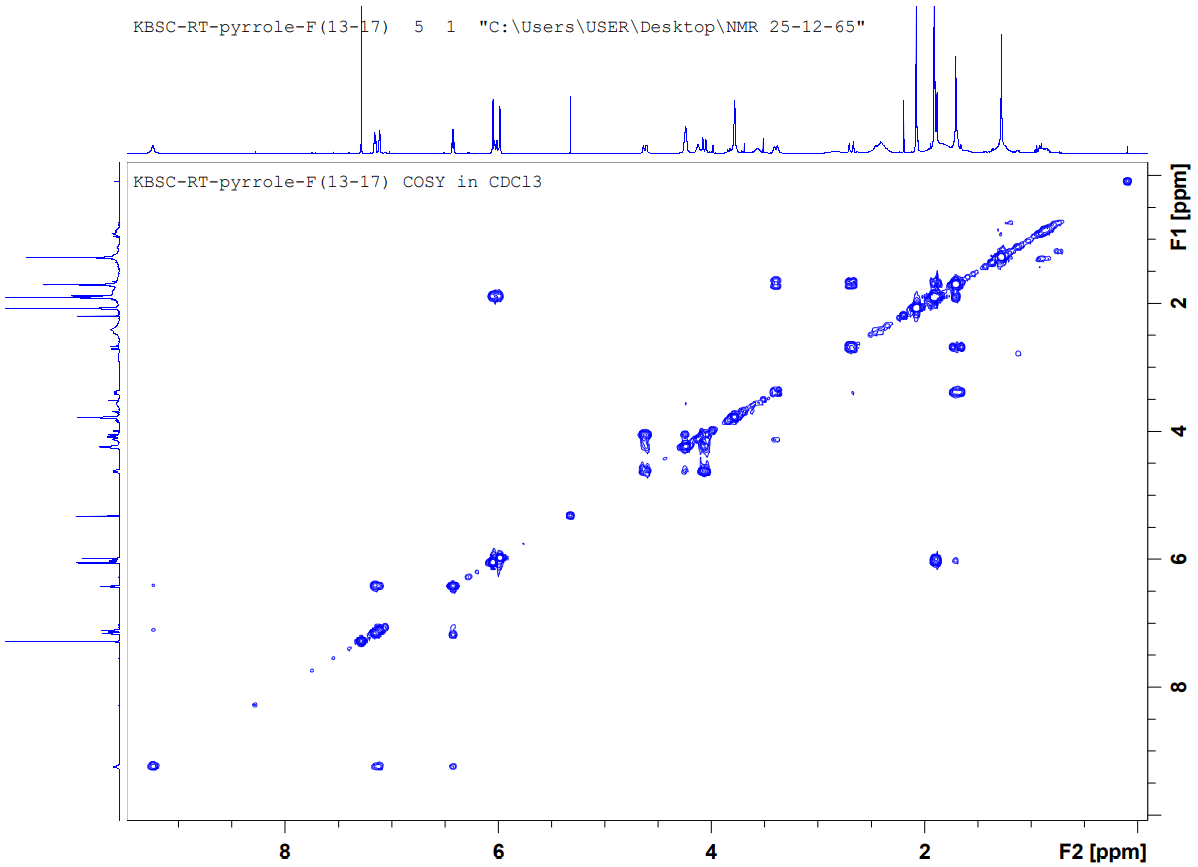


**Figure S77.** COSY (400 MHz) spectrum of **3o** in CDCl_3_


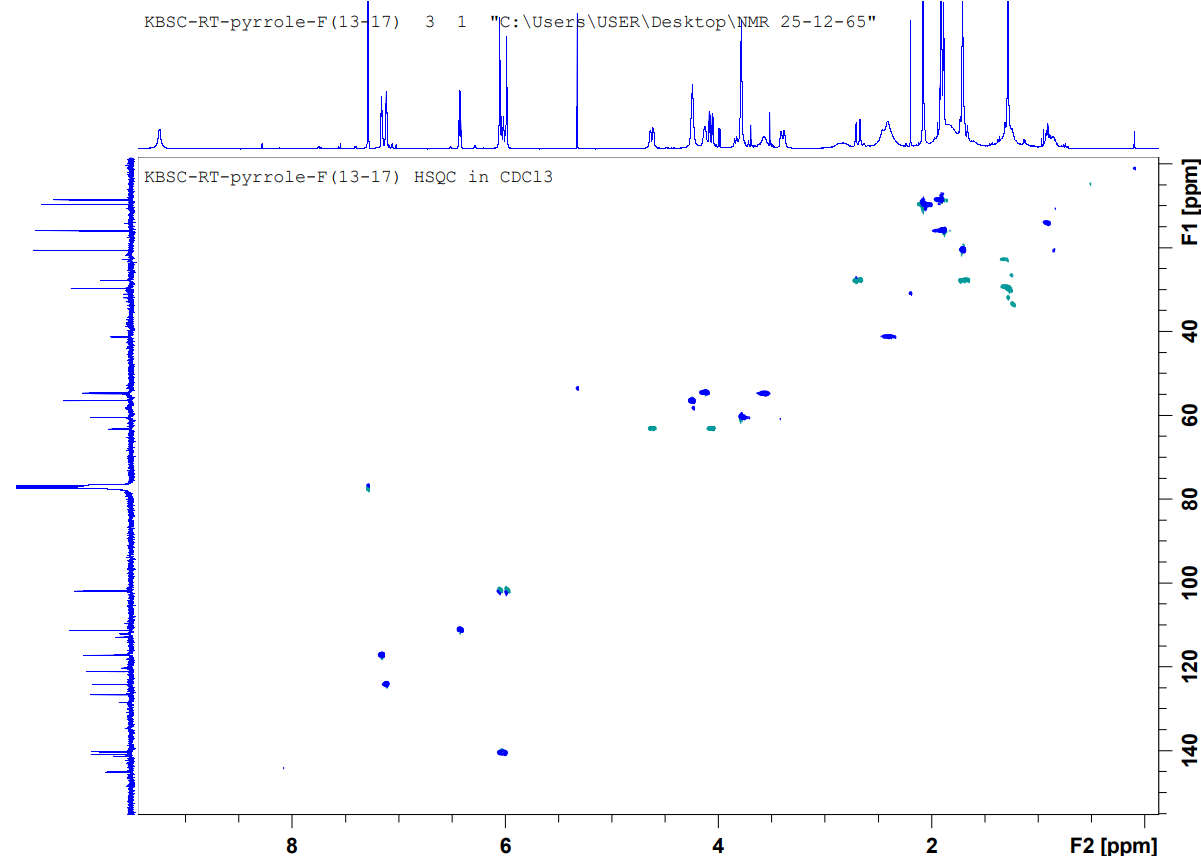


**Figure S78.** HSQC (400 MHz) spectrum of **3o** in CDCl_3_


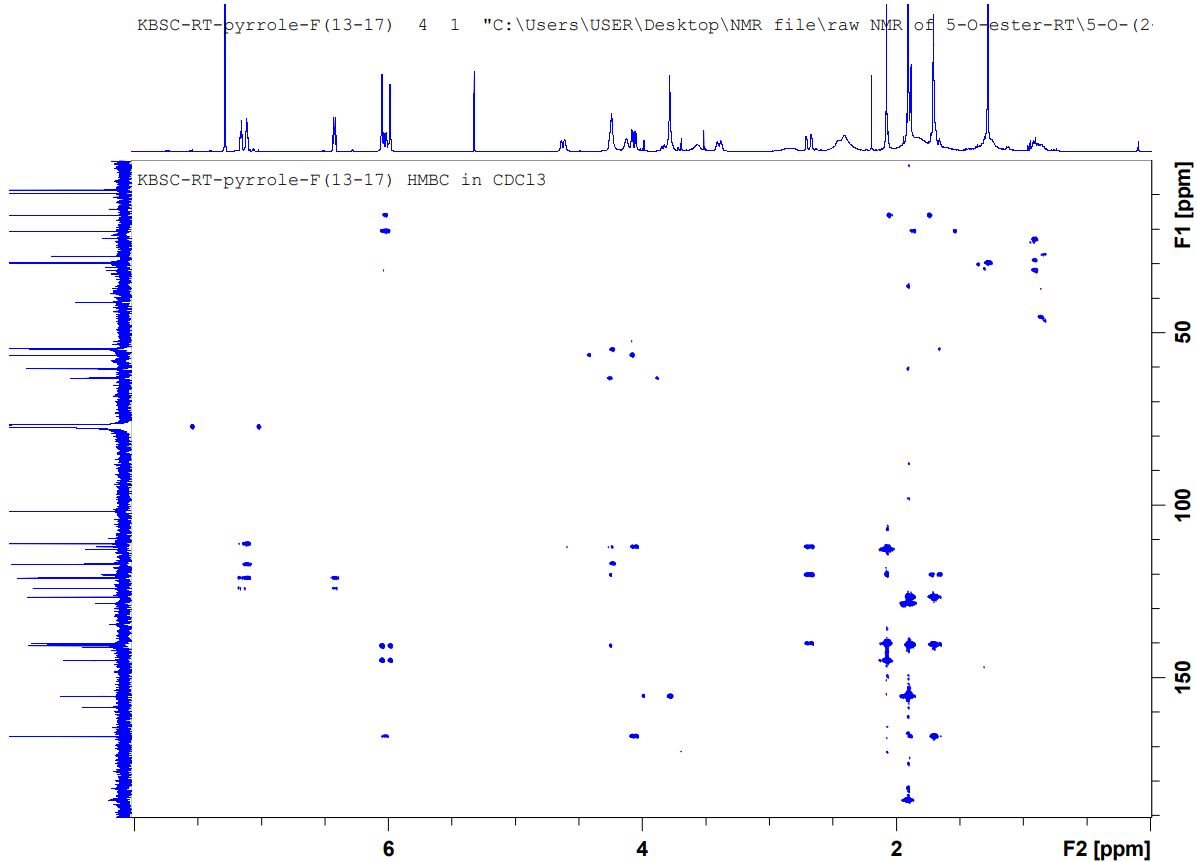


**Figure S79.** HMBC (400 MHz) spectrum of **3o** in CDCl_3_

# Physical and spectroscopic data of **4a**

*7-O-(propionyl) ester derivative of renieramycin S* **4a**: The title compound was synthesized from **2** (21.9 mg, 0.04 mmol), DMAP (11.6 mg, 0.10 mmol), EDCI (18.0 mg, 0.10 mmol) and propionyl chloride (0.02 mL, 0.19 mmol) to afford **4a**; yield 52% (brsm); yellow amorphous powder;$[]_{D}^{25}$ +71.0 (*c* 0.10, CHCl_3_); ECD Δ*ε* (*c* 64.76 μM, methanol, 20 ^o^C) −2.4 (301), −6.1 (279), +15.6 (253), −7.0 (224), +2.1 (209), +11.6 (200), −3.2 (198), −13.9 (194), −0.3 (192) nm; IR (ATR) ν_max_ 2921, 2851, 1770, 1714, 1651, 1456, 1375, 1230, 1146, 954, 769 cm^−1^; ^1^H NMR (CDCl_3_, 400 MHz) δ 5.96 (1H, q, *J* = 7.2 Hz, 26-H), 4.72 (1H, dd, *J* = 11.6, 2.4 Hz, 22-H_α_), 4.09 (1H, d, *J* = 2.4 Hz, 21-H), 4.01 (1H, d, *J* = 2.8 Hz, 11-H), 3.98 (1H, d, *J* = 2.4 Hz, 1-H), 3.96 (3H, s, 17-OCH_3_), 3.92 (1H, dd, *J* = 11.6, 2.0 Hz, 22-H_β_), 3.39 (1H, br d, *J* = 7.2 Hz, 13-H), 3.14 (1H, dt, *J* = 11.6, 2.8 Hz, 3-H), 2.91 (1H, dd, *J* = 17.6, 2.4 Hz, 4-H_α_), 2.74 (1H, dd, *J* = 21.2, 7.2 Hz, 14-H_α_), 2.65 (2H, q, *J* = 7.6 Hz, 2'-H), 2.32 (1H, d, *J* = 21.2 Hz, 14-H_β_), 2.28 (3H, s, NCH_3_), 1.94 (3H, s, 6-CH_3_), 1.90 (3H, s, 16-CH_3_), 1.83 (3H, d, *J* = 7.2 Hz, 27-CH_3_), 1.58 (3H, s, 28-CH_3_), 1.42 (1H, overlapped, 4-H_β_), 1.25 (3H, overlapped, 3'-CH_3_); ^13^C NMR (CDCl_3_, 100 MHz) δ 185.7 (C-15), 184.5 (C-5), 182.5 (C-18), 177.8 (C-8), 171.2 (C-1'), 166.4 (C-24), 155.2 (C-17), 149.0 (C-7), 142.2 (C-20), 142.0 (C-10), 140.7 (C-26), 136.4 (C-9), 134.8 (C-19), 133.2 (C-6), 128.7 (C-16), 126.2 (C-25), 116.8 (21-CN), 61.5 (C-22), 60.9 (17-OCH_3_), 58.2 (C-21), 56.3 (C-1), 54.6 (C-13), 54.2 (C-11), 54.0 (C-3), 41.5 (NCH_3_), 27.1 (C-2'), 25.6 (C-4), 21.0 (C-14), 20.4 (28-CH_3_), 15.8 (27-CH_3_), 9.4 (6-CH_3_), 8.9 (3'-CH_3_), 8.6 (16-CH_3_); HRESIMS *m/z* 618.2451 ([M+H]^+^, calculated for C_33_H_36_N_3_O_9_, 618.2446).


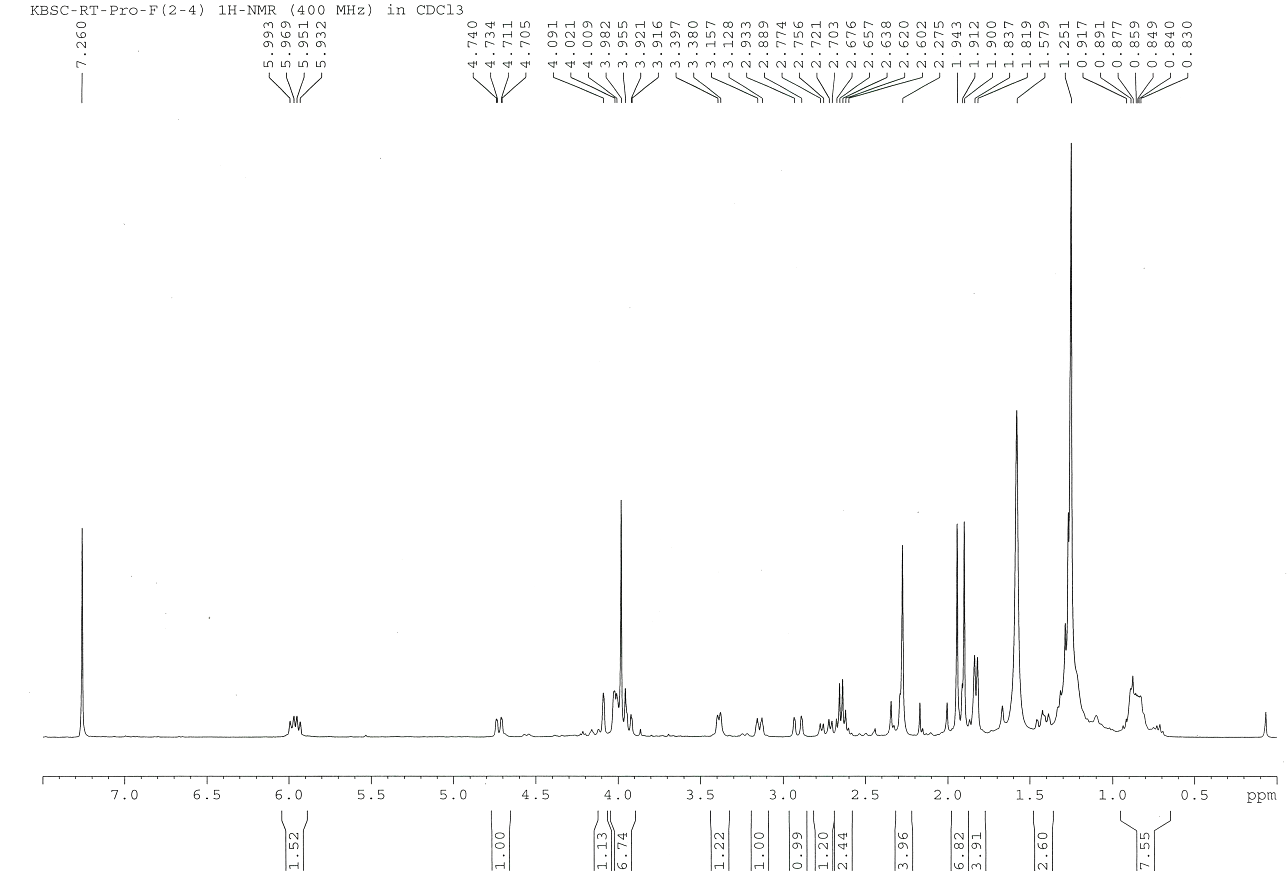


g: grease, h: hexane

g, h

g, h

**Figure S80.** ^1^H NMR (400 MHz) spectrum of **4a** in CDCl_3_


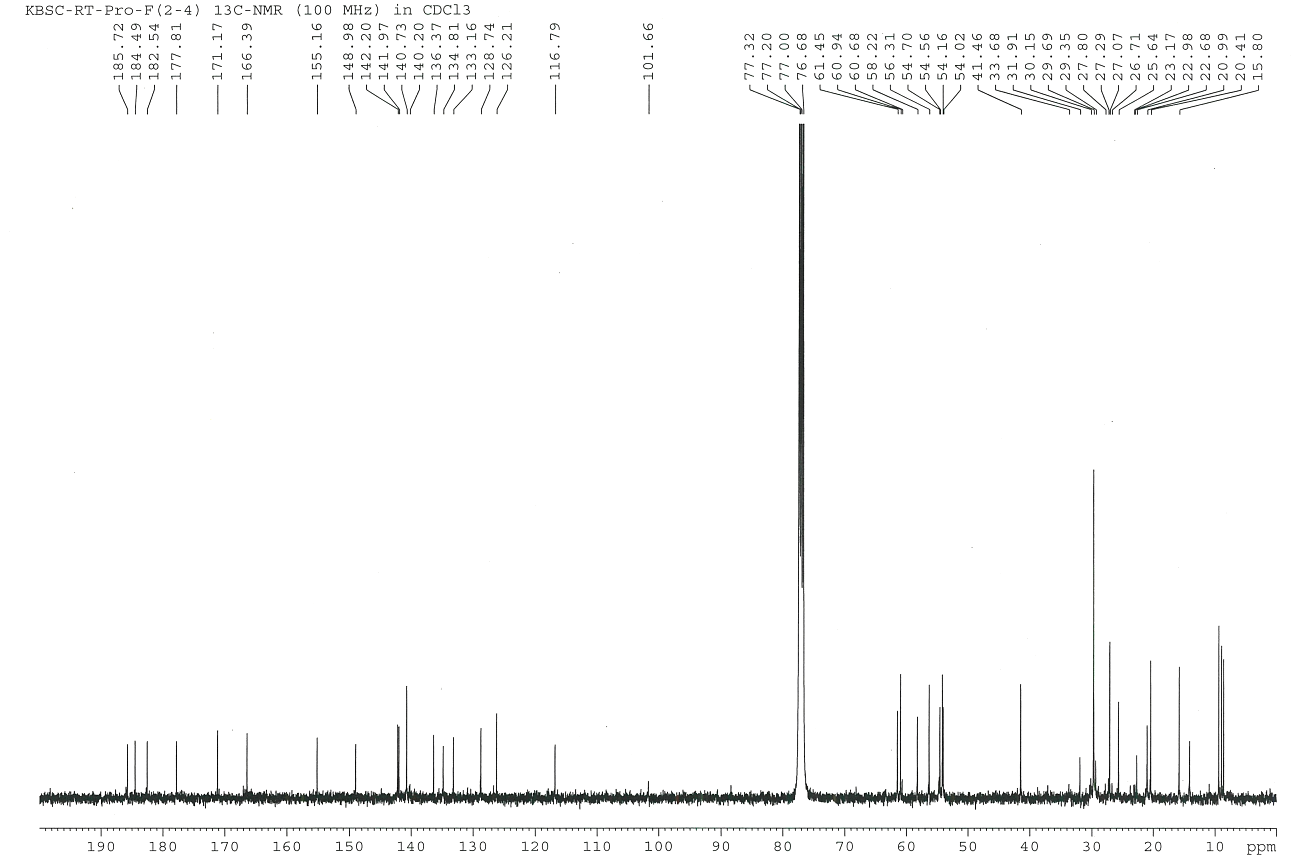


h

h

g

h

g: grease, h: hexane

**Figure S81.** ^13^C NMR (100 MHz) spectrum of **4a** in CDCl_3_


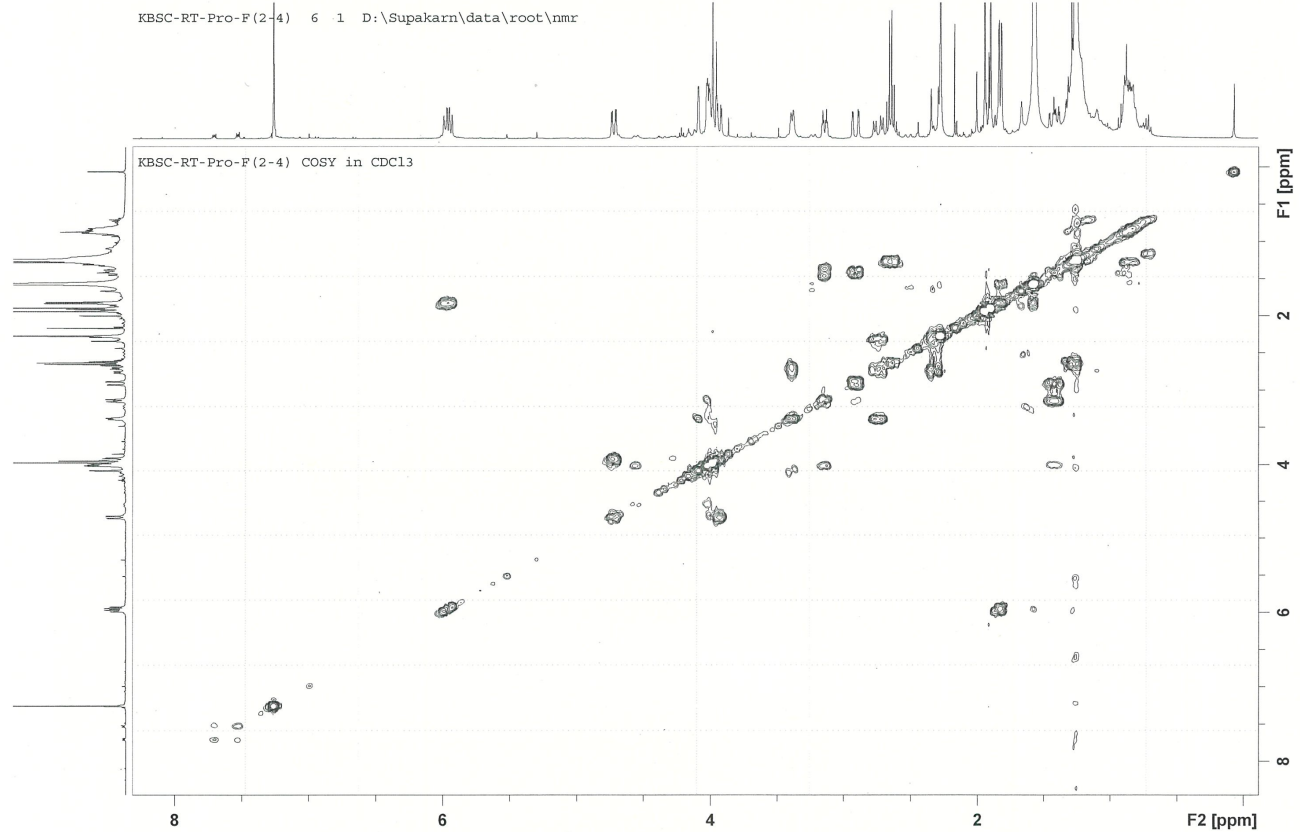


**Figure S82.** COSY (400 MHz) spectrum of **4a** in CDCl_3_


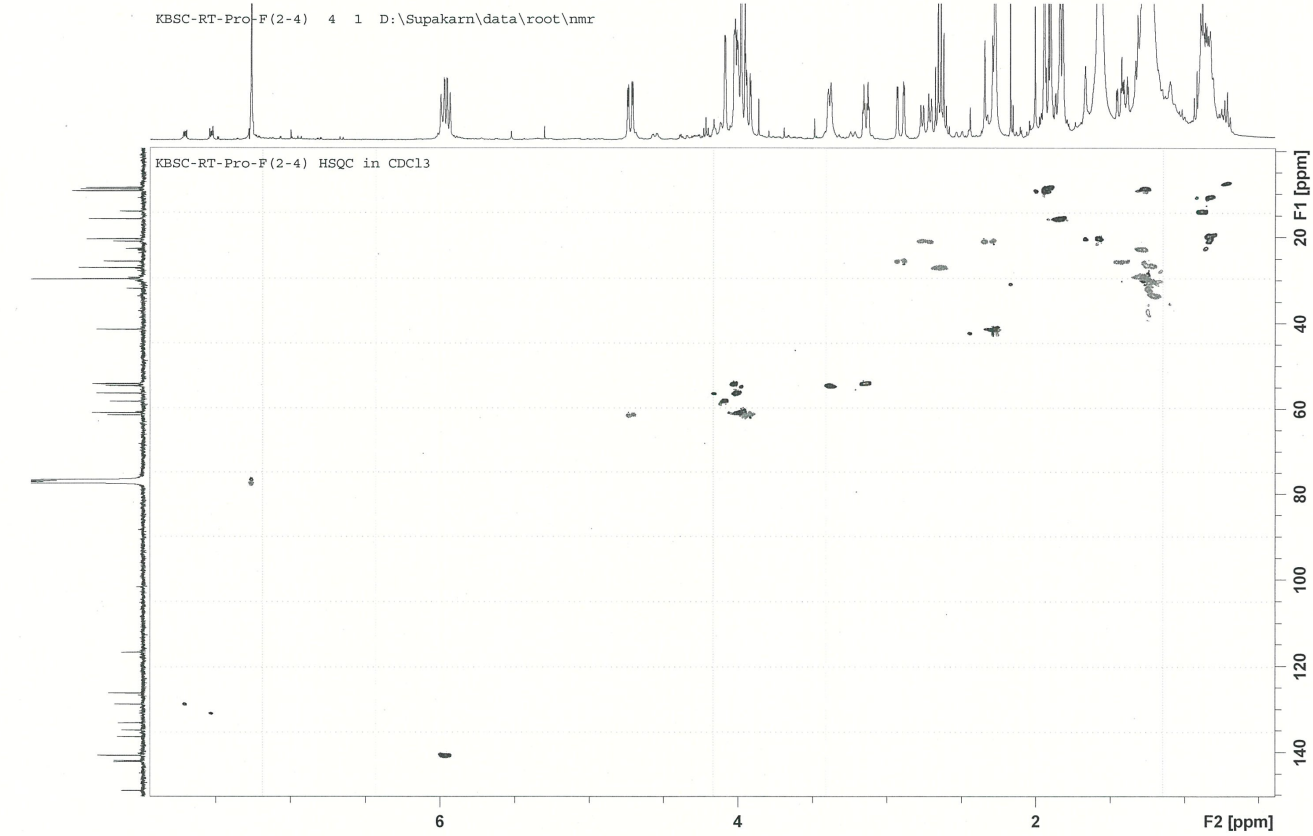


**Figure S83.** HSQC (400 MHz) spectrum of **4a** in CDCl_3_


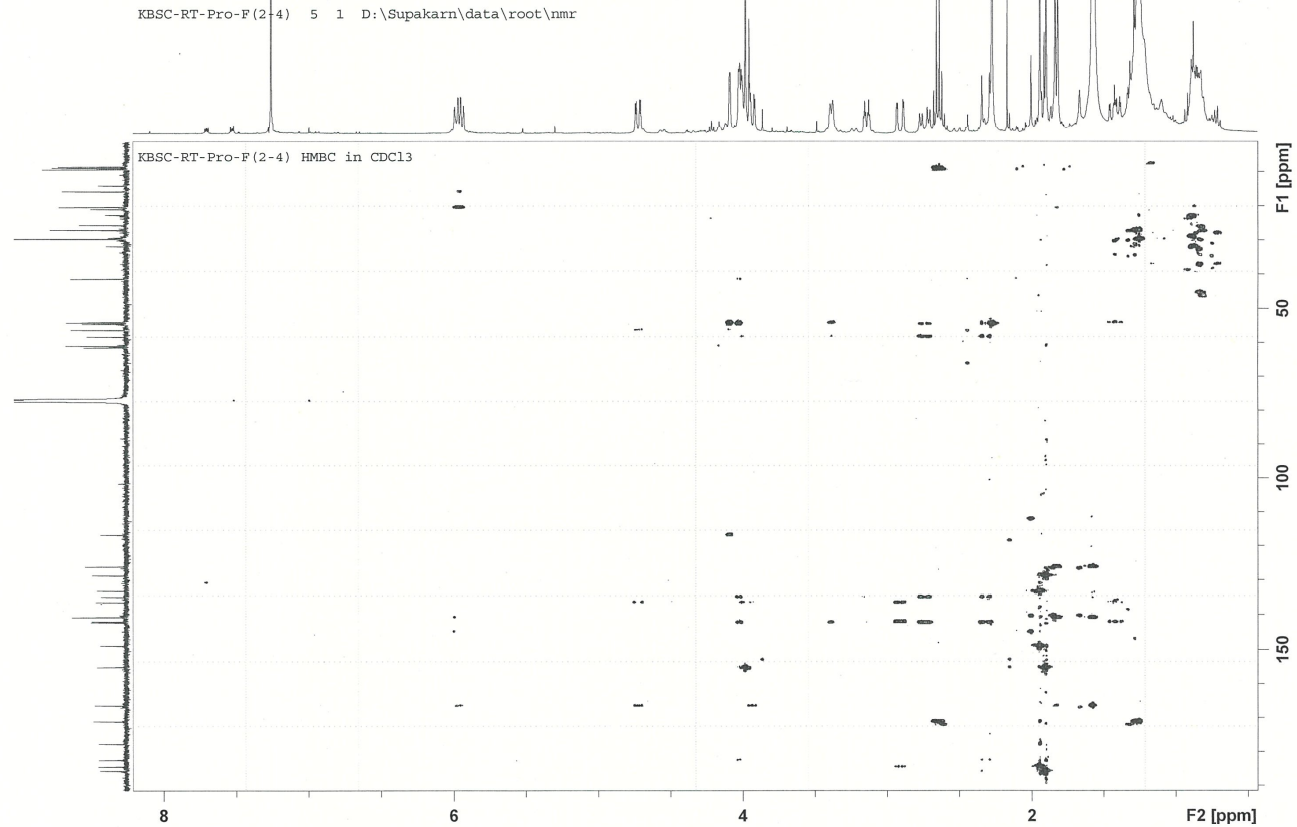


**Figure S84.** HMBC (400 MHz) spectrum of **4a** in CDCl_3_

# Physical and spectroscopic data of **4b**

*7-O-(3-pyridinecarbonyl) ester derivative of renieramycin S* **4b**: The title compound was synthesized from **2** (24.2 mg, 0.04 mmol), DMAP (12.8 mg, 0.11 mmol), EDCI (19.9 mg, 0.11 mmol) and nicotinoyl chloride hydrochloride (37.4 mg, 0.21 mmol) to afford **4b**; yield 33% (brsm); yellow amorphous powder; $[]_{D}^{25}$ +39.9 (*c* 0.33, CHCl_3_); ECD Δ*ε* (*c* 24.00 μM, methanol, 20 ^o^C) −2.6 (312), +9.8 (259), −7.0 (227), −5.2 (219), +4.0 (208), −4.6 (206), −21.5 (201), +4.6 (198), +30.2 (193) nm; IR (ATR) ν_max_ 2925, 2852, 1748, 1715, 1652, 1455, 1230, 1147, 1080, 728 cm^−1^; ^1^H NMR (CDCl_3_, 400 MHz) δ 9.36 (1H, s, 3'-H), 8.90 (1H, dd, *J* = 4.9, 1.6 Hz, 5'-H), 8.48 (1H, dt, *J* = 7.9, 1.6 Hz, 7'-H), 7.57 (1H, dd, *J* = 7.9, 4.9 Hz, 6'-H), 5.98 (1H, qd, *J* = 7.2, 1.4 Hz, 26-H), 4.72 (1H, dd, *J* = 11.8, 3.0 Hz, 22-H_α_), 4.10 (1H, br d, *J* = 2.3 Hz, 21-H), 4.05 (1H, dd, *J* = 2.7, 0.9 Hz, 11-H), 4.02 (1H, overlapped, 1-H), 4.00 (3H, s, 17-OCH_3_), 3.97 (1H, dd, *J* = 11.8, 3.0 Hz, 22-H_β_), 3.42 (1H, br d, *J* = 7.4 Hz, 13-H), 3.19 (1H, dt, *J* = 11.2, 2.7 Hz, 3-H), 2.96 (1H, dd, *J* = 17.5, 2.7 Hz, 4-H_α_), 2.76 (1H, dd, *J* = 21.0, 7.4 Hz, 14-H_α_), 2.34 (1H, d, *J* = 21.0 Hz, 14-H_β_), 2.30 (3H, s, NCH_3_), 2.04 (3H, s, 6-CH_3_), 1.91 (3H, s, 16-CH_3_), 1.84 (3H, dq, *J* = 7.2, 1.4 Hz, 27-CH_3_), 1.60 (3H, dq, *J* = 1.5, 1.5 Hz, 28-CH_3_), 1.47 (1H, ddd, *J* = 17.5, 11.3, 2.7 Hz, 4-H_β_); ^13^C NMR (CDCl_3_, 100 MHz) δ 185.7 (C-15), 184.2 (C-5), 182.6 (C-18), 177.4 (C-8), 166.4 (C-24), 161.7 (C-1'), 155.2 (C-17), 153.4 (C-5'), 150.5 (C-3'), 148.7 (C-7), 142.3 (C-20), 142.3 (C-10), 140.9 (C-26), 139.0 (C-7'), 136.6 (C-9), 134.7 (C-19), 134.0 (C-6), 128.8 (C-16), 126.2 (C-25), 124.5 (C-2'), 124.1 (C-6'), 116.8 (21-CN), 61.5 (C-22), 61.0 (17-OCH_3_), 58.2 (C-21), 56.4 (C-1), 54.6 (C-13), 54.2 (C-11), 54.0 (C-3), 41.5 (NCH_3_), 25.7 (C-4), 21.1 (C-14), 20.4 (28-CH_3_), 15.8 (27-CH_3_), 9.7 (6-CH_3_), 8.6 (16-CH_3_); HRESIMS *m/z* 667.2399 ([M+H]^+^, calculated for C_36_H_35_N_4_O_9_, 667.2399).


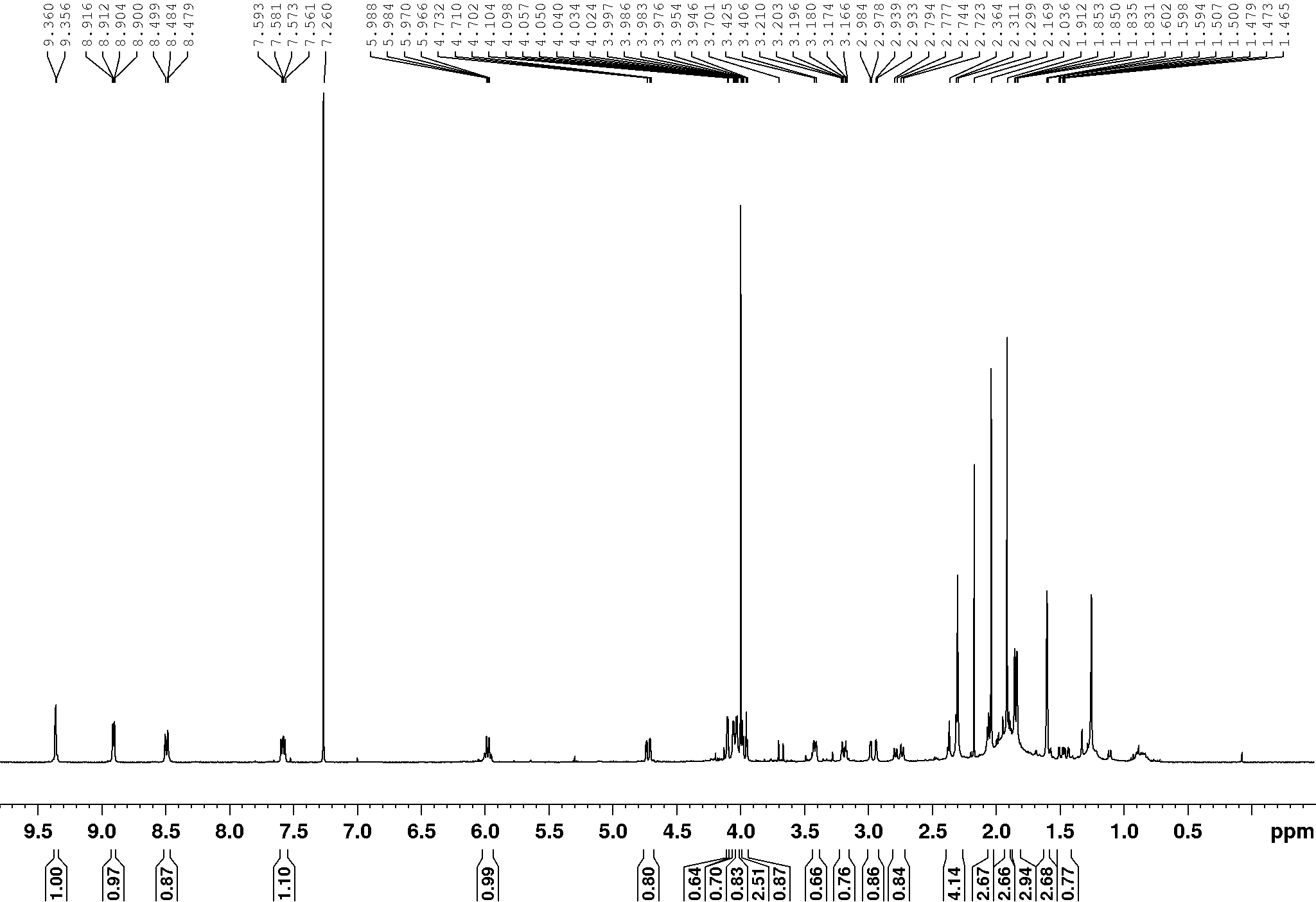


g: grease, h: hexane

g, h

g, h

**Figure S85.** ^1^H NMR (400 MHz) spectrum of **4b** in CDCl_3_


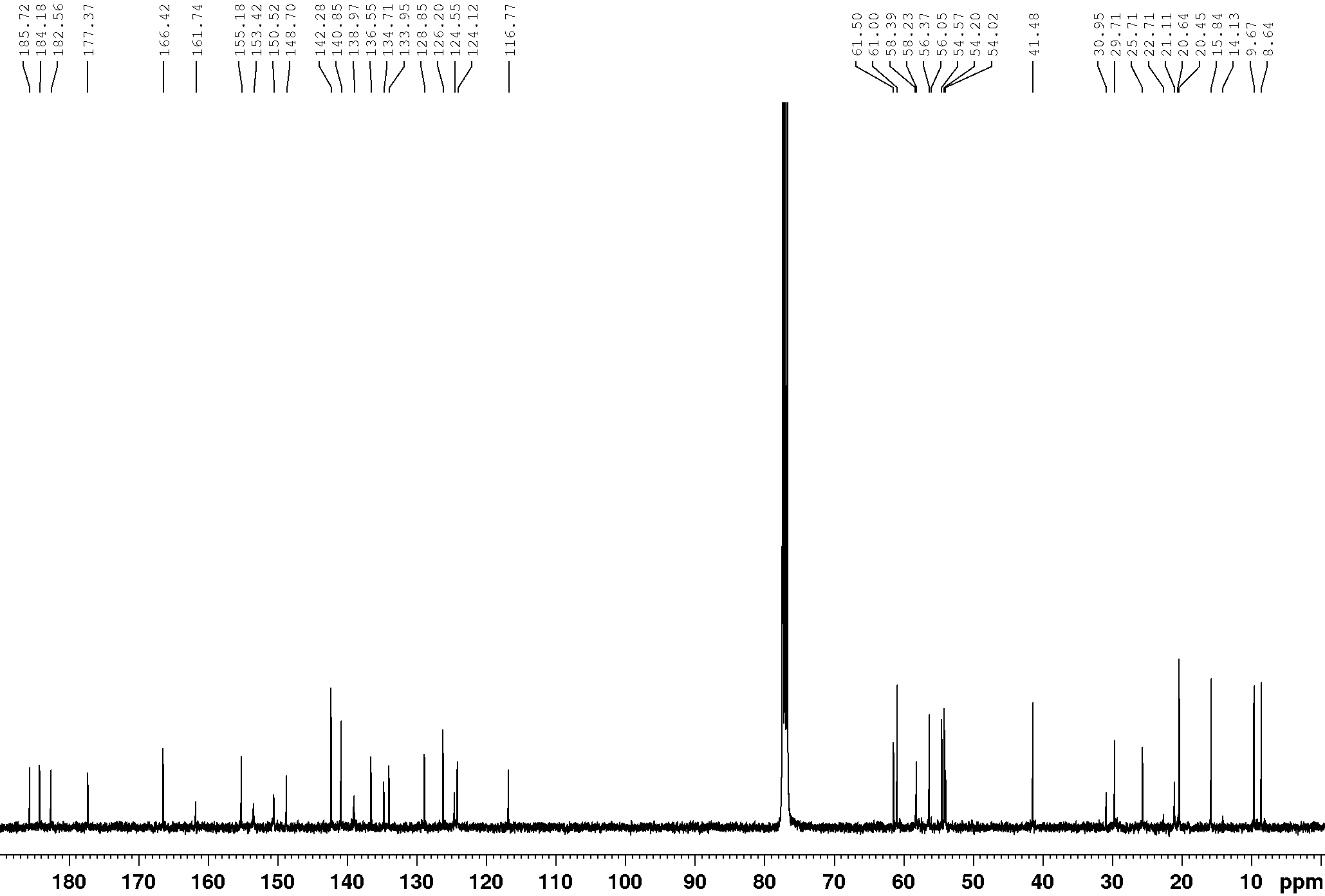


h

g: grease, h: hexane

h h

g

**Figure S86.** ^13^C NMR (100 MHz) spectrum of **4b** in CDCl_3_


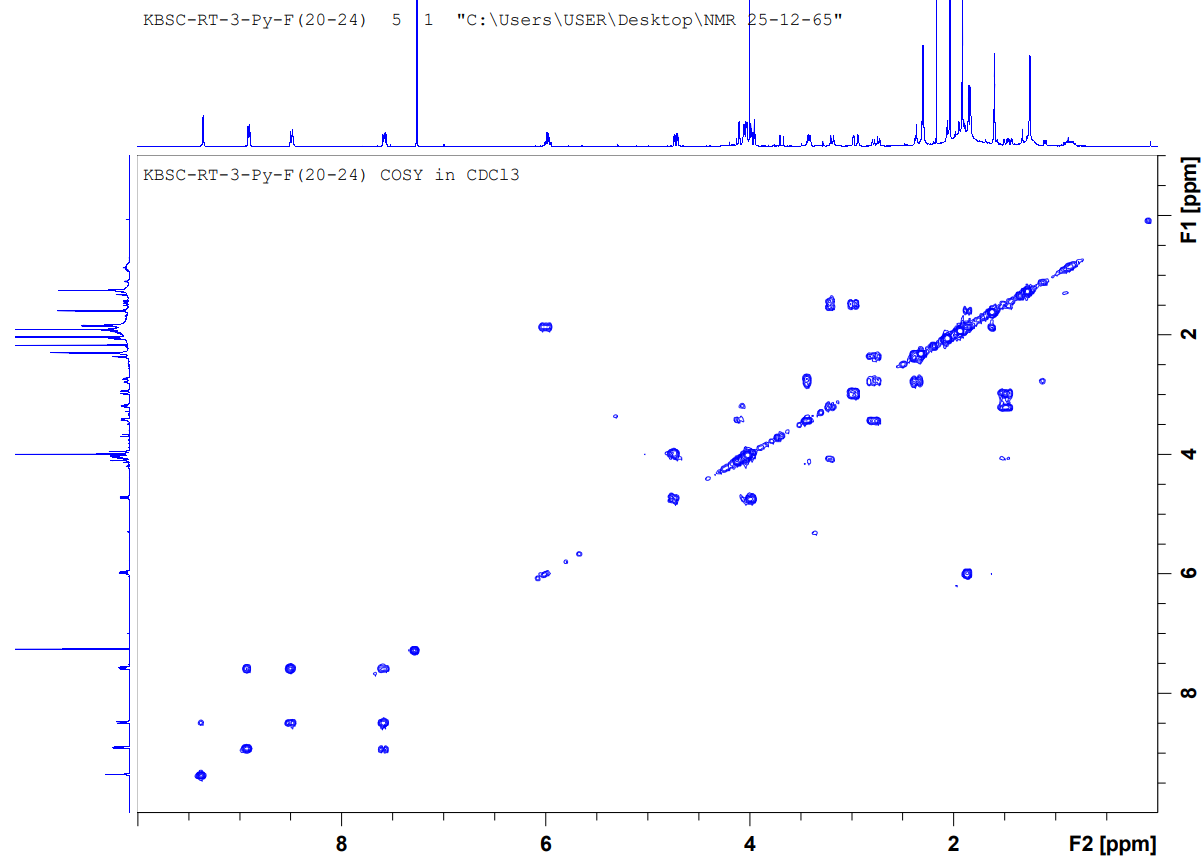


**Figure S87.** COSY (400 MHz) spectrum of **4b** in CDCl_3_


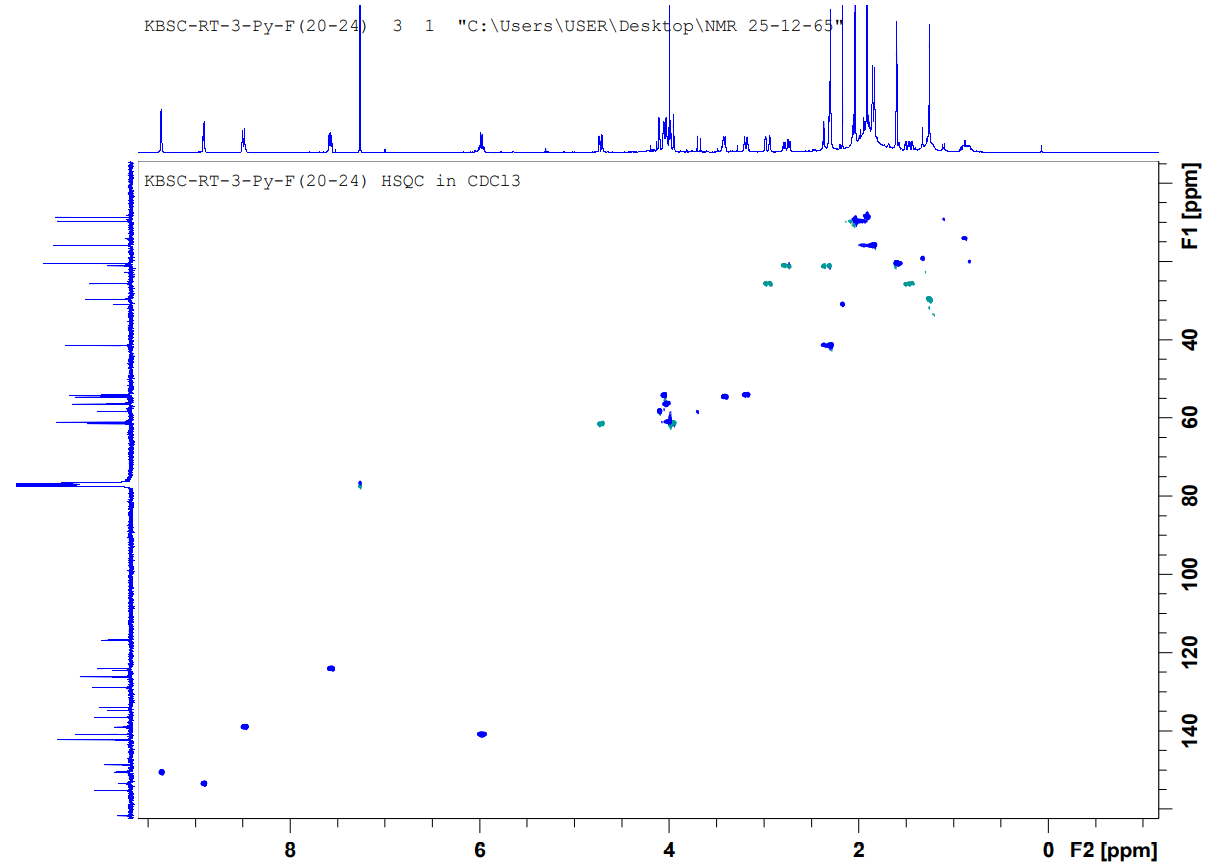


**Figure S88.** HSQC (400 MHz) spectrum of **4b** in CDCl_3_


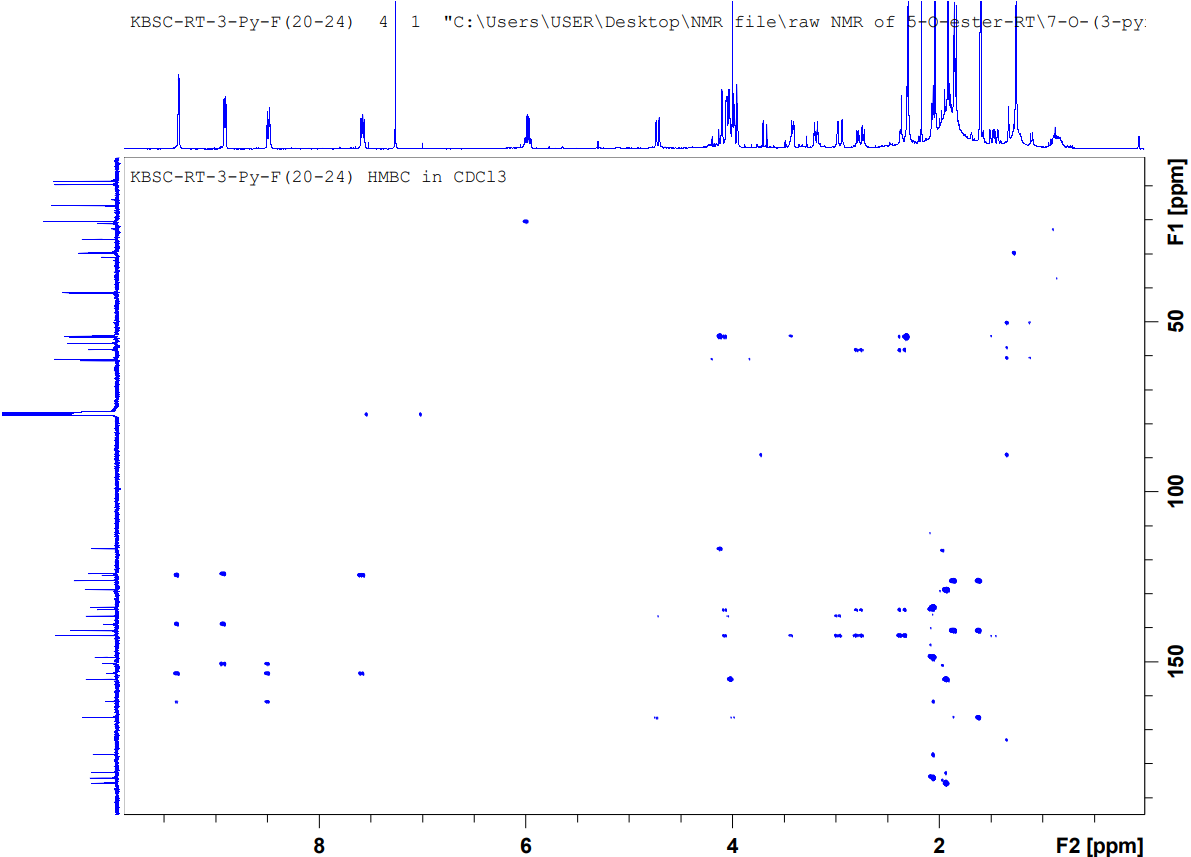


**Figure S89.** HMBC (400 MHz) spectrum of **4b** in CDCl_3_

**NOTE**

Peak of impurities; Hexane (^1^H NMR (CDCl_3_, 400 MHz) δ 1.26 (m), 0.88 (t); ^13^C NMR (CDCl_3_, 100 MHz) δ 29.8 (CH_3_), 22.7 (CH_2_), 14.1 (CH_3_)), Grease (^1^H NMR (CDCl_3_, 400 MHz) δ 1.26 (br s), 0.86 (m); ^13^C NMR (CDCl_3_, 100 MHz) δ 29.8 (CH_2_)), Acetone (^1^H NMR (CDCl_3_, 400 MHz) δ 2.06 (br s); ^13^C NMR (CDCl_3_, 100 MHz) δ 207.0 (C), 30.9 (CH_3_)), and Water (^1^H NMR (CDCl_3_, 400 MHz) δ 1.56

**REFERENCES**

(20) Daikuhara, N.; Tada, Y.; Yamaki, S.; Charupant, K.; Amnuoypol, S.; Suwanborirux, K.; Saito, N. Chemistry of renieramycins. Part 7: Renieramycins T and U, novel renieramycin–ecteinascidin hybrid marine natural products from Thai sponge *Xestospongia* sp. *Tetrahedron Lett.* **2009**, *50* (29), 4276-4278. DOI: 10.1016/j.tetlet.2009.05.014.

(35) Suksamai, D.; Racha, S.; Sriratanasak, N.; Chaotham, C.; Aphicho, K.; Lin, A. C. K.; Chansriniyom, C.; Suwanborirux, K.; Chamni, S.; Chanvorachote, P. 5-*O*-(*N*-Boc-l-Alanine)-Renieramycin T Induces Cancer Stem Cell Apoptosis via Targeting Akt Signaling. *Mar. Drugs* **2022**, *20* (4), 235. DOI: 10.3390/md20040235.

**Table S1.** Cytotoxicity of 5-O-ester derivatives of renieramycin T (**3a**−**3o**) and 7-O-ester derivatives of renieramycin S (**4a** and **4b**) against other non-lung cell lines: human keratinocyte (HaCaT) and non-tumorigenic bronchial epithelial (BEAS-2B) cell lines.

| entry | compound | 5-*O*-substituent | IC_50_ ± S.D. (nM) | |
| --- | --- | --- | --- | --- |
|  |  |  | HaCaT | BEAS-2B |
| 1 | **1** | 5,8-dicarbonyl | 8.01 ± 0.53 | 74.95 ± 9.77 |
| 2 | **2** | H | 89.16 ± 4.29 | 162.70 ± 24.18 |
| 3 | **3a** | acetyl | 9.49 ± 2.12 | >250 |
| 4 | **3b** | propanoyl | 33.69 ± 9.43 | 90.20 ± 9.49 |
| 5 | **3c** | *N*-Boc-L-glycinoyl | 23.15 ± 1.38 | >250 |
| 6 | **3d** | *N*-Boc-L-alaninoyl | 58.51 ± 6.68 | 232.33 ± 26.11 |
| 7 | **3e** | *N*-Boc-L-valinoyl | 80.18 ± 4.10 | >250 |
| 8 | **3f** | *N*-Boc-L-phenylalaninoyl | 61.17 ± 3.43 | 180.65 ± 12.09 |
| 9 | **3g** | 2-pyridinecarbonyl | 74.34 ± 6.88 | >250 |
| 10 | **3h** | 4-pyridinecarbonyl | 53.81 ± 7.07 | 222.70 ± 23.05 |
| 11 | **3i** | 2-quinolinecarbonyl | 27.96 ± 3.76 | 150.77 ± 19.03 |
| 12 | **3j** | 3-quinolinecarbonyl | 108.93 ± 7.38 | >250 |
| 13 | **3k** | 3-pyridine acryloyl | 9.64 ± 0.93 | 114.10 ± 13.17 |
| 14 | **3l** | 3-indolecarbonyl | 109.27 ± 6.99 | >250 |
| 15 | **3m** | 2-furancarbonyl | 42.06 ± 3.13 | >250 |
| 16 | **3n** | 2-thiophenecarbonyl | 138.67 ± 6.16 | >250 |
| 17 | **3o** | 2-pyrrolecarbonyl | 65.16 ± 1.52 | 220.20 ± 29.72 |
| 18 | **4a**^a^ | 5,8-dicarbonyl | 229.60 ± 22.71 | >250 |
| 19 | **4b**^b^ | 5,8-dicarbonyl | 191.93 ± 22.22 | >250 |
| 20 | cisplatin | - | 14.33 × 10^3^ ± 2.05 × 10^3^ | >20 × 10^3^ |
| 21 | doxorubicin | - | 915.43 ± 50.21 | 0.663 × 10^3^ ± 0.12 × 10^3^ |

HaCaT and BEAS-2B was tested for 24 h.

^a^ 7-*O*-(propanoyl) ester derivative of renieramycin S (**4a)**

^b^ 7-*O*-(3-pyridinecarbonyl) ester derivative of renieramycin S (**4b**)
